# Recent developments in the synthesis of regioregular thiophene-based conjugated polymers for electronic and optoelectronic applications using nickel and palladium-based catalytic systems

**DOI:** 10.1039/c9ra09712k

**Published:** 2020-01-27

**Authors:** Bibi Amna, Humaira Masood Siddiqi, Abbas Hassan, Turan Ozturk

**Affiliations:** Department of Chemistry, Quaid-i-Azam University Islamabad 45320 Pakistan; Istanbul Technical University, Department of Chemistry 34469 Maslak Istanbul Turkey ozturktur@itu.edu.tr; TUBITAK-UME, Chemistry Group of Laboratories PO Box 54 Gebze Kocaeli 41471 Turkey

## Abstract

Thiophene-based conjugated polymers hold an irreplaceable position among the continuously growing plethora of conjugated polymers due to their exceptional optical and conductive properties, which has made them a centre of attention for the past few decades and many researchers have contributed tremendously by designing novel strategies to reach more efficient materials for electronic applications. This review aims to highlight the recent (2012–2019) findings in design and synthesis of novel thiophene-based conjugated polymers for optical and electronic devices using organometallic polycondensation strategies. Nickel- and palladium-based protocols are the main focus of this account. Among them nickel-catalyzed Kumada catalyst-transfer polycondensation, nickel-catalyzed deprotonative cross-coupling polycondensation, palladium-catalyzed Suzuki–Miyaura and Migita–Kosugi–Stille couplings are the most popular strategies known so far for the synthesis of functionalized regioregular polythiophenes exhibiting fascinating properties such as electronic, optoelectronic, chemosensitivity, liquid crystallinity and high conductivity. This account also presents a brief overview of direct arylation polymerization (DArP) protocol that has shown a great potential to lessen the drawbacks of conventional polymerization techniques. DArP is a cost-effective and green method as it circumvents the need for the synthesis of arylene diboronic acid/diboronic ester and distannyl arylenes using toxic precursors. DArP also puts off the need to preactivate the C–H bonds, hence, presenting a facile route to synthesize polymers with controlled molecular weight, low polydispersity index, high regioregularity and tunable optoelectronic properties using palladium-based catalytic systems.

## Abstract 2

Thiophene-based conjugated polymers are important conjugated polymers due to their exceptional optical and conductive properties, over the past few decades many researchers have designed novel strategies to reach more efficient materials for electronic applications.

## Graphical abstract

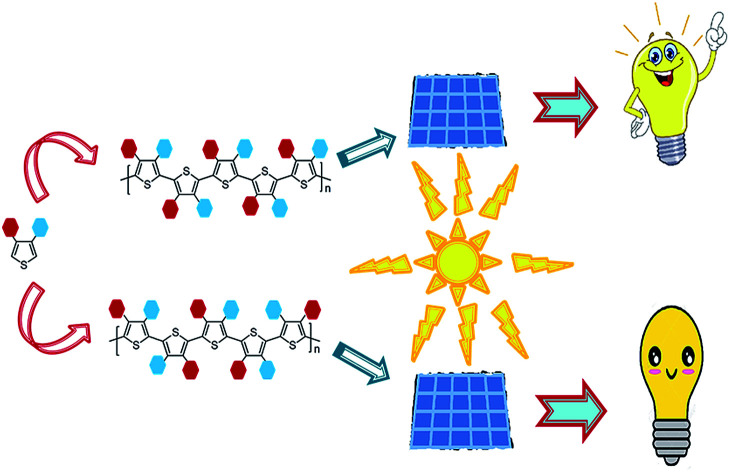

## 1. Introduction

Conjugated polymers are macromolecules with an unsaturated backbone containing alternating single and double/triple bonds. An extended system of delocalized π-electrons is created by their overlapping π-orbitals, which induce many useful and interesting electrical and optical properties.^1–5^ Owing to their tremendous conducting properties these polymers have been utilized in a broad spectrum of applications including organic thin-film transisters,^6^ conductive polymers^7^ sensors for organic,^8,9^ inorganic,^10–12^ and biological materials,^13,14^ photovoltaic cells,^15,16^ electron beam lithography^17^ and optically active films and solutions of chiral polymers.^18^ Up till now a wide variety of conjugated polymers such as poly(pyrrole)s,^19–22^ poly(benzoazole)s,^23,24^ poly(thiophene)s,^25–29^ poly(cyclopentadithiophene)s,^30^ poly(fluorine)s,^31,32^ poly(carbazole)s,^21^ poly(arylene-ethynylene)s,^33–36^ poly(selenophene)s,^37,38^ poly(benzotriazole)s,^39,40^ and poly(phenylene)s^41,42^ and their derivatives have been synthesized and their potential applications in optical and electrical devices have been evaluated. A useful feature of conjugated polymers is their tunability. The properties of these polymers can be tailored by use of different substituents, alternating backbone units and incorporating various heteroatoms. Number of powerful methodologies has been developed to access conjugated polymers including widely used cross-coupling strategies.^43,44^

### 1.1. Thiophene-based conjugated polymers

Polythiophenes represent one of the most important classes of conjugated donor polymers useful for wide range of applications such as field-effect transistors,^25,45–49^ plastic solar cells,^1,7,50–53^ light emitting diodes^1,7,50,51,53,54^ and conductive poymers^1,7,51,55,56^ due to their exceptional electrical and optical properties as well as excellent chemical and thermal stability.^51,57–60^ The control of the energy bandgaps (HOMO–LUMO energy levels) has been a central goal of the synthetic chemistry of functional π-conjugated systems. The energy levels and absorption properties of the polythiophenes can be attuned by introduction of conjugated substituents into their backbone or into the side chains. Poly(3-hexylthiophene) (P3HT) is a most commonly used material among a broad class of polythiophenes due to its good processability, high charge carrier mobility and above all, easy synthesis.^61^ Unfortunately, the main problem with P3HT is its high HOMO level and large optical band gap (*E*^opt^_g_ ∼ 1.9 eV) which causes inadequate absorption in visible region and poor *V*_oc_ values of fabricated devices.^53,62–64^ Several synthetic strategies have been developed to address these drawbacks including introduction of conjugated side chains as substituents on the polymer main chain,^65–74^ synthesis of fused and rigid planar rings to stabilize the quinoid resonance structure^75–89^ and construction of the backbone consisting of electron donating thiophene units alternated with some electron withdrawing unit to develop donor–acceptor type polymers.^80–84^

### 1.2. Significance of the substitution on thiophene monomer

Unsubstituted polythiophenes were synthesized *via* chemical polymerization at the initial stages of the history of polythiophenes.^85–89^ These unsubstituted polythiophenes were thermally stable and highly conductive but were insoluble.^90,91^ In order to prepare soluble polythiophenes, alkyl substituents were introduced at position-3 of the thiophene unit which was then polymerized using the protocol previously used for the synthesis of unsubstituted polythiophenes in the late 1980s.^92–96^ However, these chemical and electrochemical polymerization techniques resulted in the random couplings in the poly(3-alkylthiophenes) (P3AT) yielding only 50–80% head to tail couplings due to the multiple head–head and tail–tail couplings. Head-to-tail regioregular P3ATs were synthesized for the very first time using McCullough method in 1992.^97^ While it enhanced the electrical conductivity of the polymer due to the formation of well-organized three-dimensional polycrystalline structure, this method also paved the way for the synthesis of many other functionalized polythiophenes.

### 1.3. Effects of various functional groups on the properties of polythiophenes

To date many functionalize polythiophenes have been synthesized consisting of thiophene monomers with various substituents at 3-position. While different alkyl substituents are the most commonly used, the use of esters (–COOR),^98,99^ acetyl (–COR),^100^ amide (–CONHR),^101^ alkoxy (–OR),^102–105^ alkylthio (–SR),^106–108^ sulfonyl (–SO_3_R),^109^ alkylamino (–NHR and NRR′)^110^ and fluoroalkyl^111–119^ groups have also been reported. It has now been established that functionalization of polythiophenes not only enhanced their solubility and processability, it also altered their optical and electronic properties. In addition to this, the side chains of the polymers were reported to be helpful in the chemical sensing as molecular recognition units.^120,121^

#### 1.3.1. Substituents with electron withdrawing inductive effect

Introduction of ester group at position-3 of thiophene monomer resulted in a blue shift in *λ*_max_ compare with that of P3ATs, which might be due to the wider band gap resulting from the electron-withdrawing nature of the carbonyl moiety.^98^ Polythiophenes substituted with partially fluorinated alkyl chains exhibited unique properties including chemical and oxidative resistance, and hydrophobicity. Such polymers formed highly ordered solid-state structure and were also reported to show liquid crystalline behavior.^122,123^ Regioregular polythiophenes (rrPT) with ether substituents, where oxygen is not directly connected to the ring, exhibited ion-binding properties toward Pb^2+^, Hg^2+^ and Li^+^. These polymers showed very high conductivities after iodine doping.^124^

#### 1.3.2. Substituents with electron donating inductive effect

Introduction of electron-donating groups presented several advantages over alkyl substituted ones. For instance, alkoxy group, with a heteroatom directly attached to the thiophene ring resulted in a decreased band gap by raising their HOMO level, which resulted in low oxidation potential, and the conducting state in the polythiophene was stabilized.^124^ rrPT substituted with alkylthio groups showed low solubility in common organic solvents such as THF, xylene and chloroform, while they were fairly soluble in carbon disulfide.^55^ These polymers exhibited high conductivity of 100 S cm^−1^ after iodine-doping.^125^ Relatively fewer polythiophenes with 3,4-alkylthio substituents have been studied so far. One of the most interesting properties of these polythiophenes that distinguished them from others is the complexing ability of sulfur atoms of thioether groups towards various “soft” metallic ions. This capability of alkylthio substituted polythiophenes introduced the possibility of using the sensitivity of optoelectronic properties of these polymers to develop sensor systems to detect the presence of species that may alter these properties. Such compounds may therefore be used as catalysts for the hydroformylation reaction or as active site of the chemosensors.^126–128^

### 1.4. Polythiophenes substituted with chiral side chains

π-Conjugated polymers substituted with chiral side chains represent a special class of optically active polymers that has attracted much attention in view of their possible applications as enatio-selective membranes and electrodes, suitable for chiral sensing as well as electrochemical asymmetric synthesis.^129–131^ Side chain chirality imparts helical order in solid state and in aggregates. Moreover, chiral side chains have great ability to self-assemble into novel nanostructures.^132,133^ In general, these chiral polymers display strong chiroptical properties only when their chains aggregate to form chiral superstructures. Significant optical activity could be induced into polythiophenes provided the substituent at position-3 of the thiophene monomers are placed in a regioregular head to tail fashion. This induced optical activity is either the result of an intermolecular chiral orientation of the predominantly co-planar chains of the polymer with a kind of super-coiling in crystalline hexagonal phase or result of an intramolecular helical conformation of the polymer backbone.^134^ Chiral aggregations in optically active polythiophenes are reported to be influenced with slight differences in the structure such as regioregularity and substituents as well as processing conditions, *e.g.* solution temperature,^135,136^ solvent^137,138^ and solvent additives.^139–141^ The polymer solution consisting of chiral regioregular polythiophenes showed strong circular dichroism (CD) signal at its π–π* transition upon addition of poor solvent or at low temperature suggesting the formation of helical backbone in the aggregate,^142,143^ although few conjugated polymers have also been reported to self-assemble into aggregates even in chloroform and chlorobenzene that are considered as “good” solvents.^144^

### 1.5. Regioregular polythiophenes

**Scheme 1:**
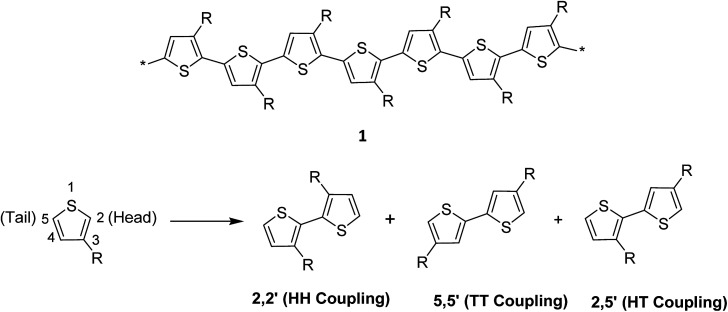
Three possible coupling modes of 3-alkylthiophene units.

Thiophene, being a five-membered ring, polymerizes at position 2 and 5 producing directionality in the resulting polymer 1. Whenever a thiophene monomer is incorporated in a growing polymer chain, the monomer unit can be added either at position 2 (head) or position 5 (tail) first. However, a mixture of all the three possible couplings usually forms in a regiorandom poly(3-alkylthiophene) (Scheme 1).

### 1.6. Advantages of regioregular polythiophenes over regiorandom analogues

The regioregularity in the polymers has led to the increased crystallinity that in turn resulted in enhanced conductivities.^145^ The synthesis of regioregular polythiophenes has produced structurally homogenous and defect-free polymers that have greatly improved photonic and electronic properties as compared to regioirregular ones.^146,147^ Regioregular polythiophenes have led to a large number of novel and important micro- and nano-scale electronic materials and devices.^6,148–153^ The random sequence of monomer units in polythiophenes hinders the close packing of the polymer chains and reduces the electrical conductivity due to the twisting of thiophene rings out of the conjugation planarity resulting from the steric repulsion among the substituted alkyl chains. Self-assembled polythiophenes exhibit better performance in electrical and optical devices as compared to regiorandom polythiophenes.^144,154^ Sterically twisted structure is produced in the polythiophene backbone due to the multiple head–head (HH) and tail–tail (TT) couplings, resulting in the loss of extended π-conjugation 2. This sterically twisted back bone leads to the reduction of high conductivity and other desirable properties in polythiophenes.
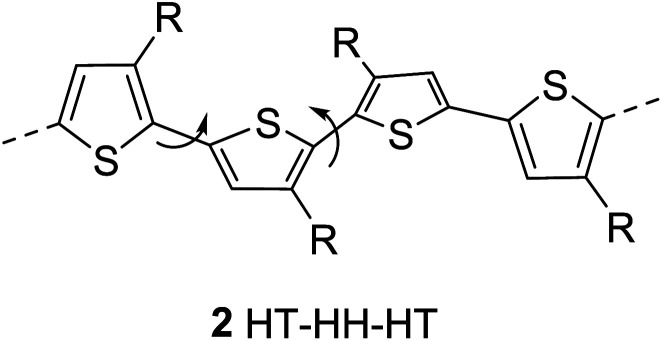


Regioregular polythiophenes possess excellent electrical properties due to their planar backbone and solid-state self-assembly that form well-organized three-dimensional polycrystalline structure, making them highly conductive due their efficient interachain and interchain charge carrier pathways.^90,154^ For instance, the mobilities of regioregular poly(3-alkylthiophenes) are higher than those of regioirregular poly(3-alkylthiophenes).^6,155,156^ The solution of regioregular polythiophenes, containing chiral side chains, show a strong circular dichroism signal due to the formation of helical backbone in aggregates, whereas regioirregular polythiophenes show only a weak CD signal. Head to tail (HT) polymers are superior compare with their head to head (HH) and regiorandom isomers and possess higher field-effect mobility and light-emitting ability.^157,158^

## 2. Transition metal catalyzed synthesis of thiophene-based polymers

Perfect control over the incorporation of each thiophene unit in a consecutive head to tail manner is important for the synthesis of regioregular polythiophenes, and employment of transition metal catalysts has effectively served this purpose since the first synthesis of regioregular head-to-tail coupled poly(3-alkylthiophene) (P3ATs) by McCullough early in 1992 using nickel catalyst.^97^

### 2.1. Historical background

In 1980, Yamamoto and co-workers succeeded in synthesizing the first regular poly(thiophene-2,5-diyl) *via* nicke-catalyzed Kummada-type polymerization of 2,3-dibromothiophene, and it was found that these polythiophenes had interesting properties, conductivity of which increased by a factor of 10^7^ upon iodine doping. They were highly stable under ambient conditions.^85^

**Scheme 2:**
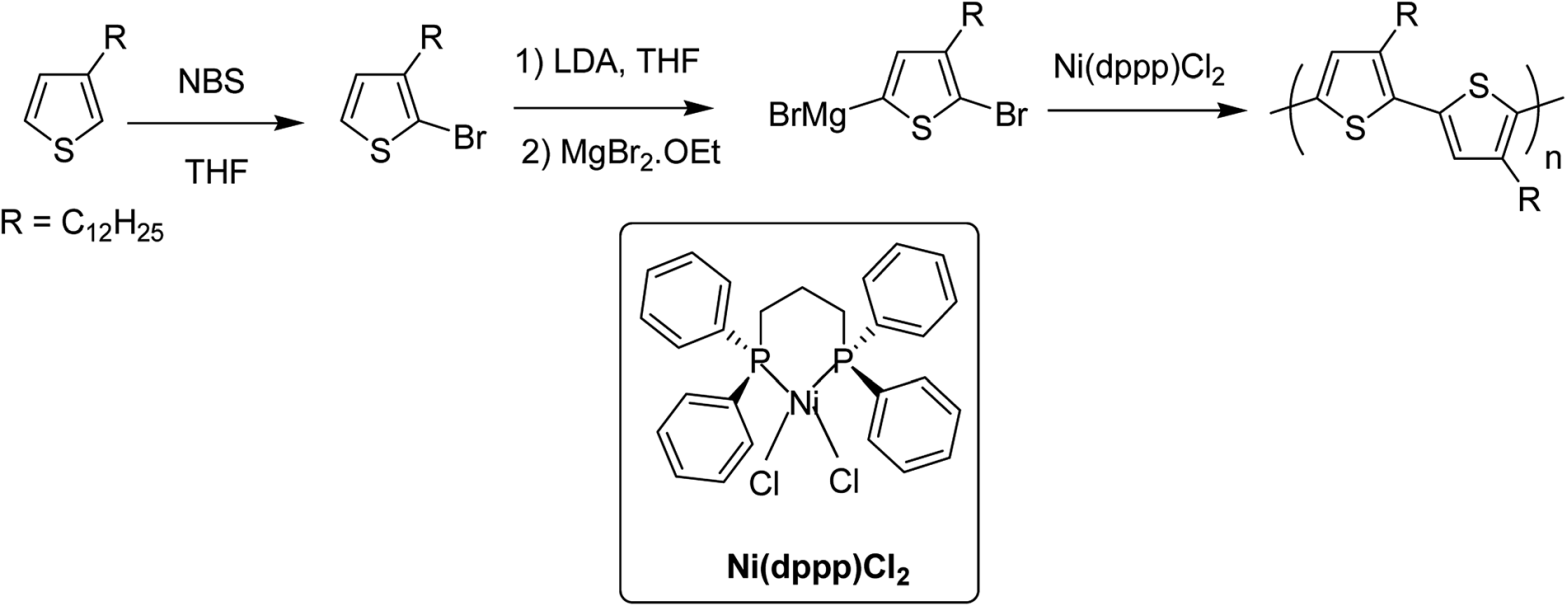
The original McCullough method for the synthesis of poly(3-alkylthiophene)s.

**Scheme 3:**
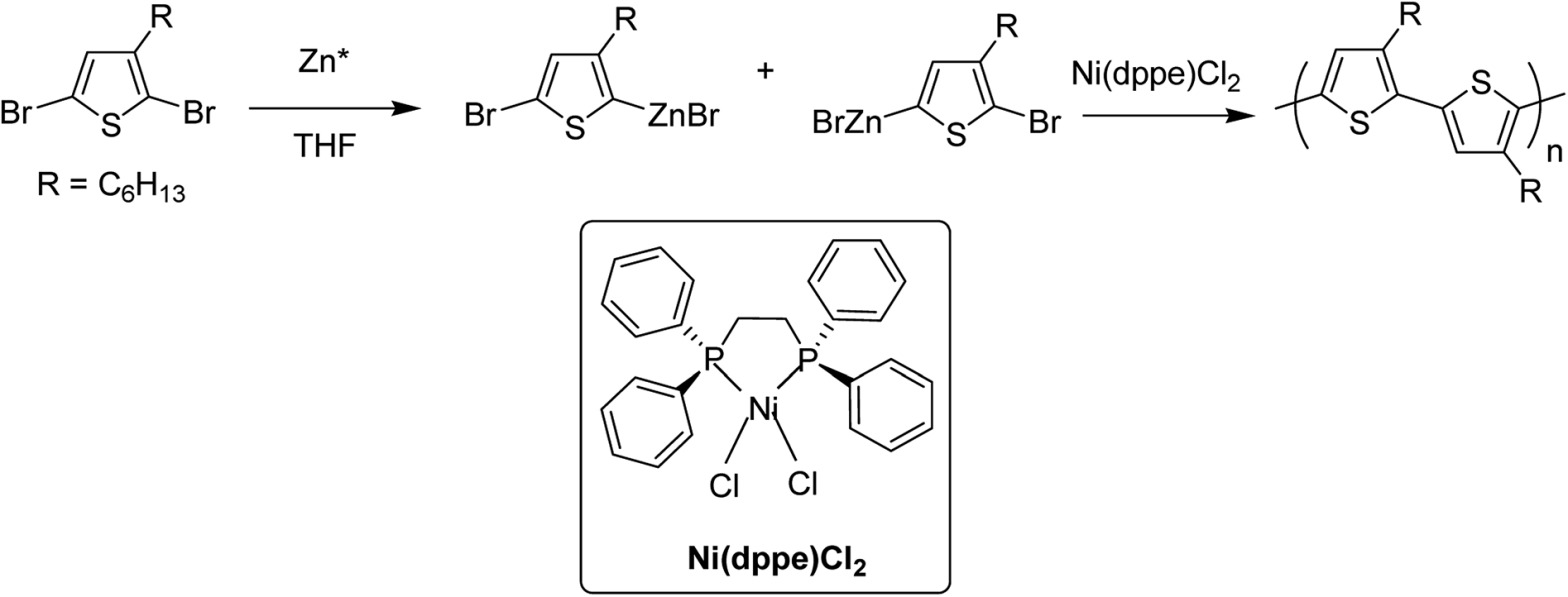
The original Rieke method for the synthesis of poly(3-alkylthiophene)s.

In 1992, McCullough achieved regioregular synthesis of poly(3-alkylthiophene) with 91% HT–HT coupling (Scheme 2). In the same year, Rieke and Chen synthesized regioregular poly(3-alkylthiophene) using a different approach, exploiting regioselectivity rather than regiospecificity.^159^ When 3-alkyl-2,5-dibromothiophene was treated with highly reactive Rieke zinc (Zn*), 3-alkyl-2-bromo-5-(bromozincio)thiophene and 3-alkyl-5-bromo-2-(bromozincio)thiophene were yielded in 9 : 1 ratio (Scheme 3). The subsequent polymerization proved very sensitive to the type of catalyst used. Use of Ni(dppe)Cl_2_ resulted in 99% HT–HT couplings. On the other hand, completely regiorandom P3HTs were afforded using Pd(PPh_3_)_4_, whereas Pd(dppe)Cl_2_ resulted in 70% HT–HT coupling. In the following years, transition metal catalyzed cross-coupling reactions were exploited further in order to develop compatibility with various functional groups, which resulted in successful synthesis of many new polythiophenes.

Formation of conjugated polymers essentially lies in the efficient carbon–carbon bond formation between unsaturated carbons in aromatic monomer units. In addition to the oxidative and electrochemical polymerization,^159–161^ transition metal catalyzed cross-coupling reactions provide a particularly powerful tool for the synthesis of conjugated polymers.^162^ The reaction in general, involves oxidative addition of transition metal catalyst across C–X bond of an electrophile, which leads toward transmetallation with the main group metal of organometallic nucleophile. Finally, the C–C bond is formed *via* reductive elimination with a concomitant regeneration of active catalyst.

Most commonly used transition metal catalysts for polycondensation reactions are palladium and nickel-based catalysts, although some other metals have also been used. The organometallic nucleophiles employed in transition metal catalysts include stannyl reagents (Stille coupling),^163^ boron reagents (Suzuki–Miyaura coupling),^164^ copper (Sonagashira coupling)^165^ and Grignard reagents (Kumada–Corriu coupling).^166^ Thus, consecutive transformations in the catalytic cycle can be made in order to extend the conjugation lengths. Regioregularity of the polymer can be achieved easily when nucleophilic and electrophilic centers of monomeric units are readily accessible. The enhancement of regioregularity through these advanced metal-catalyzed methodologies leads to various beneficial outcomes such as an intensified extinction coefficient, an increase in the mobility of the charge carriers and a red shift in absorption in solid state.

Since the advent of the concept of regioregular polythiophenes and an understanding of their useful properties and their utilization in various potential applications, number of research groups put their efforts to synthesize novel thiophene-based monomers, and their subsequent polymerization to afford homopolymers as well as copolymers with some acceptor monomeric units.

A brief survey has been provided for the synthesis of differently substituted and fused polythiophenes, including recent contributions made by different research groups, regarding the synthesis and characterization of novel thiophene based-monomers and their subsequent polymerization using nickel and palladium based catalytic systems with a special emphasis on the recent advancement in Ni-catalyzed C–H functionalization polymerization, Ni-catalyzed Kumada catalyst-transfer polycondensation and Pd-catalyzed direct arylation polymerization approachs. The survey includes how they are helpful in addressing the drawbacks of widely used conventional cross-coupling polymerization protocols.

## 3. Nickel catalyzed synthesis of thiophene-based polymers

Since the first regioregular synthesis of 3-alkylthiophene, nickel-catalyzed methodologies have emerged as one of the most vital components in the synthesis of regioregular polymers. To date, many new approaches based on Ni-catalysis have been developed and are being utilized succesfully in the synthesis of novel conjugated polymers. Ni-catalyzed C–H functionalization polymerization and Kumada catalyst-transfer polymerization (KCTP) are the most popular protocols being used for the synthesis of conjugated polymers nowadays. Some researchers have utilized Ni-catalyzed Suzuki, Negishi and Murahashi coupling reactions to polymerize thiophene-based monomers. Some of the very recent work reported by exploiting Ni-catalysis is briefly discussed in this review.

### 3.1. Ni-catalyzed C–H functionalization polymerization

Ni-catalyzed dehydrobrominative polycondensation is conducted by deprotonation at C–H bond of thiophene monomer using bulky magnesium amide, TMPMgCl·LiCl (chloromagnesium 2,2,6,6-tetramethylpiperidide lithium chloride) also known as Knochel–Hauser base.^167,168^ This method helps polymerization to take place at room temperature within shorter time period.^169–171^ Knochel–Hauser base is more effective for regioselective deprotonation of arenes as compared to the strong bases such as lithium amides (R_2_NLi) and alkyl lithium reagents (RLi), traditionally used for deprotonation. These strong bases lead to undesirable reactions due to their strong nucleophilicity (*e.g.* Chichibabin addition) and high reactivity. Low stability of lithium amides in THF solutions at room temperature is another serious limitation, which requires *in situ* generation of these reagents. Moreover, the requirement of low temperatures (−78 to −90) for the protonation of arenes further complicates the scale-up of these reactions.^170,172–174^

Employment of 2-halo-3-substituted thiophenes instead of 2,5-dihalothiophenes along with the Knochel–Hauser^167,175^ base have been the key for the successful synthesis of thiophene-based polymers.^170,176–182^ Magnesium amide does not interfere with the propagation of the polymer during the course of the reaction.^164^ Dehydrobrominative polycondensation proceeds with the higher atom efficiency compared to the dehalogenative polycondensation employing Grignard reagent. The loss of two halogen atoms from the thiophene monomer could be problematic as it results in a greater mass loss in the polymerization reaction while dehydrobrominative polycondensation results in the synthesis of highly regioregular head-to-tail polythiophenes with the improved atom efficiency.^170^ This protocol has been successfully employed by different research groups to synthesize highly regioregular thiophene-based polymers, from which some of the recent works have been highlighted here.

In 2012, Shunsuke Tamba and co-workers synthesized, poly(thienylenepyridinylene), poly(thienylenephenylene) and poly(benzodithiophene) by nickel catalyzed C–H functionalized polycondensation using Knochel–Hauser base.^178^ These polymers were obtained under mild reaction conditions in superior atomic efficiency by C–H coupling.

**Scheme 4:**
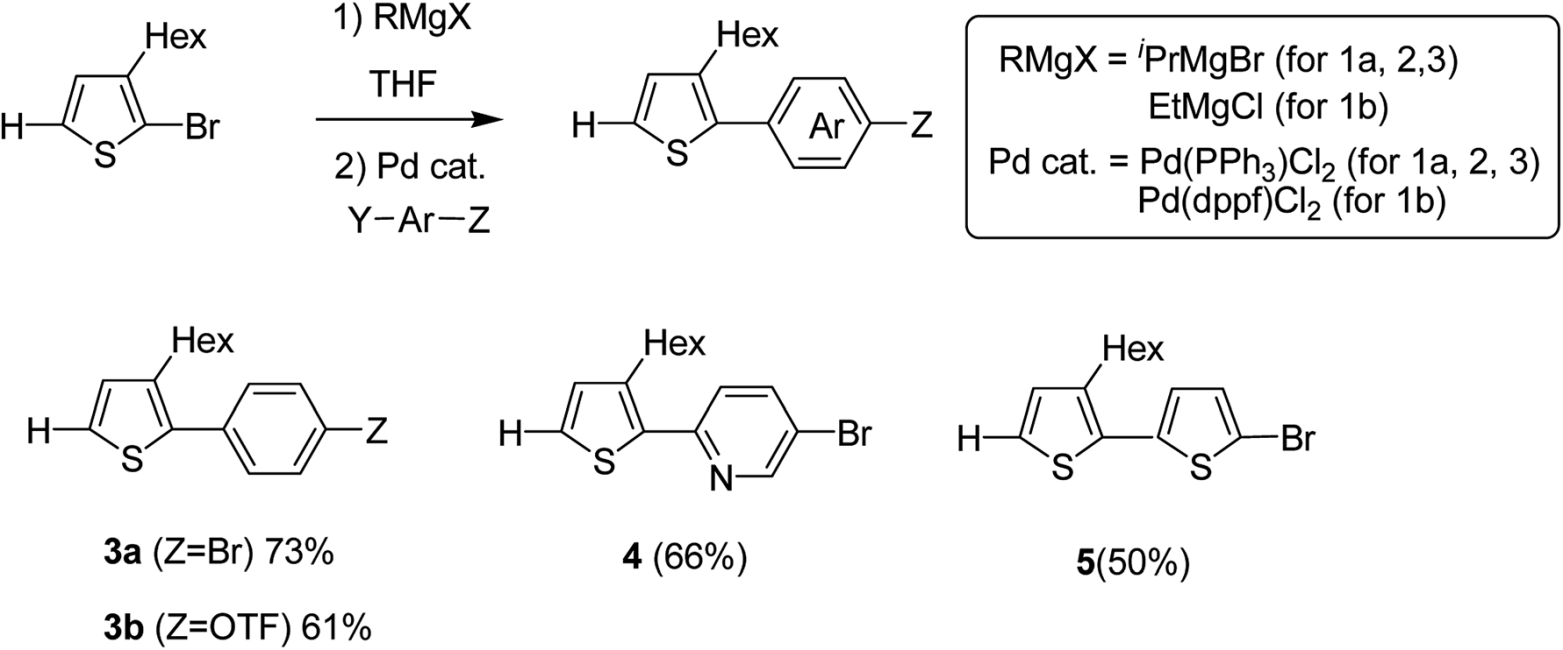
Kumada–Tamao–Corriu cross-coupling in the presence of a palladium catalyst for the synthesis of 3-hexylthiophene-based monomers 3a, 3b, 4 and 5.

Monomers 3–5 were synthesized through the reaction of the aryl halides and thienyl Grignard reagent *via* Kumada–Tamao–Corriu cross-coupling using palladium catalyst. 3-Hexyl-2-halomagnesio-thiophene was generated using the bromine–metal exchange reaction with iPrMgBr. Newly produced thienyl Grignard reagent was coupled with 1,4-dibromobenzene using 0.5 mol% Pd(PPh_3_)_2_Cl_2_ to obtain 3a in 73% yield. Similarly, products 4 and 5 were obtained in 66 and 50% yields by the reaction of 5-bromo-2-iodopyridine and 2,5-dibromothiophene, respectively. Using Pd(PPh_3_)_2_Cl_2_, coupling product 3b was obtained in only 21% yield due to the low catalytic activity of Pd-catalyst, while 5.0 mol% of Pd(dppf)Cl_2_ resulted in the 61% yield of 3b due to its high catalytic activity (Scheme 4).

**Scheme 5:**
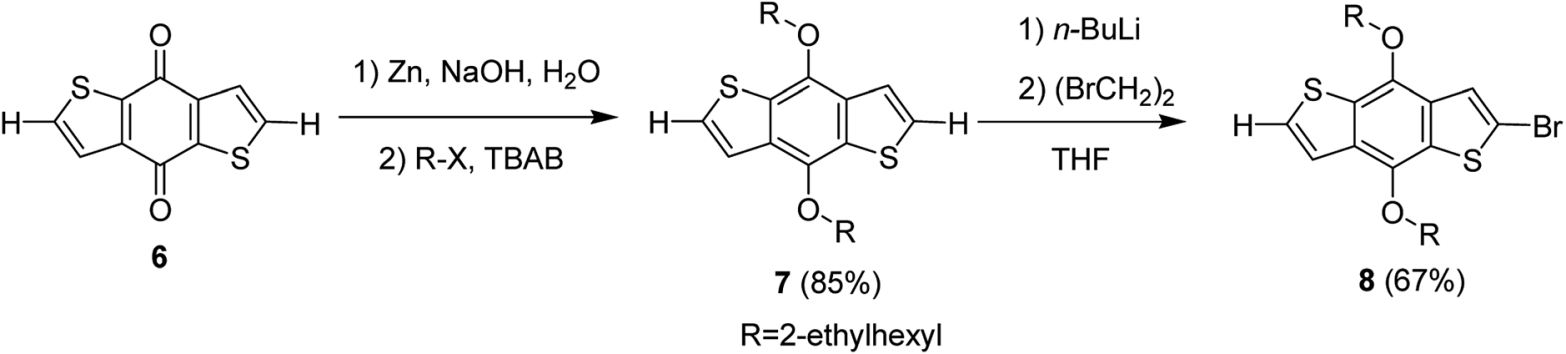
Synthesis of 2-bromo-4,8-di(2-ethylhexyloxy)benzo[1,2-*b*;3,4-*b*′]dithiophene monomer.

Hou and Yang's procedure^183^ was used to synthesize benzo[1,2-*b*:4,5-*b*′]dithiophene-4,8-dione 6 in 63% yield, using thiophene-3-carboxylic acid (Scheme 5). Aqueous solution of sodium hydroxide, containing zinc powder, was applied to reduce quinone 6, which was then treated with catalytic amount of tetrabutylammonium bromide and alkyl bromide. Reaction with 2-ethylhexylbromide was performed to obtain alkylated product 7 in 85% yield. Bromination reaction was conducted with the help of *n*-butyllithium using proton abstraction reaction, which, on subsequent treatment with 2-ethylhexylbromide, yielded brominated product 8.

**Scheme 6:**
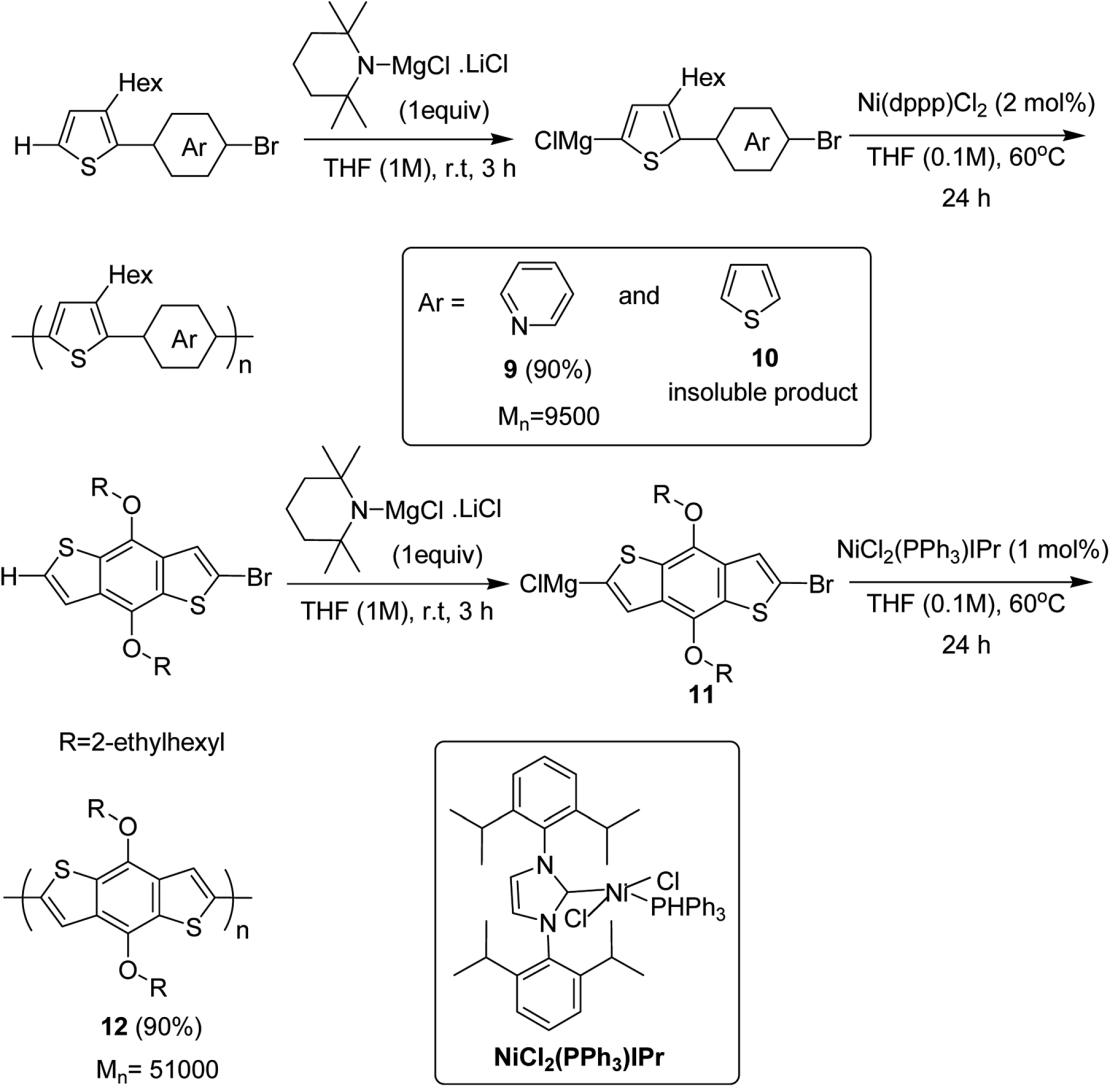
Ni-catalyzed synthesis of poly(thienylenpyridinylene) 9, poly(thienylenephenylene) 10 and poly(benzodithiophene) 12.

Ni(dppp)Cl_2_ was applied for the C–H functionalization polycondensation, where a Knochel–Hauser base was used to afford polymeric product of 2-(5-bromopyridine-2-yl)-3-hexylthiophene 4 to afford polymer 9 with 90% yield. Polymerization of 2-(5-bromothiophene-2-yl)-3-hexylthiophene 5 resulted in a highly insoluble polymer 10. The reaction of benzodithiophene 8 with TMPMgCl LiCl to produce intermediate 11 and the subsequent addition of nickel catalyst bearing an IPr ligand produced the corresponding polymer 12 with excellent yield of 90% (Scheme 6).

Shunsuke Tamba and co-workers, in 2014, polymerized 3-hexylthiophene to afford head to tail type regioregular poly(3-hexylthiophene) by using nickel(ii) catalyst for the deprotonative C–H functionalization polycondensation of 2-(phenylsulfonyl)-3-hexylthiophene.^184^ 2-(Phenylsulfonyl)-3-hexylthiophene 13, was used as a monomer precursor, which was subjected to deprotonation with Knochel–Hauser base at room temperature. Ni(dppe)Cl_2_ was added as a catalyst to the reaction mixture, which yielded polythiophene with number average molecular weight of (*M*_n_) 9300 g mol^−1^. ^1^H NMR analysis confirmed 99% head-to-tail (HT) regioregularity of the desired polymer.

**Scheme 7:**
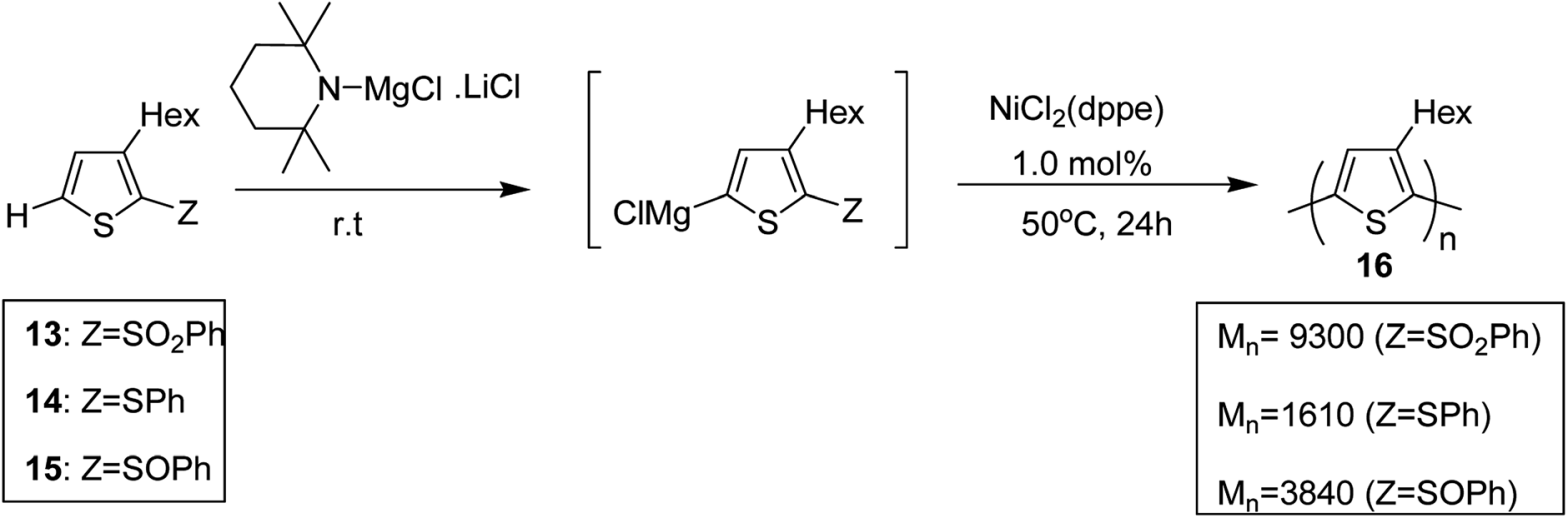
Deprotonative polymerization *via* C–S bond cleavage in the presence of Ni catalyst.

It is worth noting that carbon–carbon bond formation using transition metal catalysis occurred through C–S bond cleavage, which is a new class of cross-coupling polycondensation reactions. Polymerization of 3-hexylthiophen-2-yl phenyl sulfide monomer 14 was performed under similar conditions to obtain P3HT 16 with much lower yield and molecular weight (*M*_n_ = 1610 g mol^−1^). 2-Phenylsulfinyl-3-hexylthiophene 15, a sulfoxide monomer, was also polymerized under similar conditions to afford the corresponding polymer with 74% yield and molecular weight of 3840 g mol^−1^ (*M*_n_). These results showed that phenyl sulfone served as the most effective leaving group in the polymerization reactions of thiophene (Scheme 7).

**Scheme 8:**
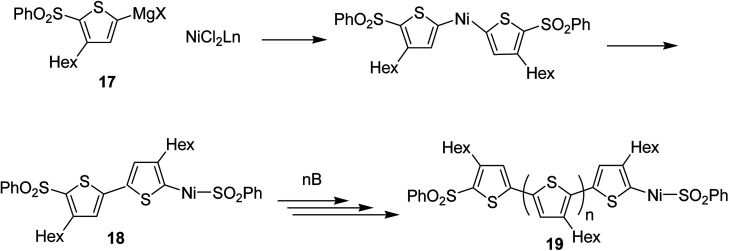
Proposed mechanism of the cross-coupling polymerization *via* C–S bond cleavage.

Concerning the polymerization mechanism of halothiophenes, the initiation reaction in polymerization is considered to be reductive tail to tail homocoupling of metalated sulfonyl thiophene and oxidative addition of Ni(0) species into the C–S bond. Homocoupling of metalated monomer 17 takes place initially to give 18, propagation reaction occurs by incorporation of the monomer 17 at the terminal C–S bond. An end group of 19 is the terminal thiophene having SO_2_Ph group (Scheme 8).

In 2013, Shunsuke Tamba and co-workers reported the use of [CpNiCl(SIPr)] catalyst for the synthesis of extremely high molecular weight head-to-tail type regioregular poly(3-alkylthiophene) *via* dehydrobrominative polycondensation.^179^ Polymerization of 2-bromo-3-hexylthiophene proceeded with a catalytic amount of [CpNiCl(SIPr)] and an equivalent amount of Knochal–Hauser base to yield regioregular poly(3-hexylthiophene) 16 up to *M*_w_ = 815 000. A self-standing film of polythiophene was obtained with *M*_w_ = 414 000, while the attempted formation of corresponding films with lower molecular weight (*M*_w_ = 38 000) was not successful. Mechanical characteristics of this self-standing film are of great interest in terms of the relationship of its physical properties with its mechanical treatment as a high performance material.

**Scheme 9:**
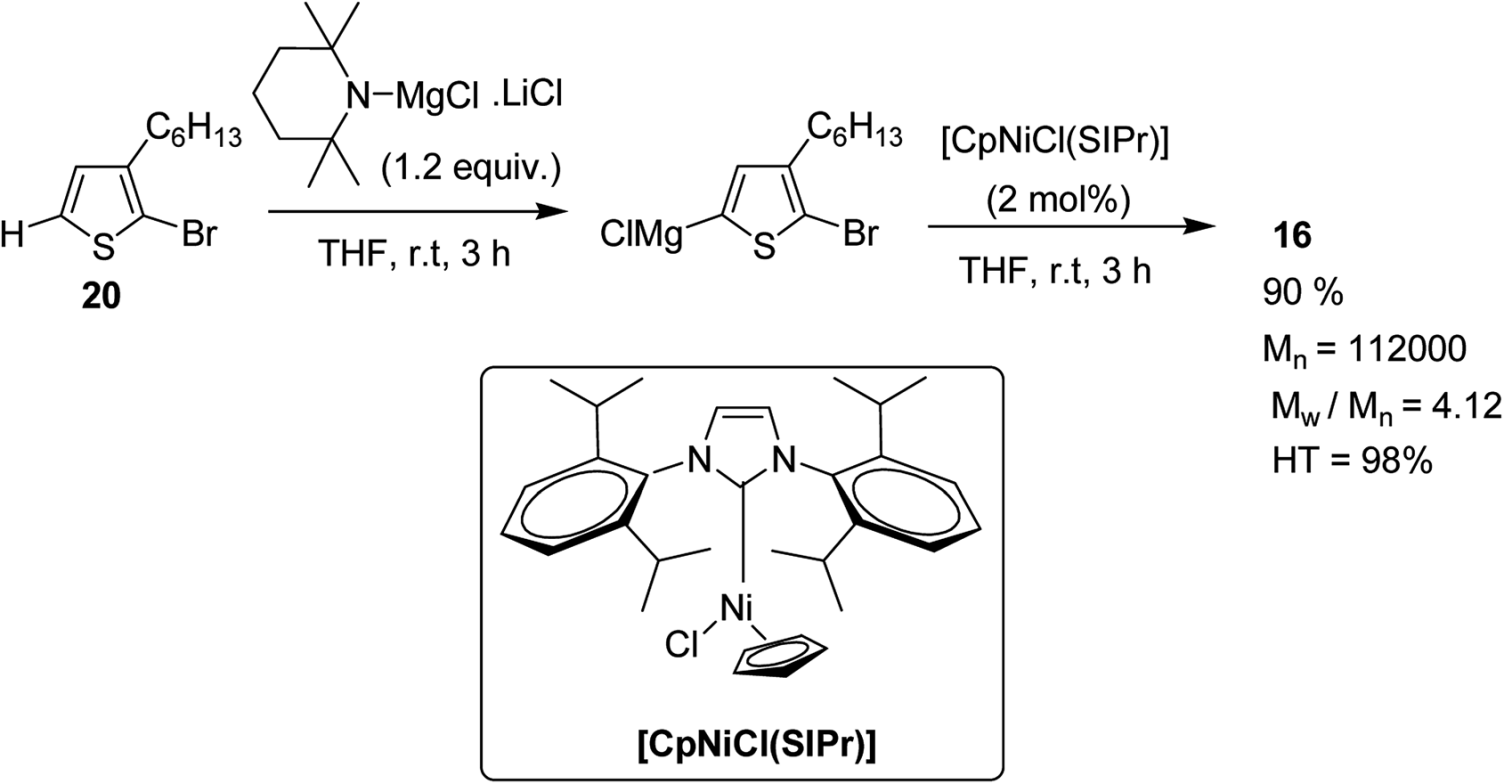
Synthesis of poly(3-hexylthiophene) 16.

Polymerization of 2-bromo-3-hexylthiophene 20 was conducted using 2 mol% of nickel catalyst and 1.2 equivalents of TMPMgCl·LiCl at 25 °C for 24 hours yielding the corresponding polymer 16 in 90% yield with head-to-tail regioregularity of 98%. The obtained high molecular weight polymer had reasonable solubility in 1,2-dicholorobenzene and chloroform. [CpNiCl(SIPr)]-catalyzed polymerization was performed under several different conditions and the results are enlisted in Table 1, which showed that optimum conditions for obtaining high molecular weight poly-3-hexylthiophene are room temperature and 0.5–2 mol% catalyst loading (Scheme 9).

**Table 1:** Polymerization of 2-bromo-3-hexylthiophene 20 with [CpNiCl(SIPr)]^a^

Entry	Ni-Catalyst (mol%)	Temperature (°C)	Yield^b^ (%)	*M*_n_ c	*M*_w_ c	*M*_w_ c/*M*_n_
1	3	r.t	27	103 000	214 000	2.08
2	2	r.t	81	224 000	414 000	1.85
3	1	r.t	65	174 000	345 000	1.98
4^d^	0.75	r.t	74	233 500	815 000	3.49
5^d^	0.5	r.t	85	172 600	630 000	3.65
6	2	40	54	10 700	21 600	2.02
7^e^	2	0	33	126 000	295 000	2.34

a Reaction was performed with 1.0 mmol of monomer 20 and 1.0 equiv. of TMPMgCl·LiCl in THF for 3 hours (metalation) and 24 hours (polymerization).

b Isolated yield.

c *M*_n_, *M*_w_ and *M*_w_/*M*_n_ values were estimated SEC analysis using chloroform as an eluent.

d Reaction was carried out with 20 mmol of 18 and 60 mL THF.

e Polymerization time 72 hours.

The optimized polymerization conditions of 2-bromo-3-hexylthiophene were also applied to the synthesis of other 3-substituted bromothiophenes in the presence of [CpNiCl(SIPr)] catalyst. Polymerization of these monomers proceeded efficiently to afford high molecular weight polythiophenes (Table 2).

**Table 2:** [CpNiCl(SIPr)]-catalyzed polymerization of 2-bromo-3-substituted thiophenes^a^

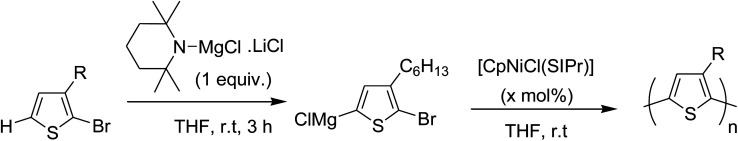							
–R	Time (h)	*x*	Yield (%)	*M*_n_ b	*M*_w_ b	*M*_w/_ *M*_n_ b	HT^c^ (%)
–C_8_H_17_	96	1.5	47	87 000	288 000	3.31	98
–C_12_H_25_	48	2.0	85	70 400	276 000	3.92	99

a Reaction was conducted with monomer (0.5 mmol) and TMPMgCl·LiCl (0.5 mmol) in 5 mL of THF at room temperature for 3 hours (metalation) and at room temperature for 24 hours (polymerization).

b Estimated by SEC analysis using chloroform as an eluent.

c Estimated by ^1^H NMR.

**Scheme 10:**
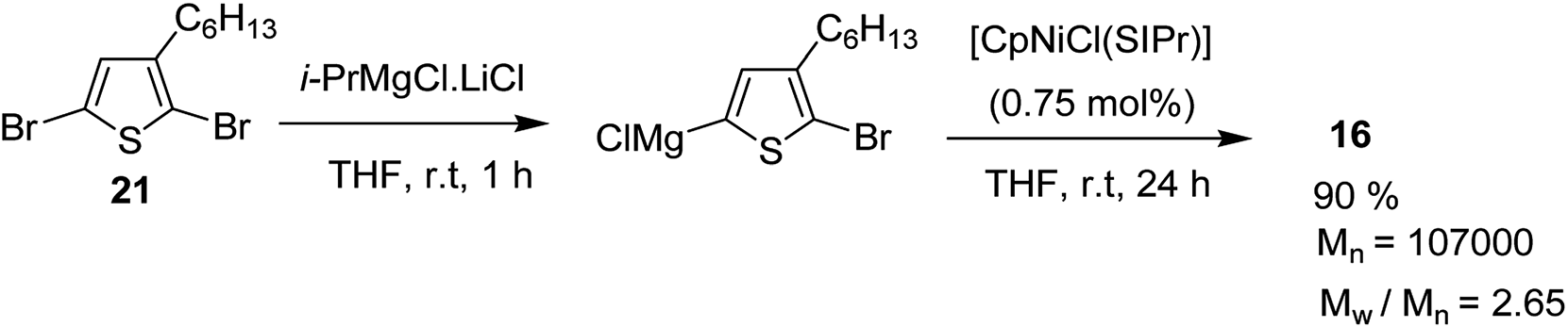
[CpNiCl(SIPr)] catalyzed GRIM polymerization of 2,5-dibromo-3-hexylthiophene 21.

[CpNiCl(SIPr)] catalyst was also found to be effective in GRIM polymerization of 2,5-dibromo-3-hexylthiophene. Treatment of 21 with ^i^PrMgCl·LiCl in THF at room temperature and subsequent addition of Ni-catalyst initiated the polymerization reaction. Further stirring at room temperature yielded poly(3-hexylthiophene) 16 with *M*_n_ = 107 000 (Scheme 10).

**Scheme 11:**
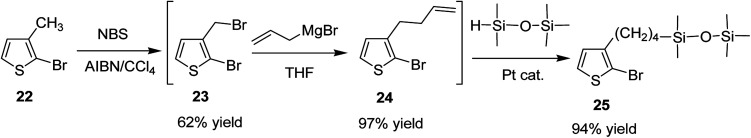
Synthesis of thiophene monomer (precursor) 25 bearing siloxane.

Keisuke Fujita and co-workers synthesized polythiophenes substituted with siloxane moiety at 3-position in 2016.^185^ Synthesis of siloxane bearing monomer started with radical bromination of 2-bromo-3-methylthiophene 22 with NBS in the presence of azoisobutyronitrile (AIBN) to afford bromomethylated intermediate 23, which was subjected to allylation by treatment with allyl magnesium bromide to give ω-olefinic product 24. Silylated product 25 was obtained in a quantitative yield by hydrosilylation of 24 using platinum catalyst (Scheme 11).

**Scheme 12:**
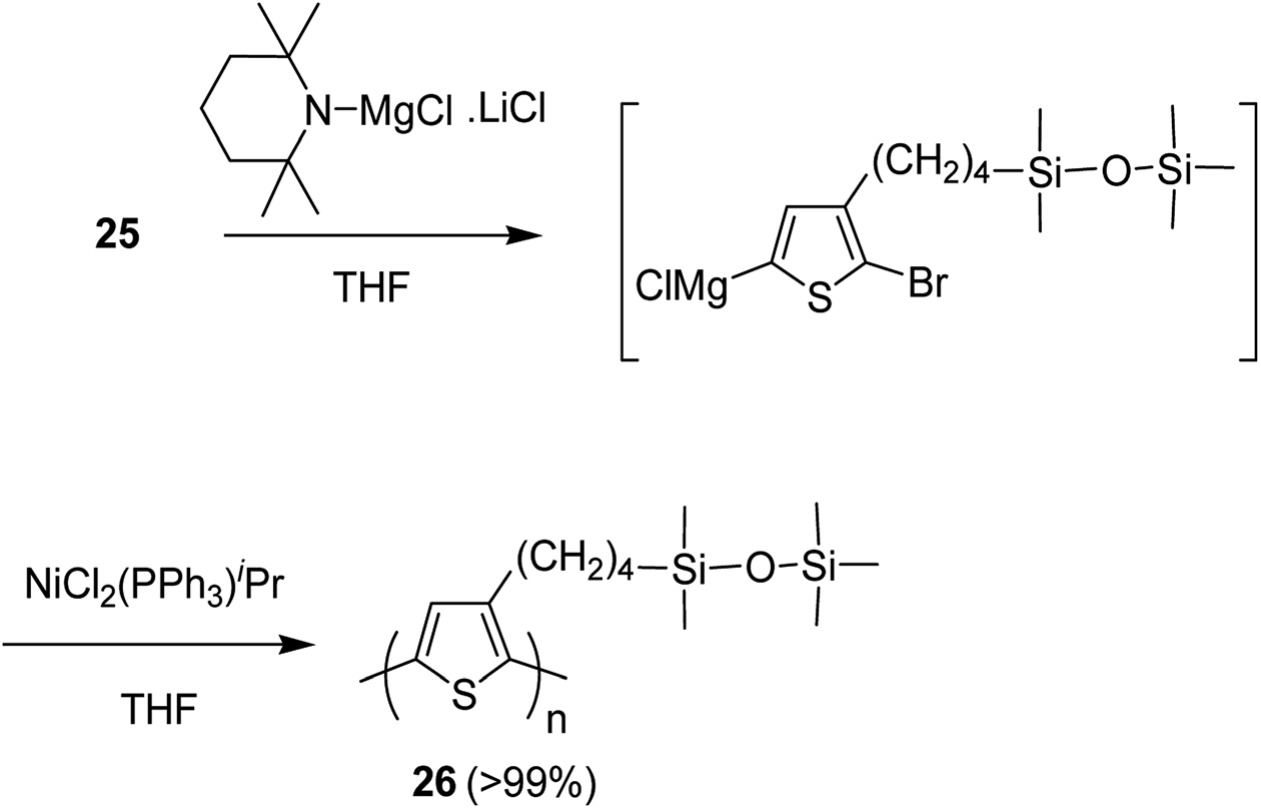
Deprotonative polymerization of 25 with nickel(ii) catalyst.

Nickel-catalyzed deprotonative polycondensation of 2-bromo-3-substituted thiophene 23 was performed using TMPMgCl·LiCl. Deprotonation step was conducted at 60 °C for 1 hour to yield the corresponding thiophene magnesium species, which was then treated with 0.1–5 mol% Ni(PPh_3_)Cl_2_ to afford the desired regioregular head to tail polythiophene 24. The obtained polythiophene bearing siloxane as a substituent was found to be dissolved in a variety of organic solvents especially in hexanes, allowing formation of thin films (Scheme 12).

Chia-Hua Tsai and co-workers in 2016 demonstrated the synthesis of periodic π-conjugated polymers of group 16 heterocycles (thiophene, furan and selenophene) with relatively low dispersities and controlled chain length using catalyst-transfer polycondensation.^186^ In order to ensure the well-defined sequence, the copolymers were synthesized by linking short oligomers through catalyst-transfer polycondensation (CTP). The redox potentials and optical band gaps were reported to vary with the composition of the copolymers in a predictable manner. Moreover, the periodic sequences exhibited well-defined morphologies, and the packing patterns mimic those of regioregular P3HT.

**Scheme 13:**
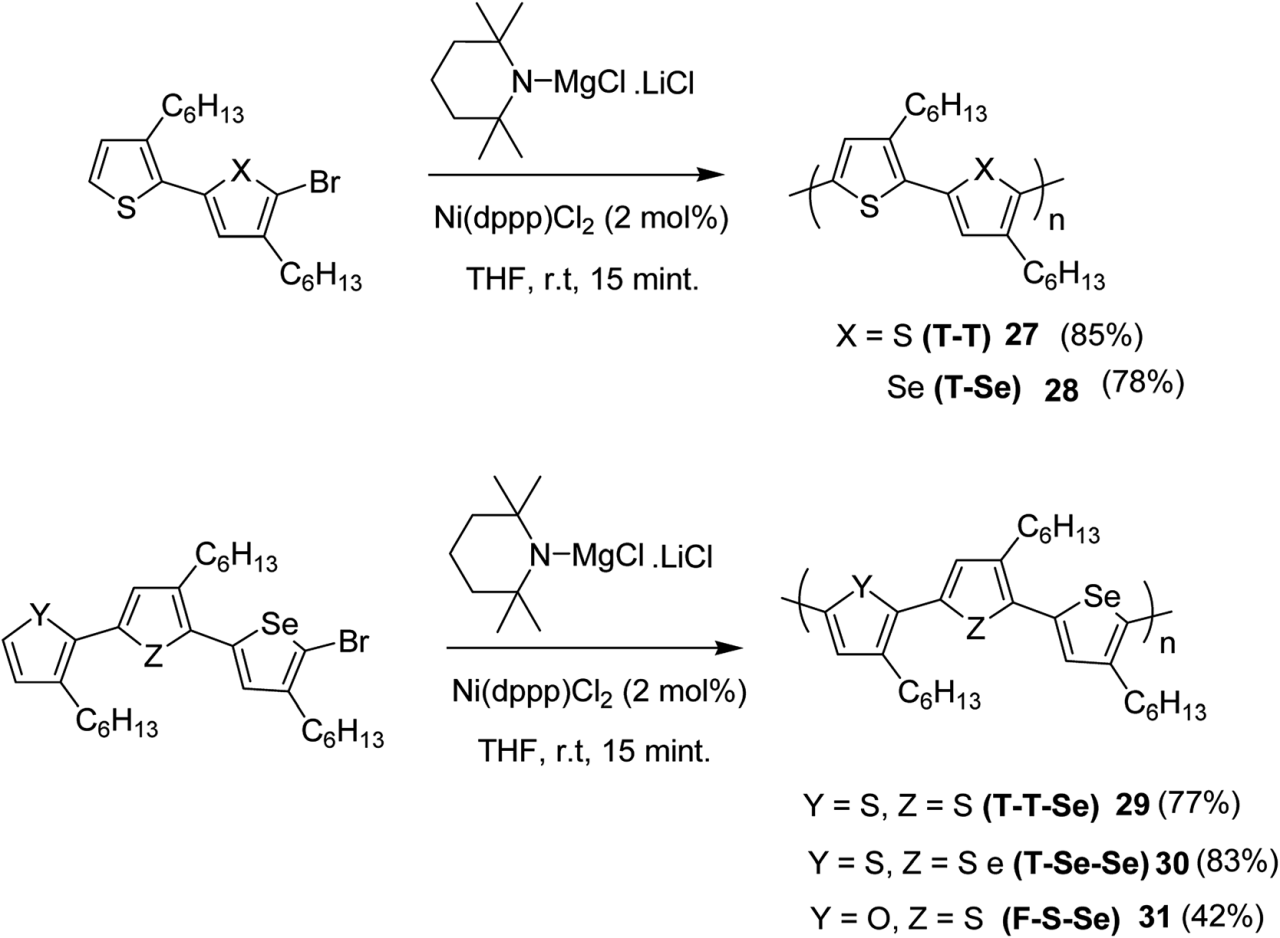
Synthesis of periodic copolymers 27–31.

All the monomers were synthesized by the cross-coupling reactions of individual heterocyclic ring using Pd_2_dba_3_ in 1,4-dioxane at 100 °C, and then NBS was used to introduce bromine units. TMPMgCl·LiCl selectively deprotonated the monomers at position 5, and Ni(dppp)Cl_2_ catalyst was added to initiate the polymerization. Reaction mixtures were quenched after 15 minutes and the polymers 27–31 were precipitated using 6 M HCl/MeOH solution and washed with methanol (Scheme 13).

### 3.2. Ni-catalyzed Kumada catalyst-transfer polymerization (KCTP)

**Scheme 14:**
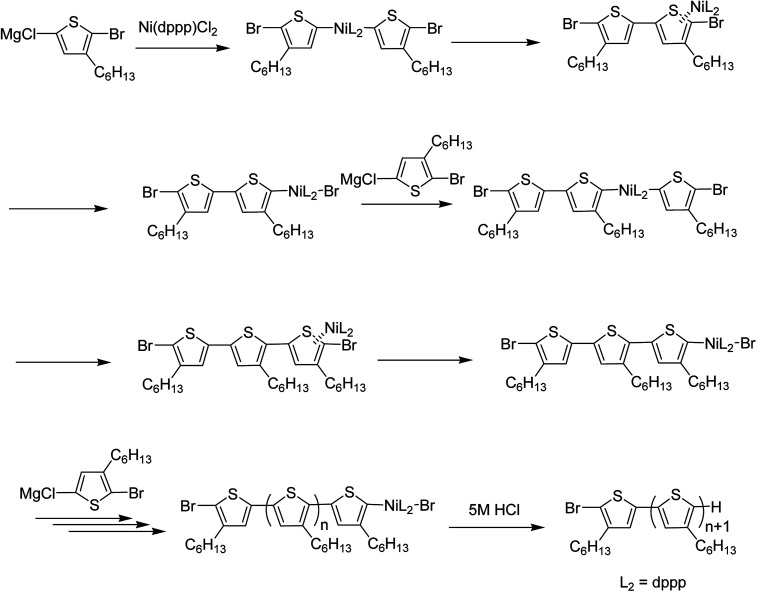
Catalyst-transfer condensation polymerization mechanism.

Nickel-catalyzed Kumada catalyst-transfer polymerization (KCTP), also referred to as Grignard metathesis polymerization (GRIM), is an extremely fast developing protocol for the preparation of well-defined conjugated polymers. Since the discovery of Yokozawa^25,187^ and McCullough^49^ in 2004, polymerization of poly(3-hexylthiophene) has been considered to proceed *via* chain-growth mechanism (Scheme 14) instead of step-growth mechanism, and various conjugated polymers including thiophene-,^22^ phenylene-,^188^ pyrrole-^189^ and fluorine-based^190,191^ conjugated homopolymers, all-conjugated block copolymers,^192–196^ low bandgap homopolymers^197,198^ and polymer brushes,^199–203^ have been synthesized *via* KCTP/GRIM.

The term “Kumada catalyst-transfer polycondensation” was created by Yokozawa and co-workers, which reflects an essential step (intramolecular catalyst transfer process) during the catalytic cycle of polymerization, while the term Grignard metathesis (GRIM) refers to the stage of monomer synthesis rather than to the chain-growth process itself.^187^ Chain growth mechanism offers more control over molecular weight distribution and end group functionalization compared to step-growth polymerization mechanisms (mostly Suzuki and Stille couplings) and is more effective in achieving reproducible material properties and subsequently, reproducible device parameters.^187,204^ Low degree of control over growing polymer chain often leads to batch-to batch differences and altered material properties which is undesirable to obtain reproducible results. Discovery of chain growth mechanism has extended the scope of KCTP and is widely used to synthesize novel thiophene-based polymers with tailored architecture. Some of the very recent work in this regard is reviewed below.

Colin R. Bridges and co-workers in 2014 reported the first example of the synthesis of electron-rich/electron-deficient, all conjugated diblock copolymers using Ni(ii) diimine catalyst, [*N*,*N*′-dimesityl-2,3-(1,8-naphthyl)-1,4-diazabutadiene]dibromonickel (MesAn), with an electron donating ligand.^205^ This catalyst formed strong association with both electron-rich and electron-deficient monomers due to which it could be very effective in performing their controlled polymerization.

**Scheme 15:**
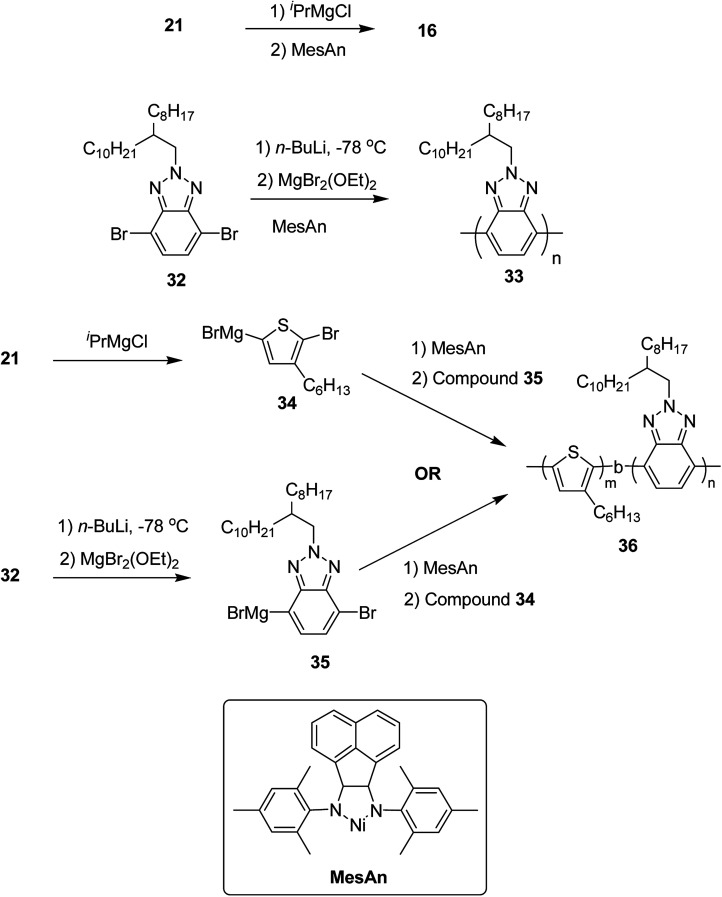
Synthesis of homopolymers P3HT 16 and PBTz 33 and P3HT-*b*-PBTz block copolymer 36.

Poly(3-hexylthiophene) (P3HT) 16 and polybenzotriazole (PBTz) 33 were chosen as the electron-rich and electron deficient-blocks, respectively. MesAn catalyst had been used previously for the synthesis of polyolefins but was never tested for the synthesis of conjugated diblock copolymers *via* Kumada catalyst transfer polymerization (KCTP). MesAn association complex with P3HT and PBTz monomers showed stabilization of 148.3 and 143.8 kJ mol^−1^, respectively. These complexes exhibited greater stability than other Ni(ii)-diimine catalyst-monomer systems,^206^ suggesting a good control of MesAn over both benzotriazole and thiophene polycondensation. Chain transfer or chain termination reactions are prevented by strong catalyst affinity to the monomer, thus, allowing more control over polymerization. Controlled polymerization is evident by the narrow dispersities of the polymers with the molecular weights that could be controlled by the catalyst to monomer ratios. To test this, homopolymers 16 and 33 were synthesized from their respective monomers 2,5-dibromo-3-hexylthiophene 21 and 4,7-dibromo-2-(2-octyldodecyl)-2*H*-benzo[*d*][1,2,3]triazole 32. At 2 mol% loading of catalyst, 16 was produced with *M*_n_ = 13.3 kDa, *Đ* = 1.25 and 33 with *M*_n_ = 10.00 kDa, *Đ* = 1.27. These molecular weight and dispersity values are similar to those obtained for the polymerization of other conjugated polymers produced by using this catalyst : monomer ratio. Decrease in the ratio to 1 mol% resulted in the expected doubling of molecular weight, *M*_n_ = 19.0, *Đ* = 1.31 for 16 and *M*_n_ = 19.1, *Đ* = 1.49 for 33, indicating a good control of MesAn over the polymerization of both electron-rich and electron-deficient monomers. Block copolymer 36 was synthesized by sequential addition of thiophene and benzotriazole monomers *via* intermediates 34 and 35. The composition of block copolymer, P3HT:PBTz closely resembled to the feed ratio (Table 3). These newly synthesized donor-*block*-acceptor copolymers exhibited interesting electrochemical and phase separation properties (Scheme 15).

**Table 3:** Molecular weight and dispersity for P3HT 16, PBTz 33 and P3HT-*b*-PBTz 36 block copolymers

Feed composition (P3HT:PBTz)	Polymer composition (P3HT:PBTz)^a^	Catalyst loading (mol%)	*M*_n_ b (kDa)	*Đ* b
0 : 100	0 : 100	2	10.0	1.25
0 : 100	0 : 100	1	19.1	1.49
100 : 0	100 : 0	2	13.3	1.27
100 : 0	100 : 0	1	19.0	1.31
25 : 75	20 : 80	1	18.6	1.42
50 : 50	50 : 50	1	20.6	1.74
75 : 25	79 : 21^c^	1	21.2	1.39

a Determined with the help of ^1^H NMR.

b Dispersity and molecular weight determined by size exclusion chromatography (SEC) in 1,2,4-trichlorobenzene at 140 °C.

c 33 used as a macroinitiator.

In 2015, Zhuping Fei and co-workers reported two strategies for the synthesis of regioregular 3-alkyl-4-fluorothiophenes, F-P3HT 37, F-P3OT 38 and F-P3EHT 39, containing straight (hexyl and octyl) and branched (2-ethylhexyl)alkyl groups, respectively.^207^ Comparison of the properties of the fluorinated polymers with their non-fluorinated analogues revealed that backbone fluorination results in an increase in the polymer ionization potential without causing a significant change in the optical band gap, indicating that fluorination leads to lowering of both the HOMO and LUMO energy levels. Average charge carrier mobilities for the fluorinated polymers are found to be increased up to a factor of 5 in the field-effect transistors. Fluorination also enhances the tendency to aggregate in the solution.

**Scheme 16:**
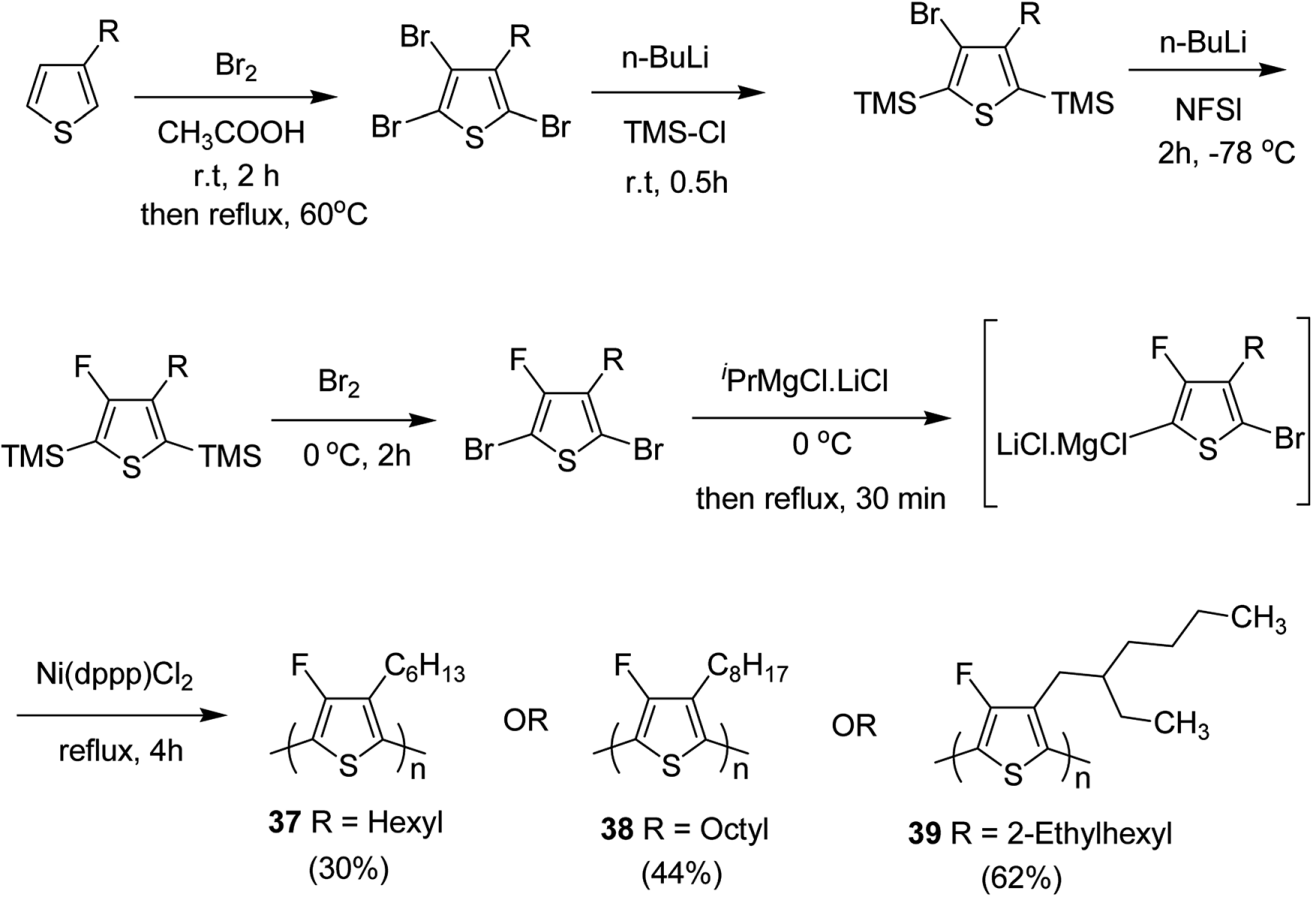
Synthesis of monomers and polymers F-P3HT 37, F-P3OT 38 and F-P3EHT 39 through GRIM route.

For the synthesis of fluorinated polymers 37, 38 and 39, Grignard metathesis (GRIM) route was used due to its well-known robustness and good control over the synthesis of P3HT. Thiophenes were fluorinated in the 3 and 4 positions by electrophilic fluorination of lithiated thiophenes, although the fluorination of electron rich aromatic thiophenes at these positions is non-trivial. 2 and 5 positions of the thiophenes were protected *via* TMSCl in order to prevent the rearrangement of 3 or 4 lithiated thiophenes to thermodynamically more stable 2 and 5 positions (Scheme 16).

**Scheme 17:**
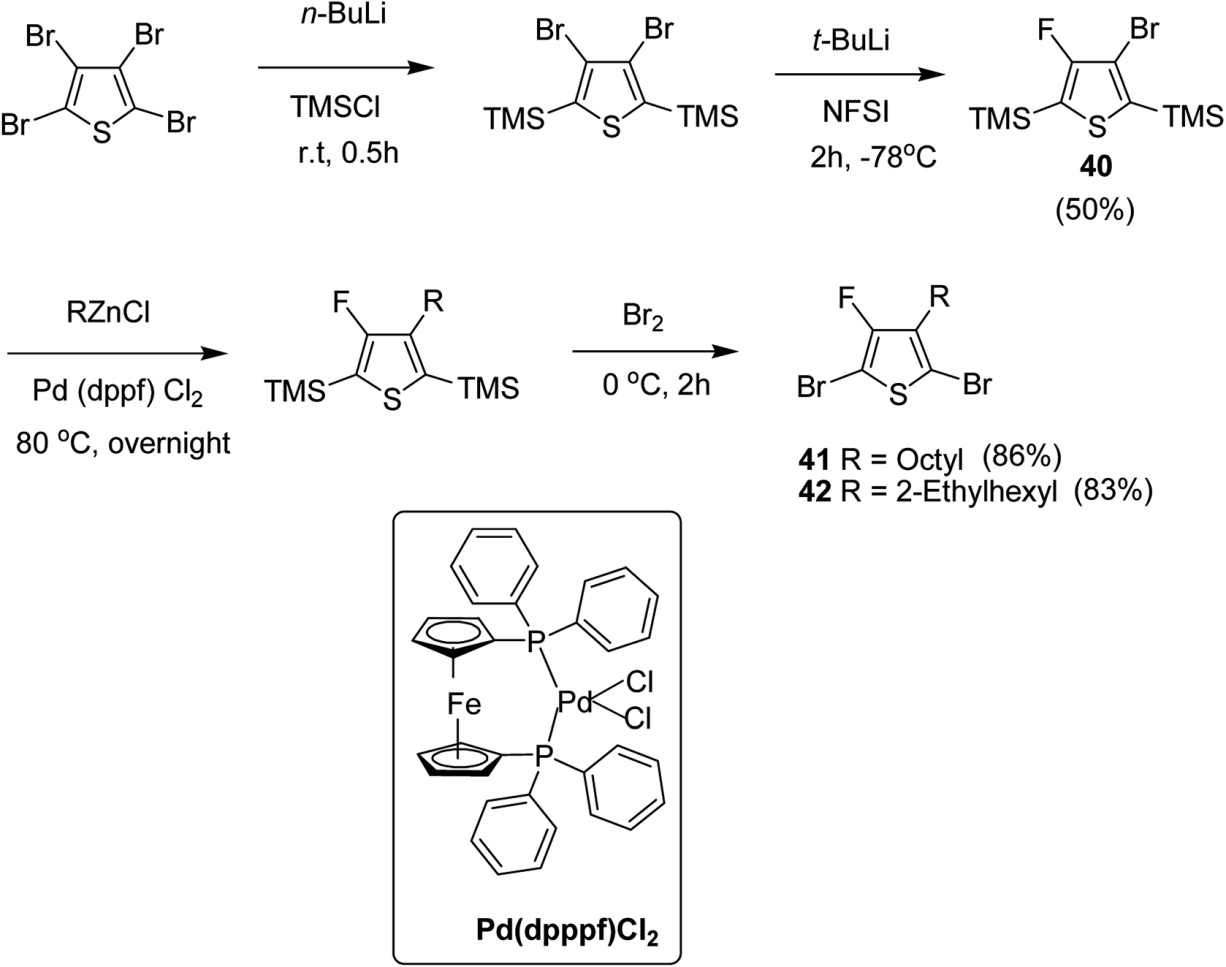
Improved synthetic route to fluorinated intermediates 41 and 42.

Early-stage introduction of the alkyl side chain proved to be problematic, which means that all the four steps needed to be repeated in order to change the alkyl side chain. Keeping in view the tedious nature of separation of fluorinated monomer from non-fluorinated byproduct, an alternate synthetic route was designed in which the side chain was introduced after the fluorination step. Fluorinated intermediate 40 was prepared from commercially available 2,3,4,5-tetrabromothiophene in two steps and a reverse-phase chromatography could be used to separate fluorinated product from the non-fluorinated byproduct. Surprisingly, 40 was found to be unreactive to the standard Kumada coupling conditions used for 3-bromothiophene, thus, Negishi cross-coupling with octyl or 2-ethylhexyl zinc bromide was used in order to incorporate alkyl side chains to the synthesis of monomers 41 and 42, employing Pd(dppf)Cl_2_ as a catalyst (Scheme 17).

Silvia Destri and co-workers in 2015 reported the synthesis of a novel poly(3-alkyloxythiophene), bearing a chiral centre with particular reference to the evolution of the optical activity in passing from true solution to the solid state (ordered powders and film).^208^ Analysis of this compound supplied a strong indication to be possibly used as an inverse chiral probe as it completely lost chiroptical signal after the crystallization.

**Scheme 18:**
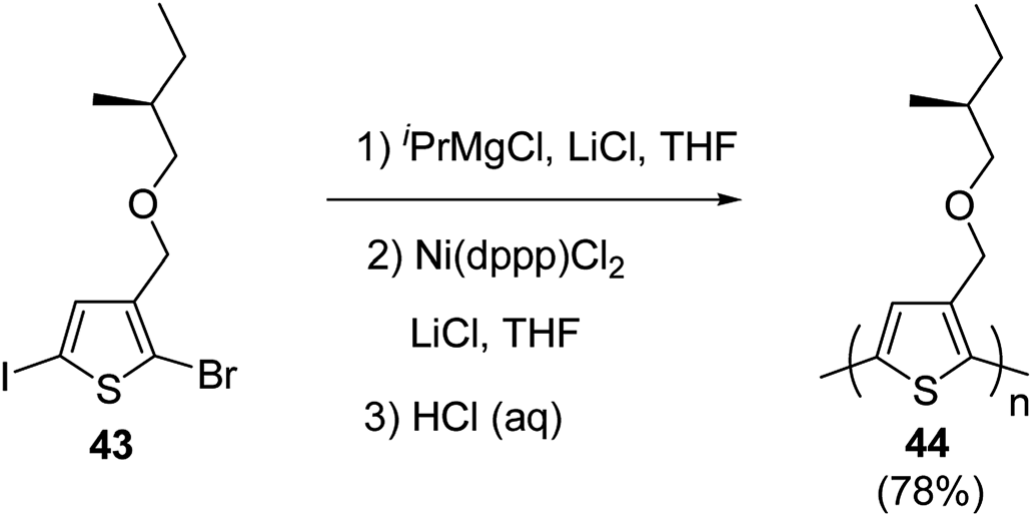
Synthetic route to regioregular poly{3-[(*S*)-(2-methylbutyloxy)methyl]thiophene} 42.

Kumada catalyst-transfer polycondensation was used to synthesize regioregular poly{3-[(*S*)-(2-methylbutyloxy)methyl]thiophene} 46, starting from 2-bromo-5-iodo-3-[(*S*)-(2-methylbutyloxy)methylbutyl]thiophene 45, in which one equivalent of isopropyl-magnesium chloride was added prior to the addition of catalyst Ni(dppp)Cl_2_. A low molecular weight polymer (*M*_n_ = 3.4 kg mol^−1^, nearly 20 repeating units) was obtained after 24 hours of reaction at room temperature with greater than 90% regioregularity. In order to increase the molecular weight of the polymer, lithium chloride was used along with the Ni(dppp)Cl_2_ catalyst, following the procedure reported by Ueda and co-workers for the monomers containing oxyethylene side chains. Use of lithium chloride resulted in number average molecular weight of 10.6 kg mol^−1^ and polydispersity index of 1.39 (Scheme 18).

**Scheme 19:**
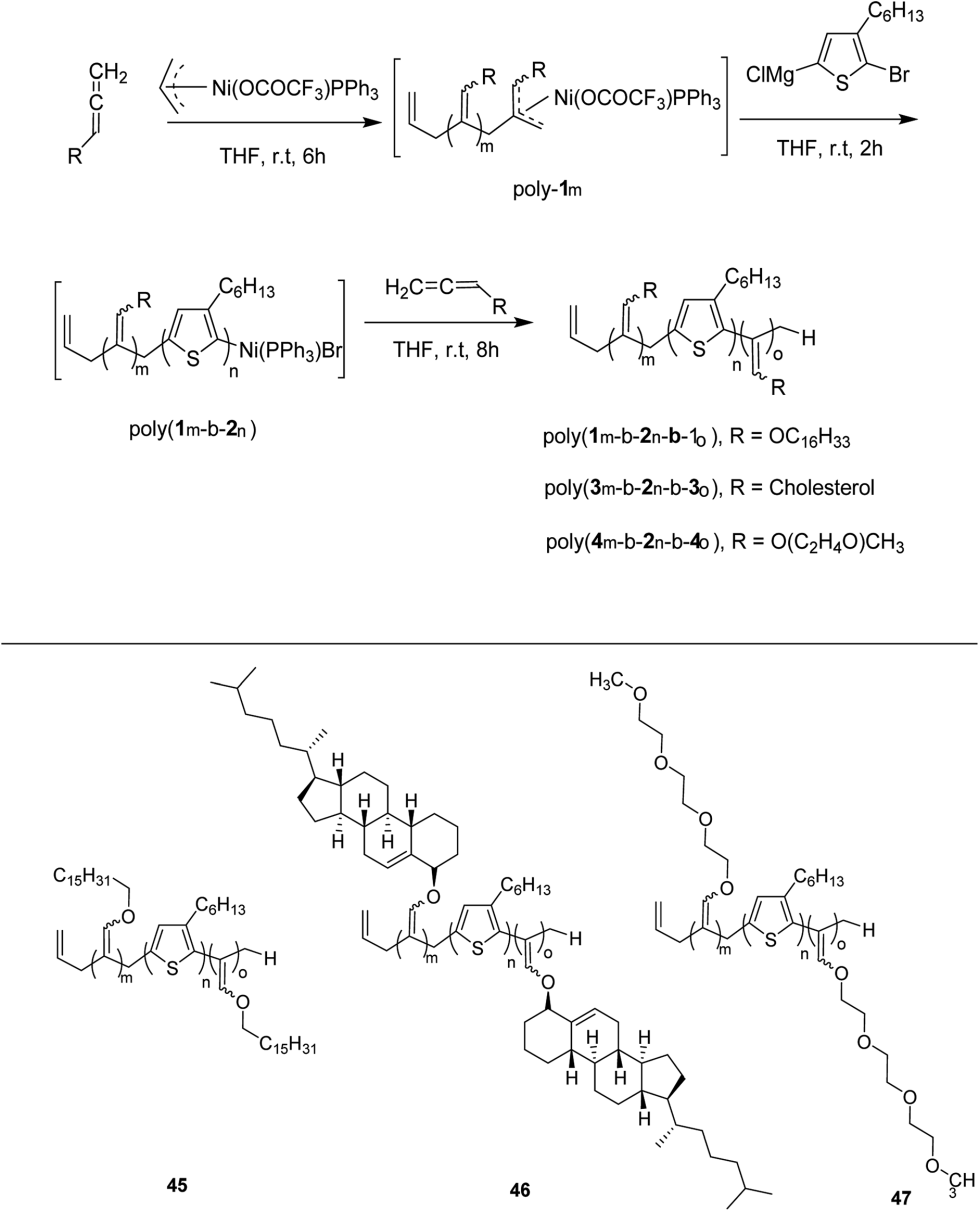
One-pot synthesis of P3HT triblock copolymers 45–47.

Zhi-Peng Yu and co-workers reported facile synthesis of a family of coil-rod-coil triblock copolymers 45–47 in 2016, consisting of poly(3-hexylthiophene) and poly(hexadecyloxylallene) using π-allylnickel(ii) complex as a catalyst.^209^ This one-pot block copolymerization was accomplished through three sequential living polymerizations, affording well-defined PHA-*b*-P3HT-*b*-PHA copolymer 45, with controlled molecular weight, narrow dispersity index and tunable composition. This isolated copolymer was found to be self-assembled into supramolecular helical structure with equivalent of left- and right-handedness. The helicity of these assemblies could be easily tuned by introducing chiral cholesteryl pendants on the polyallene segments. Moreover, same synthetic strategy was applied to synthesize water soluble amphiphilic triblock copolymer PTA-*b*-P3HT-*b*-PTA 46, containing hydrophobic P3HT and hydrophilic poly(triethyleneglycolallene) (PTA), which was found to exhibit multiresponsiveness including pH, temperature and solvent (Scheme 19). Results for one-pot synthesis of triblock copolymers through sequential living block copolymerization are summarized in the Table 4.

**Table 4:** Results for one-pot synthesis of triblock copolymers through sequential living block copolymerization using the Ni(ii) complex as a single catalyst

Polymers	Homopolymers^a^		Diblock copolymers^a^		Triblock copolymers		Yield^c^ (%)	Block ratio^d^ (m : n : o)
	*M*_n_ b	*M*_W_/*M*_n_^b^	*M*_n_ b	*M*_W_/*M*_n_^b^	*M*_n_ b	*M*_W_/*M*_n_^b^		
Poly(1m-*b*-2n-*b*-1_o_)	5.6	1.18	12.4	1.22	16.2	1.25	83	20 : 40 : 20
Poly(1m-*b*-2n-*b*-1_o_)	11.5	1.22	24.8	1.13	33.1	1.22	78	40 : 80 : 40
Poly(1m-*b*-2n-*b*-1_o_)	5.8	1.15	9.5	1.28	44.1	1.19	86	20 : 20 : 120
Poly(3m-*b*-2n-*b*-3_o_)	4.2	1.14	7.8	1.19	12.2	1.22	84	10 : 20 : 10
Poly(4m-*b*-2n-*b*-4_o_)	3.3	1.18	6.7	1.21	10.7	1.25	68	15 : 20 : 20
Poly(4m-*b*-2n-*b*-4_o_)	4.1	1.19	6.4	1.33	9.8	1.22	65	20 : 15 : 20
Poly(4m-*b*-2n-*b*-4_o_)	4.8	1.19	8.1	1.28	28.1	1.29	61	20 : 20 : 100

a The *M*_n_ and *M*_w_/*M*_n_ of the polymers were determined by analysis *via* SEC of aliquots removed from the respective reaction mixtures prior to the addition of a new monomer.

b *M*_n_ and *M*_w_/*M*_n_ were measured by SEC and are reported as their polystyrene equivalents.

c Isolated yield over the three steps.

d Block ratios deduced from the ^1^H NMR analysis.

Chunhui Zhao and co-workers in 2017 utilized the cyclopolymerization technique for the synthesis of polythiophenes for the first time.^210^ Cyclopolymerization is useful for the monomers containing two polymerizable moieties and results in the formation of insoluble cross-linked polymer networks. In cyclomerization, alternating intramolecular–intermolecular chain propagation produces a series of cyclic molecules along the polymer chain and, to achieve this, the monomers are generally designed such that thermodynamically favored five or six-membered rings are formed. However, with an elaborate monomer design, large macro cycles can be produced as well. Monomer for cyclopolymerization, indicated as GM*m* (GM = Gemini monomer and *m* = strap length), was synthesized in seven steps, in which 2-bromo-5-iodo-thiophenes were tethered by alkylene straps and they were able to be processed by catalyst-transfer polymerization (CTP).

**Scheme 20:**
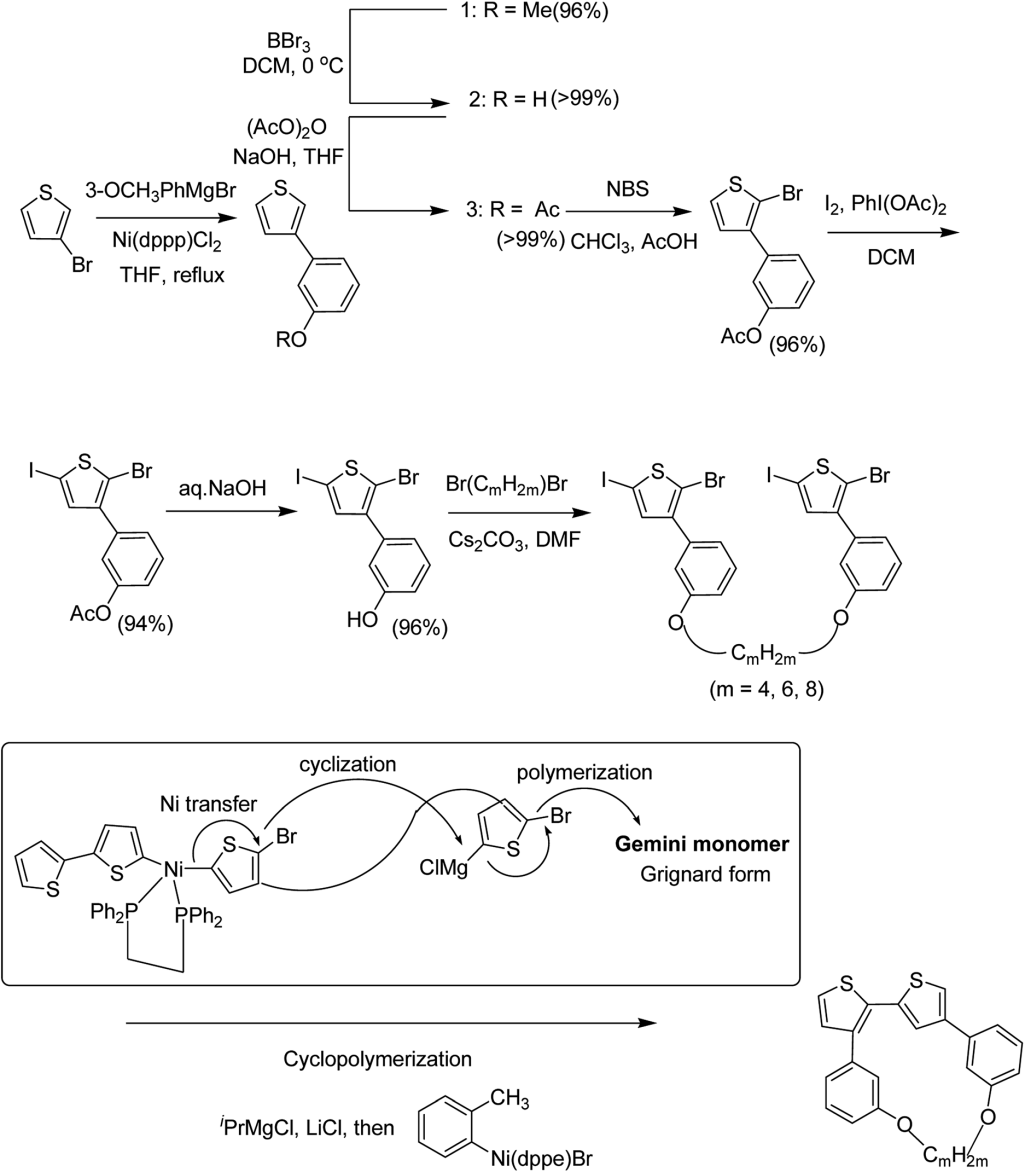
Proposed cyclopolymerization mechanism based on catalyst-transfer polycondensation.

Catalyst transfer polycondensation of GM*m*s under common conditions (GM*m* = 100 mM, Ni(dppp)Cl_2_, THF, room temperature) produced orange colored insoluble precipitates, suggesting that Gemini monomers underwent cross-linking. In order to obtain soluble polymer, catalyst system and concentrations were optimized. Diluted concentration conditions (GM*m* = 6 mM) and use of an external catalyst ((*o*-tolyl)(1,2-bis(diphenylphosphino)ethane)nickel bromide), resulted in a polymeric product soluble in common organic solvents (Scheme 20).

Zhi-Peng Yu and co-workers in 2017 reported one pot synthesis of triblock copolymers consisting of poly(3-hexylthiophene) (P3HT), poly(phenylisocyanide) (PPI) and poly(hexadecyloxylallene) (PHA) blocks, through three sequential living polymerizations of the corresponding monomers using Ni(ii) complex as a single catalyst.^211^

**Scheme 21:**
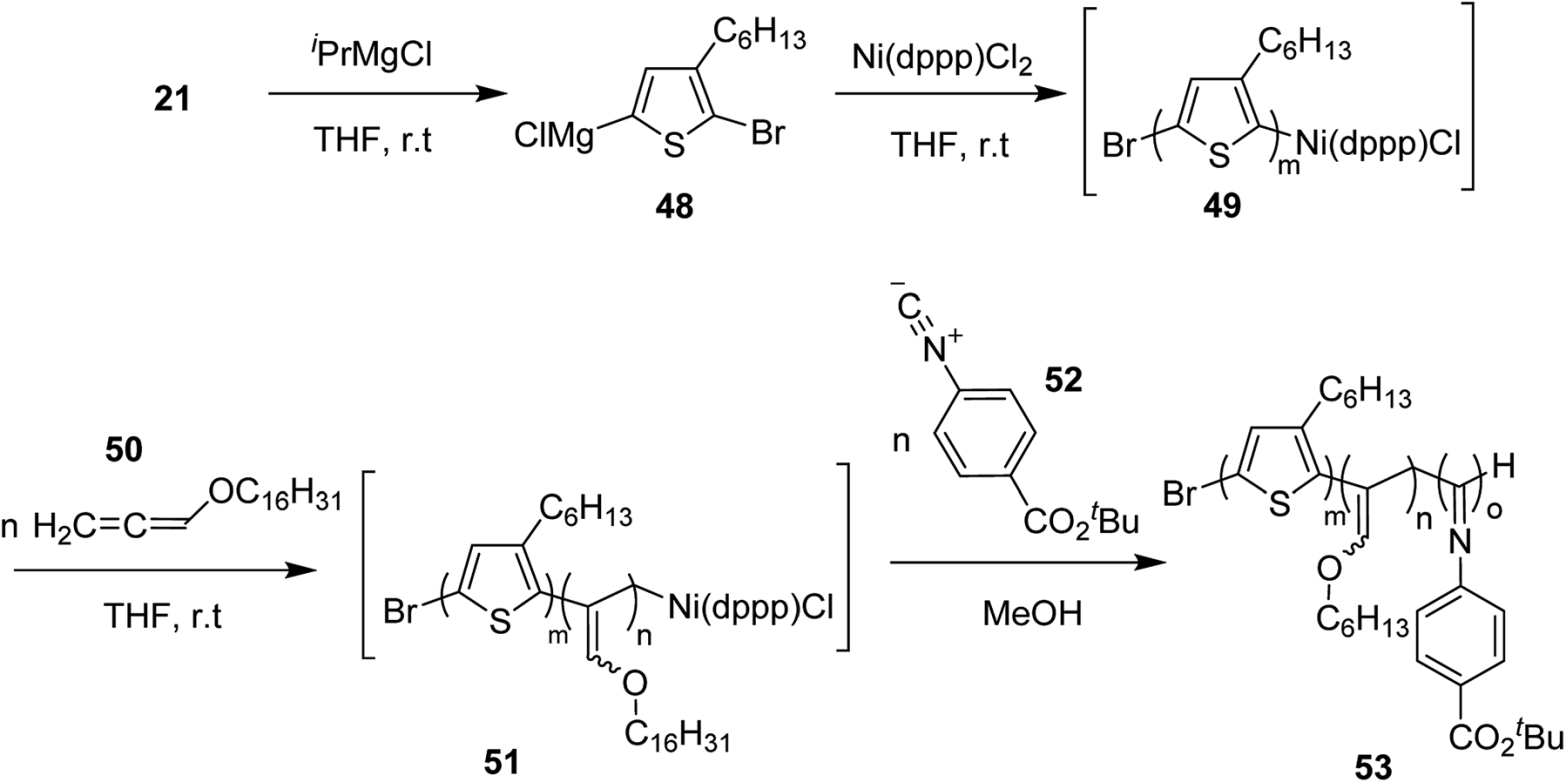
One-pot synthesis of triblock copolymer 53 containing poly(3-hexylthiophene) (P3HT), poly(hexadecyloxy allene) (PHA) and poly(phenyl isocyanide) (PPI) blocks.

Ni(ii)-terminated P3HT 49 was first prepared through the polymerization of 2-bromo-3-hexyl-5-chloromagnesiothiophene 48 with Ni (dppp)Cl_2_ [dppp = 1,3-bis(diphenylphosphanyl)propane] as a catalyst in THF at room temperature, following the Kumada catalyst-transfer polymerization (KCTP) mechanism. After the completion of the polymerization, the polymer solution was treated with hexadecyloxyallene 50 in THF at room temperature, which was considered on the basis of its good solubility. After the formation of Ni(ii)-terminated block copolymer 51, third monomer, *tert*-butyl-4-isocyanobenzoate 52 was added to the copolymer solution under dry nitrogen atmosphere and stirred for 2 hours. Triblock copolymer 53 was afforded after the subsequent workup of the crude polymer mixture. The properties of the copolymer could be tuned by changing the sequence of the monomers in the polymer chain by changing the order of their addition (Scheme 21).

**Scheme 22:**
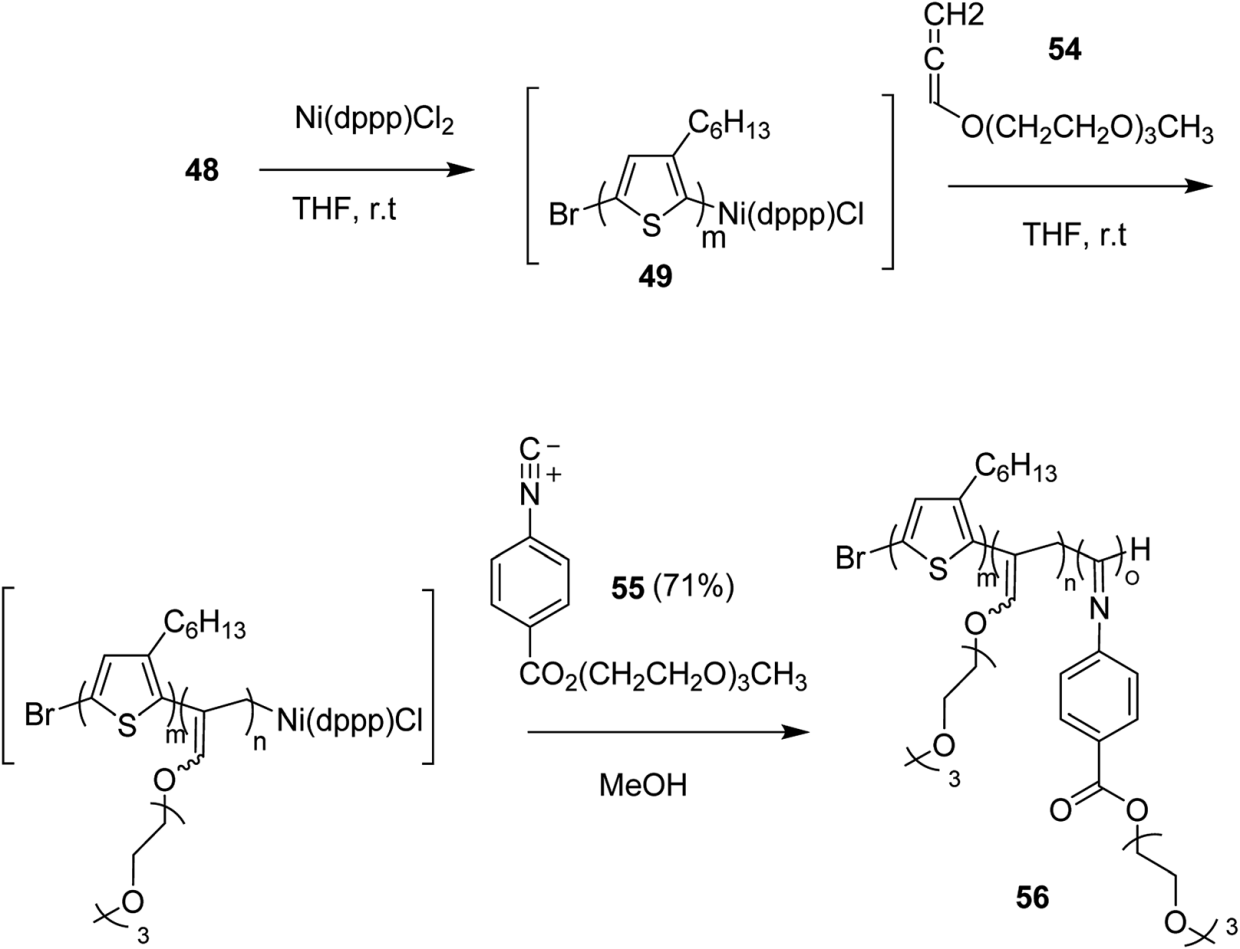
One-pot synthesis of amphiphilic triblock copolymer 56.

This synthetic protocol also proved advantageous for the synthesis of amphiphilic triblock copolymers. Monomers 48, 54 and 55 were incorporated to prepare triblock copolymer 54 composed of hydrophobic P3HT, hydrophilic poly (triethylene glycol allene) and hydrophilic PPI bearing triethylene glycol monomethyl ether. Interestingly, these amphiphilic triblock copolymers exhibited tunable light emissions with response to various environmental stimuli such as pH, temperature and solvent. Remarkably, white-light emission can be readily achieved in solution, gel, and also solid state (Scheme 22).

Pan Wang and co-workers in 2018, developed sulfone-containing chiral helical polymers poly-3-(alkylsulfone)thiophene (P3AST) 63 and 64, which confirmed a new design for the creation of large Faraday effect.^212^ These polymers exhibited tunable and large Faraday rotations with absolute verdet constants up to (7.63 ± 0.78) × 10^4^ deg per T per m at 532 nm. It was determined that the magnitude and sign of the verdet constant are related to the helicity of the polymer at the measured wavelength. These magneto-optic MO values rival the present record material and also demonstrated that verdet constants can be tuned that would be challenging to obtain using present inorganic materials.

**Scheme 23:**
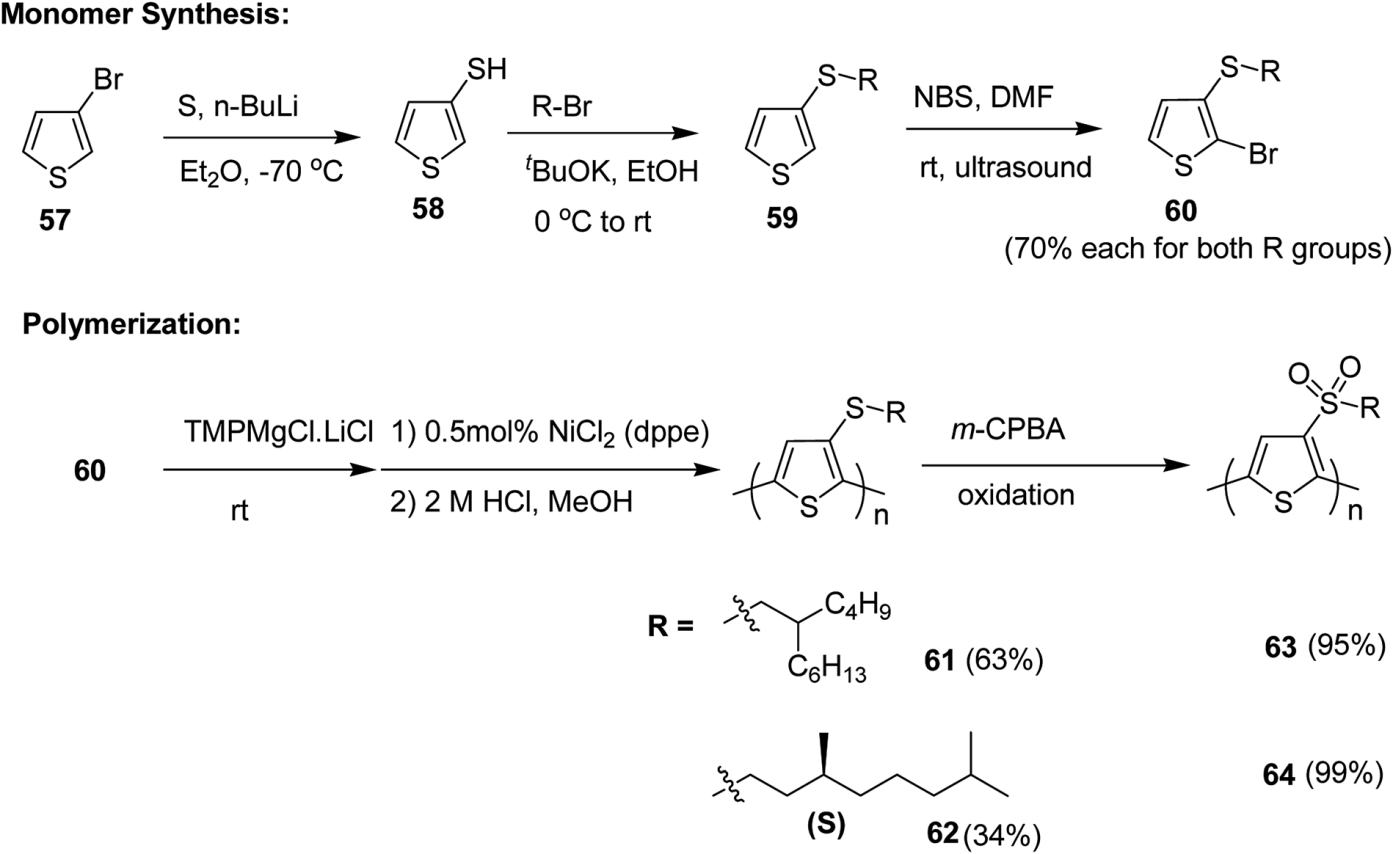
Synthetic route for head to tail regioregular P3ATTs and P3ASTs.

Synthesis of poly[3-(alkylthio)thiophene]s P3ATTs 61 and 62 and poly[3-(alkylsulfone)thiophene]s P3ASTs 61 and 62 began with a lithium–halogen exchange reaction of 3-bromothiophene 57. Subsequent quenching with sulfur yielded 3-thiophenethiol 58. 3-(Alkylthio)thiophenes 59 were obtained by reacting corresponding nucleophilic thiolate with bromoalkanes. Selective monobromination with NBS and ultrasound yielded monobrominated product 60, which was employed for Kumada polymerization. Monomer 60 was deprotonated with Knochel's base and Ni(dppe)Cl_2_ was used as a catalyst for the polymerization reaction. With this protocol, the polymers of P3ATTs having racemic side chains (*S*-P1) 61 and chiral side chains ((*S*)-*S*-P2) 62 were synthesized in 63% and 34% yields, respectively. 61 was obtained with *M*_n_ of 54.6 kDa, possessing head to tail (HT–HT) regioregularity of >96%, which is an improvement from a previously published results^213–215^ (Scheme 23).

P3ATTs were oxidized with *m*-CPBA to sulfone-containing polymers SO2-P1 63 and (*S*)-SO2-P2 64 in >99% yield with an excellent solubility in most of the organic solvents. The respective molecular weights and polydispersity indices of all the polymers are given in Table 5. The effective molecular weight of sulfone-based polymers was smaller compared to their sulfide counterparts, which might be attributed to the conformational differences or undetected degradation from oxidation *via m*-CPBA.

**Table 5:** *M*_n_ and *Đ* of the P3ATTs and P3ASTs

Polymer	*M*_n_ (kDa)/*Đ*^a^
61	54.6/1.37
63	32.7/1.42
62	11.8/1.17^b^
64	20.0/1.61

a Evaluated by GPC in THF against a polystyrene calibration at room temperature.

b Determined from the soluble part in THF at room temperature.

In 2019, Koomkoom Khawas and co-workers reported the catalytic-initiated Kumada catalyst transfer polymerization (KCTP) protocol for the synthesis of aromatic end-functionalized, defect-free poly(3-hexylthiophene), P3HTs, with controlled molecular weight.^216^ Oxidative addition of aromatic bromide to *in situ* formed Ni(0) complex of diphenylphosphino propane (dppp)_2_, generated Ni(ii) catalytic initiators 66, which were used to synthesize a series of end-functionalized P3HTs having different aromatic end groups (Scheme 21). For the synthesis of Ar-terminated P3HTs (Ar-P3HT), four aromatic bromides namely (4-bromophenoxy)(*tert*-butyl)dimethylsilane 65a, 4-bromobenzyl bromide 65b, 4-bromotoluene 65c and 4-bromoanisole 65d were employed. The reason of considering silyl protected 4-bromophenol as one of the Ar–Br is that phenolic OH end-functionalized P3HT could be obtained by simply deprotection of silyl ether after the completion of the polymerization.

**Scheme 24:**
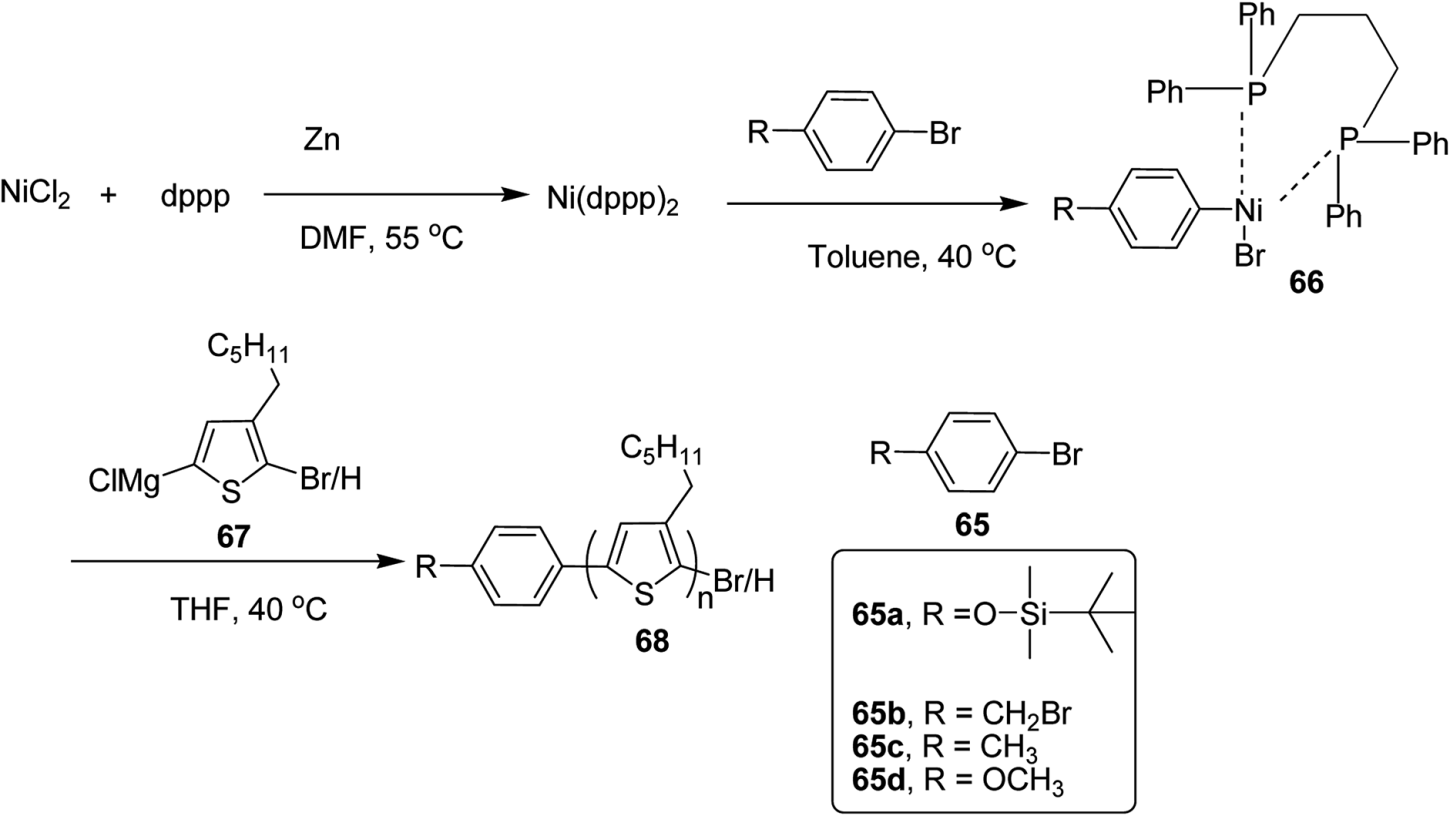
Synthesis of end-functionalized regioregular P3HTs, 68a–d*via* KCTP.

In order to synthesize nickel catalytic initiator, reduction of anhydrous NiCl_2_ was performed using zinc dust in dry DMF, which was followed by addition of diphenylphosphinopropane (dppp) under argon atmosphere to generate Ni(0) complex, Ni(dppe) *in situ*. Then the solution of aryl bromides 65a–d were added dropwise to the solution of Ni(0) complex at room temperature to synthesize Ni(ii) catalytic initiators 66. 2-Bromo-5-chloromagnesio-3-hexylthiophene 67 was synthesized from 2-bromo-3-hexyl-5-iodothiophene by metal exchange reaction using isopropylmagnesium chloride. For the polymerization, Ni(ii) complex solution was added to the Grignard reagent solution of the monomers to obtain the corresponding polymers 68a–d. Corresponding to 65a, 65b, 65c and 65d respectively (Scheme 24). An overview of the properties of the polymers has been provided Table 6.

**Table 6:** Characterization of differently end-terminated P3HT samples synthesized by different catalytic initiators

P3HT	NiCl_2_ (mol%)	*M*_n_ (NMR)	*M*_n_ (GPC)	PD1	Yield (%)
66a	1.85	9853	7438	1.15	71
66b	0.70	26 887	23 260	1.30	68
66c	2.08	7849	6958	1.22	55
66d	3.34	5511	5010	1.21	62

The versatile nature of the catalytic initiators consisting of different aromatic groups was exhibited by the initiation of polymerization and these initiators are believed to be useful for the synthesis of different P3HT based architectures and polymer brushes. This protocol is considered to be highly useful for large scale synthesis of conjugated block copolymers, end-functionalized P3HTs and complex architectures of P3HT, as all the steps are *in situ*, well connected to each other and do not require any separation and purification of the intermediate compounds. Molecular weight of the polymer could easily be controlled by varying the amount of NiCl_2_ with respect to the feeding monomer.

In 2019, Christoph Horn and co-workers reported the synthesis of novel side-chain semi fluorinated thiophene monomer 69, synthesized by reacting 3-thiophene ethanol with 4,4,5,5,6,6,7,7,8,8,9,9,9-tridecafluorononan-1-ol (Scheme 22).^217^ Kumada catalyst transfer polycondensation protocol was employed to synthesize regioregular side-chain semifluorinated thiophene polymer P3sfT 75 with narrow polydispersity index of 1.11, number average molecular weight (*M*_n_) of 25 900 g mol^−1^ and high regioregularity (>98%). P3sfT exhibited high self assembly and crystallinity in the solid state. The order is even more pronounced compared to the P3HT because of the extension of the side chains by fluorinated methylene groups. It was concluded that the order of the backbone polymer was enhanced due to the stronger self-organization of side chains, which resulted in the strong formation of layered structure as well as π–π stacking. P3sfT 75 showed high potential as a semiconductor in organic electronics due to its high self-assembly.

**Scheme 25:**
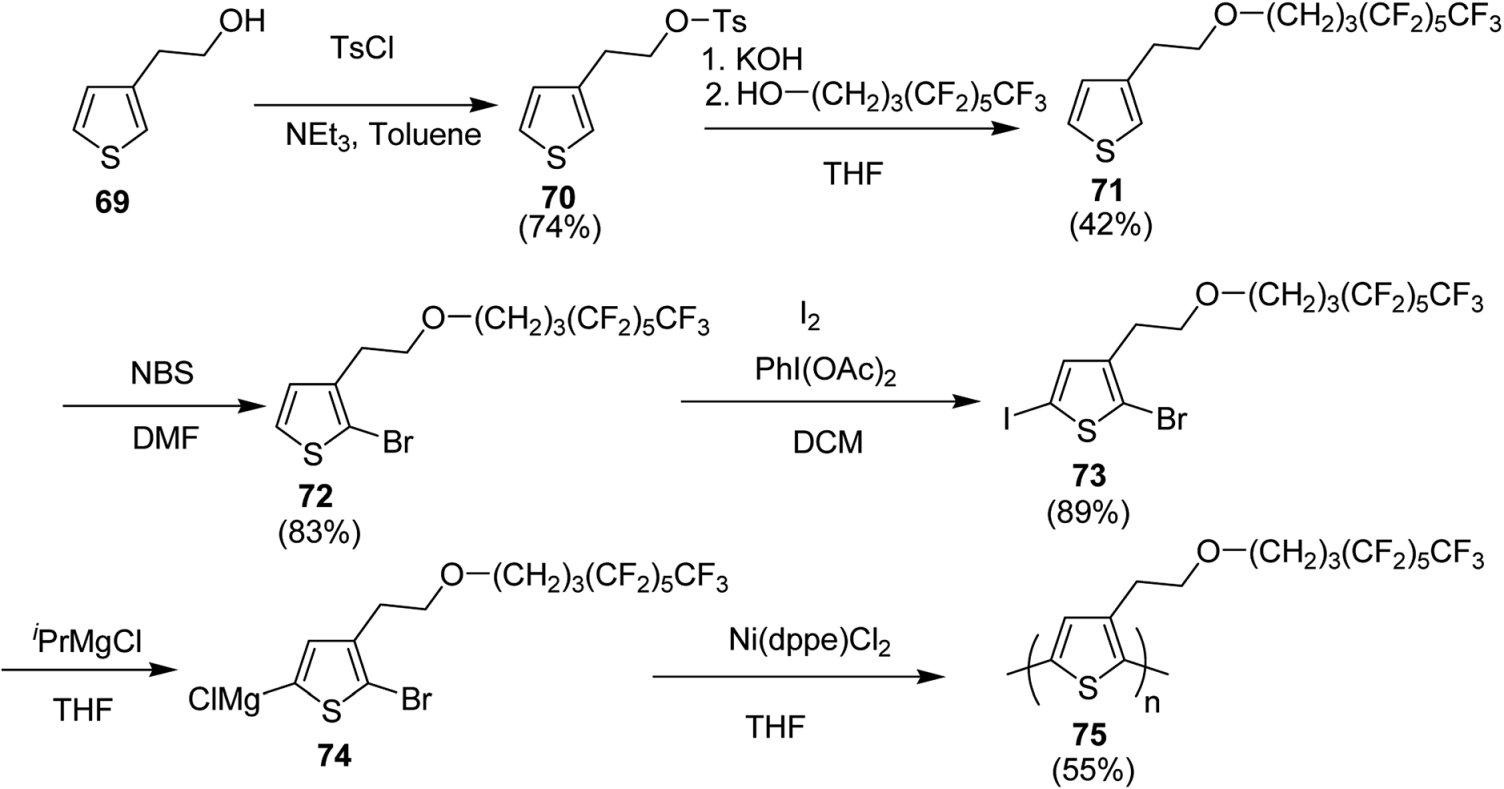
Synthetic route towards the synthesis of side-chain semi fluorinated polythiophene 75.

Synthesis of P3sfT 75 started with 3-thiophenmethanol 69, activated by the tosyl chloride to give 70 in order to facilitate a nucleophilic reaction. Fluorinated side chain was incorporated by the reaction with H3F6 alcohol 4,4,5,5,6,6,7,7,8,8,9,9,9-tridecaflurornonan-1-ol in the presence of potassium hydroxide (KOH) which provided partial deprotonation of the alcohol. Side-chain semifluorinated thiophene 71 was selectively halogenated at position-2 using 1 equivalent of NBS to afford 72, followed by the iodation at position-5, for the selective magnesio-halogen exchange to yield 2-bromo-5-chloromagnesio-3-(2-((4,4,5,5,6,6,7,7,8,8,9,9,9-tridecafluorononayl)oxy)ethyl)thiophene 74. Regioselectivity of P3sfT was enhanced by the asymmetric nature of the monomer precursor 73. Ni-catalyst, Ni(dppe)Cl_2_ was employed to polymerize the Grignard compound of the monomer 74 to yield polymer 75 (Scheme 25).

### 3.3. Ni-catalyzed Suzuki, Murahashi and Negishi coupling polymerization

**Scheme 26:**
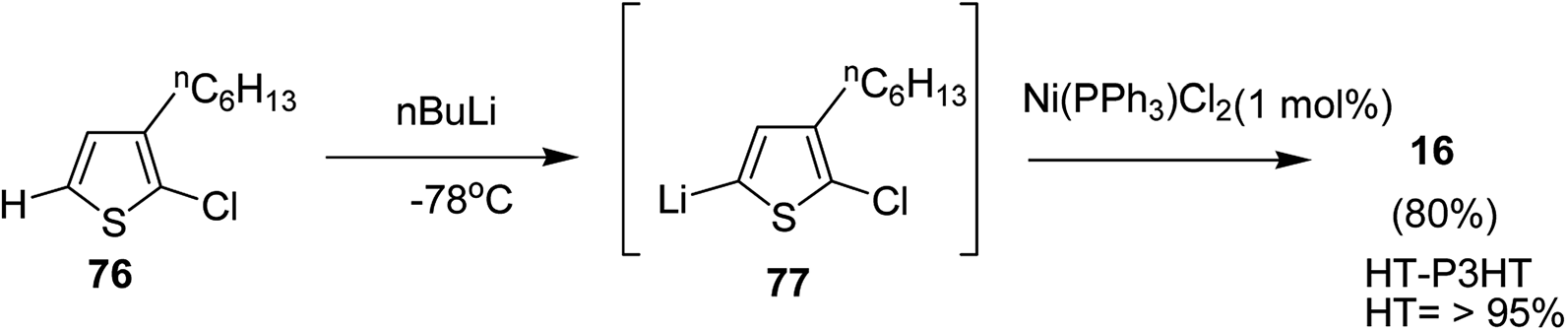
Murahashi coupling polymerization of 2-chloro-3-hexylthiophene monomer 76.

In 2013, Kanta Fuji and co-workers explored Murahashi coupling as a versatile preparative tool for polycondensation of hetero arylenes having extended π-conjugation.^218^ Nickel catalyst bearing an N-heterocyclic carbene (NHC) ligand was shown to be highly effective in contrast with the previous reports, in which palladium was an effective catalyst for Murahashi coupling whereas nickel was considered to be less effective transition metal catalyst. Three classes of lithiations including lithium–bromine exchange, deprotonation and halogen dance were exhibited to form lithiated monomers, which were subsequently polymerized using Ni–NHC complex to obtain the corresponding polymers. Only chlorothiophene 76 underwent direct lithiation whilst the polymerization of bromothiophene did not give desired results. Ni-catalyst, NiCl_2_(PPh_3_)IPr [IPr = 1,3-bis(2,6-diisopropylphenyl)imidazole-2-ylidene] proved highly effective in polymerizing the lithiated chlorothiophene monomer 77 to yield poly(3-hexylthiophene) (P3HT) 16 in 80% isolated yield with polydispersity index (PDI) of 1.93 and number-average molecular weight (*M*_n_) of 10 100 g mol^−1^. Improved polydispersity index was achieved by using cyclopentyl methyl ether (CPME) as a solvent under similar conditions (PDI = 1.35, *M*_n_ = 12 500 g mol^−1^) (Scheme 26).

**Scheme 27:**
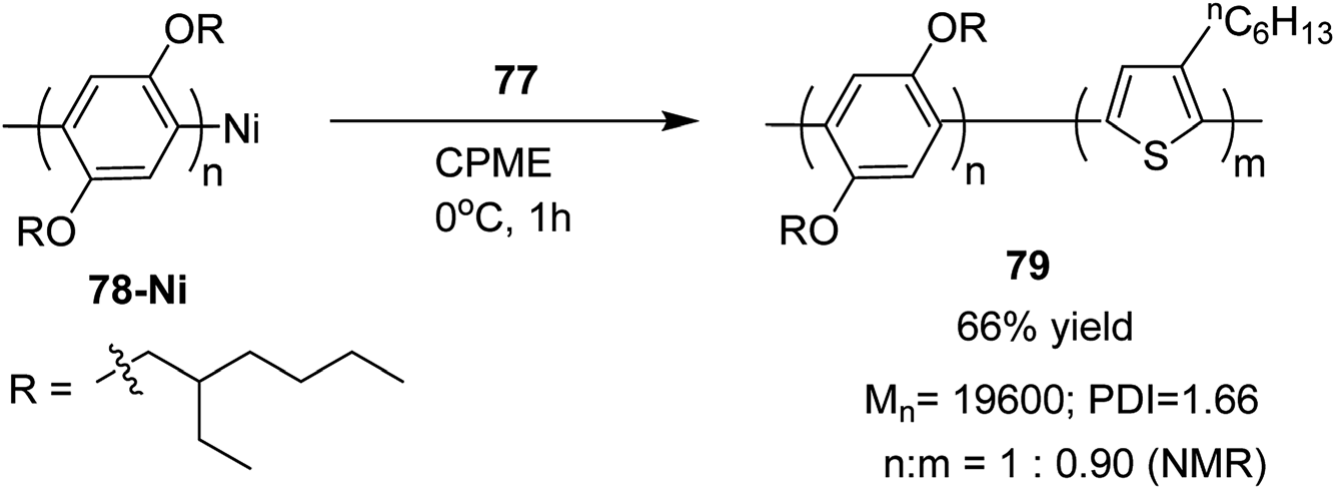
Block copolymerization of monomer 77 and 78*via* Murahashi coupling polycondensation.

Block copolymer of lithiated monomer could be obtained by an end functionalization of 78. Corresponding block copolymer 79 was obtained by addition of lithiated chlorothiophene to the reaction mixture containing polyarylene 78. In copolymer, monomer ratio (polyarylene/polythiophene) was 1 : 0.90, which was confirmed by ^1^H NMR analysis (Scheme 27).

**Scheme 28:**
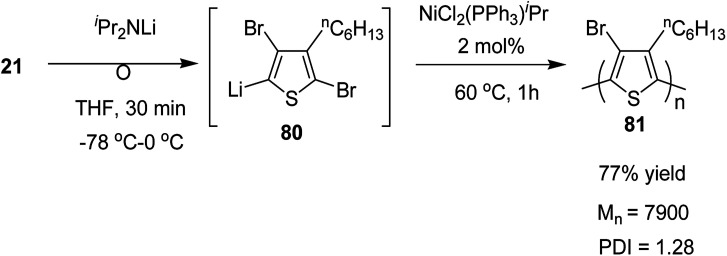
Murahashi coupling polycondensation of 21*via* halogen dance.

Organolithium species, generated by halogen dance rearrangement, was also found to undergo Murahashi coupling polymerization to yield a new class of polythiophene. Treatment of 2,5-dibromo-3-hexylthiophene 21 with lithium diisopropylamide (^i^Pr_2_NLi) in THF induced halogen dance rearrangement at −78 to 0 °C leading to the formation of 4-lithiated intermediate 80, which, upon addition of NiCl_2_(PPh_3_)^i^Pr, afforded the corresponding polythiophene 81 in 77% yield bearing a bromine substituent at position 4 of the thiophene. It could lead to further transformations of the C–Br bond (Scheme 28).

Negishi-type catalyst-transfer polycondensation (NCTP) protocol was introduced by Goto and co-workers in 2014 for the synthesis of P3HT-*b*-poly(3-octadecylthiophene), using zincate complex, Bu_4_ZnLi_2_.^219^ Furthermore, two-stage polymerization of poly(3-hexylthiophene) was also achieved by NCTP using zincate complex. Polymerization results of NCTP using Ni catalysts with varied phosphine ligands were found to be strongly influenced by electron donating ability of ligands as well as steric hindrance based on the factors of bite angle and cone angle of Ni-catalysts.

**Scheme 29:**
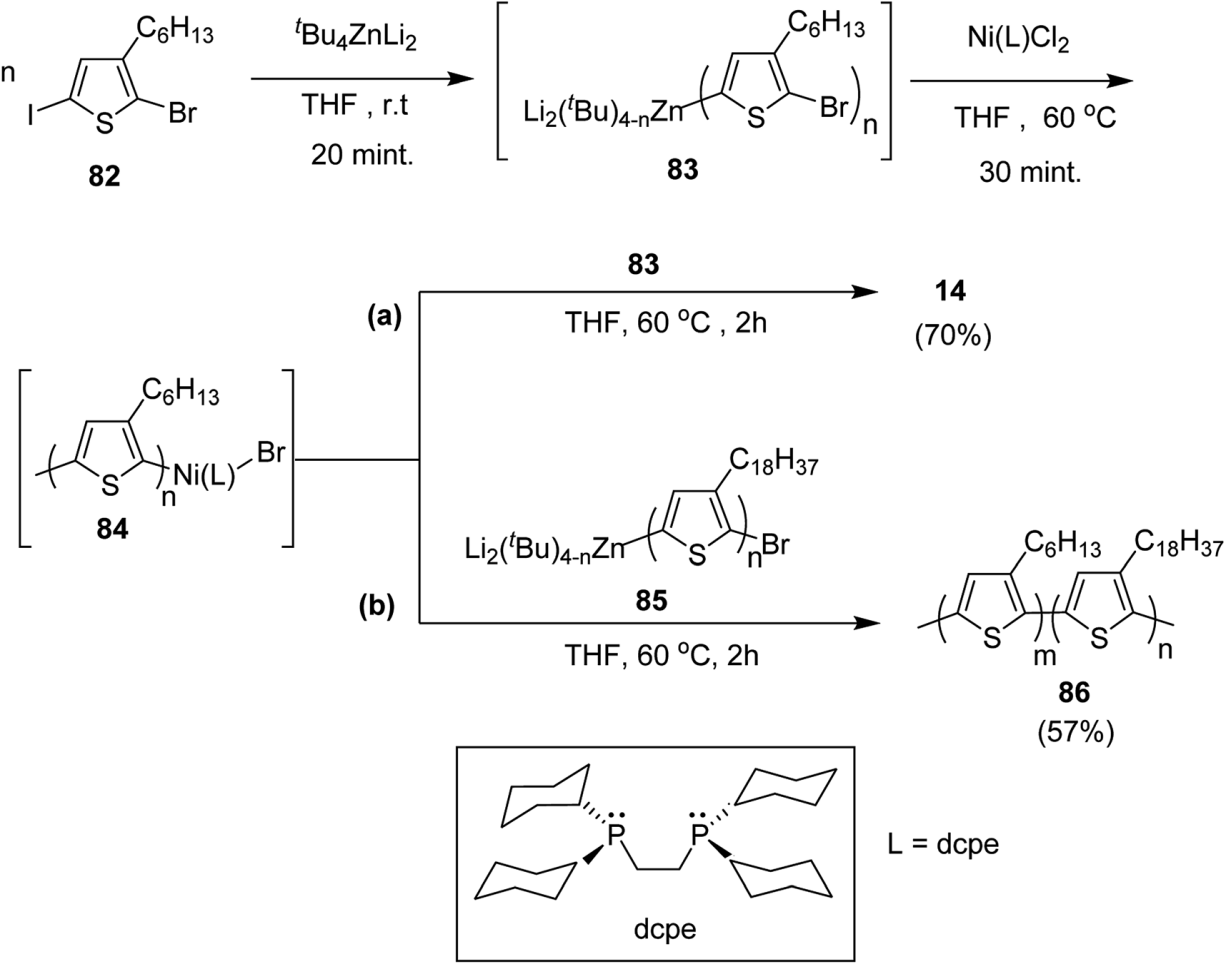
Synthesis of (a) P3HT 14 by two-stage NCTP and (b) 86 by NCTP.

2-Bromo-5-iodo-3-hexylthiophene 82 was treated with zincate complex dissolved in THF to afford 83 (Scheme 29). Finally, Ni-catalyst (0.0114 mmol) solution, prepared by mixing Ni(PPh_3_)_2_Cl_2_ (9.4 mg, 0.014 mmol) and bis(dicyclohexylphosphino)ethane (dcpe) (11.1 mg, 0.0263 mmol) in THF (5 mL), was added to start the polymerization, which resulted in the formation of P3HT-Ni (L)-Br 84.

Living nature of NCTP method was confirmed by adding monomer 82 to the remaining solution of the first P3HT block. Increase in the number average molecular weight from *M*_n_ = 5060 g mol^−1^ to *M*_n_ = 16 100 g mol^−1^ confirmed efficient chain extension. The same two stage-polymerization protocol was applied to synthesize block copolymer of 3-hexylthiophene with 3-octadecylthiophene using 85 as a second monomer. P3HT-*b*-poly(3-octadecylthiophene) 86 with *M*_n_ of 15 000 and dispersity index of 1.08 was afforded with 3HT/3ODT molar ratio of 42/58.

**Scheme 30:**
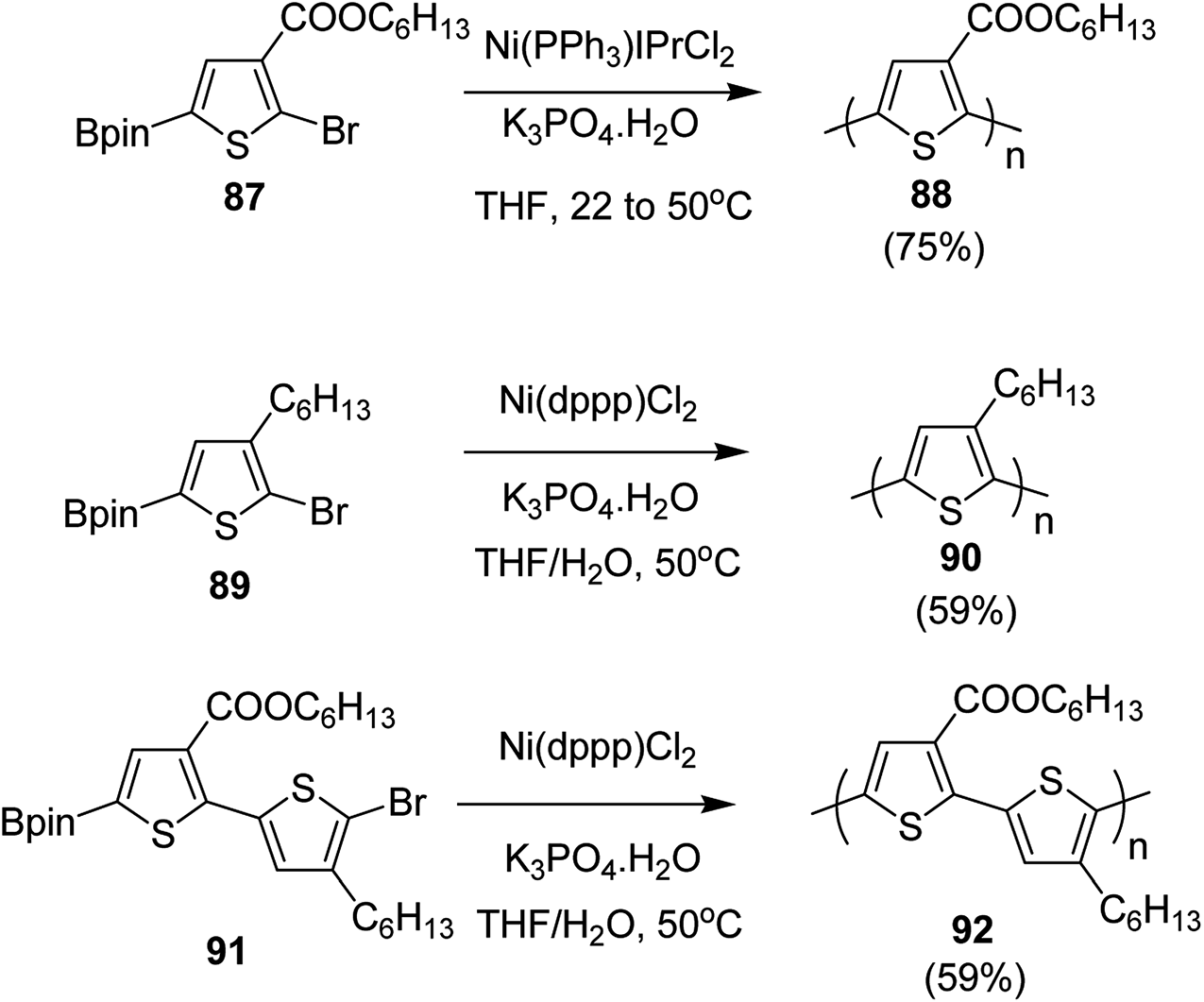
Ni-catalyzed Suzuki CTP to synthesize 88, 90 and 92.

In 2016, Yunyan Qiu and co-workers for the very first time, reported chain growth Suzuki cross coupling for catalyst-transfer polycondensation of ester-functionalized thiophene, employing commercially available nickel precatalysts (Scheme 30).^220^ This protocol was exploited for the controlled synthesis of poly(hexylthiophene-3-carboxylate) P3HET (88) and poly(3-hexylthiophene) P3HT (90). Borylation of the thiophene ring of the monomers with pinacolborane was achieved using an iridium-catalyzed C–H borylation reaction.^221^ Versatility of this method was also illustrated by synthesizing alternating copolymer 92 from borylated thiophene monomer and 3-hexylthiophene. It was revealed that water was necessary for promoting the controlled polymerization of all three monomers. Water from K_3_PO_4_·H_2_O was found to be sufficient for promoting the controlled reaction of monomer 87, while additional water resulted in an excellent control over dispersity and molecular weight of the polymers, produced from monomers 89 and 91.

## 4. Pd-catalyzed synthesis of thiophene-based polymers

Palladium-based catalysts are one of the most efficient catalysts for cross-coupling reactions known so far. An avalanche of data is available regarding Pd-catalyzed reactions and progress is still continued. Palladium catalysis has played a vital role in the synthesis of conjugated polymers and various Pd-catalyzed cross-coupling reactions, especially Suzuki and Stille couplings, are widely used in the synthesis of monomers as well as polymers. Some of the recent progress in synthesis of polythiophenes using different Pd-catalysts is reviewed below.

### 4.1. Pd-catalyzed Suzuki-coupling polymerization

Kazuyoshi Watanabe and co-workers in 2012 reported the synthesis of new derivatives of polythiophenes and their phenylene copolymers *via* introduction of chiral alkoxy substituents into their side chains.^222^ These polymers exhibited fluorescence ranging from blue to red in films and from blue to orange in chloroform solutions. Enantiotropic main-chain liquid crystallinity was also shown by these polymers at elevated temperatures. For the polymers comprising upto three aromatic rings in repeating units, bisignate cotton effect was observed in π–π* transition region of CD spectra due to the formation of polymer assembly with an interchian helically π-stacked structures.

**Scheme 31:**
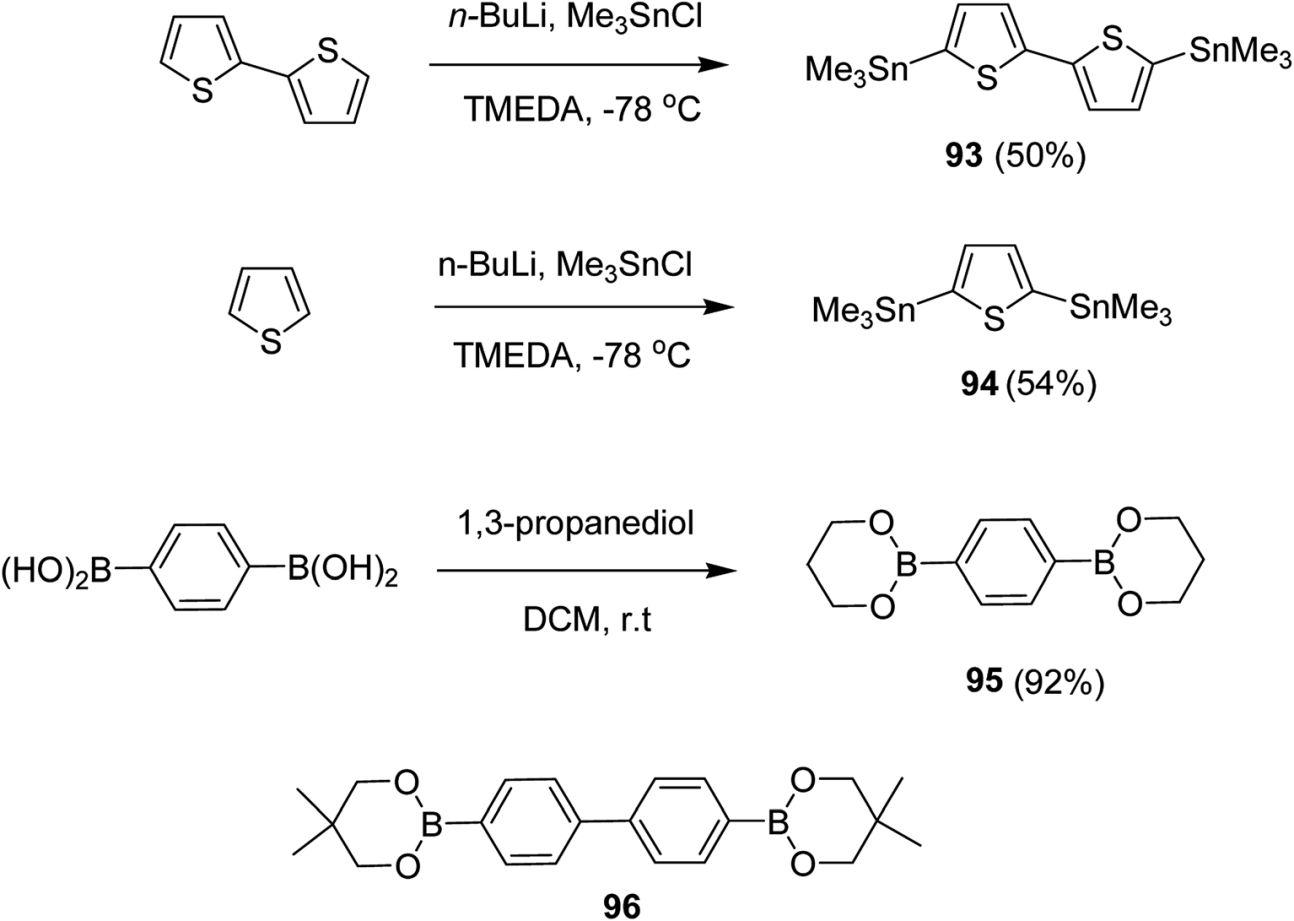
Synthetic doutes for the synthesis of thiophene- and phenylene-based monomers 93–96.

NBS was used to brominate 2 and 5-position of 3-thiophenecarboxylic acid 97 to afford 2,5-dibromo-3-thiophenecarboxylic acid 98, which underwent esterification with (*R*)-(−)-(2) and (*S*)-(+)-(2)-nonanol with the help of triphenylphosphine (TPP) and diethylazodicarboxylate (DEAD) to afford *R*- and *S*-2 99. In the similar fashion, esterification of 2,5-dibromobenzoic acid 105 was carried out with (*R*)-(−)-(2) and (*S*)-(+)-(2)-nonanol to yield *R*- and *S*-3 106. 5,5′-Bis(trimethylstannyl)-2,2′-bithiophene 93 was synthesized by lithiating bithiophene using *n*-BuLi and subsequent quenching with trimethyltinchloride. 2,5-Bis(trimethylstannyl)thiophene 94 was synthesized following the same method. 1,4-Phenylenediboronic acid bis(1,3-propanediol)cyclic ester 95 was synthesized from 1,3-propanediol and 1,4-phenylenediboronic acid (Scheme 31).

**Scheme 32:**
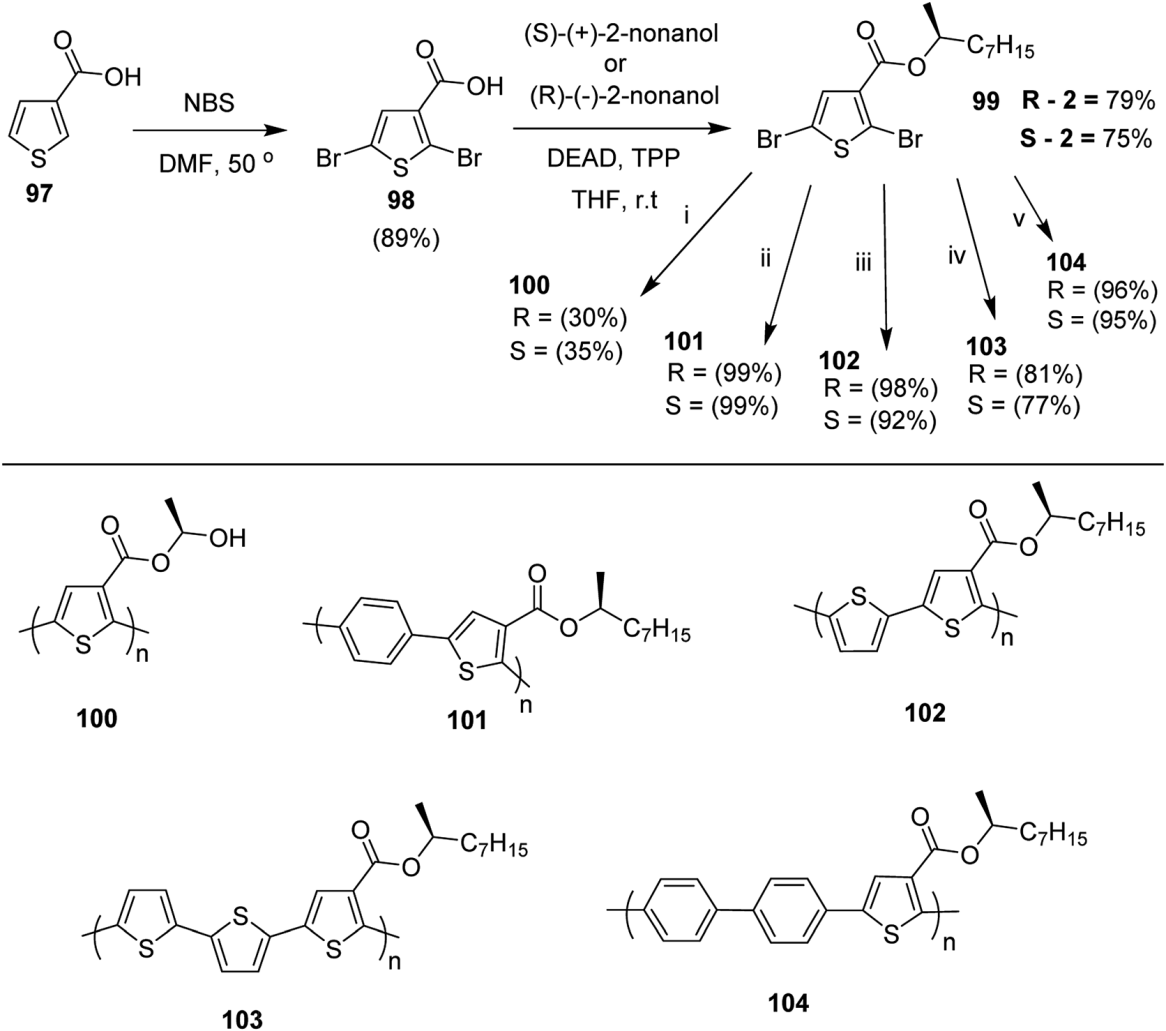
Synthesis of polythiophene and thiophene-based copolymers with chiral nonyloxy carbonyl substituents. Reagents and conditions: (i) Ni(cod)_2_, bpy, DMF, 80 °C; (ii) 95, Pd(PPh_3_)_4_, NaHCO_3_, THF, H_2_O, reflux; (iii) 94, Pd_2_(dba)_3_, TFP, THF, 50 °C; (iv) 93, Pd_2_(dba)_3_, TFP, THF, 50 °C; (v) 96, Pd(PPh_3_)_4_, NaHCO_3_, THF, H_2_O, reflux.

**Scheme 33:**
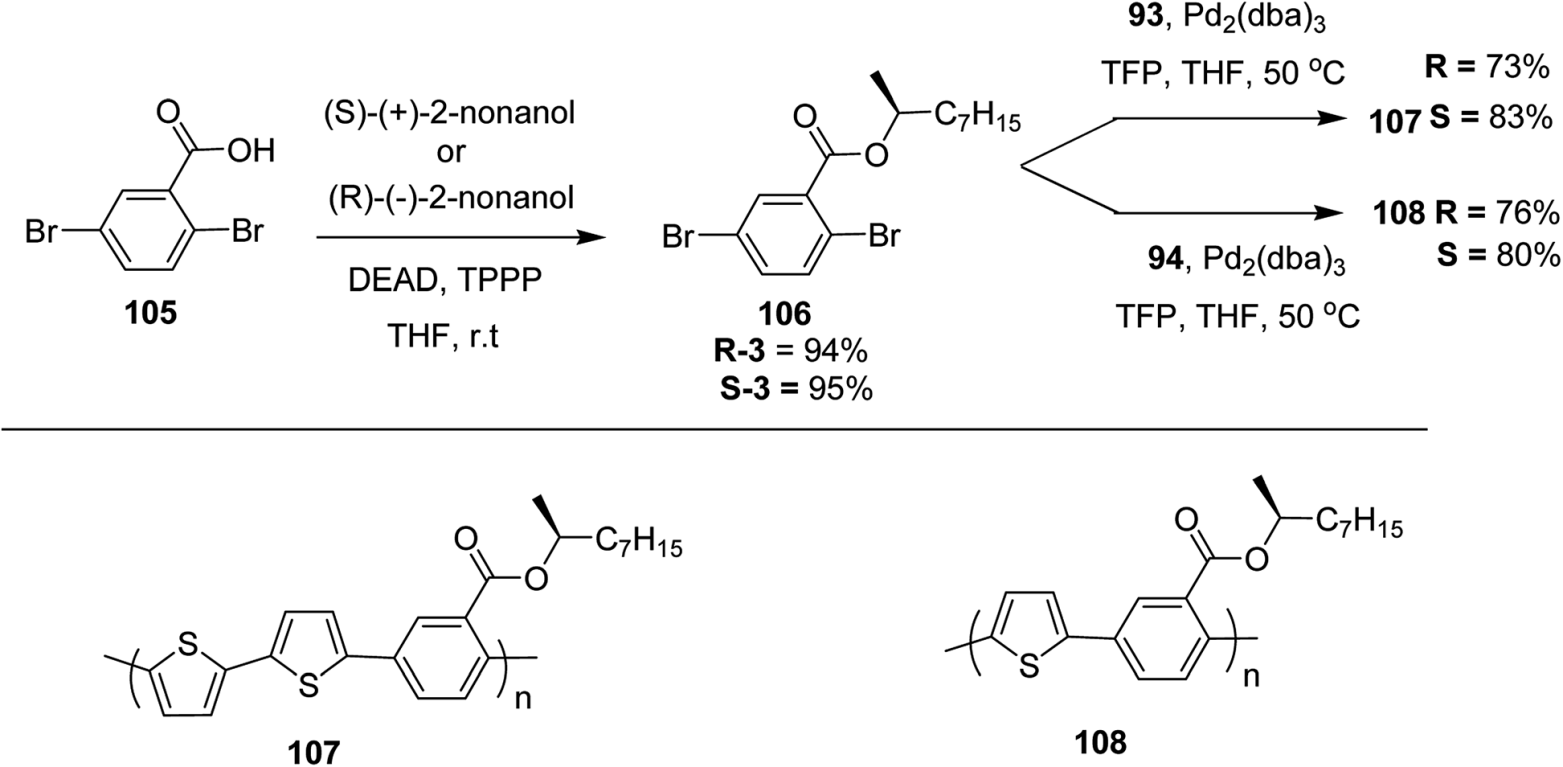
Copolymerization of thiophene and phenylene monomers to synthesize copolymers 107 and 108.

Suzuki coupling polymerization reaction was employed to copolymerize *R*- and *S*-2 *R*/*S*-2 99 with 4,4′-biphenyldiboronic acid, bis(neopentylglycol)cyclic ester 96 using Pd(PPh_3_)_4_ as a catalyst to synthesize *R*- and *S*-P (PPT*) 104. Similar procedure was used to copolymerize *R*- and *S*-2 with 95 to afford *R*- and *S*-P(PT*) 101. Pd_2_(dba)_3_ as a catalyst was used to copolymerize *R*- and *S*-3 with 93 to yield *R*- and *S*-P (TTP*) 107, *via* Stille coupling reaction. *S*- or *R*-P(TP*) 108, P (TT*) 102 and P (TTT*) 103, were synthesized *via* Stille coupling polycondensation. Yamamoto polymerization process was used to synthesize *R*- or *S*-P (T*) 100 from *R*- or *S*-2, using Ni(cod)_2_ catalyst (Schemes 32 and 33).

**Scheme 34:**
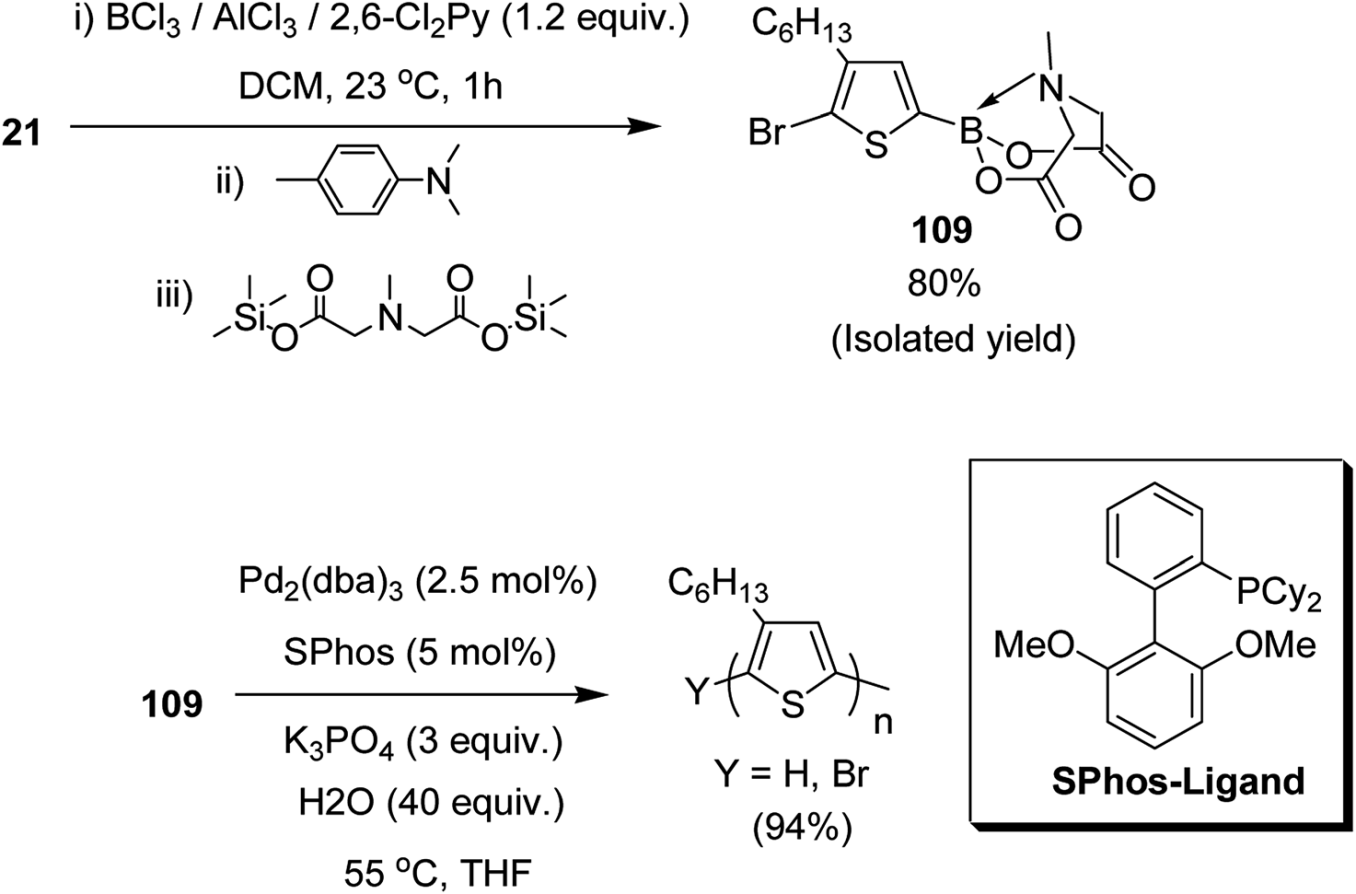
One-pot synthetic route to 109 by direct electrophilic borylation and esterification with TMS_2_–MIDA and polymerization of 109.

Josue Ayuso and co-workers in 2015 reported the synthesis of highly pure *N*-methyliminodiacetic acid (MIDA) boronate ester thienyl monomer 109*via* direct electrophilic borylation of 2,5-dibromo-3-hexylthiophene 21, and its subsequent use to obtain regioregular poly(3-hexylthiophene-2,5-diyl), rr-P3HT, by Suzuki–Miyaura polymerization.^223^ This approach provides a simple route towards the synthesis of bifunctional monomers required for polymerization reaction, and also avoids the use of unstable boronic acid intermediates during their synthesis. The rigid tridentate MIDA group binds boron strongly to provide an exceptional stability to even electron rich heteroaryl boronate esters under acidic conditions. The hydrolysis of MIDA boronate esters proceeded slowly under mild basic conditions to gradually unmask the active boron transmetallating agent. In this way, the concentration of the sensitive boron species was minimized in the reaction mixture, which in turn, is helpful in reducing undesired competitive side reactions such as protodeboronation (Scheme 34).

**Scheme 35:**
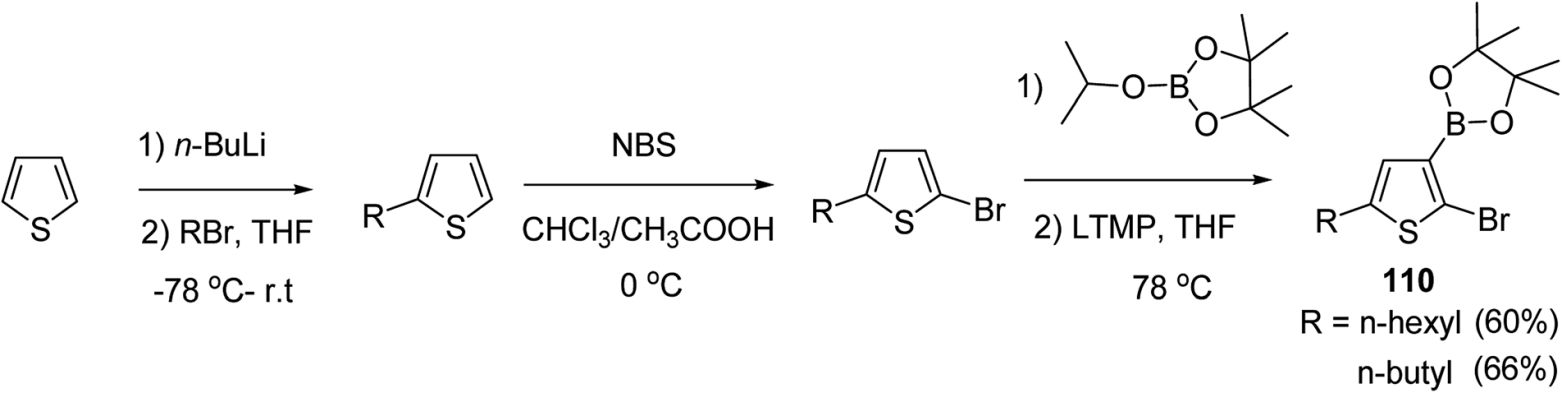
Synthetic route for the synthesis of monomer 2-bromo-5-alkylthiophen-3-ylboronic acid pinacol ester 110.

In 2016, Hong-Hai Zhang and co-workers reported the first synthesis of regioregular poly(5-alkyl-2,3-thiophene)s (P5HT) 111, which is an *ortho*-linked isomer of a well-known conjugated polymer, poly(3-alkyl-2,5-thiophene) (P3HT), *via* Suzuki–Miyaura cross-coupling polycondensation, using PEPPSI-IPr as a catalyst.^224^ Strong repulsion originating from highly angled connections made this synthesis quite challenging, and very few examples of the synthesis of poly(*o*-arylene)s *via* direct polymerizations have been reported so far.^225–229^ Commercially available thiophene was used to synthesize 2-bromo-5-alkylthiophen-3-ylboronic acid pinacol ester 110 through three steps, consisting of alkylation using *n*-butyl lithium, bromination with NBS and then boronation in gram scale quantities. *In situ* quenching strategy was used to prevent halogen dance rearrangement during the borylation step (Scheme 35).

Commercially available PEPPSI-IPr was found to be the best overall catalyst, affording polymers 111a,b with narrow polydispersity index and tunable molecular weight (Table 7). The comparison of UV-visible absorption of P5HT (*λ* = 345 nm) with that of P3HT (*λ* = 450 nm) showed low degree of conjugation in P5HT than in P3HT, which might be a result of helical geometry of the P5HT 111 compared to the P3HT's more planar geometry. Moreover, 111 was also reported to produce green fluorescence under UV irradiation (*λ* = 360 nm).

**Table 7:** Suzuki cross-coupling polymerization of different monomers at the gram scale catalyzed by PEPPSI-IPr

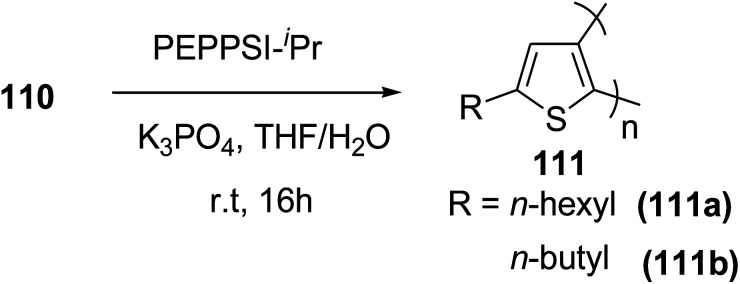				
Entry^a^	R	Yield^b^ (%)	*M*_n_ c	PDI^c^
111a	*n*-Hexyl	70	6400	1.35
111b^d^	*n*-Butyl	67	5500	1.42

a Polymerization was performed in gram scale, monomer (4 mmol), PEPPSI-IPr (4 mol%), K_3_PO_4_ (40 mmol), THF (240 mL), H_2_O (12 mL).

b Isolated yield.

c GPC analysis (polystyrene as standard, THF, 40 °C).

d 2.5% PEPPSI-IPr was used.

**Scheme 36:**
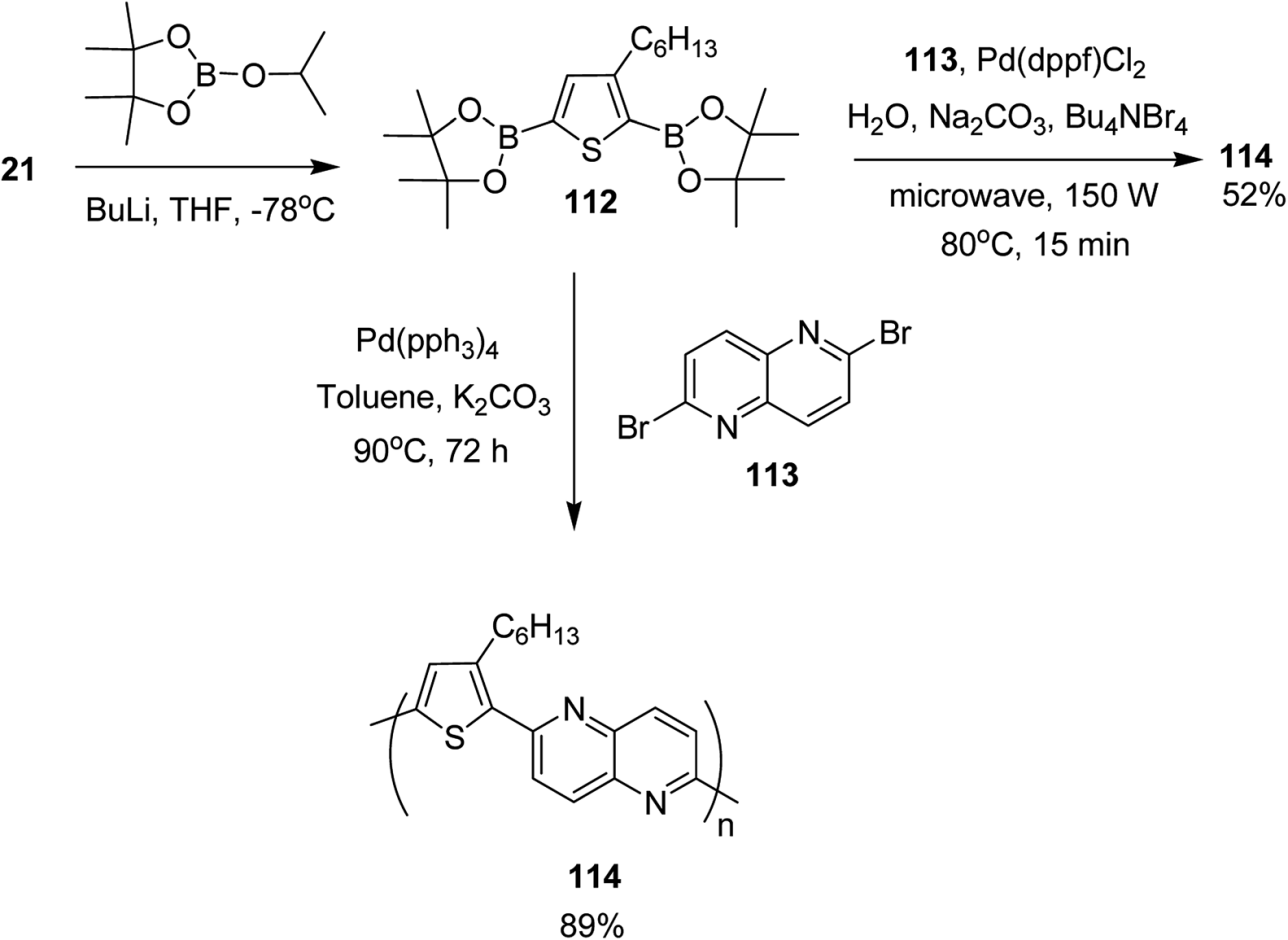
Synthetic route for the synthesis of polythiophene-based copolymer 114.

In 2017, Mohammad N. Siddiqui and co-worker reported the synthesis of poly[1,5-naphthyridine-(3-hexylthiophene)] 114, a semi conducting polymer, by employing both microwave-assisted and conventional Suzuki cross-coupling reactions between 3-hexylthiophene-2,5-diboronic ester 112 and 2,6-dibromo-1,5-naphthyridine 113 (Scheme 36).^230^ Optical bandgap for this copolymer was calculated to be 2.26 eV. It was employed in dye-sensitized solar cells (DSSCs) as photosensitizer. The solar cell under AM 1.5 G illumination at 100 mW cm^−2^ showed power conversion efficiency (PCE) of 0.67% with a short circuit current (*J*_sc_) of 2.0 mA cm^−2^, a fill factor (FF) of 55% and an open circuit voltage (*V*_oc_) of 621 mV.

Monomer 112, 2,5-bis(4,4,5,5-tetramethyl-1,3,2-dioxoborolane)-3-hexylthiophene, was synthesized by slow addition of *n*-butyllithium to the solution of 2,5-dibromo-3-hexylthiophene 21 in THF and stirring the resulting mixture at −78 °C for 2 hours. Afterward, 2-isopropoxy-4,4,5,5-tetramethyl-1,3,2-dioxoborolane was added into the reaction mixture and the reaction was stirred at −78 °C for additional 1 hour, which was followed by an overnight stirring at room temperature. The reaction mixture was extracted with DCM and purified by column chromatography to obtain the monomer 112.

In order to synthesize the polymer 114*via* conventional Suzuki coupling protocol, Pd(PPh_3_)_4_ was added to the solution of 113 in toluene, and the reaction mixture was stirred for 72 hours at 90 °C. The reaction mixture was extracted with ethylacetate after cooling to the room temperature, followed by washing with acetone and methanol by Soxhlet extraction for a day to yield polymer 114 in 89%. Microwave-assisted polymerization of the monomers 112 and 113 was carried out by adding tetra-*n*-butylammonium bromide and Na_2_CO_3_ to the solution of 112 and 113 in H_2_O in a microwave tube. Pd(dppf)Cl_2_ was added after bubbling nitrogen gas through the solution and the reaction mixture was heated at 80 °C for 15 minutes. Polymer 114 was obtained in 52% yield after washing with CH_3_OH and decanting.

**Scheme 37:**
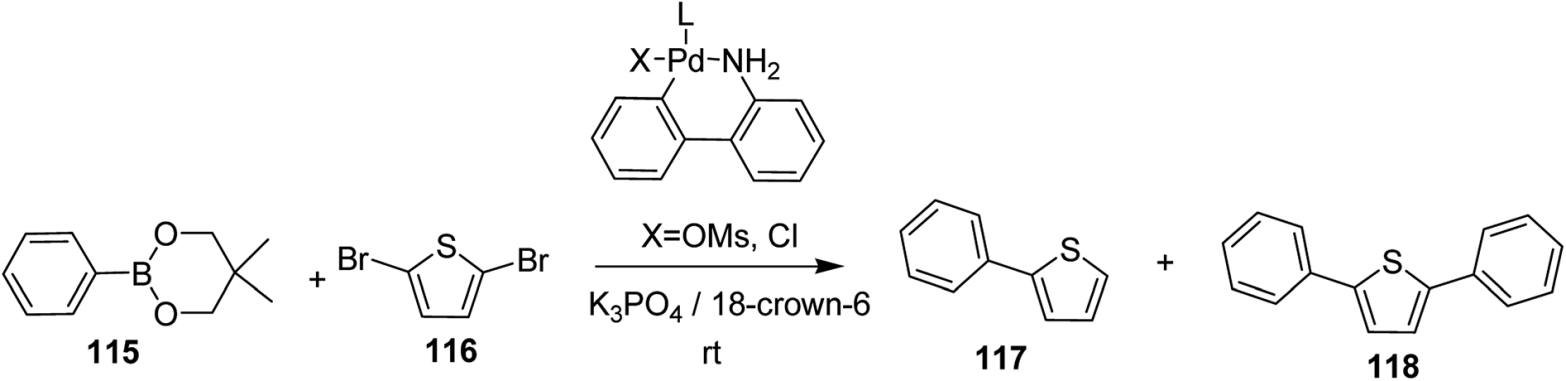
Model reaction of 2,5-dibromothiophene 116 with phenylboronic acid ester 115.

In 2018, Kantaro Kosaka and co-workers investigated several palladium catalysts having bulky phosphine ligands other then tertiary tributylphosphine (*t*-Bu_3_P) for Suzuki catalyst transfer condensation polymerization.^231^ Reaction of 2,5-dibromothiophene 116 with diphenylboronic acid ester 115 was chosen as a model reaction to investigate the variety of Pd precatalysts. It was found that diphenyl-substituted thiophene was formed exclusively when di-*tert*-butyl(4-dimethylaminophenyl)phosphine (AmPhos) was used. Disubstituted thiophene 118 was formed preferentially compared to the monosubstituted thiophene 117, because the intramolecular transfer of Pd catalyst on the thiophene occurred after the first substitution with 115, demonstrating high transfer ability of AmPhos Pd catalyst (Scheme 37).

**Scheme 38:**
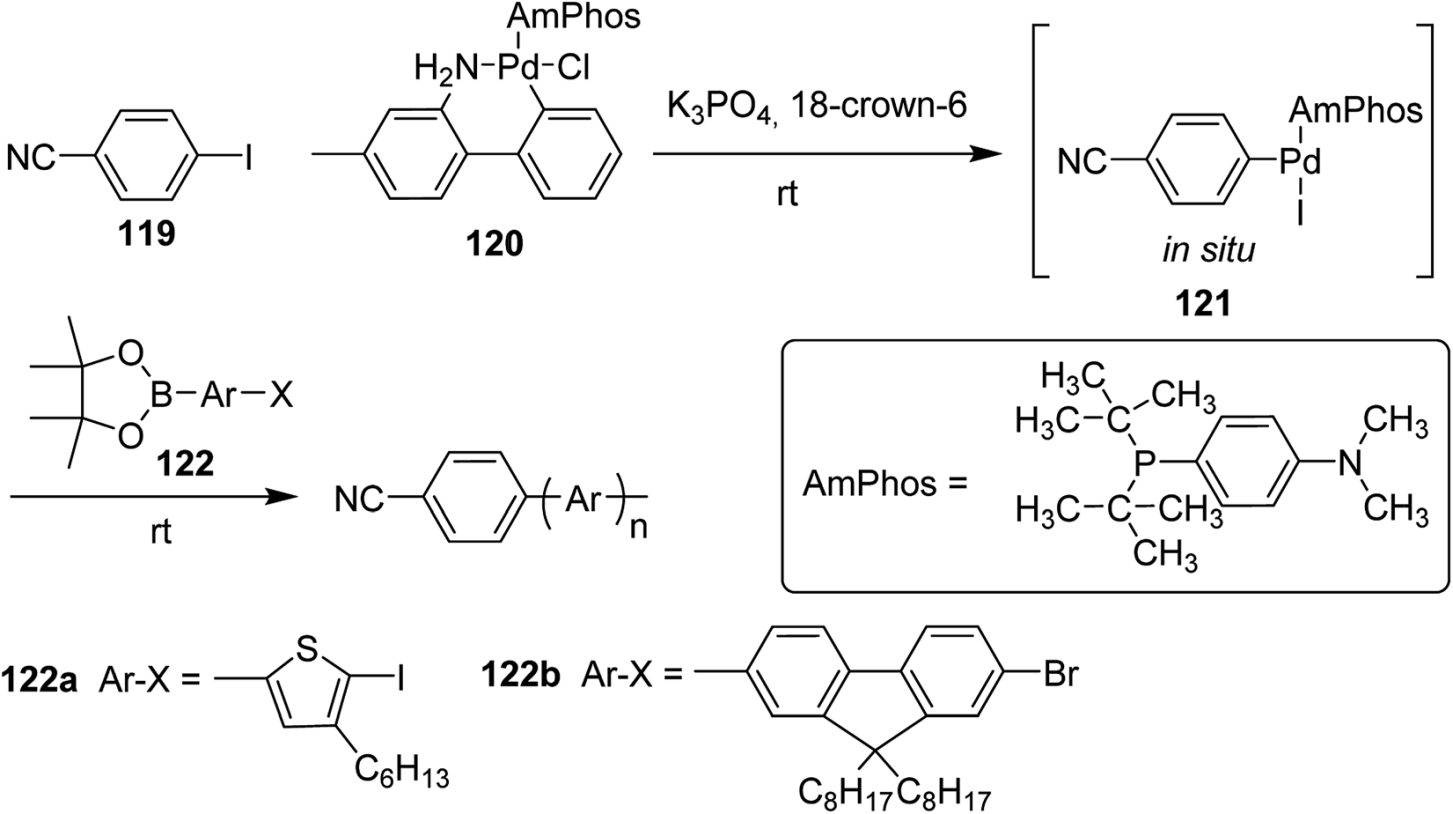
Polymerization of 122 with an initiator generated *in situ* from 120 and 119 at room temperature.

Polymerization of fluorene and thiophene with 4-iodobenzonitrile 119 using an *in situ* generated 4-cyanophenyl Pd (AmPhos) initiator 121 from AmPhos Pd precatalyst 120, proceeded through CTCP mechanism, yielding polythiophene 122a and polyfluorene 122b with controlled polymer ends and low dispersity (Scheme 38).

**Scheme 39:**
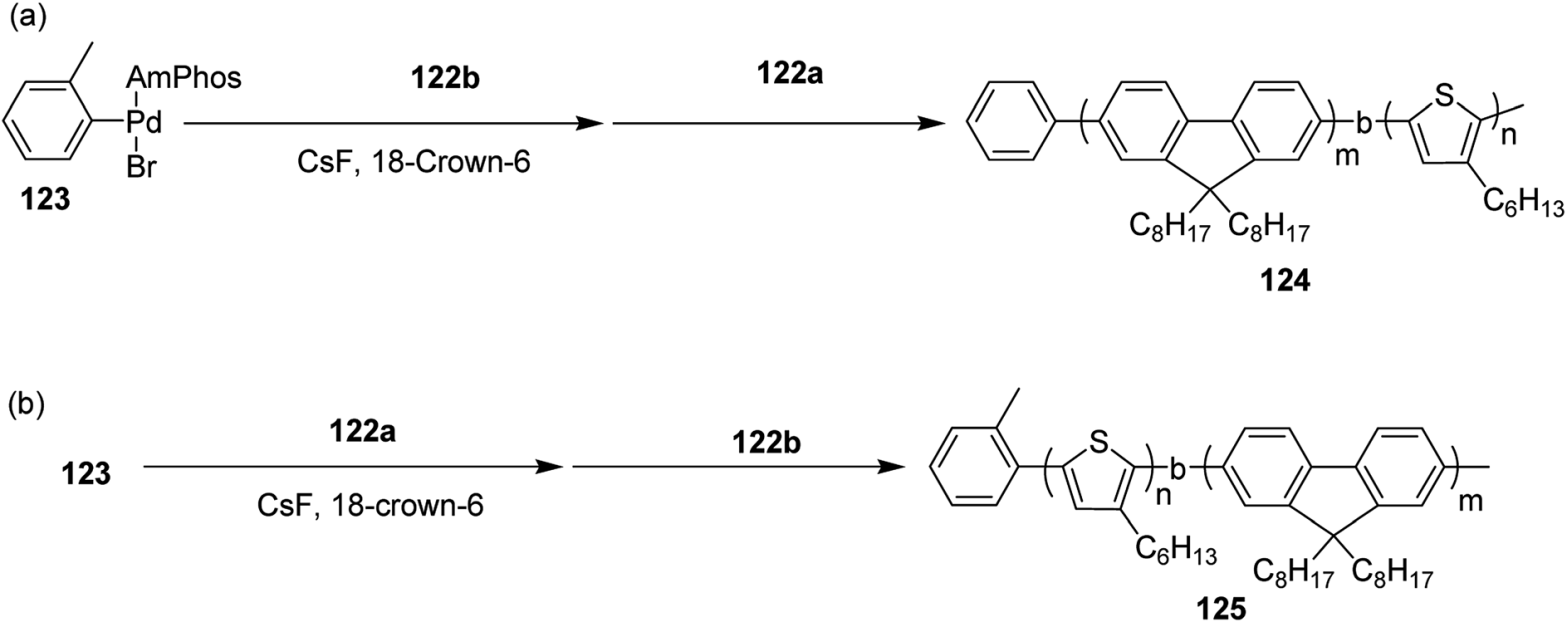
Block copolymerization of 122a and 122b with Pd initiator 121 at room temperature to synthesize copolymers 122 and 123.

**Scheme 40:**
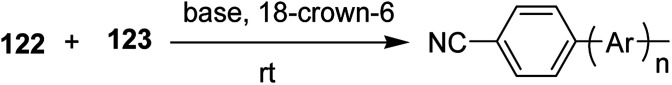
Polymerization of 122 with isolated AmPhos Pd Initiator 123 at room temperature.

Block copolymerization of fluorene and thiophene with 123 proceeded irrespective of the polymerization order. In block copolymerization, insensitivity of PdAmPhos to the polymerization order suggested possible synthesis of acceptor–donor–acceptor and donor–acceptor–donor triblock copolymers *via* Suzuki–Miyaura CTCP (Scheme 39). Moreover, (tolyl)PdAmphos (Br) 123 was also synthesized, and it was found that the mixture of 123 and cesium fluoride (CsF) yielded poly(3-hexylthiophene) with the dispersity index of 1.18 (Scheme 40). Table 8 summarizes yields and molecular weights of all the polymers obtained *via* polymerization of 122 with AmPhos Pd initiator 123.

**Table 8:** Polymerization of 122 with AmPhos Pd initiator 123^a^

Entry	122	[122]_0_/[123]_0_	Base	Yield^c^ (%)	*M*_n_ d	*M*_w_/*M*_n_^d^
1	122a	20	K_3_PO_4_	98	3330	1.61
2	122b	10	K_3_PO_4_	76	7970	1.50
3^b^	122a	20	K_3_PO_4_	95	5040	1.37
4^b^	122a	20	CsF	94	5700	1.18
5^b^	122a	12	CsF	68	3740	1.19
6^b^	122a	30	CsF	95	10 400	1.33
7^b^	120b	10	CsF	72	6300	1.22
8^b^	120b	20	CsF	85	15 300	1.43

a Polymerization of 122 with 123 ([122]_0_ = 8.33 × 10^−3^ M) in THF and water (water/THF (v/v) = 0.08) at rt.

b Catalyst 123 and base were stirred for 1 hour prior to polymerization.

c Isolated yield.

d Estimated by GPC based on polystyrene standards (eluent: THF).

### 4.2. Pd-catalyzed Stille-coupling polymerization

In 2012, Zhi-Guo Zang and co-workers reported the synthesis of two novel conjugated polythiophene derivatives PT4TV 133 (Scheme 39) containing thienylene–vinylene (TV) as a side chain and PT4TV-C 136 (Scheme 40) having thienylene–vinylene side chain attaching the carbonyl group *via* copolymerization of thiophene thienylene–vinylene side chain and unsubstituted terthiophene unit.^232^ Side chain isolation approach was employed to preserve backbone planarity, which combined asymmetric thiophene units with the conjugated side chains and unsubstituted trithienyl spacers in order to minimize the steric interactions between neighboring side chains. PT4TV and PT4TV-C displayed an increased π–π* transition absorption along with the steep absorption edge and shoulder peak indicating well-ordered polymer main chains and packed side chains compared to the previously reported conjugated side chain. Polythiophenes (CSC-PTs) with concentrated side chains.^233–239^ Introduction of electron-deficient carbonyl moiety in PT4TV-C resulted in a down-shifted homo energy level and a red-shifted absorption but aroused the problem of poorer planarity of the polymer backbone. PT4TV having more ordered in structure exhibited superior photovoltaic properties.

**Scheme 41:**
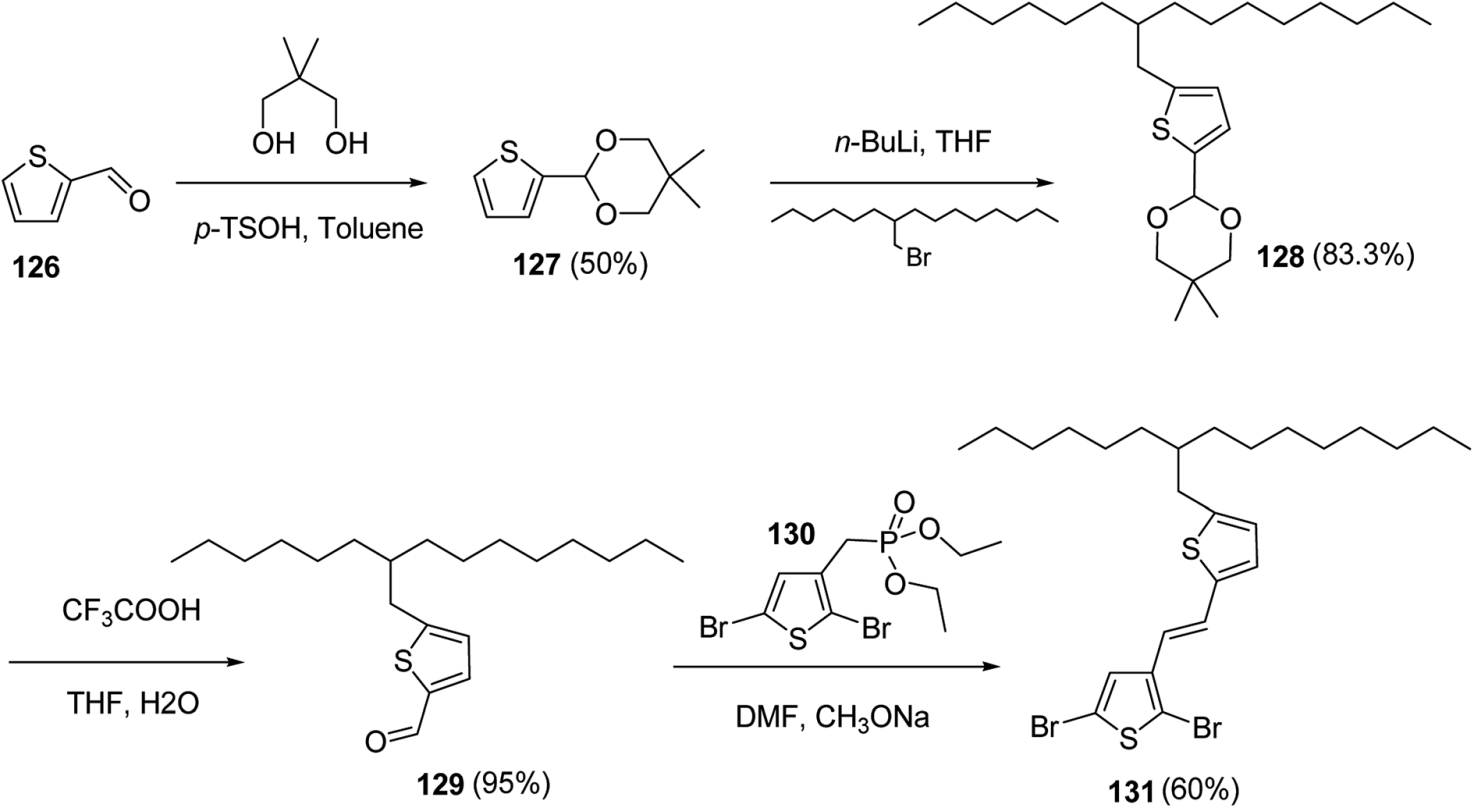
Synthetic route for the monomer 131.

**Scheme 42:**
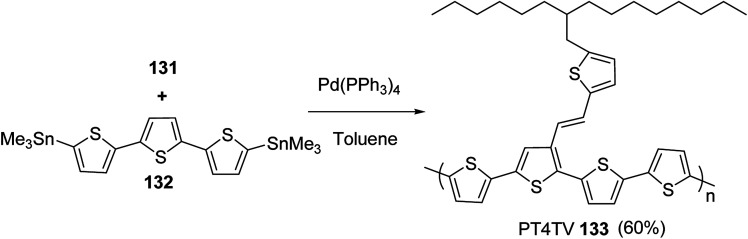
Synthesis of polymer 133.

**Scheme 43:**
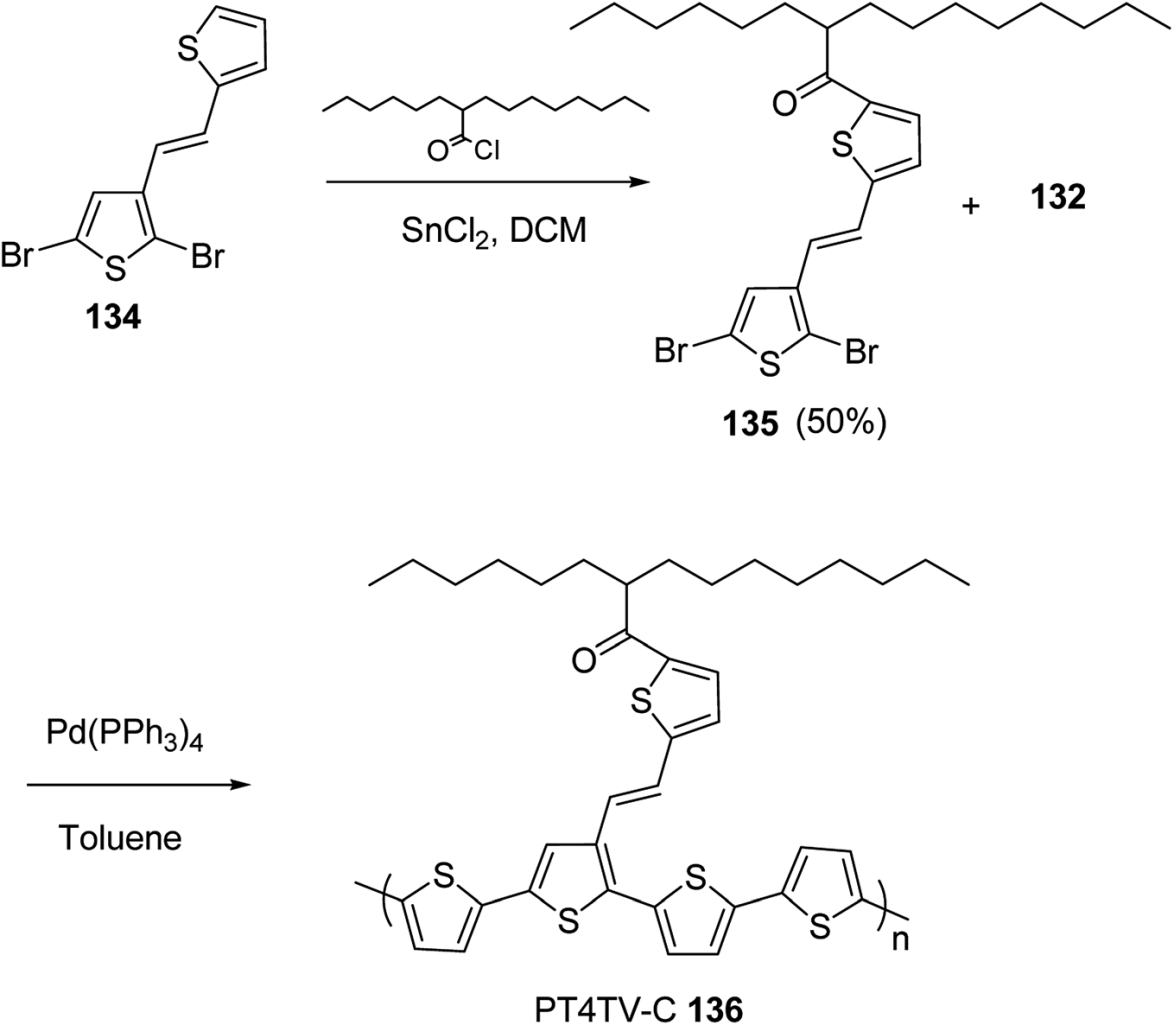
Synthesis of polymer 136.

Synthesis of PT4TV started with the protection of thiophene-2-carbaldehyde 126 by reacting it with 2,2-dimethyl-1,3-propandiol yielding acetal 127, which was alkylated by lithiation using *n*-butyllithium to give acetal derivative 128 after quenching with alkyl bromide. Dedioxanylation with trifluoroacetic acid was conducted to convert 128 into its corresponding aldehyde 129. Horner–Emmons–Wittig reaction was applied to couple carbaldehyde 129 with the phosphonate precursor 130 to afford dibrominated monomer 131. Acylated thiophene monomer 135 was synthesized by Friedel–Craft acylation of compound 133 in the presence of SnCl_2_ and 2-hexyldecanoyl chloride (Scheme 41). Stille coupling reaction of dibromo compounds 131 and 135 with distannylthiophene 132 resulted in the production of polymer PT4TV 133 and PT4TV-C 136, respectively. The two polymers were fairly soluble in chlorinated solvents for their application in polymer solar cells (Schemes 42 and 43).

Raja Shahid Ashraf and co-workers in 2014 reported the synthesis of diketopyrrolopyrrole-based copolymers consisting of different chalcogenophenes such as thiophene, tellurophene and selenophene for organic photovoltaic devices and field effect transistors.^240^ The polymer band gaps were reported to be narrowed by increasing the size of the chalcogen atom due to the LUMO energy level stabilization. Moreover, the larger intermolecular heteroatom–heteroatom interactions were also increased by increasing heteroatomic size, which led to an enhanced field effect mobilities of 1.6 cm^2^ (V s)^−1^ due to the formation of polymer aggregates. All these polymers exhibited high photoresponse in near-infrared region with tremendous photocurrents, making these polymers promising candidates for tandem solar cells.

**Scheme 44:**
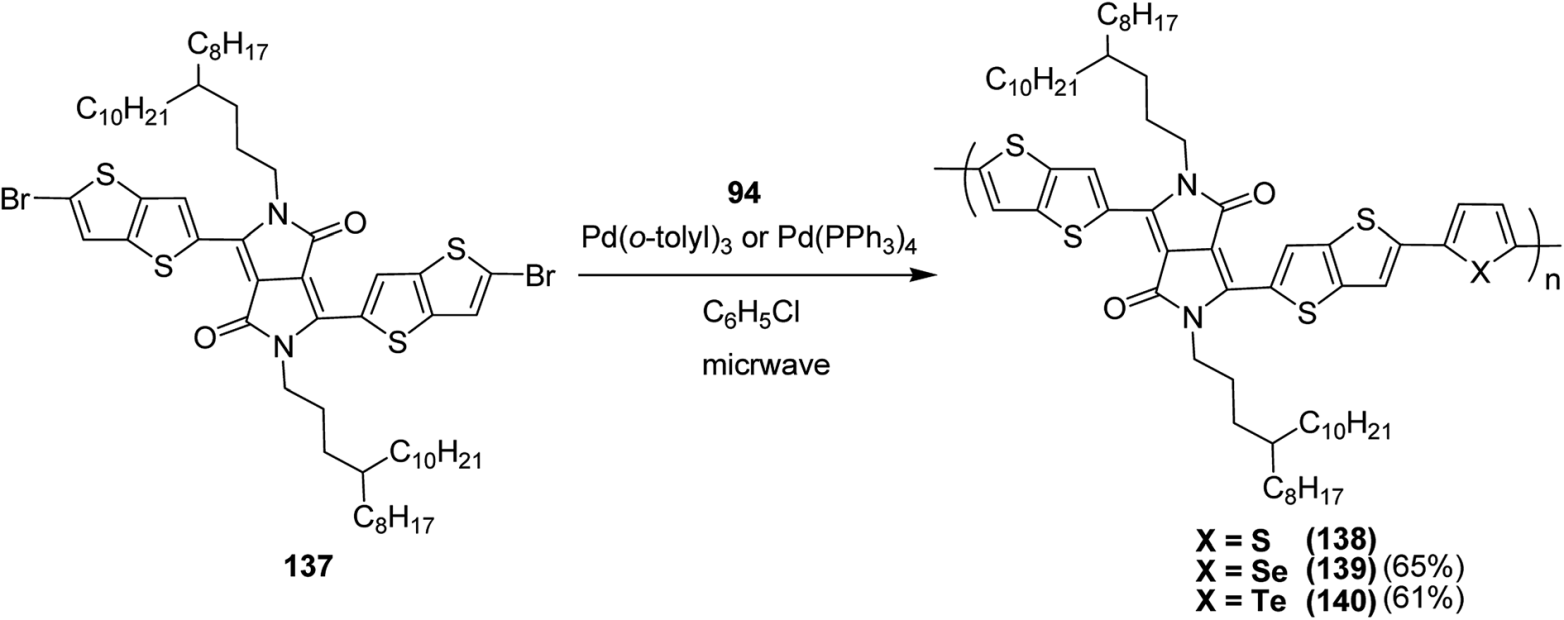
Synthesis of polymers 138–140 using Pd-catalyzed Stille coupling polymerization.

Pd-catalyzed Stille cross-coupling polymerization was used to copolymerize dibrominated C3-DPPT monomer with bis-stannylated thiophene, tellurophene and selenophene to afford C3-DPPT-T 138, C3-DPPT-Se 139 and C3-DPPT-Te 140, with the number average molecular weight of 80, 91 and 95, respectively, and relatively narrower dispersity of {*Đ* = (T = 1.9), (Te = 3.0) and (Se = 2.5)} (Scheme 44). The two newly synthesized polymers, C3-DPPT-Se 139 and C3-DPPT-Te 140, were compared experimentally and computationally with the previously reported C3-DPPT-T copolymer 138. Crystallinity of the neat polymer films were shown to be greatly influenced by the size of the chalcogen atom. Reduction in the aromaticity caused a slight decrease in the *E*_LUMO_ and increase in the *E*_HOMO_ values with an increase in the chalcogen size, resulting in decreased optical band gaps and a red shift in the UV-vis absorption.

In 2014, Shaoqing Zhang and co-workers reported the synthesis and characterization of two new benzodithiophene (BDT)-based polymers, PBDTTT-EFF 146a and PBDTTT-EFS 146b bearing selenophene and furan as side chains with already reported PBDTTT-EFT 146c polymer having thiophene as a side group.^241^ It was shown that HOMO levels, absorption bands, aggregation sizes and crystallinities of these polymers were affected by the dihedral angle between benzodithiophene units and conjugated side groups. PBDTTT-EFT 146c and PBDTTT-EFS 146b showed comparable photovoltaic characteristics in device, and PCEs of 8.78% and 9.0% were obtained, relatively. The device containing PBDTTT-EFF 146a showed *J*_sc_ of 11.77 mA cm^−2^ and an open circuit voltage (*V*_oc_) of 0.69 V, which are lower than those in the devices based on the other two polymers. Over all this work suggested that the photovoltaic properties of benzodithiophene-based polymers could be tuned by introduction of conjugated side groups having different steric hindrance.

**Scheme 45:**
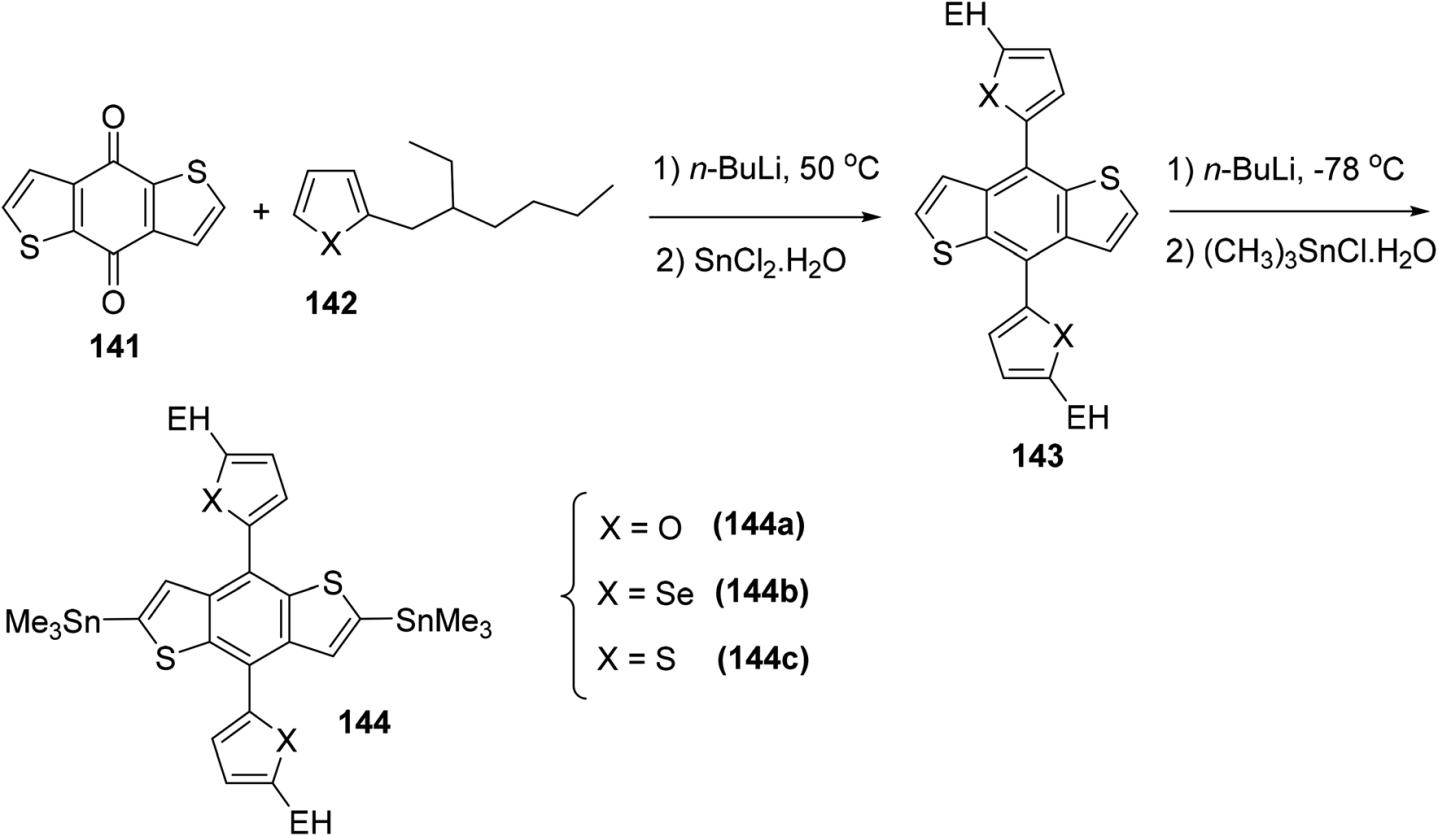
Synthesis and molecular structures of the monomers 144a–c.

**Scheme 46:**
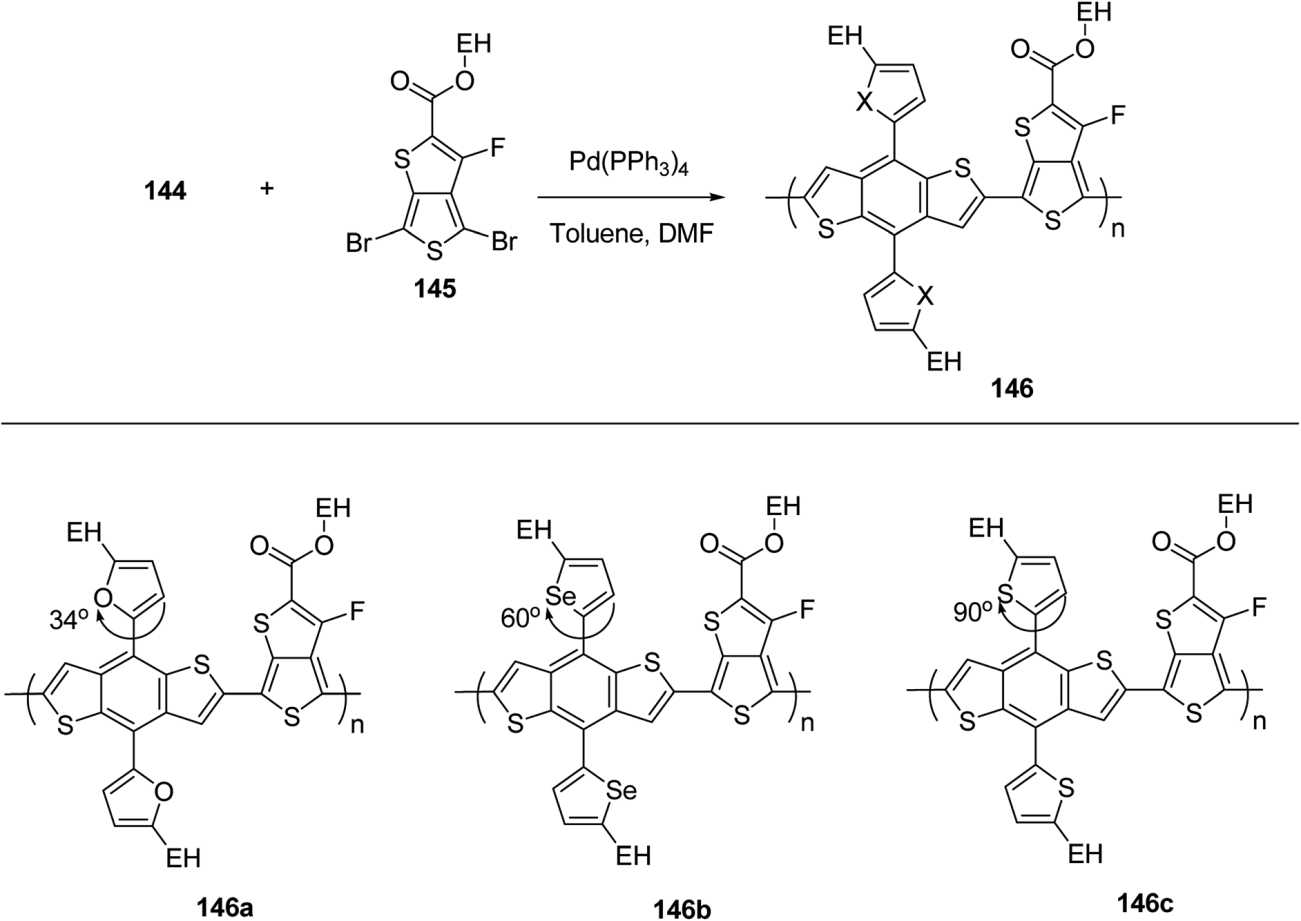
Synthesis and molecular structures of the polymers 146a–c.

4,8-Dihydrobenzo[1,2-*b*:4,5-*b*′]dithiophen-4,8-dione 141 was reacted with compound 142 to yield BDT core 143 which was stannylated to obtain final BDT-based monomers BDT-F 144a, BDT-T 144b and BDT-S 144c (Scheme 45). Benzodithiophene (BDT) monomers with 2-alkylselenophenyl, 2-alkylthienyl and 2-alkylfuryl side groups were polymerized with thieno[3,4-*b*]thiophene (TT) unit 143, using Stille coupling polycondensation to synthesize PBDTTT-EFF 146a, PBDTTT-EFS 146b and PBDTTT-EFT 146c. All these polymers showed similar thermal stabilities and decomposition onset points at *ca.* 350 °C (Scheme 46).

In 2014, Iain Meager and co-workers reported the synthesis of a new thieno[3,2-*b*]thiophene isoindigo (iITT) based monomer unit 151, and its subsequent polymerization with thiophene, bithiophene and benzodithiazole to furnish three new copolymers with narrow band gap semiconducting properties for OFET applications.^242^ It was found that extension of the fused ring system attached to the isoindigo core could serve to further enhance molecular orbital overlap along the polymer backbone and, hence, facilitate good charge transport characteristics. All the three newly synthesized polymers showed good ambipolar properties when used as a semiconducting channel in top-gate/bottom-contact OFET devices as well as good stability with high temperature annealing, exhibiting an increase in the crystallinity of the polymers which directly corresponds to the improvement in the charge carrier mobility.

**Scheme 47:**
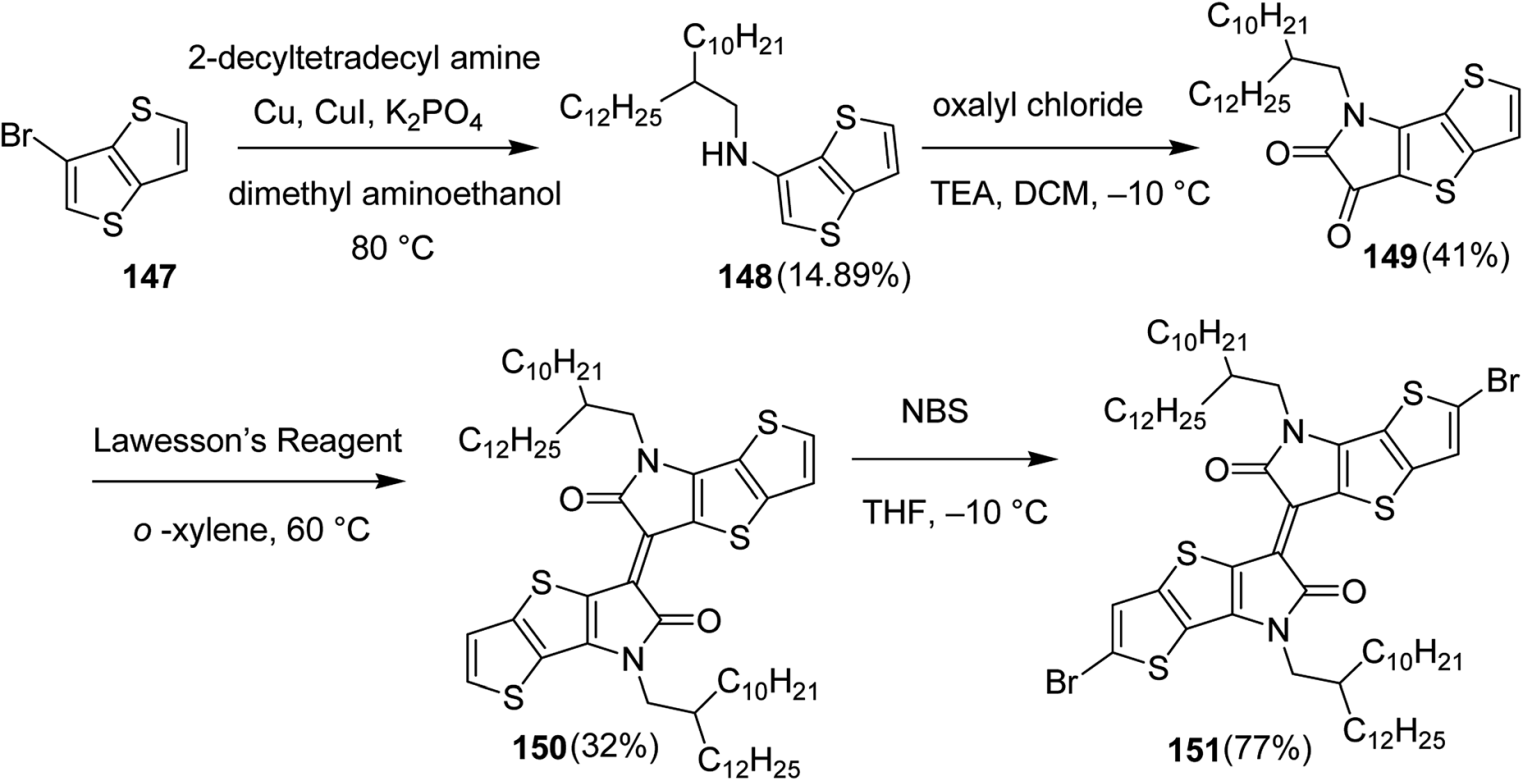
Synthesis of new dibrominated iITT monomer unit 151.

Synthesis of the ilTT monomer is a first example of conjugated six fused ring isoindigo system. 3-Bromothieno[3,2-*b*]thiophene 147, synthesized from thieno[3,2-*b*]thiophene, applying Cu-catalyzed Ullmann coupling reaction, was used to introduce branched 2-decyltetradecyl amine at its position-3 to yield 148. Amine functionality was reacted with oxalyl chloride to form pyrrole dicarbonyl ring at the 2 position of the thieno[3,2-*b*]thiophene to yield 149. ilTT core was obtained by dimerization of dicarbonyl unit in the presence of Lawesson's reagent^243^ to give 150, and its subsequent treatment with bromine yielded the final monomer 151 (Scheme 47).

**Scheme 48:**
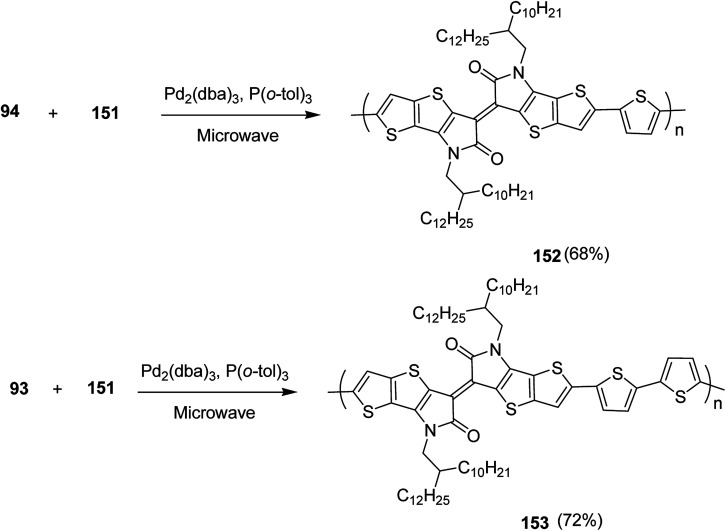
Microwave assisted copolymerisation of dibrominated iITT monomer 151 with distannylated thiophene 94 and bithiophene 93 to afford polymer 152 and 153.

**Scheme 49:**
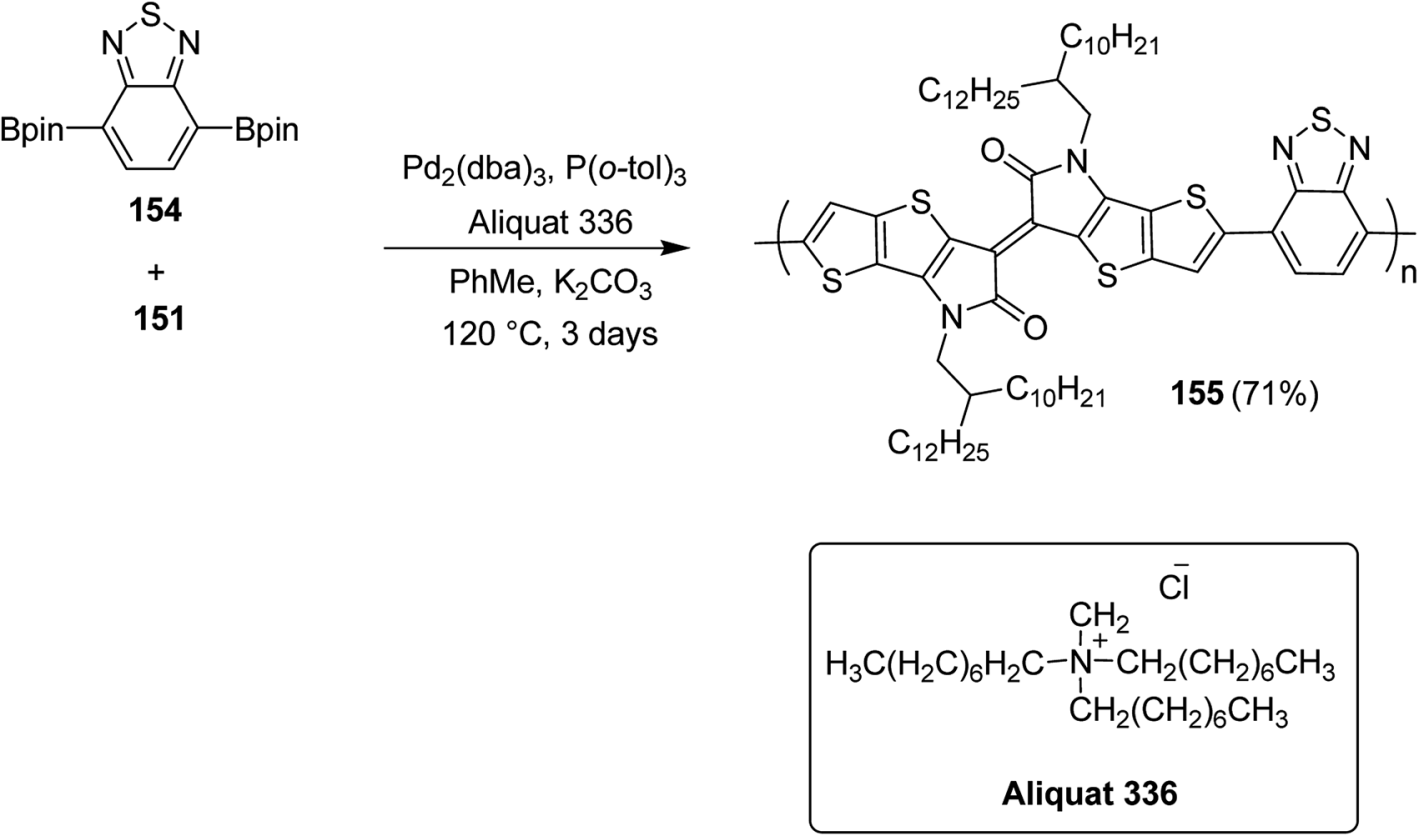
Copolymerisation of a dibrominated iITT monomer with benzothiadiazole 154 to afford polymer 155.

All the three polymers 152, 153 (Scheme 48) and 155 (Scheme 49) were synthesized by Pd-catalyzed cross-coupling reaction. Copolymers of thiophene and bithiophene were synthesized using Stille coupling polymerization, whilst Suzuki coupling method was used to polymerize ilTT with benzothiadiazole. Each polymer was obtained in comparable weight average molecular weight (*M*_w_) between 17–30 kDa with low polydispersity indexes (PDI) around 2.

In 2014, Ming Wang and co-workers reported the synthesis of regioregular polymer 160 incorporating two different donor units, indacenodithiophene (IDT) 156 and cyclopentadithiophene (CPDT) 159, and pyridyl[2,1,3]thiadiazole (PT) 157 acceptor unit in the backbone.^244^ In polymer 160 backbone, PT 157 orientations were strictly organized with pyridyl N-atoms pointing toward CPDT fragments. 160 was compared with its regiorandom counterpart PICP-RA, which displayed a higher level of molecular order, resulting in higher power conversion efficiencies in bulk heterojunction (BHJ) organic solar cells. PICP:PC_61_BM blends yielded devices having an open circuit voltage of 0.86 V, while maintaining power conversion efficiency of approximately 6%. This *V*_oc_ value is particularly high for bulk heterojunction system as compared to the analogous narrow band gap conjugated polymers, indicting a very low *E*_g_–*V*_oc_ loss.

**Scheme 50:**
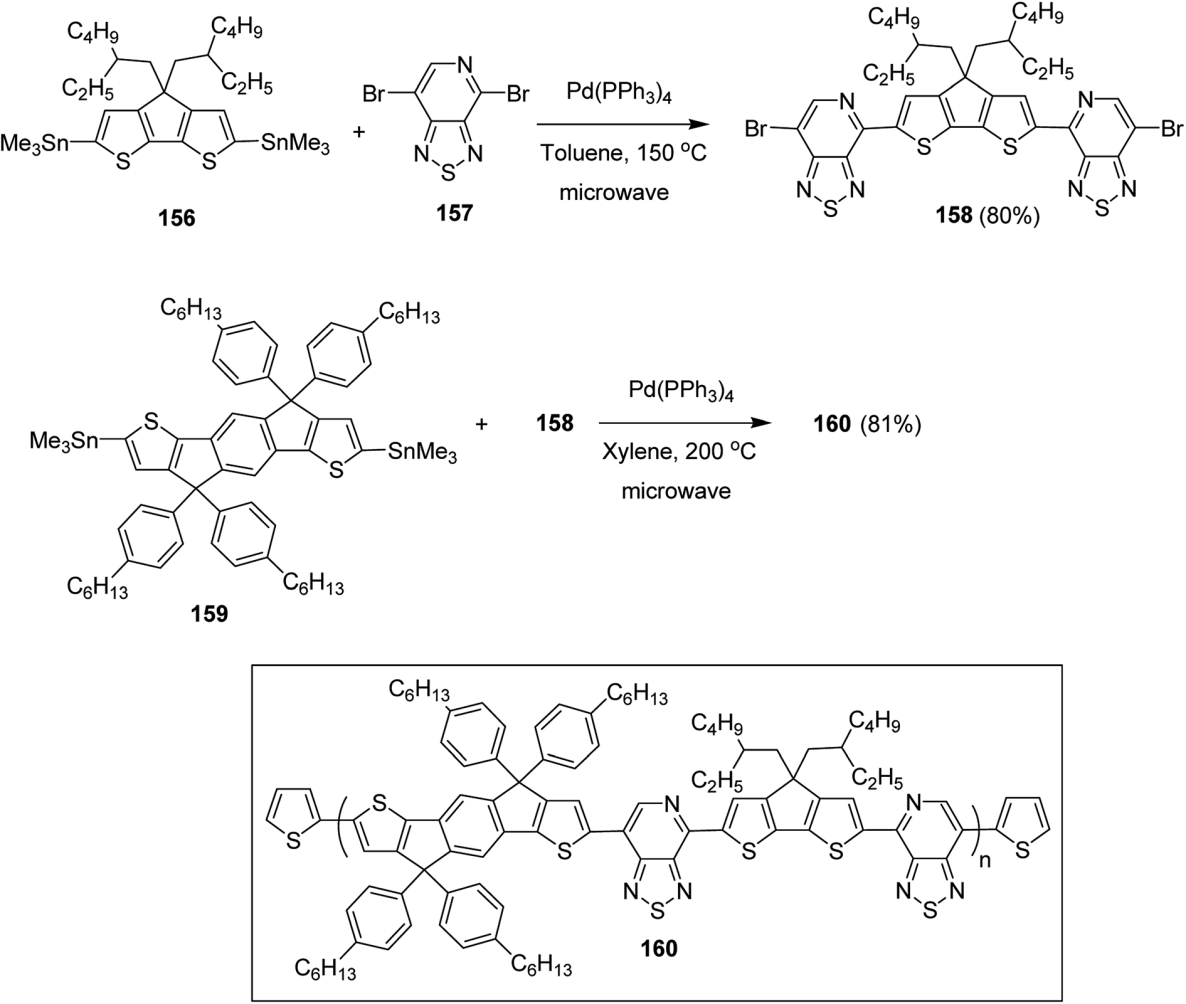
Synthesis and chemical structure of polymer 160.

In order to achieve regioregularity in the polymer 160, the monomers 156 and 157 were coupled *via* Stille cross-coupling reaction to afford a symmetrical monomeric unit 158 with 80% yield and a regioregularity over 99%. Desired polymer 160 was synthesized by polymerizing 158 and 159 under microwave heating at 200 °C using Pd (PPh_3_)_4_ catalyst (Scheme 50).

Benedetta M. Squeo and co-workers in 2015 reported the synthesis of new ultra low band gap (LBG) polymers based on α,β-unsubstituted BODIPY for the first time *via* Stille cross-coupling polymerization.^245^ 4,4-Difuoro-4-bora-3*a*,4*a*-diaza-*s*-indacene 165, commonly known as BODIPY is one of the less explored electron-deficient monomers for the synthesis of near infrared (NIR) conjugated polymers. Dibromo BODIPY monomer was successfully polymerized with (*E*)-1,2-bis(3-dodecyl-5-(trimethylstannyl)thiophen-2-yl)ethane 166 as a comonomer yielding a new ultra low band gap copolymer, TBDPTV 167, which displayed a panchromatic absorption spectrum ranging from 300 nm to 1100 nm, and an optical band gap (*E*^opt^_g_) of 1.15 eV, suitable for near infrared (NIR) organic photovoltaic applications as electron donor.

**Scheme 51:**
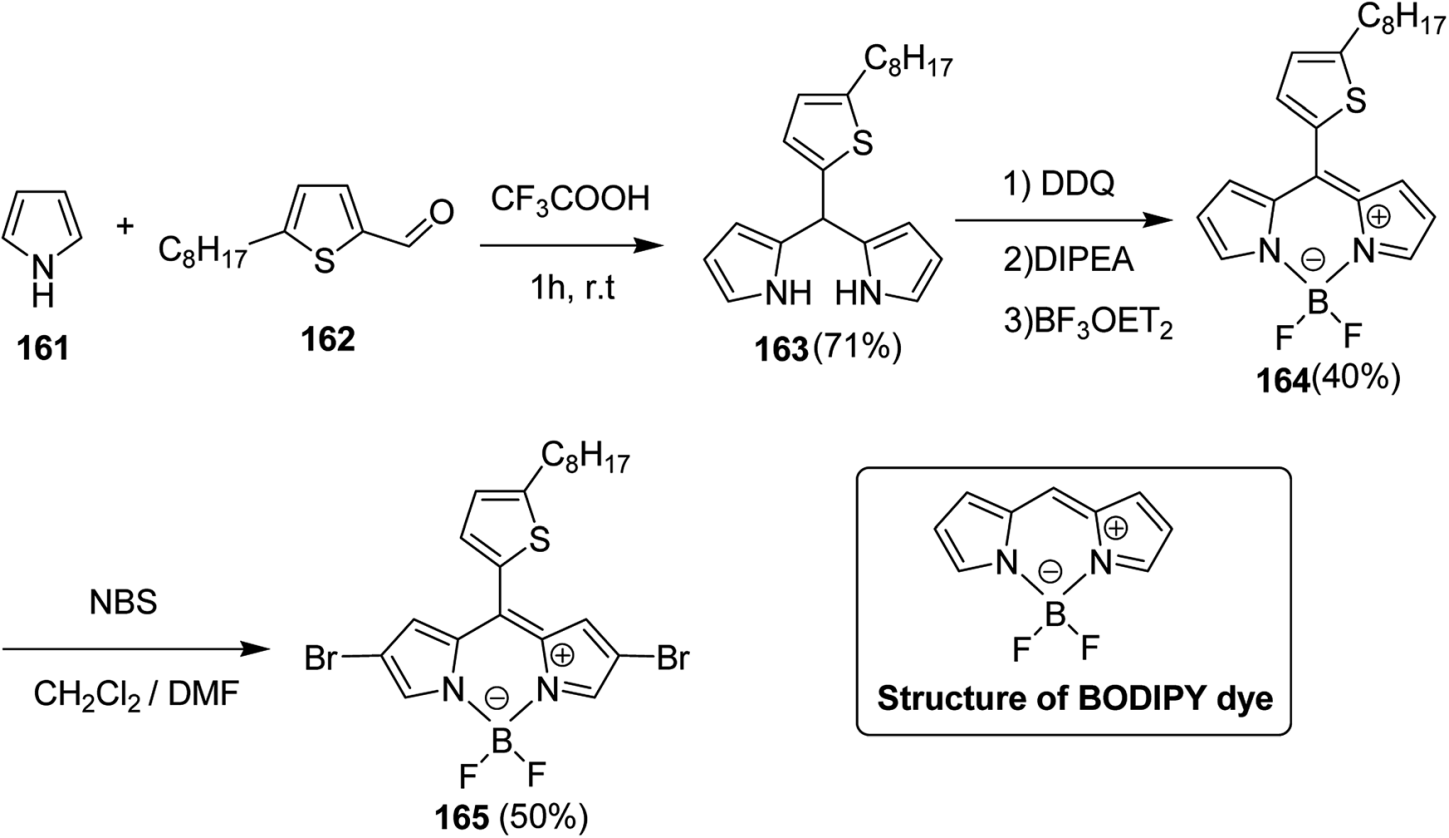
Synthesis of dibromo borondipyrromethene 165.

BODIPY dye was synthesized under neat conditions by dissolving 5-octylthiophene-2-carbaldehyde 162 into the excess of pyrrole 161 to afford 2,2′-[(5-octylthiophen-2-yl)methylene]bis(1*H*-pyrrole) 163. Monomer 163 was treated with DDQ, then, with diisopropylethylamine and finally with trifluoroborane dietherate [BF_3_O(ET)_2_] to yield corresponding borondipyrromethene 164. Dibrominated borondipyrromethene 165 was synthesized by using NBS in DMF and DCM as a 1 : 1 solvent mixture (Scheme 51).

**Scheme 52:**
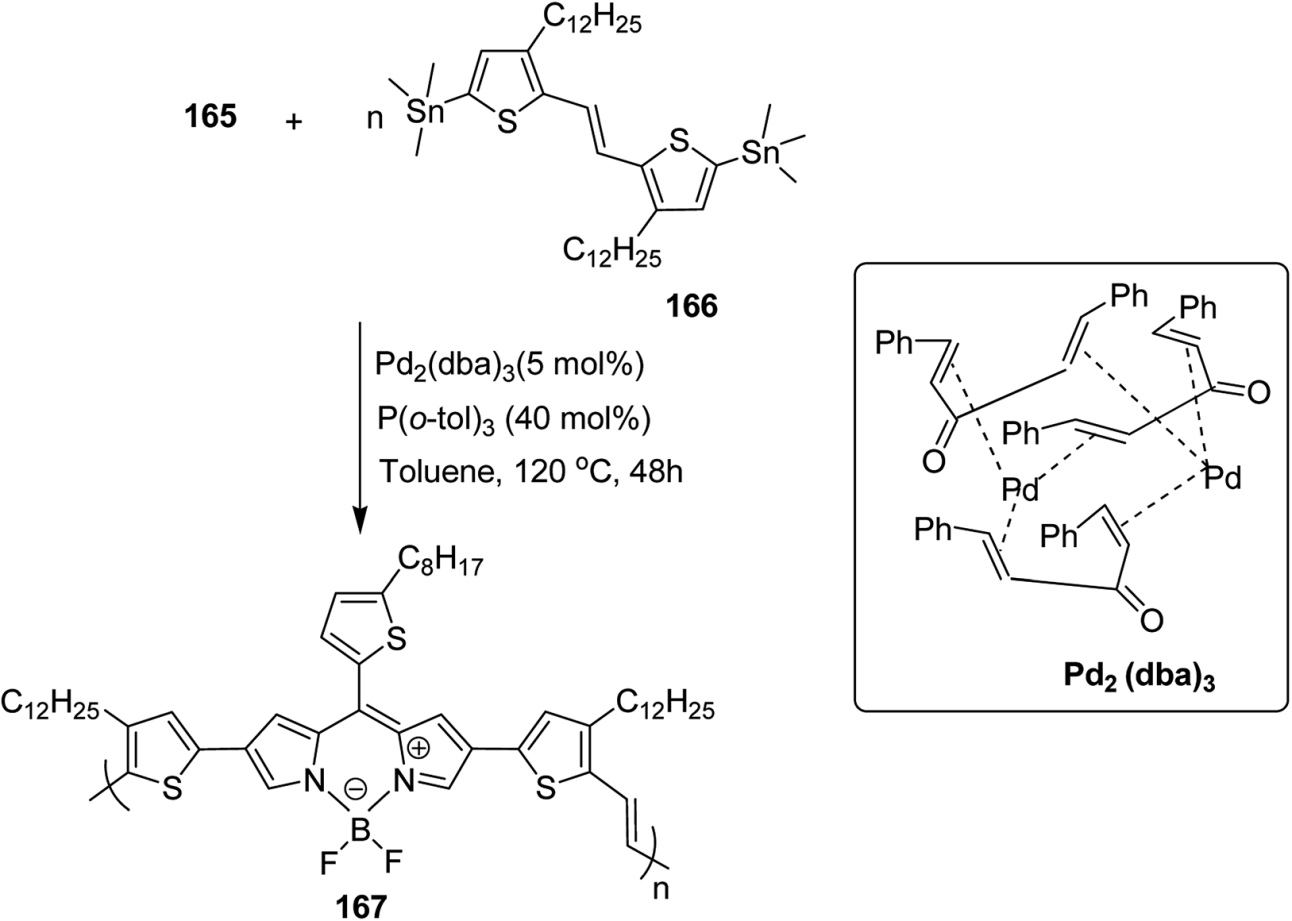
Synthesis of 167 by Stille cross coupling polymerization.

Stille cross-coupling polymerization was applied to synthesize 167, using 1 : 1 monomer feed ratios. A solution of monomer 165 and commercially available (*E*)-1,2-bi[3-dodecyl-5-(trimethylstannyl)thiophene-2-yl]ethane 166 were combined in a dry deoxygenated toluene in the presence of Pd_2_(dba)_3_ and [P(*o*-tolyl)_3_]. The resulting reaction mixture was heated at 120 °C for 48 hours to afford the desired polymer 167 after purifying the crude product by Soxhlet extraction (Scheme 52).

**Scheme 53:**
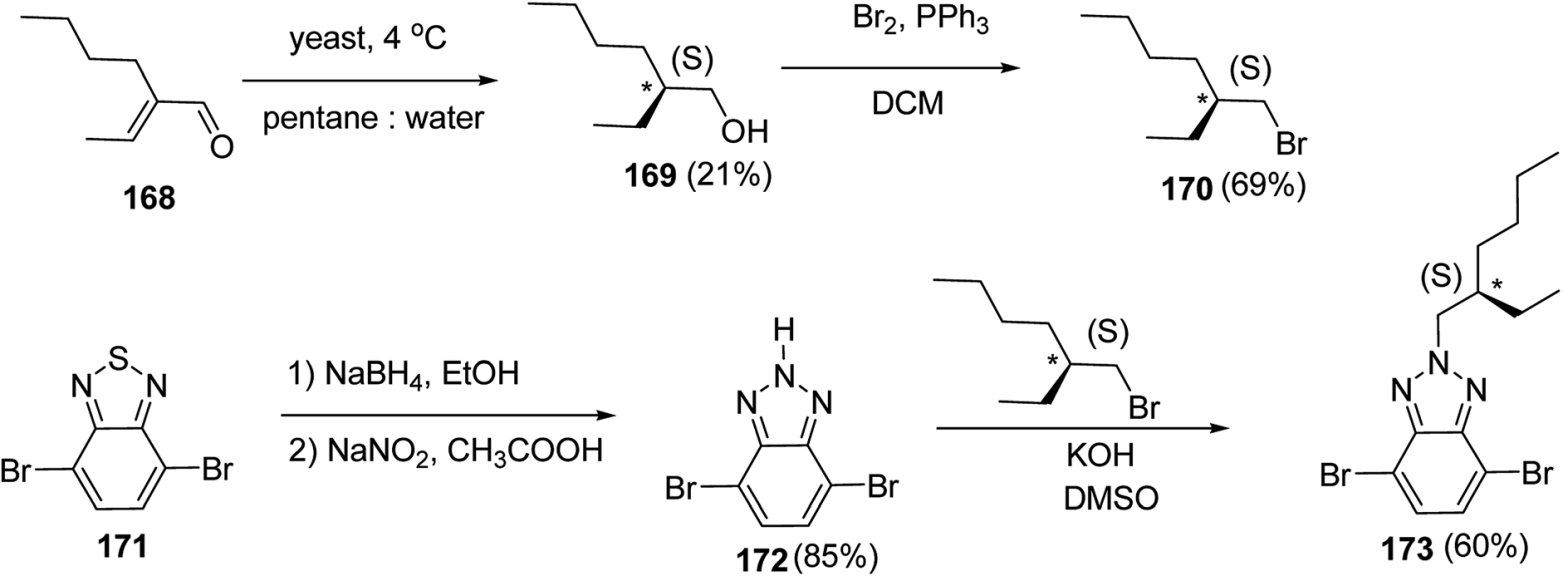
Synthesis of the monomer 173.

In 2016, Stephanie L. Fronk reported the synthesis and characterization of the conjugated polymer PBTz-Th* 174, consisting of benzotriazole and thiophene moieties bearing a chiral (*S*)-2-ethylhexyl chain on the triazzole unit.^246^ A reference polymer, PBTz-Th, having racemic 2-ethylhexyl chain was also synthesized for the comparison of chiroptical properties. Both polymers showed chain aggregation even at dilute concentrations. PBTz-Th* 174 was also characterized *via* CD (circular dichroism) spectroscopy due to the presence of a chiral side chain, while PBTz-Th did not show any response to CD. CD spectra revealed that the chains of 174 are chiral in aggregate, and this chiral ordering was also found to translate from the aggregates in the solution to solid state upon deposition of the solution, due to the relatively small calculated barrier to rotation of the BTz-Th unit.

(*S*)-2-Ethylhexan-1-ol 169 was synthesized by reduction of α,β-unsaturated aldehyde 168 using Baker's yeast as a catalyst. (*S*)-2-Ethylhexan-1-ol was reacted with bromine and triphenylphosphine in DCM to afford (*S*)-3-(bromomethyl)heptane 170. Benzothiadiazole 171 was treated with sodium borohydride and sodium nitrite to afford 172. A chiral side chain was attached to the benzotriazole framework 172 using a reported alkylation technique to yield 173 (Scheme 53).

**Scheme 54:**
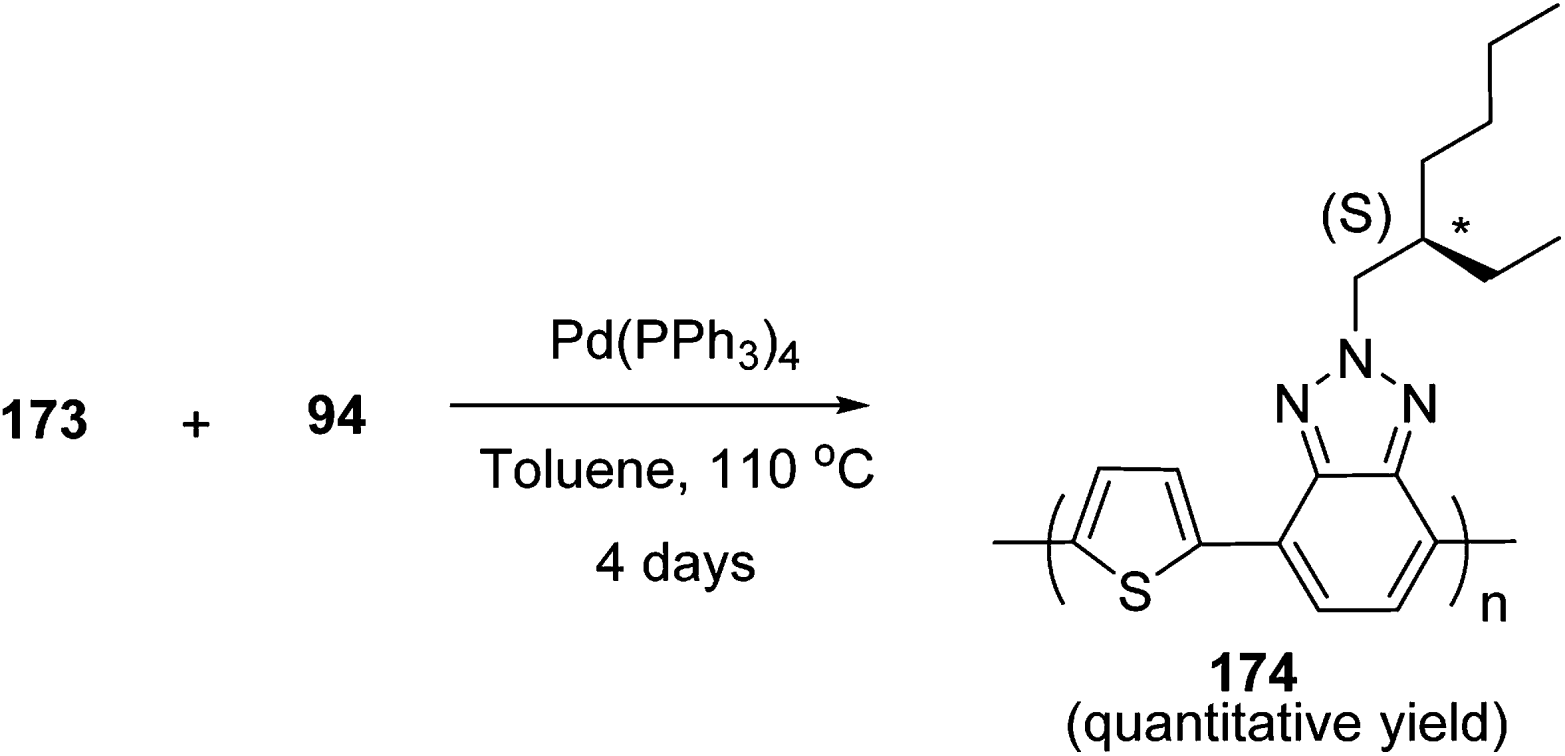
Synthesis of the polymer 174.

EH-S-BTz 173 was polymerized with bis(Me_3_Sn)thiophene 94 using conventional Stille coupling polymerization reaction affording the desired polymer 174 with number average molecular weight (*M*_n_) of 6.2k, and dispersity index (*Đ*) procedure was used to synthesize a reference polymer (PBTz-Th), having racemic 2-ethylhexyl chain, with the same molecular weight and dispersity index (Scheme 54).

Stephnie L. Fronk and co-workers in 2016 reported the synthesis of two new conjugated polymers, poly[(4,4-bis(2-ethylhexyl)cyclopenta-[2,1-*b*:3,4-*b*′]dithiophene)-2,6-diyl-*alt*-[2,1,3-benzothiadiazole-4,7-diyl] (PCPDTBT*) 180 and poly[(4,4-bis(2-ethylhexyl)cyclopenta-[2,1-*b*:3,4-*b*0]dithiophene)-2,6-diyl-*alt*-[1,2,5]-thiadiazolo[3,4-*c*]pyridine] (PCDTPT*) 183 with chiral 2-ethylhexyl side chains to investigate the effect of chiral substituent on the chiroptical properties of these polymers.^247^ Secondary structure and geometry of interchian aggregates of these polymers were studied *via* circular dichroism spectroscopy, which revealed the presence of chiral ordering in aggregates. For PCPDTBT* 180, chiral ordering was found to translate to the solid-state microstructures upon solution deposition, but not for the PCDTPT* 183. In 183, planar backbone conformation is favored due to the presence of the pyridyl nitrogen on thiadiazole[3,4-*c*]pyridine ring, presenting higher rotational barrier compared to 180. This higher rotational barrier seemed to limit the ability of 183 to achieve chiral aggregates by adopting helical structure.

**Scheme 55:**
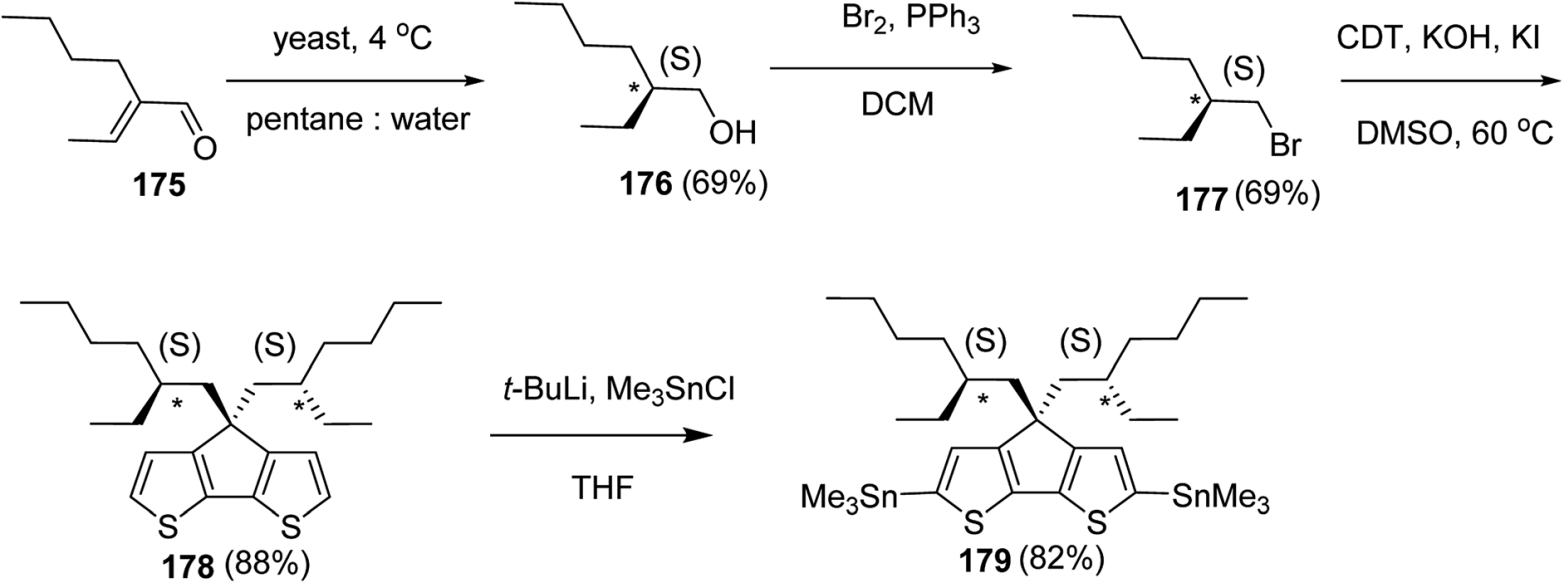
Synthesis of the monomer 179.

**Scheme 56:**
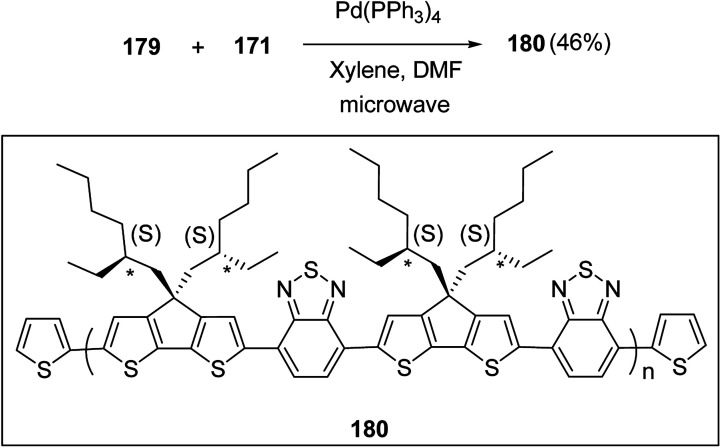
Synthesis of the polymer 180.

Baker's yeast was used as biocatalyst to reduce α,β-unsaturated aldehyde 175 to chiral alcohol (*S*)-2-ethylhexan-1-ol 176 with a 95% enantiomeric excess based upon the comparison with known optical rotation. This chiral alcohol was reacted with 1.4 equivalent of triphenylphosphine and 2.7 equivalent of bromine to afford (*S*)-3-(bromomethyl)heptane 177. 2-Ethylhexyl side chain was introduced to the cyclopentadithiophene (CDT) unit by dissolving CDT, potassium hydroxide and potassium iodide in DMSO and then adding (*S*)-3-(bromomethyl)heptanes *via* syringe and heating the reaction mixture at 60 °C for 16 hours to yield 178. Bis stannylated intermediate EH-S-CDTSn_2_179 was obtained through the reaction of EHS-CDT 178 with 3 equivalent of Me_3_SnCl and 2.5 equivalents of *t*-BuLi (Scheme 55). EHS-CDTSn_2_179 was polymerized with 4,7-dibromo-2,1,3-benzothiadiazole (BT-Br_2_) 170, applying microwave assisted Stille coupling polycondensation using Pd(PPh_3_)_4_ to afford polymer 180 with a *M*_n_ of 23k and *Đ* of 1.6 (Scheme 56).

**Scheme 57:**
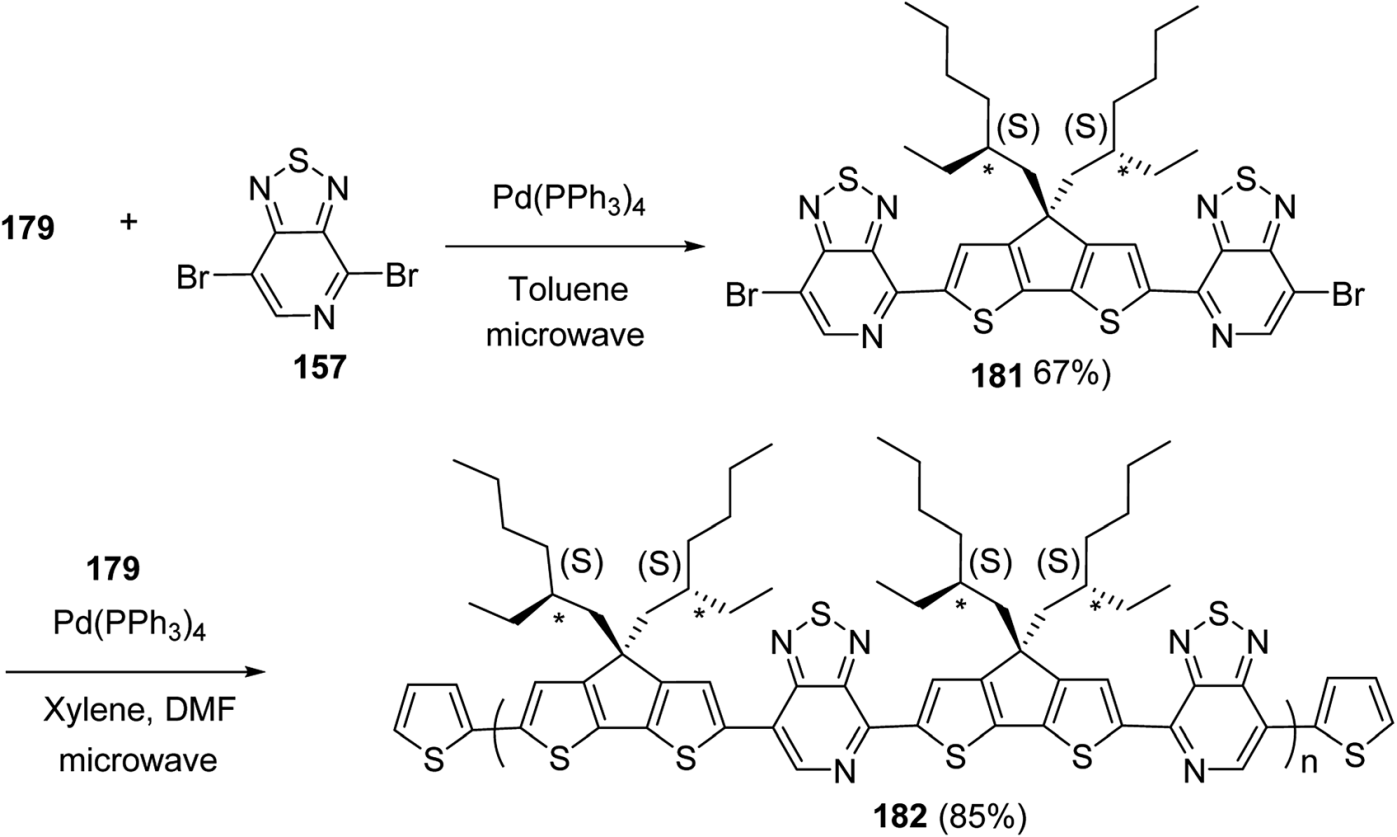
Synthetic route to the polymer 182.

PT-EH-S-CDT-PT 181 was synthesized *via* regiospecific Stille coupling reaction between PT-Br_2_157 and EH-S-CDTSn_2_179. The target polymer PCPDTBT* 182 was obtained *via* Stille coupling polymerization of EH-S-CDTSn_2_179 and PT-EH-S-CDT-PT 181 with a *M*_n_ of 17k and *Đ* of 1.7 (Scheme 57).

**Scheme 58:**
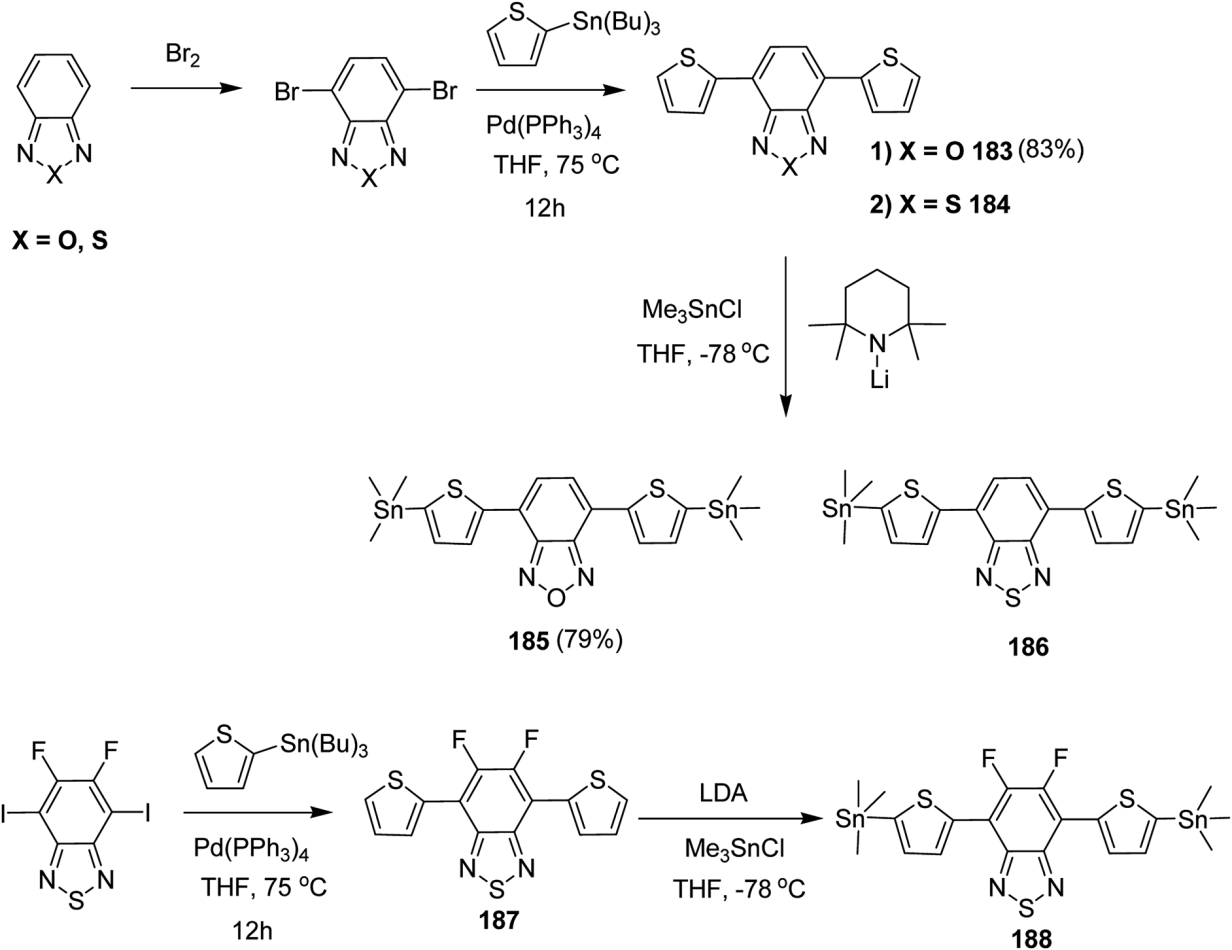
Synthesis of the monomers 185, 186 and 188.

**Scheme 59:**
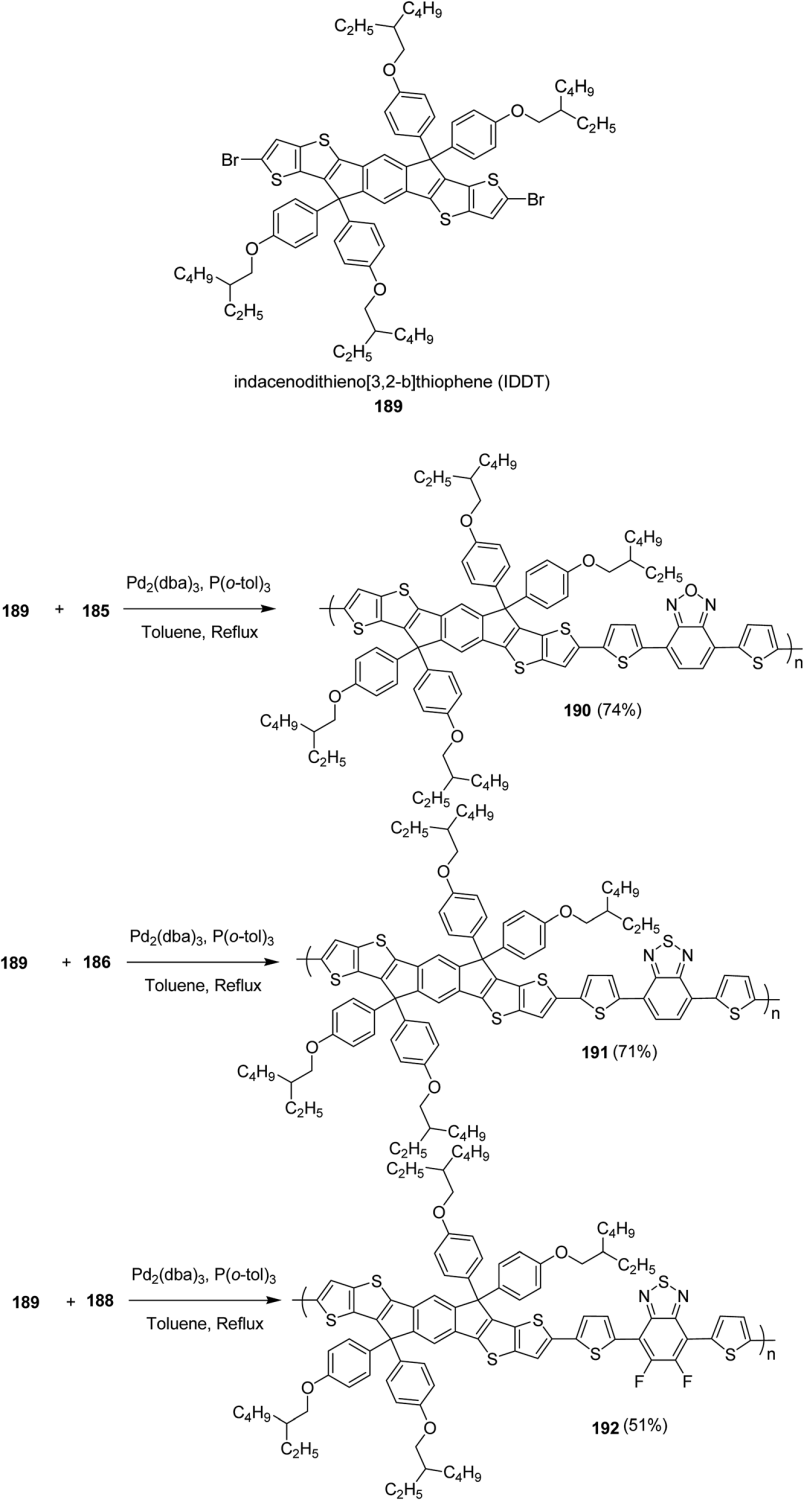
Pd-Catalyzed synthesis of IDDT-based polymers 190–192.

Ping Cai and co-workers in 2017 reported the synthesis of three novel IDTT based donor–acceptor polymers, PIDTT-DTBO 190, PIDTT-DTBT 191 and PIDTT-DTFBT 192, employing indacenodithieno[3,2-*b*]thiophene (IDTT) 189 as an electron-rich unit and benzodiathiazole (BT), difluorobenzothiadiazole (FBT) or benzoxadiazole (BO) as an electron-deficient unit, through Pd-catalyzed Stille polymerization.^248^ Electron-withdrawing atoms of the acceptor portion were varied as S, F and O for tailoring the electrochemical and optical properties as well as geometry of the structures. A range of techniques was used to investigate their effects on the film topography and photovoltaic and hole-transporting properties of the polymers 190–192. 4,7-Di(thiophen-2-yl)benzo[*c*][1,2,5]oxadiazole 183, 4,7-di(thiophen-2-yl)benzo[*c*][1,2,5]thiadiazole 184 and 5,6-difluoro-4,7-di(thiophen-2-yl)benzo[*c*][1,2,5]thiadiazole 187 were stannylated to obtain the monomers 185, 186 and 188, respectively. The weight average molecular weight (*M*_w_) of polymer PIDTT-DTBO 190 and PIDTT-DTBT 191 was 30.5 and 33.3 kDa with polydispersity index (PDI) of 2.2 and 2.4, respectively. For polymer PIDTT-DTFBT 192, a rather lower *M*_n_ of 8.3 kDa and board PDI up to 3.6 were obtained (Schemes 58 and 59).

Organic semiconducting materials based on 1,3-butadiyne unit were reported for the first time in 2017 by Brian J. Eckstein and co-workers.^249^ Alkyl-substituted 1,4-di(thiophen-2-yl)buta-1,3-diyne (R-DTB) donor building blocks were polymerized with thienyldiketopyrrolopyrrole (R′-TDPP) acceptor units to afford π-conjugated polymers TDPP-DTB, in which R and R′ groups were varied to obtain four different polymers (199–202). The solubility of the newly synthesized polymers was revealed to be strongly dependent upon the substitution pattern of the DTP and DPP monomer units. Optoelectronic devices (OFETs, OPVs) were fabricated with 1,3-butadiyne containing organic semiconductors for the very first time. DTB-based polymers 201 (R = *n*-C_12_H_25_, R′ = 2-butyloctyl) and 202 (R = 2-ethylhexyl, R′ = 2-butyloctyl) in addition to sp-hybridized carbon–carbon triple bond and with relief of steric torsion between in-chain π-blocks due to the planarization of the conjugated backbone and stabilization of frontier molecular orbitals, displayed discrete morphological pliability through thermal annealing and processing additives. Polymers, 199 and 200 were not solution processable.

**Scheme 60:**
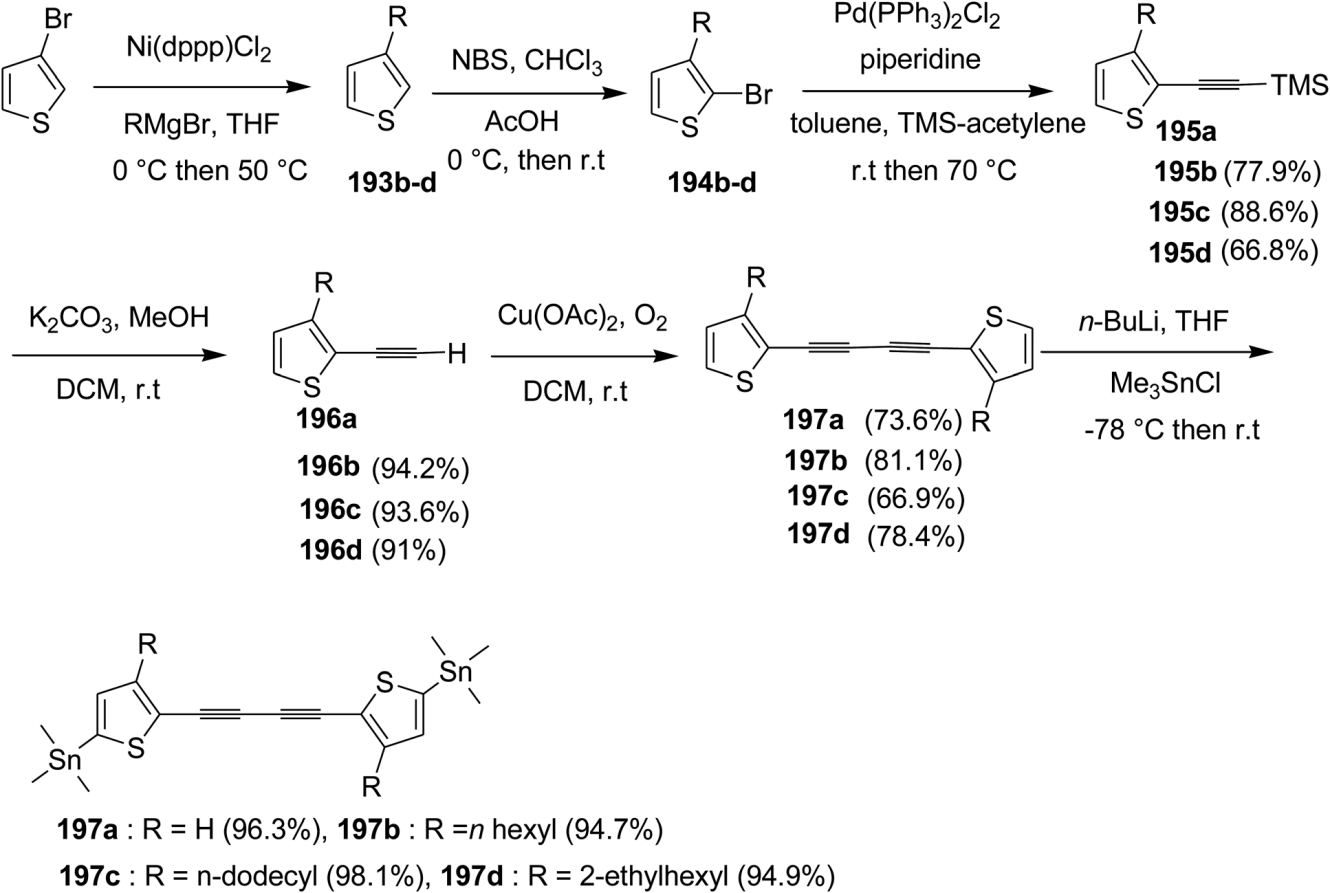
Synthesis of DTB monomers 197a–d.

**Scheme 61:**
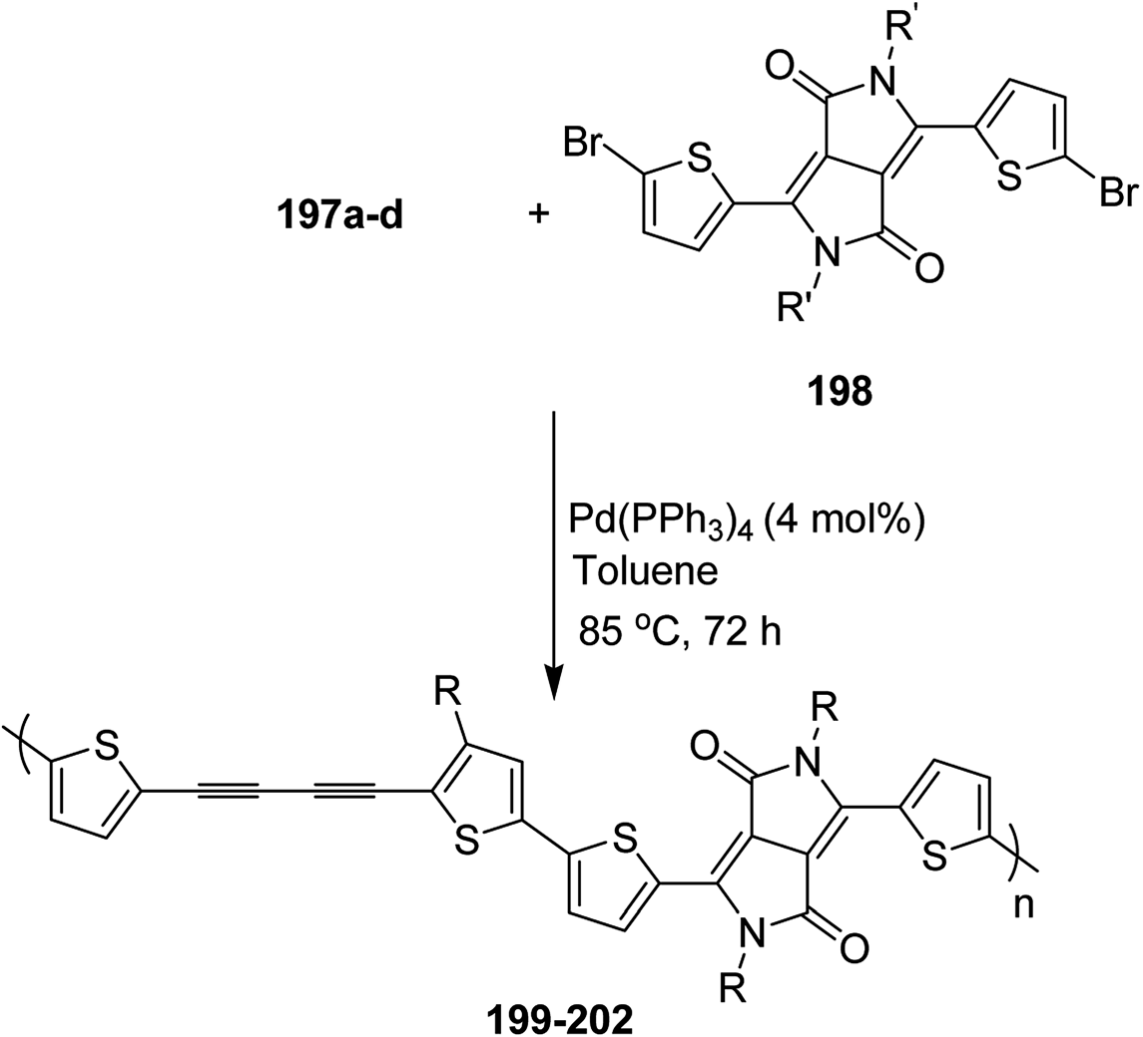
Polymerization of monomers 197a–d to obtain polymers 199–202.

Synthesis of DTB monomers started with Ni-catalyzed alkylation of 3-bromothiophene to obtain alkylated thiophenes 193b–d which were further brominated with NBS to afford 3-alkyl-2-bromothiophenes 194b–d. They were coupled with ethynyltrimethylsilane using Pd-catalyzed Sonogashira coupling to afford intermediates 195b–d. Removal of the protecting TMS group under basic conditions yielded 196a–d. Cu-catalyzed Glazer–Hay reaction was used for the homocoupling of terminal alkynes, 2-ethynylthiophenes, to obtain 1,3-butadienes 195a–d (Scheme 60). The respective polymers 199–202 were synthesized by Stille coupling polymerization of differently substituted stannylated DTB monomers with 3,6-bis(5-bromothiophen-2-yl)-2,5-bis(alkyl)pyrrolo[3,4-*c*]pyrrole-1,4(2*H*,5*H*)-dione (TDPP-Br_2_) 198 monomers (Scheme 61).
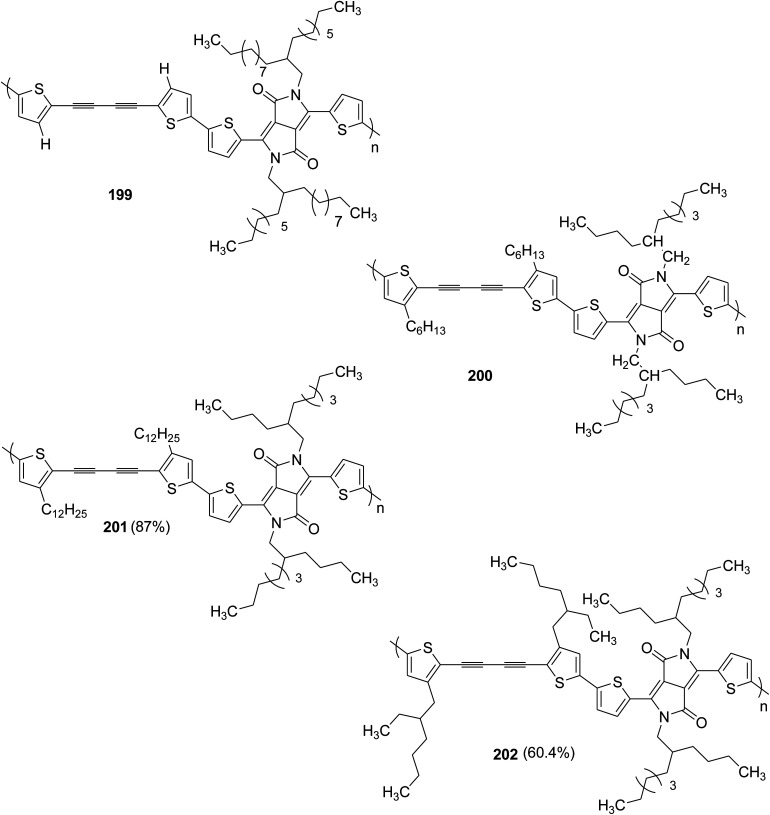


In 2017, Wenbin Lai and co-workers reported the synthesis of three novel single-component conjugated polymers possessing diketopyrrolopyrrole (DPP)-based backbone and perylene bisimide (PBI) as pendant connected with flexible alkyl chains for single-component organic solar cells (SCOSCs).^250^ DPP-based polymers are widely used as electron donors and reported to show high performance in organic solar cells.^240,251–253^ PBI derivatives with twisted and fused backbones are also used in fullerene solar cells, where they were demonstrated have superior electron transport properties.^254–256^ Therefore, it was an interesting idea to incorporate π-conjugated PB units and semicrystalline DPP backbone into one material.

Near-infrared absorbing DPP polymer PDPP2TBTD, in which DPP is alternating with benzodithiophene (BD) units, was selected to synthesize a single-component polymer 208. 209 was constructed by tuning the distance between PBI and DPP. In 210 polymer, alkylthiothiophene side chain was introduced on the BDT unit. These structural modifications helped to improve the nanophase separation of the polymers, which resulted in a PCE of 2.74% in polymer 210 based single-component organic solar cells, compared to 0.5% in 208 polymer as a photoactive layer.

**Scheme 62:**
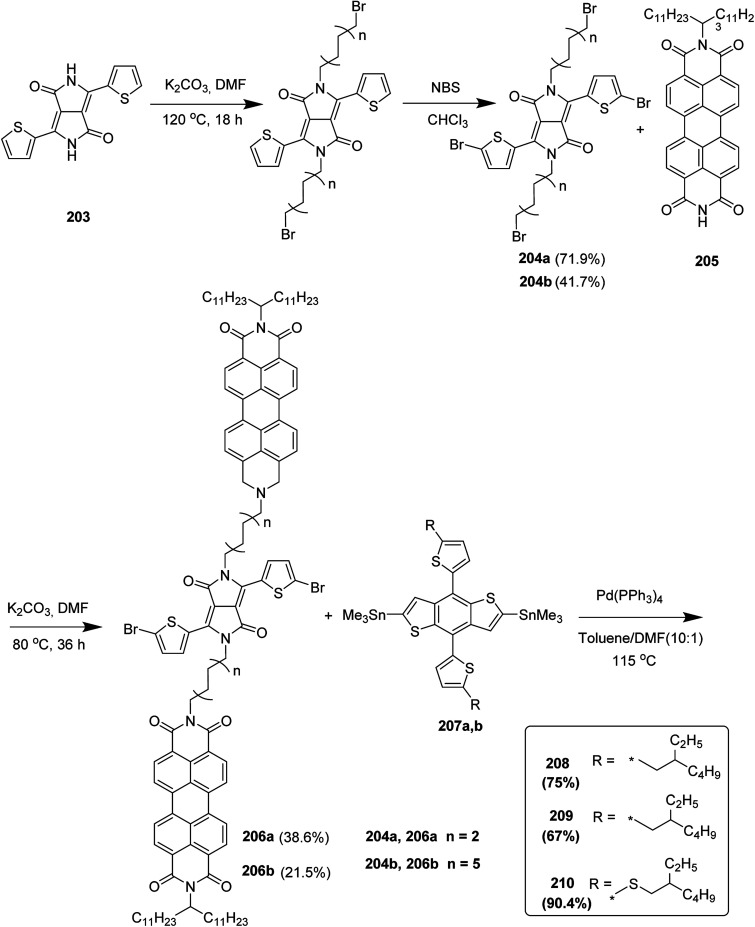
Synthetic route for the synthesis of diketopyrrolopyrrole (DPP)-based monomers 206a,b and polymers 208–210.

Starting from the DPP 203, precursors 204a and 204b were synthesized through alkylation and bromination reactions, where the four bromine atoms exhibited distinct reactivity enabling the introduction of perylene bisimide (PBI) side chain into diketopyrrolopyrrole (DPP) molecule with the help of monoalkylated PBI compound 205, affording DPP–PBI monomers 206a and 206b. Stille coupling polymerization was used to synthesize polymers 208, 209 and 210, using distannyl-BDT monomers 207a and 207b containing alkylthiophene or alkylthio-thiophene side chains. These polymers were readily soluble in chloroform. The molecular weight (*M*_n_) determined by GPC in 1,2,4-trichlorobenzene at 160 °C was 6.6, 23.5 and 20.7 kg mol^−1^ for the polymers 208, 209 and 210, respectively. All the polymers exhibited good thermal stability with 5% weight loss at temperature above 340 °C (Scheme 62).
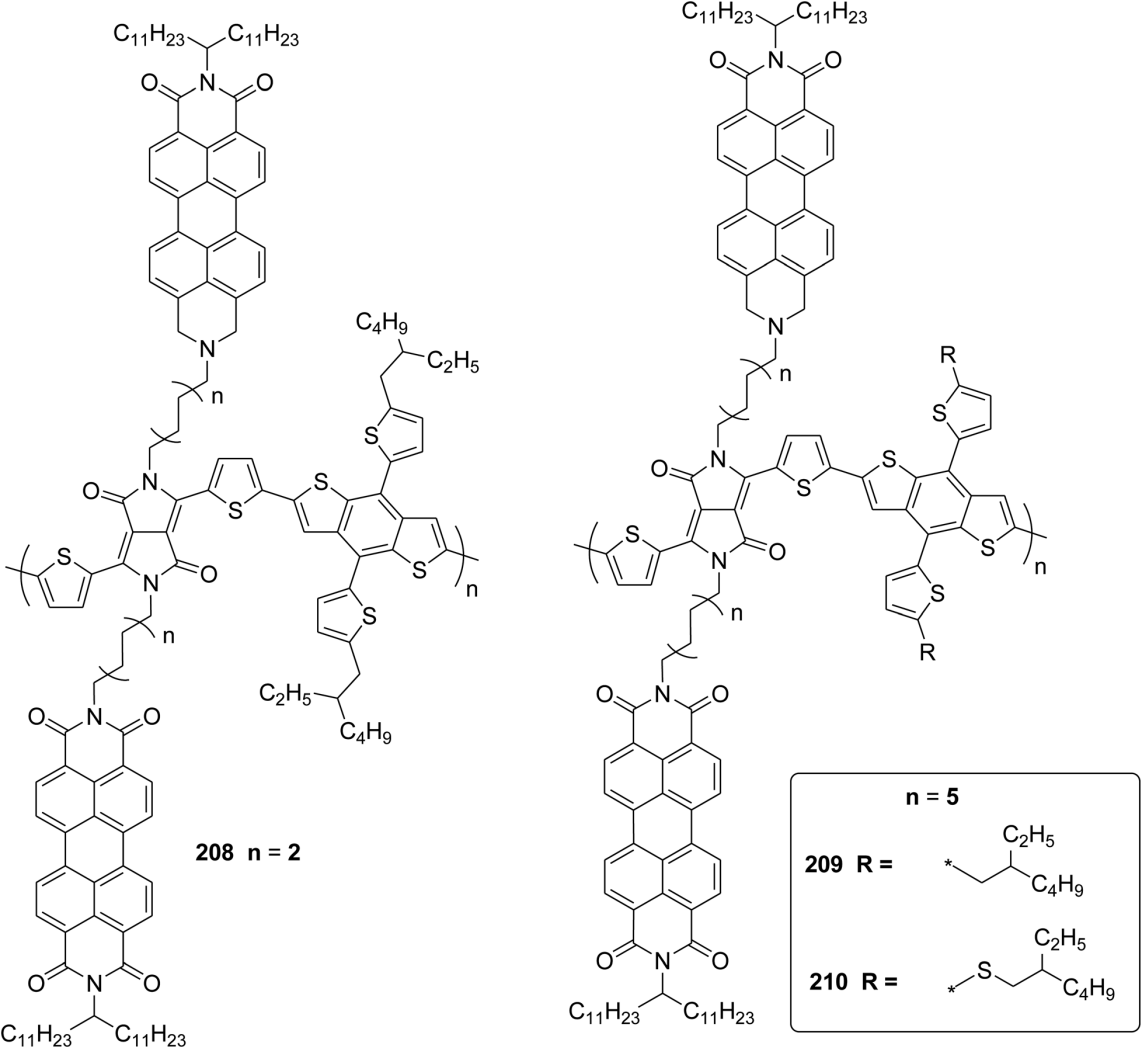


In 2018, Bogyu Lim and co-workers proposed a new strategy to synthesize well-defined regioregular alternative D–A polymers, using large molecular weight regioregular monomers (LRMs) 212.^257^ A regioregular alternative D–A polymer, rr-PBTTh, was synthesized by a systematic introduction of various conjugated moieties in a single polymer backbone using thiophene as a donor and benzothiadiazole as an acceptor unit. This polymer exhibited a highly planar conjugated backbone because of regioregularity and S⋯F and S⋯O intramolecular conformational locks. rr-PBTTh 218a–c displayed well-balanced ambipolar transport characteristics largely due to the balanced D–A molecular structure and showed promising electrochromic performance with coloration efficiency upto 321.7 cm^2^ C^−1^ and rapid response time below 0.5 s.

**Scheme 63:**
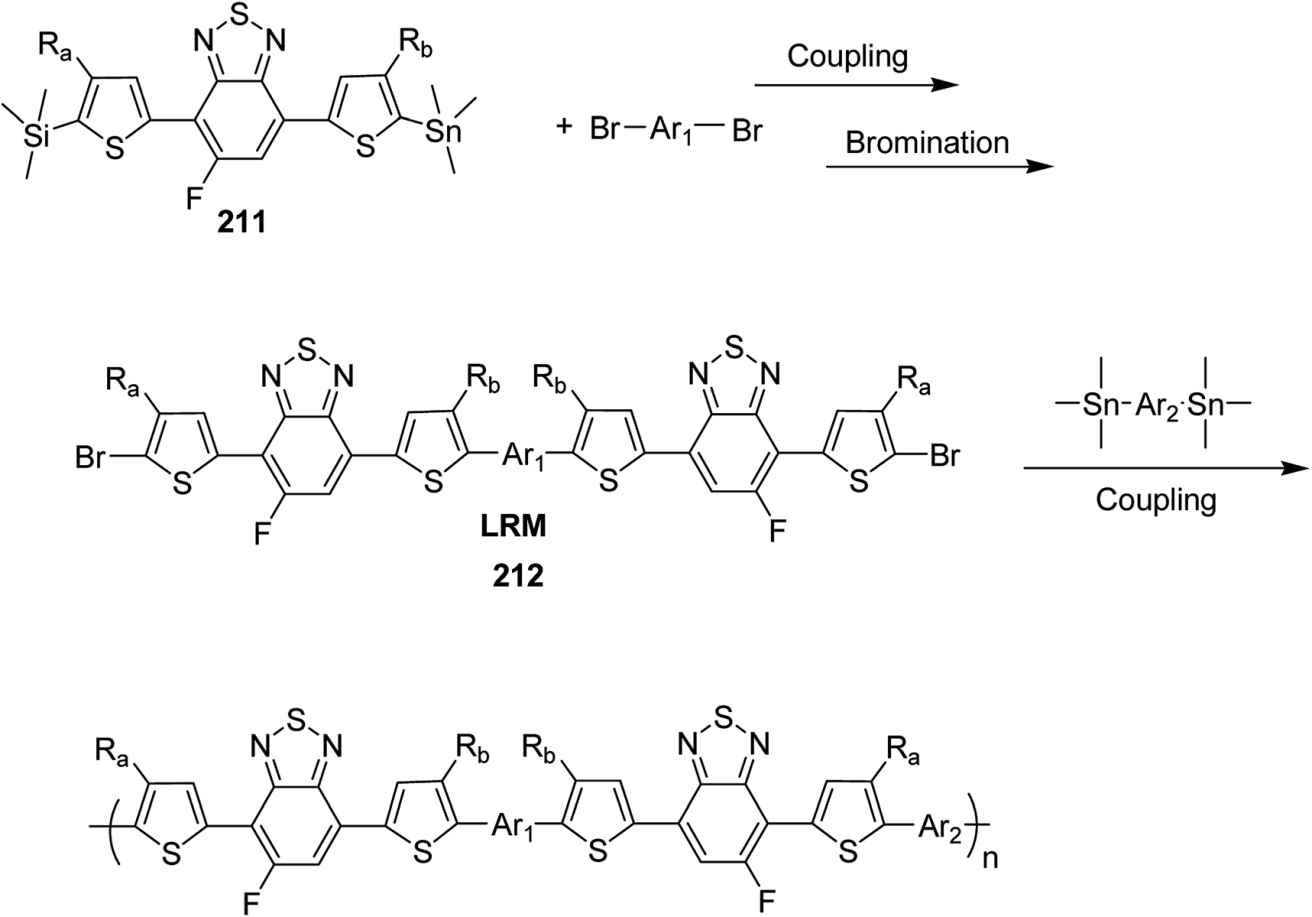
Synthetic method for a well defined regioregular polymer intermediate 211.

**Scheme 64:**
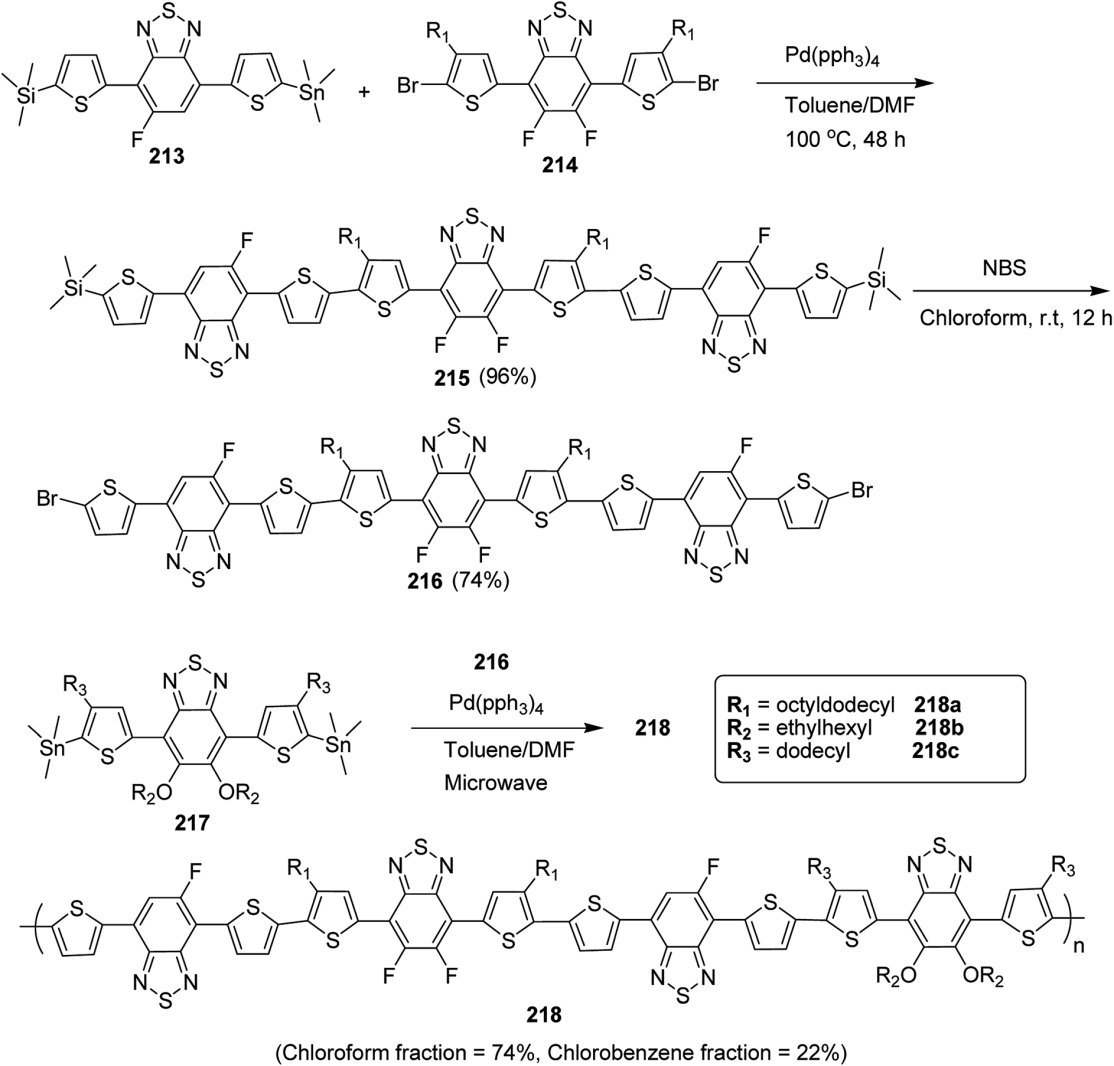
Synthesis of regioregular polymers 218a–c.

An intermediate compound 211 was prepared which provided various LRMs through simple coupling and bromination with various dibromo-Ar_1_ compounds. A large variety of polymers can be produced by these LRMs with diverse diboronic-Ar_2_ (for Suzuki reaction) or distannyl-Ar_2_ (for Stille reaction) compound. Mono fluorinated benzothiadiazole based intermediate 213 was coupled with 214*via* Stille coupling reaction to afford LRM 216. An undesirable homo-coupled by-product 215 was produced during the coupling reaction of compound 213 and 214. However, relatively high retention factor (*R*_f_) and relatively good solubility of ditrimethylsilyl based by-product 215 in chloroform and DCM eluents for column chromatography explained that the by-product could be removed after bromination. Finally, the regioregular polymers 218a–c were synthesized by a microwave-assisted Stille coupling polymerization of LRM 217 and distannyl-alkylthiophene-alkoxybenzothiadiazole based monomer 217 (Schemes 63 and 64).

**Scheme 65:**
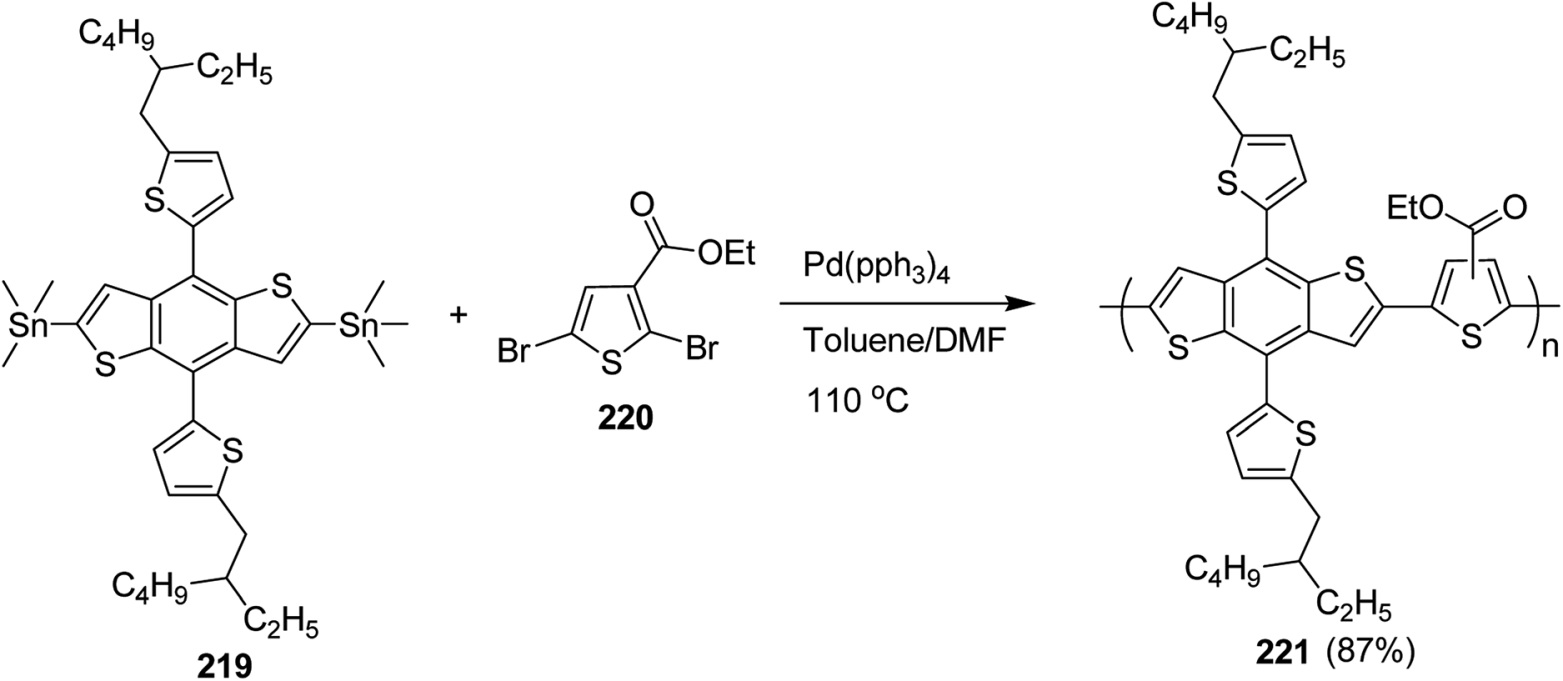
Synthesis of regiorandom polymer 221.

**Scheme 66:**
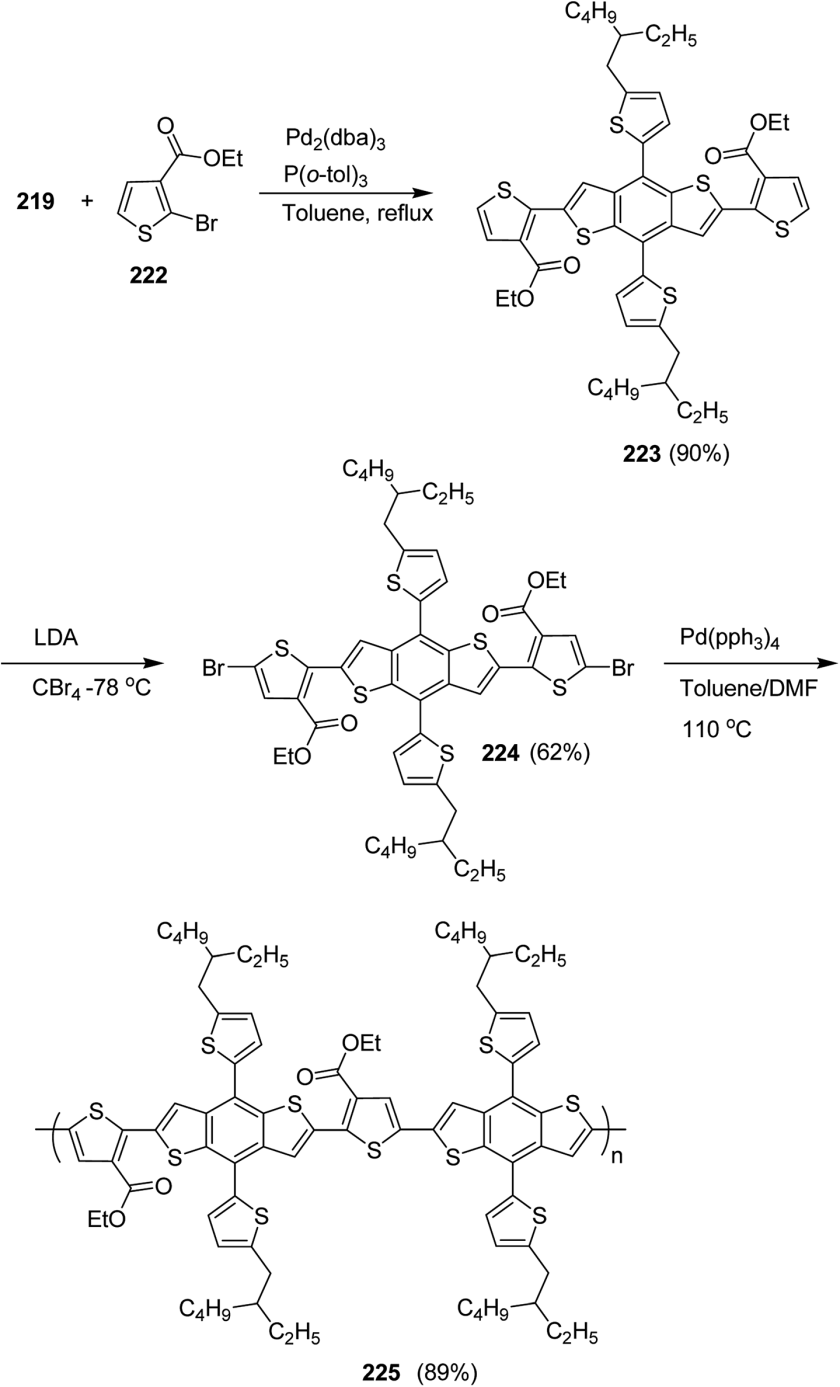
Synthesis of regioregular polymer 225.

Yahui Liu and co-workers in 2018 developed a regioregular wide bandgap polymer as a donor material to enhance the performance of non-flurrene organic solar cells.^258^ In films, regioregular polymer reg-PthE 225 (Scheme 66) showed closer packing of the polymer backbone and a large absorption coefficient as compared to the corresponding random polymer ran-PthE 221 (Scheme 65). Devices based upon reg-PthE:FTIC realized a high power conversion efficiency (PCE) of 12.07%, while the devices based on ran-PthE:FTIC achieved PCE of 9.89%. With ITCC as an acceptor, PCEs of 11.21% and 8.38% were achieved for reg-PThE and ran-PthE, respectively. Semitransparent organic solar cells having reg-PthE:FTIC, as an active layer, exhibited a PCE of 8.69% and an average visible transmittance of ∼25%.

Regioregular polymer 225 (Scheme 66) and regiorandom 221 (Scheme 65) were synthesized in 89 and 87% yields, respectively, by Stille coupling polymerization of bis(stannyl) compound 219 with ethyl-2,5-dibromothiophen-3-carboxylate 220 and dibromo monomer 223, respectively. Monomer 223 was prepared by the Stille cross-coupling reaction of 219 and monobromo 222. Lithium diisopropylamine (LDA) was empolyed to abstract hydrogens from the α-position of the thiophene moiety to afford the corresponding dianions, which were subsequently quenched with CBr_4_ to obtain dibrominated monomer 224 in 62% yield. The molecular weight (*M*_n_) and polydispersity index (PDI) were 82 kg mol^−1^ and 2.72 for reg-PThE and 62 kg mol^−1^ and 1.73 for rand-PThE, respectively. Ran-PThE was fairely soluble in chlorobenzene and 1,2-dichlorobenzene at room temperature, whereas reg-PThE was only soluble in these solvents at elevated temperature.

In 2018, Zhangfeng Li and co-workers reported the synthesis of A–D_1_–A–D_2_-type regioregular polymer 229 and regiorandom polymer 230, having the similar polymeric backbone, through two different routes.^259^ Both polymers, 229 (synthesized *via* two steps method) and 230 (synthesized *via* one step Stille coupling polymerization), incorporated two different donor units thieno[3,2-*b*]thiophene (TT) 226 and terthiophene (3T) 228 and one acceptor unit (dialkyloxybenzothiadiazole, ROBT) 225 in their backbone. TT and 3T units could induce strong π–π stacking properties because of their extended π-conjugation. Moreover, a good planarity was ensured due to the interchain noncovalent S⋯O coulombic interactions between oligothiophenes (TT and 3T) and ROBT in terpolymer 229 and 230, exhibiting large absorption bands ranging from 300 to 750 nm. Regiorandom polymer 230 showed higher power conversion efficiency of 3.40% as compared to the regioregular polymer 229 (3.00%). Higher PCE of 230 was attributed to improved short circuit current (*J*_sc_), fill factor (FF) and high hole mobility. Although the UV-visible absorption of polymer 229 was gradual red-shifted than 230, the rough surface of the active layer based on P1:PC_71_BM reduced short circuit current and charge separation, resulting in the lower polymer conversion efficiency of 229, compared to 230. These results suggested that the random polymer could be beneficial for the improvement of photovoltaic properties of conjugated polymers for polymer solar cells.

**Scheme 67:**
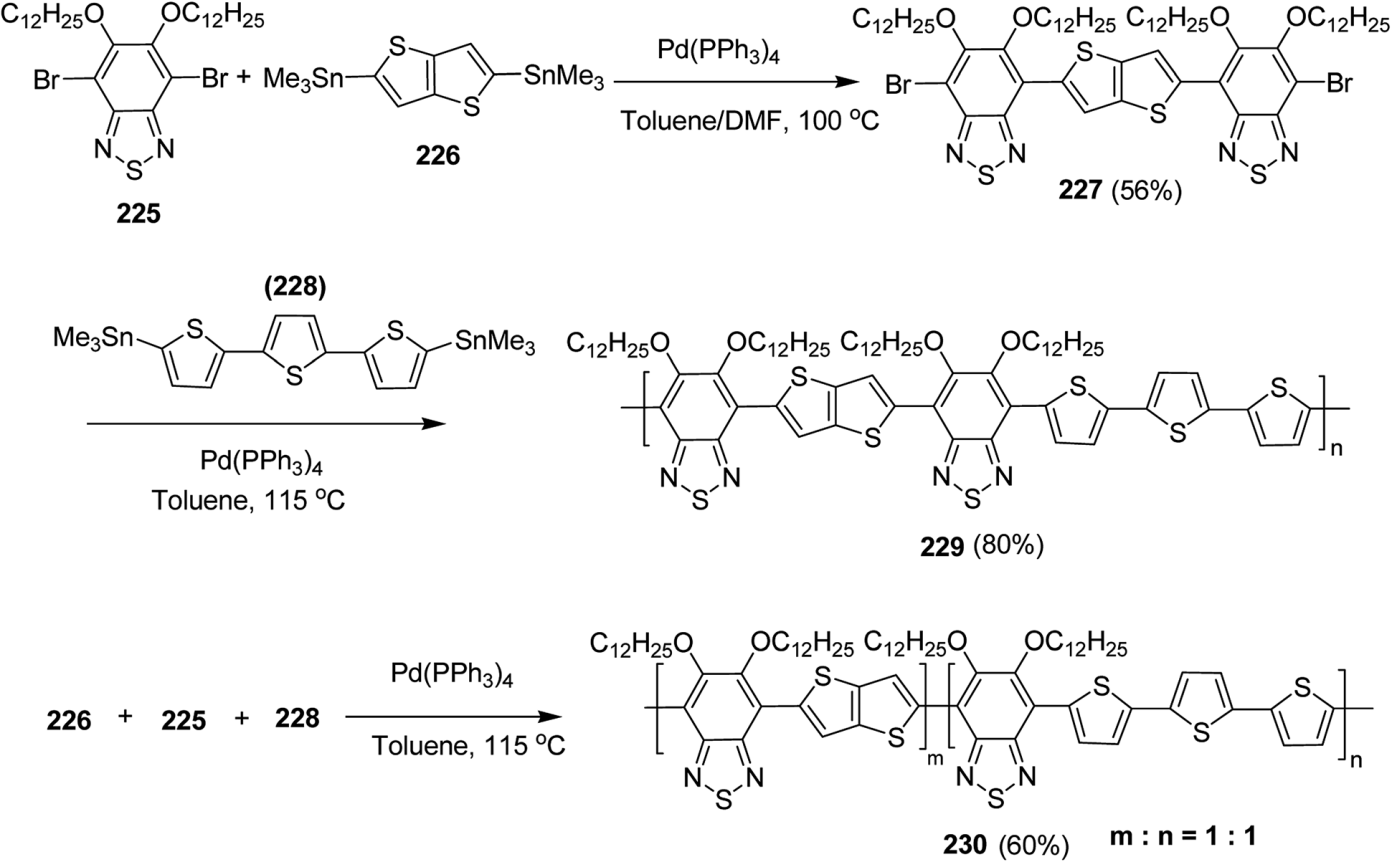
Preperation of regioregular polymer 229 and regiorandom polymer 230.

The monomer 227, used as a starting material for coupling reactions, was synthesized by 1 eq. of ROBT 225 and 2.5 eq. of TT 226. In order to avoid over polymerization of 227, the monomer 226 was slowly added into the reaction mixture and the polymer 229 was synthesized *via* two step Stille coupling reactions between 227 and 3T 228. The regiorandom polymer 230, containing TT, ROBT and 3T units was synthesized *via* one step Stille coupling reaction. Solubility of the copolymers was enhanced by the presence of two alkoxy chains on 225 (Scheme 67). Molecular weight and PDI values of both polymers are enlisted in Table 9, whilest an overview of the PSCs properties of the polymer based on the polymers:PC_71_BM is given in Table 10.

**Table 9:** Molecular weight and polydispersity index of 229 and 230

Polymers	*M*_w_ (kDa)	*M*_n_ (kDa)	PDI
P1 (227)	74.66	15.99	4.66
PR1 (228)	73.42	14.77	4.97

**Table 10:** The PSCs properties of the polymer based on the polymers:PC_71_BM with or without DIO^a^

Active layer	Solvents/vol	*V*_oc_ (V)	*J*_sc_ (mA cm^−2^)	*J*_sc_ b (mA cm^−2^)	FF	PCE^c^ (%)	PCE^best^ (%)
P1:PC_71_BM	W/O	0.68	7.85	7.00	0.49	2.54 ± 0.12	2.64
P1:PC_71_BM	4% DIO	0.62	8.22	8.00	0.59	2.98 ± 0.12	3.00
PR1:PC_71_BM	W/O	0.65	7.05	6.80	0.54	2.43 ± 0.12	2.52
PR1:PC_71_BM	4% DIO	0.62	8.53	8.64	0.60	3.30 ± 0.10	3.40

a All the active layers were spin-coated following the same method, resulting in a similar thickning of ∼100 nm.

b The EQE-integrated *J*_sc_.

c The statistic parameters were obtained from 10 individual devices.

### 4.3. Application of Pd-catalyzed direct arylation polymerization towards synthesis of thiophene-based polymers

Conventional carbon–carbon bond forming cross-coupling reactions such as Suzuki, Kumada, Negishi and Stille are of great relevance in organic and polymer chemistry. Hovewer, the use of various organometallic reagents is considered as a drawback of these coupling reactions because of the stoichiometric amount of byproducts formed during the coupling reactions. These reagents and the resulting byproducts are often toxic and environmentally risky, especially stannyl derivatives. Synthesis of the organometallic monomers often consists of multiple steps, purification of which is quite tedious. In order to address these shortcomings, a new synthetic approach, called direct arylation polymerization (DArP), came into the focus of attention of scientific community and has been developed to a great extent during the last few years. DArP offers a simple, relatively inexpensive and environment friendly protocol to construct C–C bond between an (hetero) aryl halide and a non-preactivated (hetero) aryl by directly activating one of its C–H bonds.^260–262^ This synthetic strategy eliminates the need of functionalizing the monomers with expensive organotin and organoboron derivatives which are essential for Stille and Suzuki cross-coupling polymerizations. This development resulted in up to 35% reduction in the fabrication cost of organic electronics.^263^ DArP is highly sustainable and attractive for the large-scale production of conjugated polymers due to high atom economy and few, if any, toxic byproducts.^264,265^ Well-defined and high molecular weight conjugated polymers are now available with a courtesy of this approach and some of the very recent example are discussed herein.

**Scheme 68:**
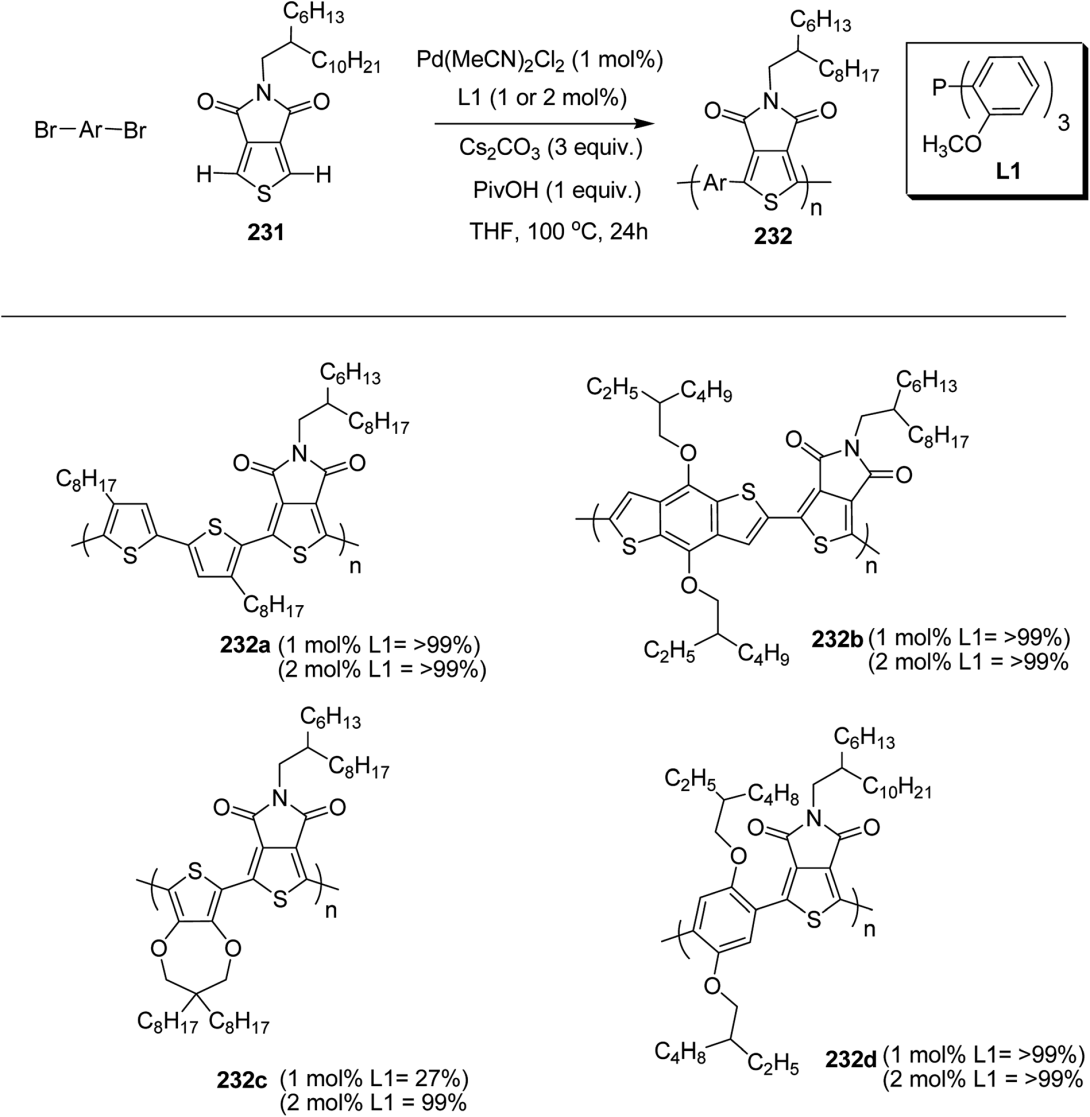
Polycondensation of dibromoarenes and thieno[3,4-*c*]pyrrole-4,6-dione *via* direct arylation polymerization to synthesize 232a–d.

In 2012, Philippe Berrouard and co-workers, for the very first time reported the polymerization of 5-(2-hexyldecyl)-5*H*-thieno[3,4-*c*]pyrrole-4,6-dione-1,3-diyl (TPD) 231 monomer as an acceptor unit using Pd-catalyzed direct arylation polymerization.^266^ In 2014, Masayuki Wakioka and co-workers conducted the same reaction under reduced catalyst loading^267^ as the palladium residue in π-conjugated polymers is known to produce detrimental effects on the device performance. This new study revealed that amount of palladium could be reduced to 1/8 of the amount used in the previous study by using Pd(MeCN)_2_Cl_2_ as catalyst along with P(C_6_H_4_-*o*-OMe)_3_ as a ligand. Polymerization proceeded smoothly at 100 °C in THF in the presence of Cs_2_CO_3_ and pivalic acid to yield TPD-based polymers, containing 4,4′-dioctyl-2,2′-bithiophene-5,5′-diyl (232a, *M*_n_ = 36 800 g mol^−1^), 4,8-bis(2-ethylhexyloxy)benzo[1,2-*b*:4,5-*b*′]dithiophene-2-diyl (232b, *M*_n_ = 31 100 g mol^−1^), 3,4-(2,2′-dioctylpropylenedioxy)thiophene-2,5-diyl (232c, *M*_n_ = 68 200 g mol^−1^) and 2,5-bis(2-ethylhexyloxy)-1,4-phenylene (232d, *M*_n_ = 65 500 g mol^−1^) (Scheme 68).

Patrick D. Homyak and co-workers in 2013 reported the use of direct arylation polymerization to synthesize new low bandgap poly(thienothiophene-*alt*-dithienylbenzodithiophene) (PTB) polymers,^268^ by reacting dithienylbenzodithiophene with thieno[3,4-*b*]thiophene acceptor blocks containing phenyl, octyl, perfluorophenyl and perfluorooctyl side groups. The strongly electron withdrawing perfluorophenyl and perfluorooctyl were observed to significantly lower the energy of both HOMO and LUMO levels. These materials showed favorably aligned energy levels compared to conventional fullerene type acceptors.

**Scheme 69:**
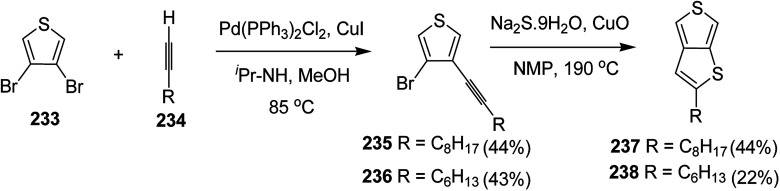
Synthesis of monomers 237 and 238.

The monomers with non-fluorinated monomeric units, 237 and 238, were synthesized by selective coupling of 3,4-dibromothiophene 233 with terminal alkyne 234*via* Sonogashira coupling reaction yielding compounds 235 and 236 with 44% and 43% yields, respectively. Subsequent cyclization of 235 and 236 with sodium sulfide in the presence of CuO at elevated temperature afforded 237 and 238 with 44% and 22% yields, respectively (Scheme 69).

**Scheme 70:**
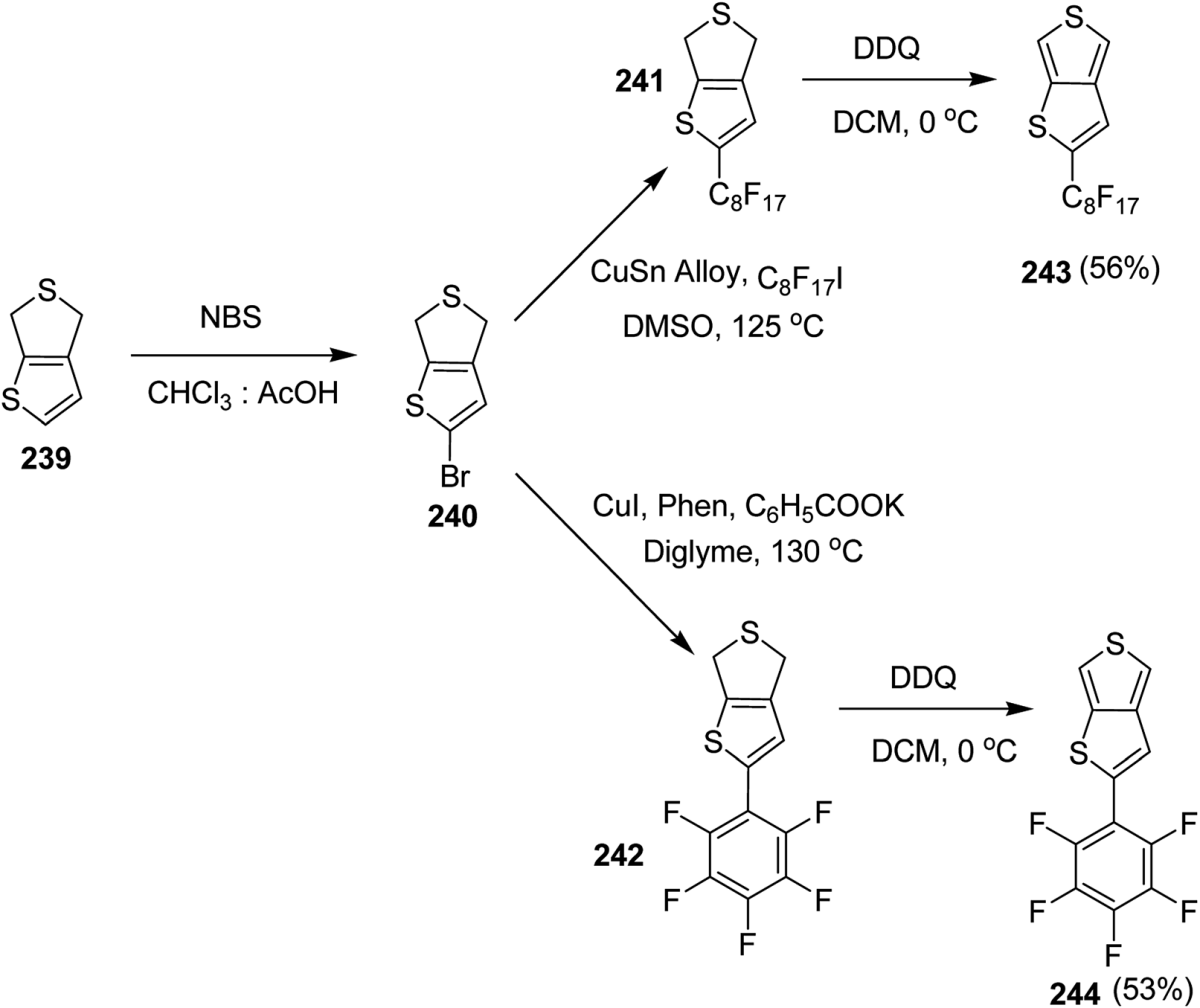
Synthesis of monomers 243 and 244.

Synthesis of 243 and 244 was performed by using dihydrothieno[3,4-*b*]thiophene 239, which was brominated by using a 1 : 1 mixture of acetic acid and chloroform to afford 240, which was converted to 241 and 242*via* existing coupling protocols designed for the thiophene derivatives.^269–274^ Subsequent oxidation of 241 and 242 by 2,3-dichloro-5,6-dicyano-1,4-benzoquinone (DDQ) yielded fluorinated monomers 243 and 244 with 56% and 53% yields, respectively (Scheme 70).

**Scheme 71:**
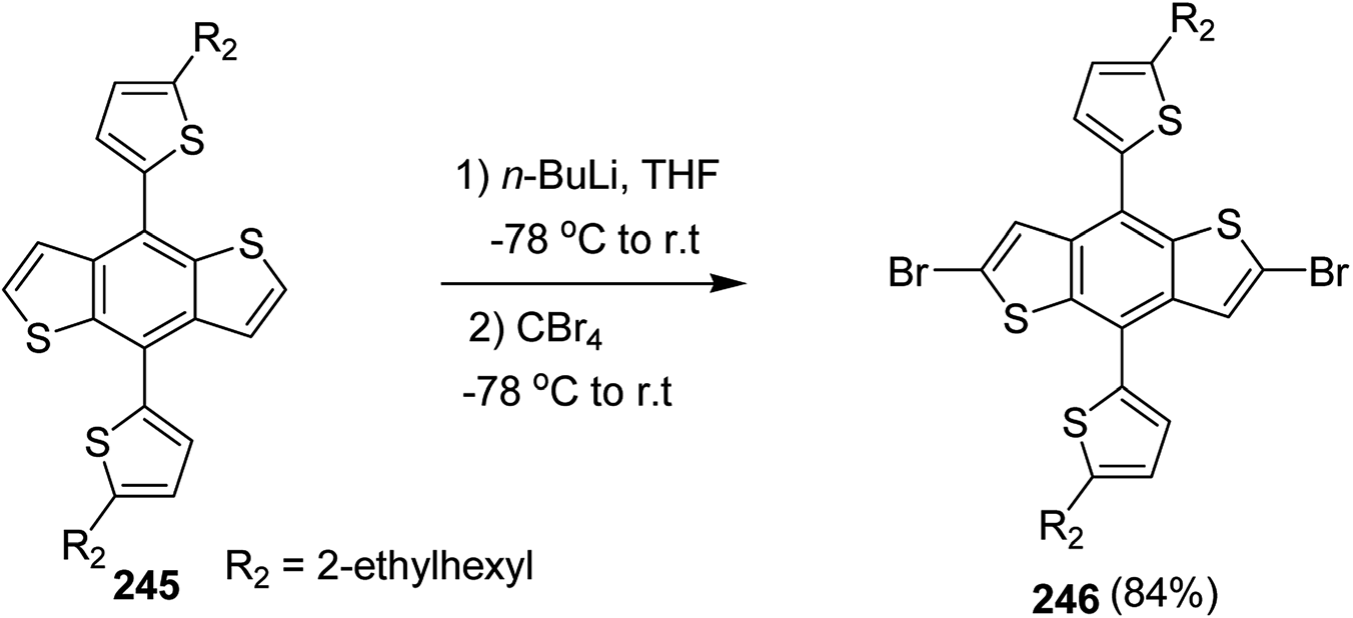
Synthesis of 246.

Monomer 246 was prepared by selective bromination of dithienylbenzodithiophene 245. Lithiation of the two α-thiophene positions on 245 and subsequent addition of CBr_4_ yielded 246 in 84% yield (Scheme 71).

**Scheme 72:**
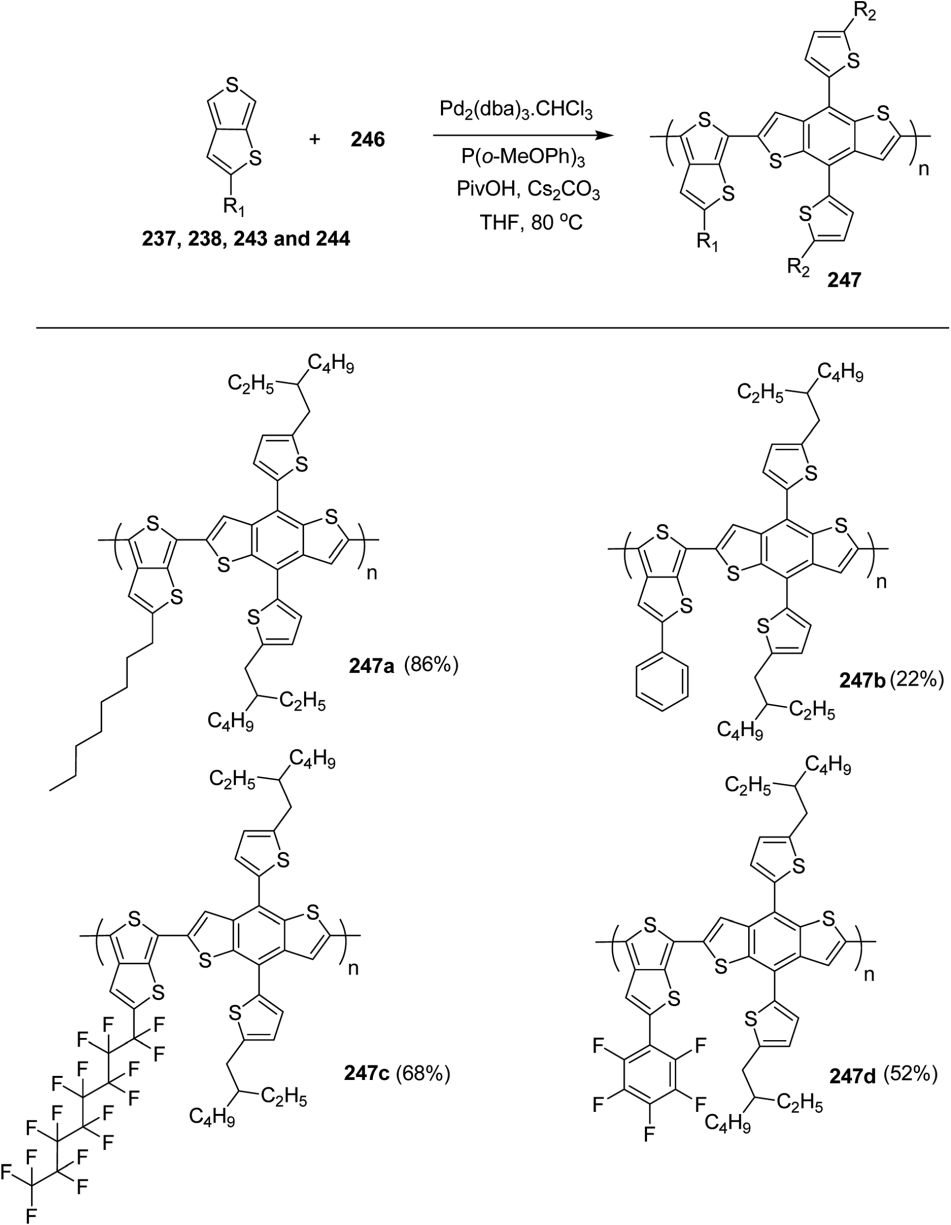
Synthesis of polymers 247a–d.

A series of alternating copolymers was synthesized by utilizing aryl halide bonds in monomer 246 and activated C–H bonds in monomers 237, 238, 243 and 244*via* direct arylation polymerization. Catalyst system comprising of Pd_2_(dba)_3_·CHCl_3_ and tris(2-methoxyphenyl)phosphine resulted in a rapid polymerization to yield 247a–d. The polymers with weight-average molecular weights (*M*_w_) of 18–77 kDa were afforded after one hour of reaction time. Portion of insoluble material was found to be increased by increasing reaction time, presumably due to cross-linking at either the β-thiophene or 3-thieno[3,4-*b*]thiophene on the benzodithiophene (Scheme 72).

**Scheme 73:**
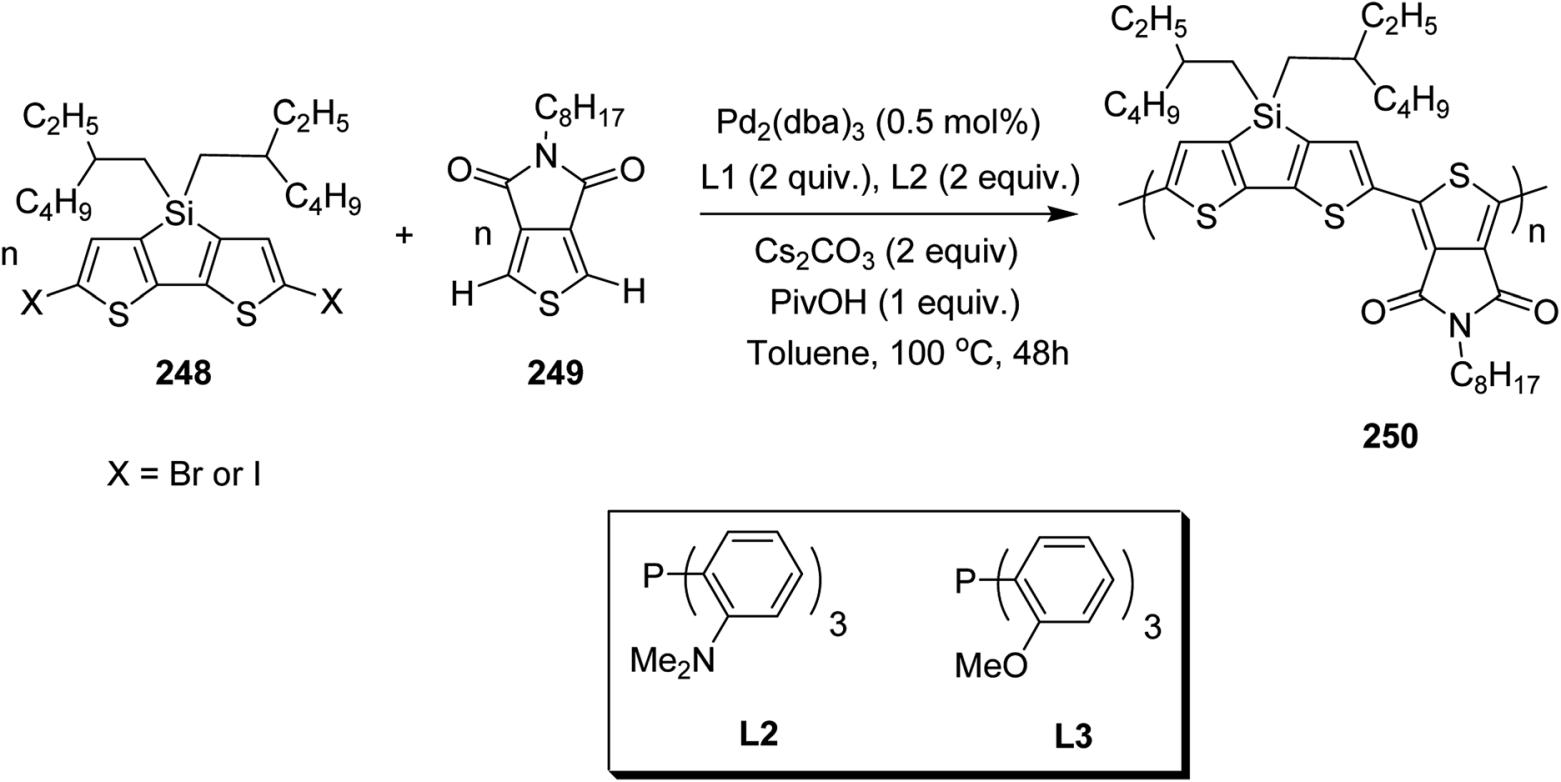
Direct arylation polymerization of 248 and 249 using a mixture of ligands L2 and L3 to synthesize 250.

In 2015, Eisuke Iizuka and co-workers reported a novel catalytic system for the synthesis of alternating copolymers of dithienosilole (DTS) 248 and thienopyrroledione (TPD) 249*via* Pd-catalyzed direct arylation polymerization approach.^275^ Combination of P(2-Me_2_NC_6_H_4_)_3_ (L2) and P(2-MeOC_6_H_4_)_3_ (L3) ligands was found to be very effective in preventing structural defects as well as formation of the side products. The polymer (DTS-*alt*-TPD) 250 was prepared by Migita–Stille cross-coupling polymerization and was reported for the first time in 2011. Attempts to make this polymer *via* direct arylation polymerization (DArP) resulted in the formation of insoluble byproducts, and the synthetic results were reported to be difficult to reproduce. The solution to this problem was found in the use of a mixture of two ligands, P(2-Me_2_NC_6_H_4_)_3_ (L2) and P(2-MeOC_6_H_4_)_3_ (L3), which proved very efficient and sufficiently reactive even in toluene as non-polar solvent and produced a copolymer with number average molecular weight of 15 000 g mol^−1^ (Scheme 73).

**Scheme 74:**
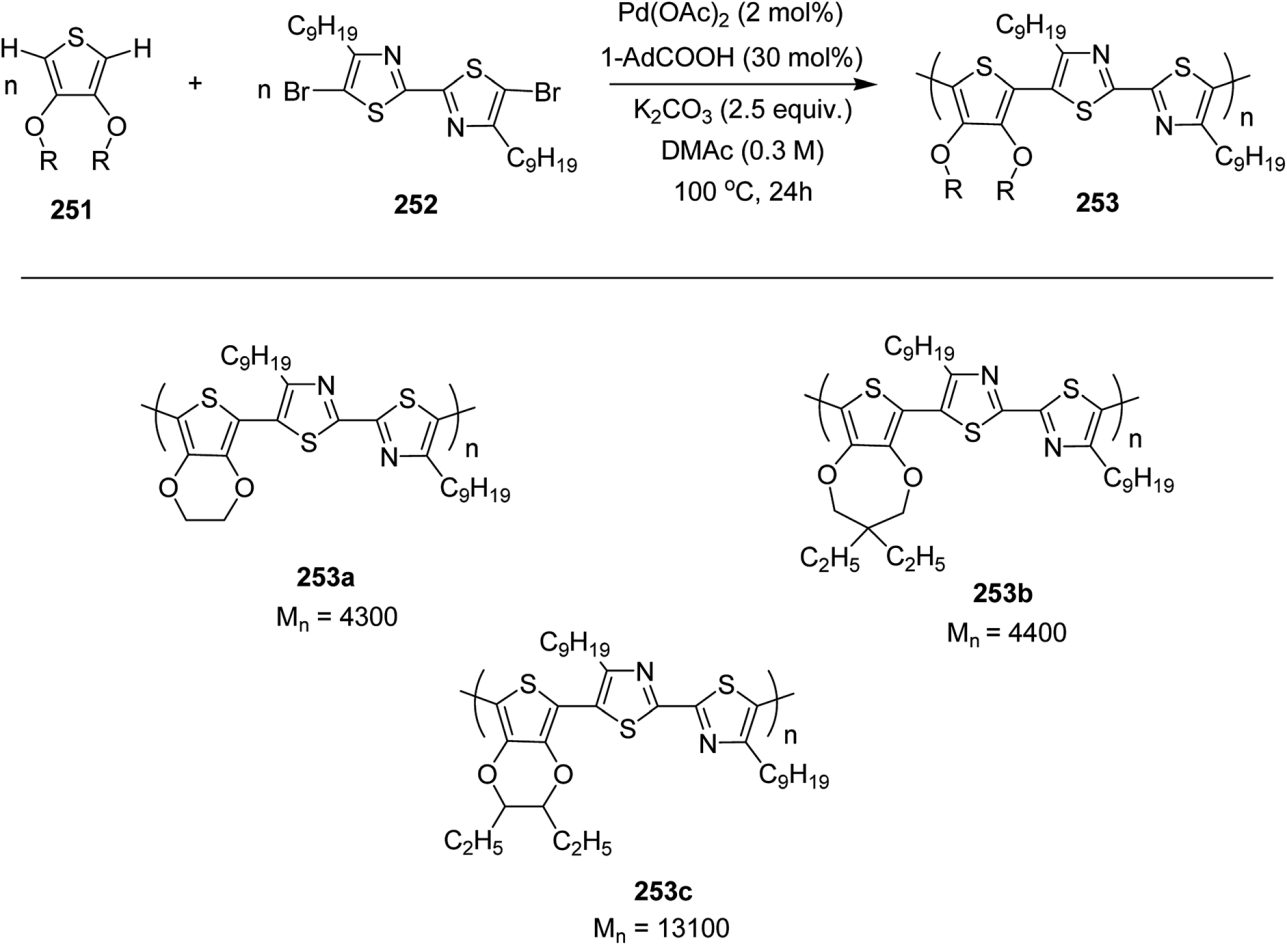
Polycondensation of EDOT derivatives with 5,5′-dibromo-4,40-dinonyl-2,2′-bithiazole 252 to synthesize polymers 253a–c.

In 2015, Wei Lu and co-workers reported the use of direct arylation polycondensation for the synthesis of highly crystalline bithiazole-based donor–acceptor type copolymers 253a–c, where bithiazole 252 served as an acceptor unit while 3,4-ethylenedioxythiophene (EDOT) derivatives 251 were employed as donors.^276^ Installment of long chain alkyls on the bithiazole monomers increased their solubility in the solvents used for polymerization reaction and, thus, proved helpful in obtaining high molecular weight polymers (Scheme 74).

**Scheme 75:**
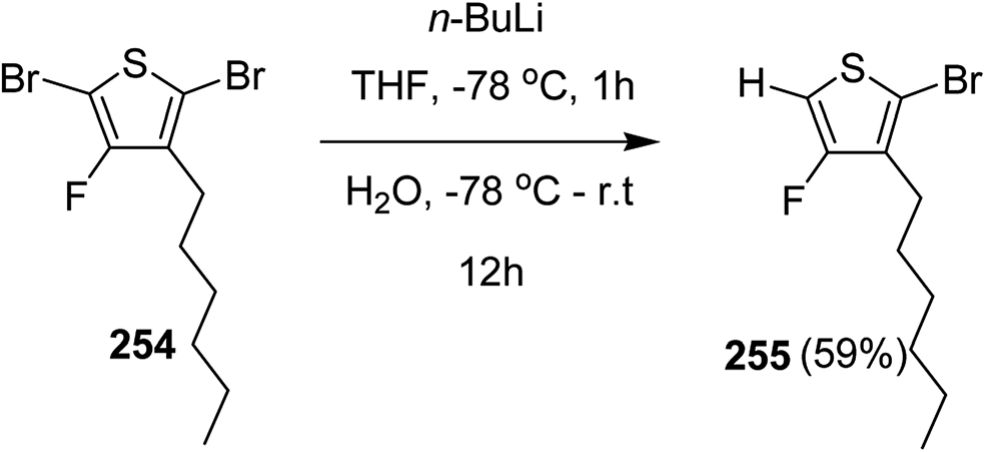
Synthesis of monomer 255 for DArP.

Patrick D. Homyak and co-workers in 2016 presented a strategy for tuning the physical properties of P3HT-based copolymers by incorporation of folurinated thiophene repeating units.^277^ Fluorinated polythiophene P(3HT-*co*-3H4FT) 257 was synthesized by systematically varying percentage of fluorinated monomer in the polymer backbone from 0 to 100%. Monomer modification approach along with a direct arylation polymerization was used to synthesize these fluorinated polythiophenes [P0 (257a), P25 (257b), P50 (257c), P75 (257d), and P100 (257e)] to demonstrate that the percentage of fluorination in the copolymer could be precisely tuned by monomer feed ratio, hence, varying the properties of P3HT polymers according to the desired amount of fluorination. Percentage of fluorinated monomer could be easily controlled by changing the feed ratio of the two monomers. Direct arylation polymerization was enabled by the use of an A–B type monomer, which contained both an arylbromide and C_SP_^2^–H bond. Initially, 2-bromo-3-hexyl-4-fluorothiophene 255 was synthesized from 2,5-dibromo-3-hexyl-4-fluorothiophene 254. Lithiation of this compound with *n*-BuLi occurred selectively at position-5 due to the presence of strong electron-withdrawing fluorine group at position-5. The desired monomer 255 was obtained after quenching the lithiated intermediate with water (Scheme 75).

**Scheme 76:**
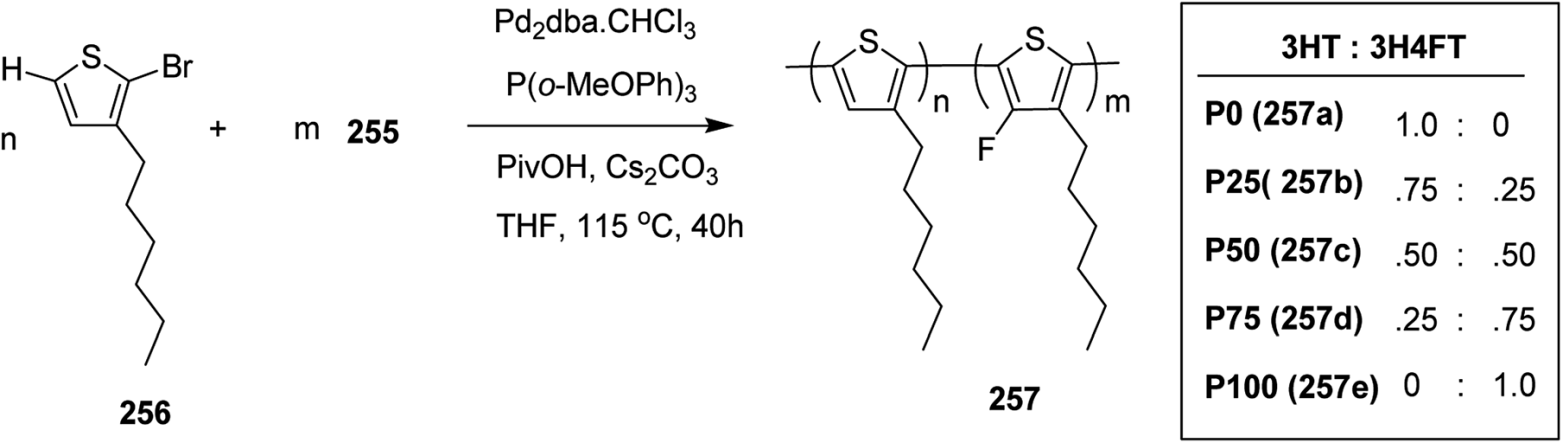
Synthesis of 257a–e (P0–P100) copolymers by DArP.

Five copolymers 257a–d, were synthesized by changing the 253 : 254 feed ratio with the 0, 25, 50, 75 and 100% of fluorinated monomeric unit corresponding to the P0, P25, P50, P75 and P100, respectively (Scheme 76). An overview of the composition, molecular weight and regioregularity of P(3HT-*co*-3H4FT)s is given in Table 11. Electronic properties of the polymers were strongly affected by increasing fluorination as evidented by the decrease in the *E*_HOMO_ level by 0.4 eV for P100 as compared to P0, which is much significant than the previously reported examples of fluorinated conjugated polymers having smaller shifts of approximately 0.1–0.2 eV. The capability to adjust *E*_HOMO_ over a range of approximately 0.4 eV could be useful for tuning exact energy levels for the optimum energy level alignment, better charge separation and improved open circuit voltage *V*_oc_.

**Table 11:** Composition, molecular weight and regioregularity of polymers 257a–e

	*M*_n_ a (kg mol^−1^)	*M*_w_ a (kg mol^−1^)	*Đ*	RR^b^ (%)	*F* c (%)
P0	13.0	18.2	1.4	95	0
P25	12.9	17.8	1.4	89	27
P50	16.4	32.5	2.0	85	50
P75	12.4	20.2	1.6	81	75
P100	5.6	6.9	1.2	78	100

a Measured by GPC *versus* polystyrene standards in 1,2,4-trichlorobenze (neat) 135 °C (1 mg mL^−1^).

b Regioregularity (RR) estimated by ^1^H NMR.

c Corresponds to percentage of monomer 255 in the final polymer, measured by ^1^H NMR.

In 2017, Amsalu Efrem and co-workers developed a direct arylation polymerization (DArP) protocol for the synthesis of high performance, narrow bandgap donor–acceptor conjugated polymer 263, composed of alternating alkyl-quaternarythiophene and 5,6-difluoro-2,1,3-benzothiadiazole units.^278^ A series of DArP optimization led to the target molecule with a number average molecular weight (*M*_n_) of 14.6 kDa without noticeable β-branching effects and homocoupling.

**Scheme 77:**
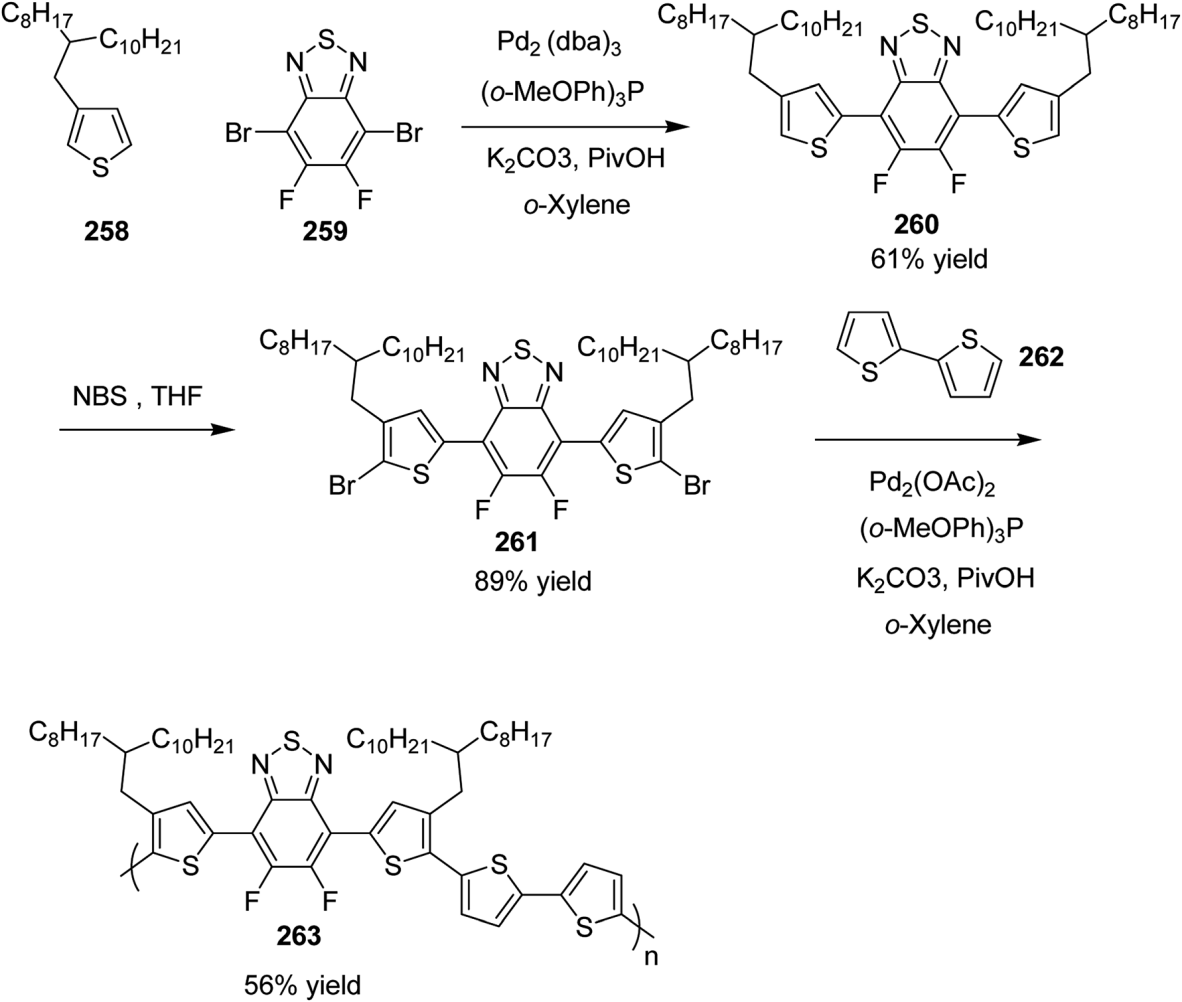
Synthesis of 263*via* DArP.

3-(2-Octyldodecyl)thiophene 258 was coupled with 4,7-dibromo-5,6-difluorobenzothiadiazole 259 to obtain 4,7-bis(4-(2-octyldodecyl)thiophen-2-yl)-5,6-difluoro[2,1,3]benzothiadiazole 260, which was further brominated with NBS to generate monomer 259, which was polymerized with 2,2′-bithiophene (BT) 262 to afford polymer 263. DArP protocol produced maximum *M*_n_ average when the reaction was conducted in the presence of Pd(OAc)_2_ using (*o*-MeoPh)_3_P as a ligand, K_2_CO_3_ as a base, PiVOH as an additive and *o*-xylene as a solvent at 100 °C for 72 hours. Alternating polymer structure and C–H selectivity of polymer 263, synthesized *via* direct arylation polymerization, are comparable to those of the same type polymers, prepared using Stille coupling, and despite of their lower molecular weight as compared to the polymers synthesizes *via* Stille coupling polymerization, showed better performances in OPV and OFET devices tested in air without any device encapsulation (Scheme 77).

In 2015, Thomas Bura and co-workers showed for the first time that unprotected thiophene units could be selectively and efficiently polymerized through direct (hetero) arylation polymerization (DHAP), employing an appropriate palladium source additive and ligand.^279^ This approach was applied for the synthesis of well-defined poly(3,3′′′-didodecyl-2,2′:5′,2′′:5′′,2′′′-quaterthiophene) from 5-bromo-3,3′′′-didodecyl-2,2′:5′,2′′:5′′,2′′′-quaterthiophene (monomer 268) and 5-bromo-3′,4′′-didodecyl-2,2′:5′,2′′:5′′,2′′′-quaterthiophene (monomer 274). Condition optimization using different ligands, catalysts and additives revealed that the use of Herrmann–Beller catalyst and P(*o*-NMe_2_Ph)_3_ ligand in the presence of bulky acidic additive (neodecanoic acid) could lead to selective thiophene–thiophene couplings. Steric protection around unsubstituted β-positions of α-bromothiophene units is needed to obtain a good selectivity of the cross-couplings at the α-positions. This can be achieved either in the presence of a substituent at an adjacent β-position or the utilization of a bulky acidic additive in the catalytic system.

**Scheme 78:**
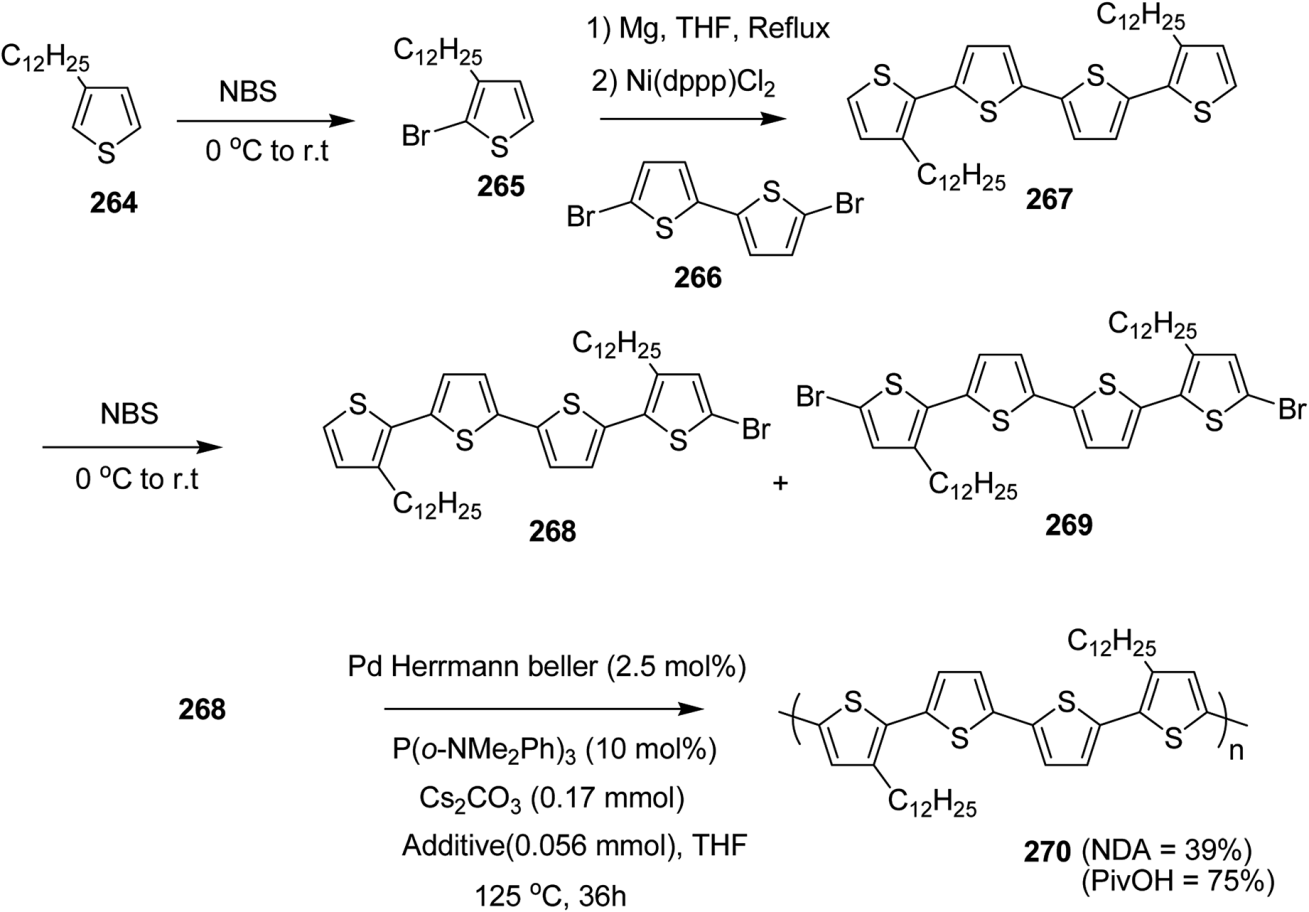
Synthesis of 268 and its polymerization to yield 270, applying Herrmann–Beller catalyst.

**Scheme 79:**
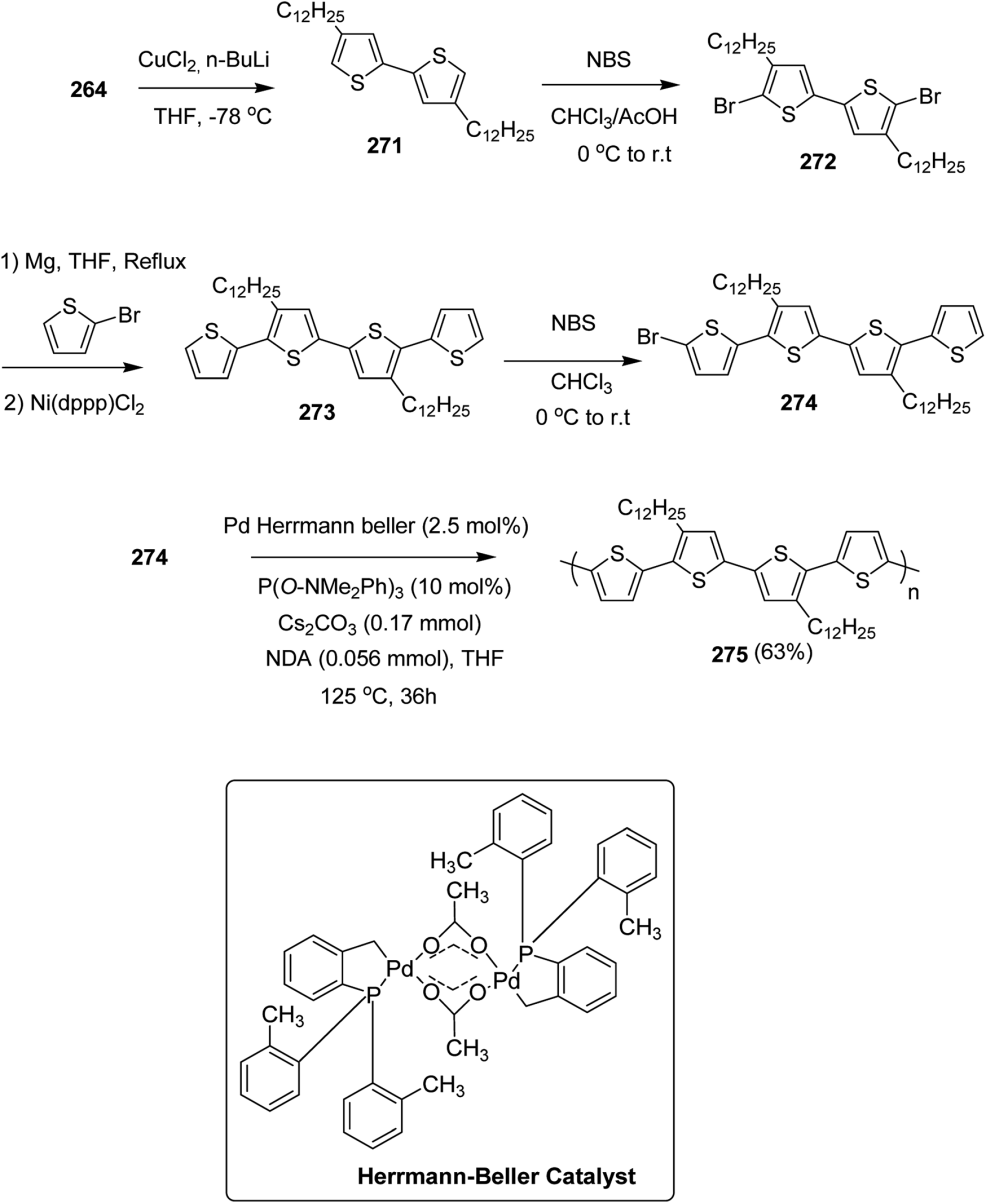
Synthesis of 274 and its polymer 275, applying Herrmann–Beller catalyst.

3-Dodecylthiophene 264 was brominated to afford 2-bromo-3-dodecylthiophene 265, which, upon reaction with 2-bromo-5-(5-bromothiophen-2-yl)thiophene 266, yielded compound 267. It was than brominated to yield monomer 268, which was polymerized using Pd Herrmann–Beller catalyst to afford polymer 269. 271 was obtained from 264 upon treatment with *n*-BuLi and CuCl_2_. Brominartion with NBS gave 272, which was coupled with 2-bromothiophene to afford 273. It was mono brominated to synthesize monomer 274, polymerization of which, in the presence of Pd Herrmann–Beller catalyst, gave 275 (Schemes 78 and 79).

Polymers synthesized using DHAP strategy showed essentially the same thermal and optical properties and are comparable to those observed with their analogues, obtained from Stille coupling and oxidative polymerization, while several synthetic steps were skipped compared to traditional Stille and Suzuki polymerization since the use of stannyl and boronate (toxic waste) comonomers are no longer required in this method.

Shotaro Hayashi and co-workers in 2017 reported direct arylation polycondensation of β-unprotected chalcogen heteroles including thiophene, furan and selenophene under phosphine-free conditions.^280^ Phosphine-free polymerization is one of the low cost and green pathways towards the synthesis of π-conjugated polymers. Polycondensation of thiophenes and furans produced insoluble polymers, resulting from β-defects and network formation, when Pd(OAc)_2_ was employed as a catalyst. Carbon-supported palladium catalyst, Pd/C, was found to be effective in performing polymerization of thiophenes and furans. The solid-supported palladium catalyst increased the regioselectivity of the reaction at the α-position of thiophene and prevented the formation of β-defects.

**Scheme 80:**
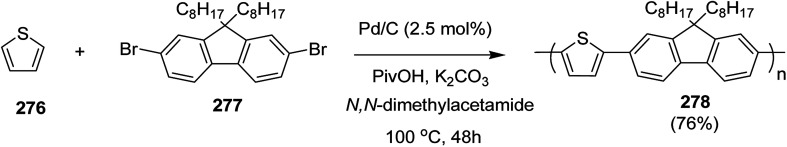
Direct arylation polycondensation of thiophene using Pd/C to synthesize 278.

For the polymerization reaction of thiophene 276 and 2,7-dibromo-9,9′-dioctyl-9*H*-fluorene 277, 2.5 mol% loading of Pd (OAc)_2_ furnished an insoluble product in 63% yield. Lowering the loading to 1.25 mol% yielded both soluble and insoluble products. Skipping the catalyst to Pd/C (2.5 mol%) resulted in the formation of soluble polymeric product 278 (*M*_n_ = 8600) in 76% yield. When loading of Pd/C was lowered to 1.25 mol%, soluble polymer with low molecular weight (*M*_n_ = 2900) was obtained in 29% yield. F8–F8 homo-coupled product was not detected (Scheme 80).

M. Wakioka and co-workers in 2017 reported the effect of mixed-ligand catalyst system towards Pd-catalyzed direct arylation polymerization.^281^ The combination of P(2-MeOC_6_H_4_)_3_ (L4) and *N*,*N*-tetramethylethylenediamine as a ligand effectively prevented defect formation in the polymerization of diketopyrrolopyrrole (DPP) units to give donor–acceptor copolymers.

**Scheme 81:**
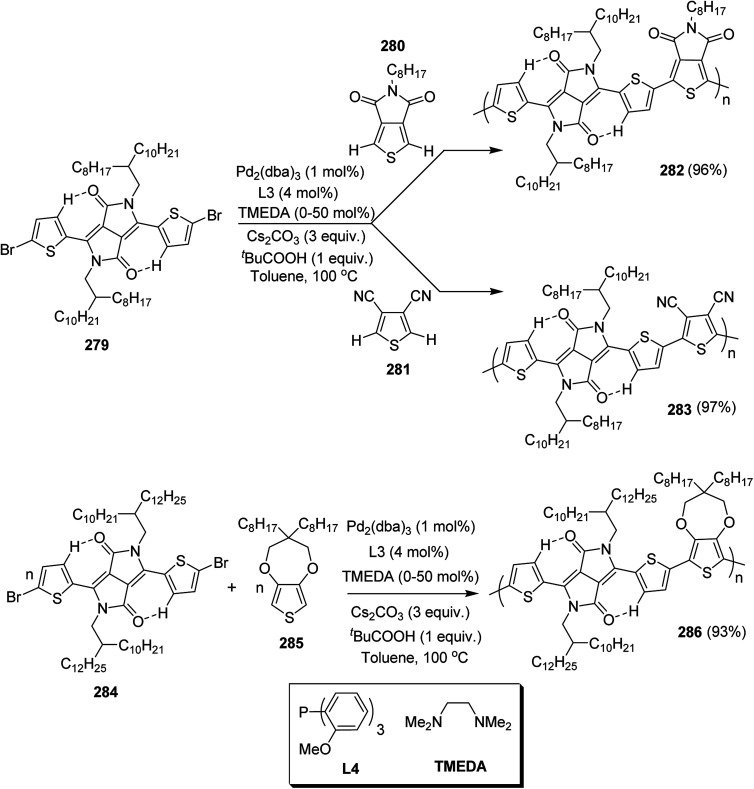
DArP of diketopyrrolopyrrole derivatives to synthesize 282, 283 and 286.

When 3,6-bis(5-bromo-2-thienyl)diketopyrrolopyrrole 279 was polymerized with 3,4-dicyanoyhiophene 280 in toluene at 100 °C for 6 hours in the presence of only L3, an insoluble material through cross-linking and branching and soluble part (*M*_n_ = 24 500 g mol^−1^), which included a large amount of homocoupling defects (12.5%), were obtained. Homocoupling defects decreased to 1.6% when L4 was used in combination with TMEDA. Use of this ligand mixture also resulted in complete suppression of the formation of insoluble material, and the molecular weight of the polymer 283 also increased remarkably (*M*_n_ = 24 500 g mol^−1^). This mixed-ligand approach was also proved to be effective for the polymerization of 280 with thienopyrrolidione 279 to yield 281 (*M*_n_ = 36 800 g mol^−1^) and 3,4-propylenedioxythiophene 285 to obtain 286 (*M*_n_ = 19 000 g mol^−1^), donor–acceptor polymers (Scheme 81).

**Scheme 82:**
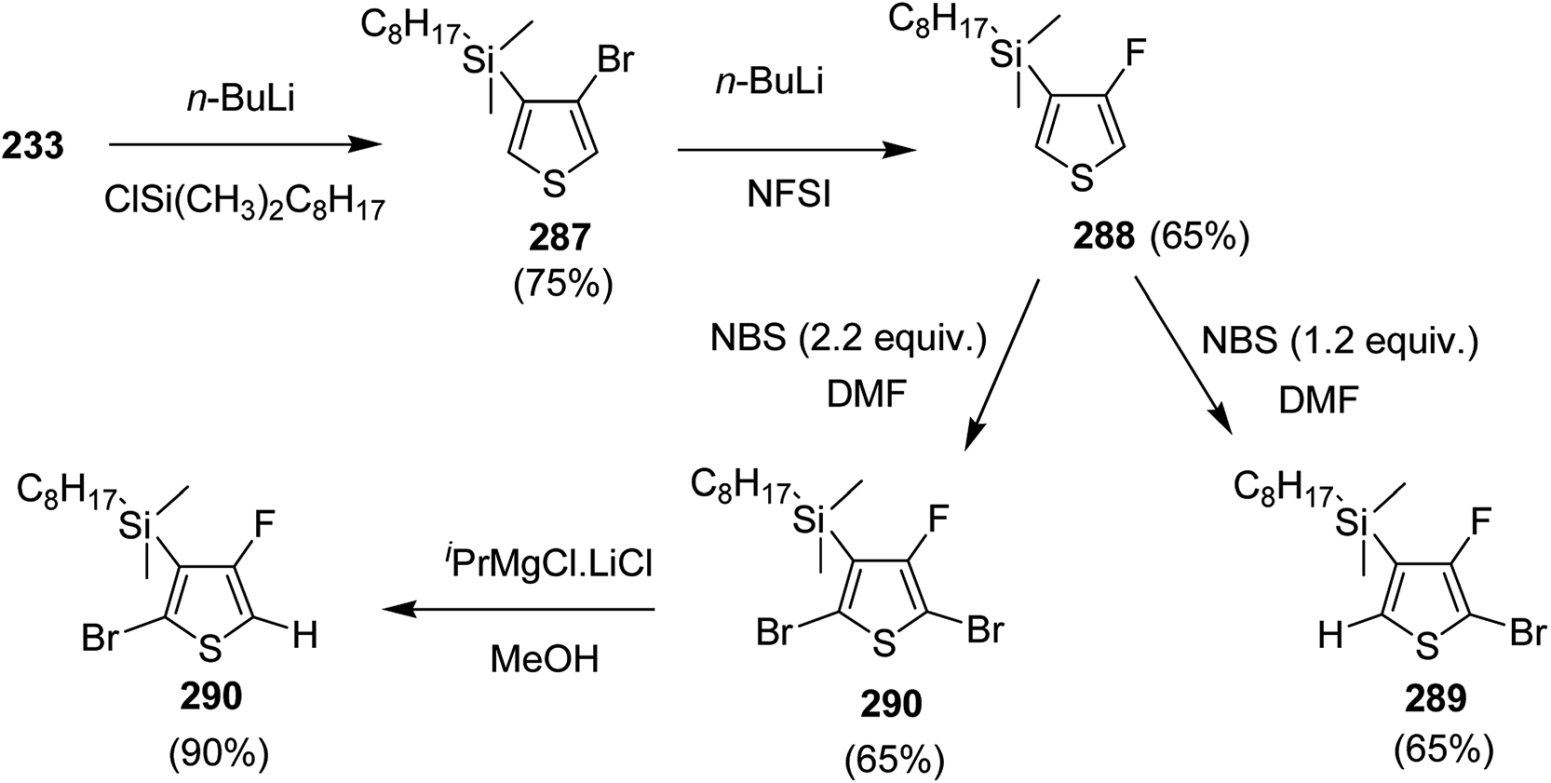
Synthesis of pivotal thiophene synthons 289 and 291 for the synthesis of fluorinated dithienobenzothiadiazole comonomers.

**Scheme 83:**
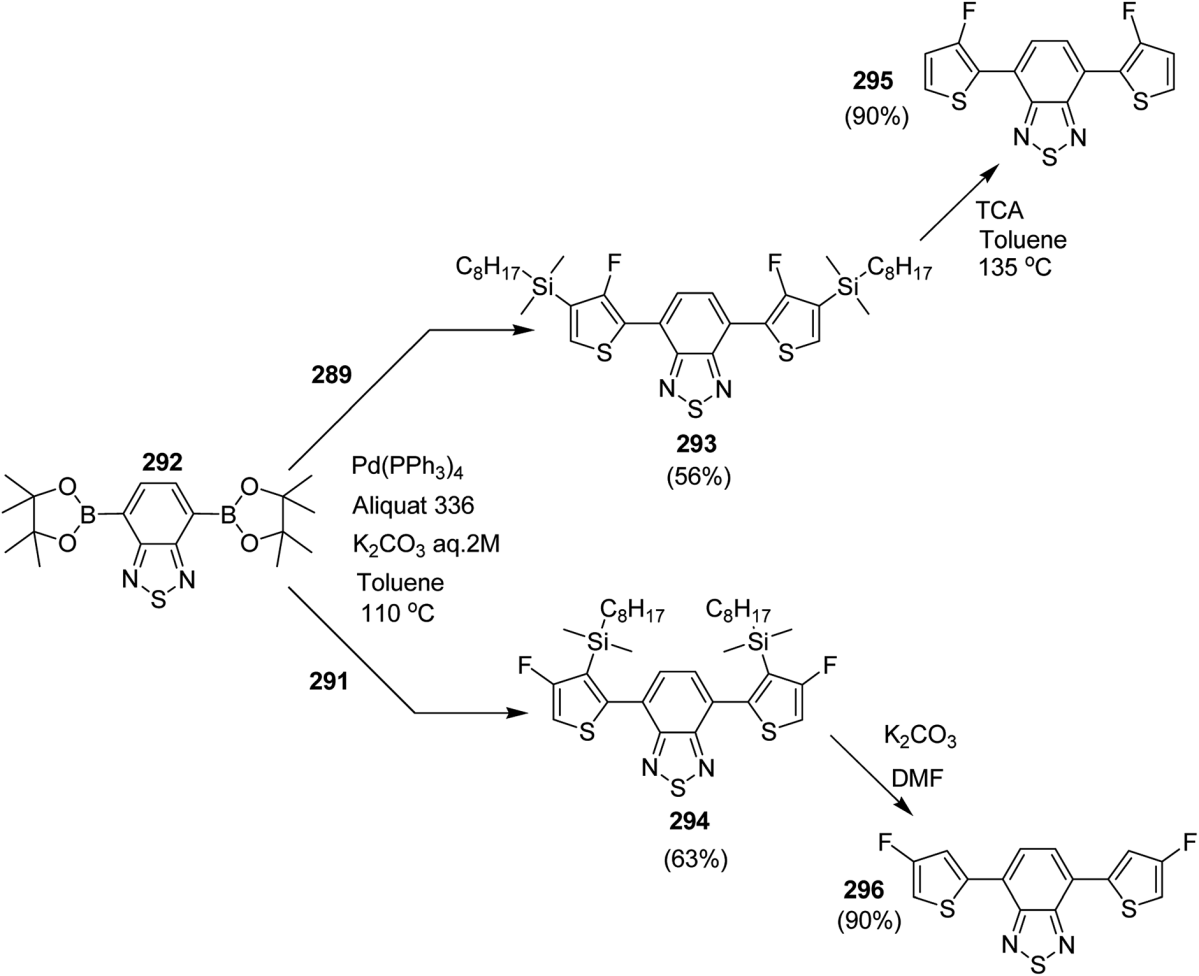
Synthesis of mono fluorinated monomers 295 and 296.

**Scheme 84:**
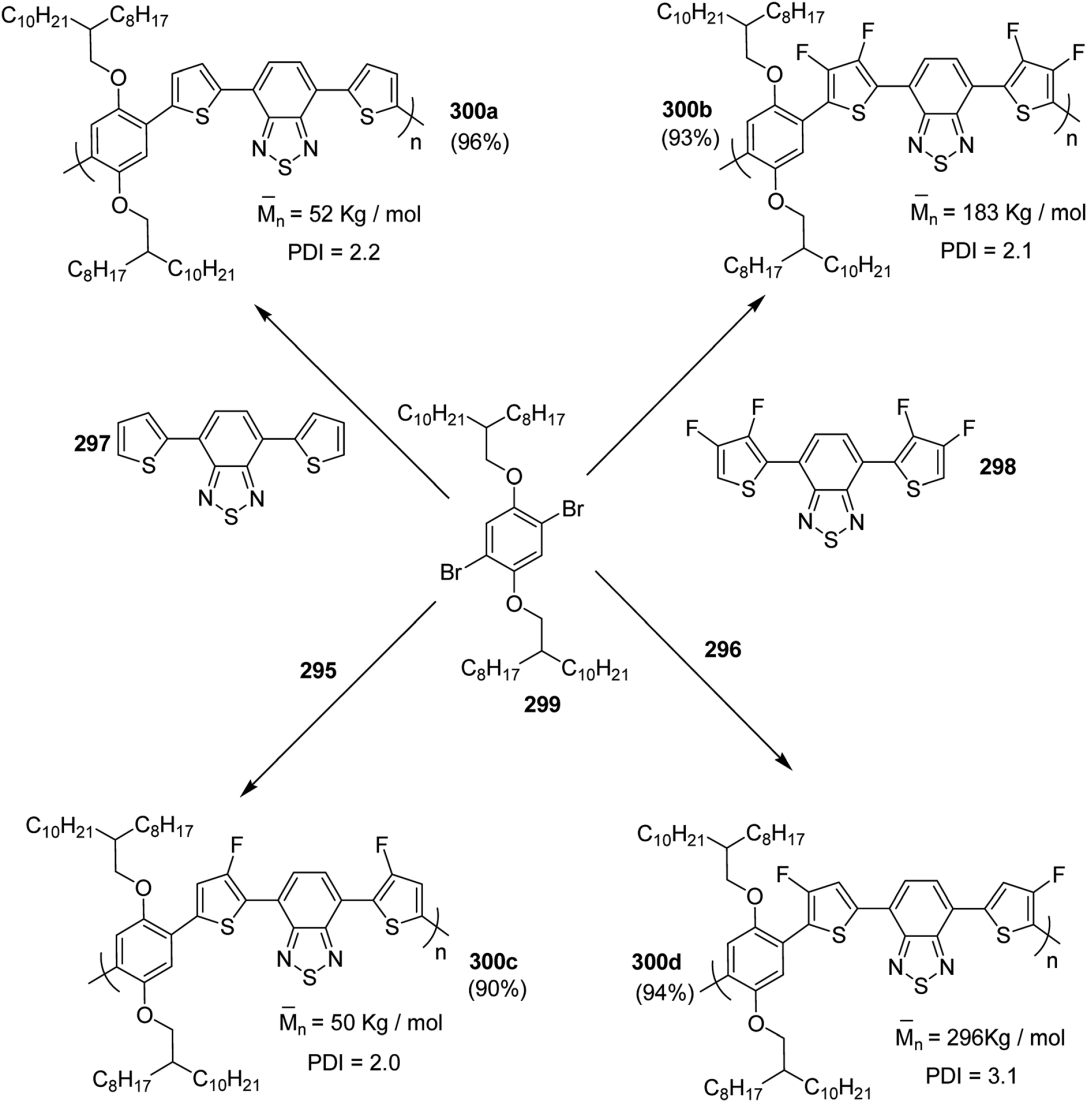
Synthesis of polymers 300a–c by DHAP. Conditions: Pd_2_dba_3_ (2% mol), (*o*-OMeC_6_H_4_)_3_P (8 mol%), Cs_2_CO_3_ (3 equiv.), and pivalic acid (1 equiv.), THF, microwave, 120 °C.

In 2017, Carl Roy and co-workers devised an efficient strategy for the synthesis and purification of novel mono-fluorinated derivatives of thiophene 289 and 291 for synthesizing fluorinated dithienobenzothiadiazole (DTBT) comonomers.^282^ It was observed that the amount and the position of fluorine atoms on the DTBT moiety could tune the regioselectivity and reactivity of direct (hetero) arylation polymerization (DHAP). Polymerization time for the fluorinated DTBT monomers reportedly decreased to only few minutes from 66 hour, required for the polymerization of non-fluorinated DTBT. Polymerization of the reaction mixtures containing 296 and 298 occurred rapidly (11 and 40 minutes, respectively) to give 300d and 300b, while 300a and 300c formed at slow rate (66 and 46 hours, respectively). 300d and 300b were found to be soluble in hot dichlorobenzene and 300a and 300c were soluble in DCM at room temperature. This difference in the reactivity of all the four monomers and their corresponding polymers is attributed to the difference in the amount and the position of fluorine installed on the flanking thiophene of the DTBT moiety (Schemes 82–84).

Colleen N. Scott and co-workers in 2017 reported the synthesis of three novel 2,5-dithienosilole with a series of difluorobenzodiimine-based acceptors, 5,6-difluoro[2,1,3]benzotriazole (DFBTA), 5,6-difluoro[2,1,3]benzothiadiazole (DFBT) and 5,6-difluorobenzoselenadiazole (DFBSe) using direct arylation polymerization reaction, which was considered as the first known example of synthesis of 2,5-dithienosilole-based polymers.^283^ Only oligomers with low molecular weight were yielded when Stille cross-coupling polycondensation reaction was performed to check the structural quality of the polymers, demonstrating the power of DArP protocol to synthesize polymers consisting of strongly electron-deficient monomeric units. Newly synthesized polymers 306a–c had reduced band gaps (<2.0 eV), and the hole mobility values in the range of 10^−2^ cm^2^ (V s)^−1^ was provided by polymer 306a, which was superior compared to the previously reported values for the 2,3-dithienosilyl-based polymers (10^−4^ to 10^−6^ cm^2^ (V s)^−1^) and comparable to the dithienosilole-based polymers.

**Scheme 85:**
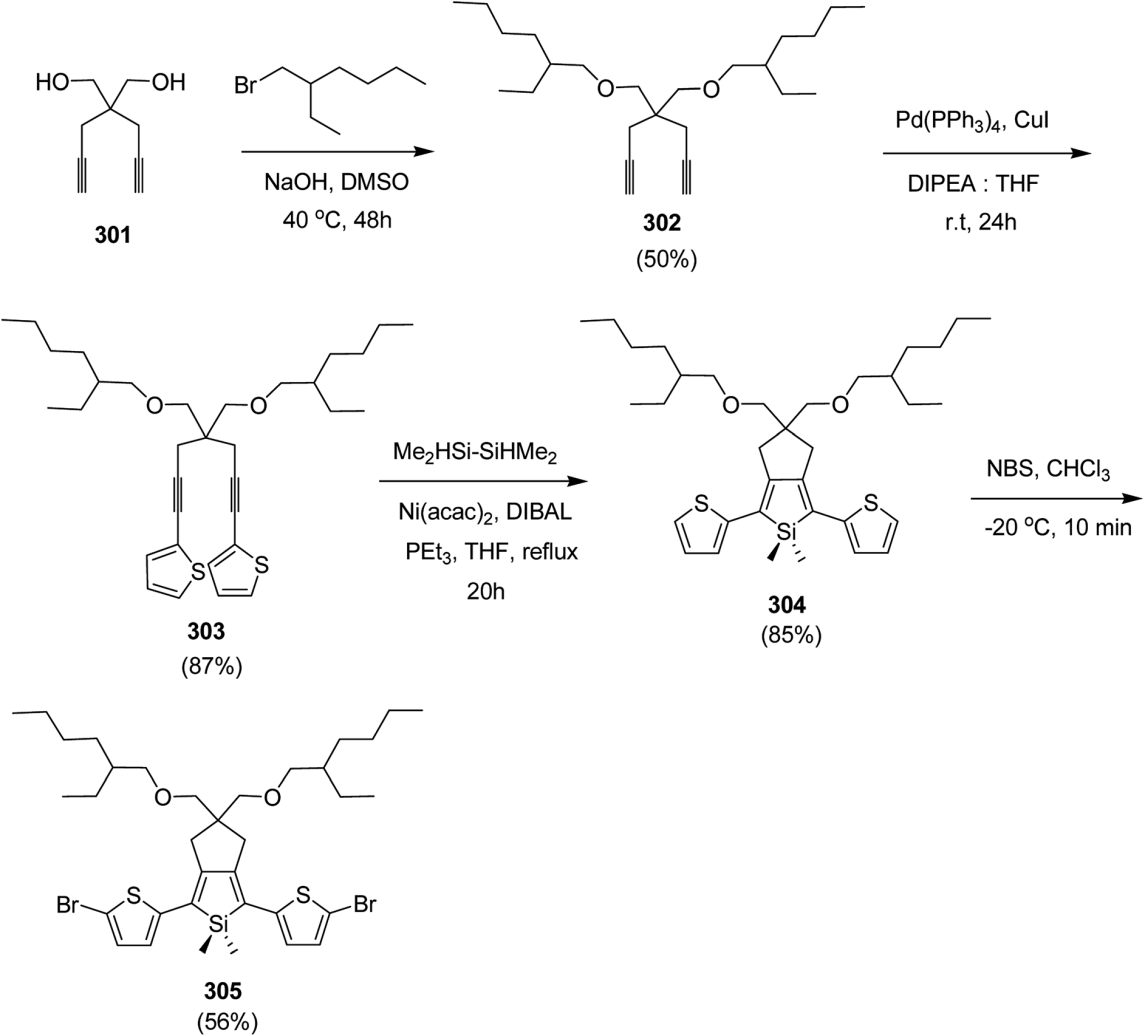
Synthesis of 2,5-dithienylsilole monomer 305.

Diol 301 was alkylated using 1-bromo-2-ethylhexane to yield compound 302, which was then capped with thiophene by Sonogoshira reaction in the presence of 2-bromothiophene to afford compound 303. Silol ring structure 304 was formed by a nickel mediated Kumada-type intramolecular cyclization, which was followed by bromination with NBS to yield monomer 305 (Scheme 85).

**Scheme 86:**
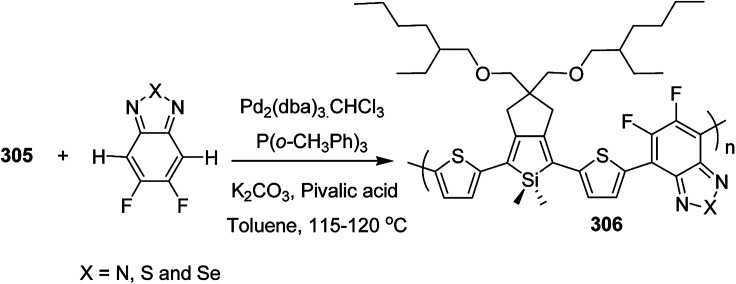
Synthesis of 2,5-dithienylsilole–difluorobenzodiimine polymers 306a–c by DArP reaction.

Polymers 306a–c, with moderate molecular weight, were obtained using Pd_2_(dba)_3_·CHCl_3_ catalyst, phosphine ligand and K_2_CO_3_ with pivalic acid. Relative molecular weights are in Table 12, which were measured by size exclusion chromatography using polystyrene as a standard. The much lower molecular weight of 306c could be the result of selenium (Se) atom inhibition of polymerization, presumably due to the coordination of Se atom with the Pd centre^284^ (Scheme 86).

**Table 12:** Recorded molecular weights of the polymers

Polymer	Structure	*M*_n_	*M*_w_	*Đ*	DP	Yield (%)
306a	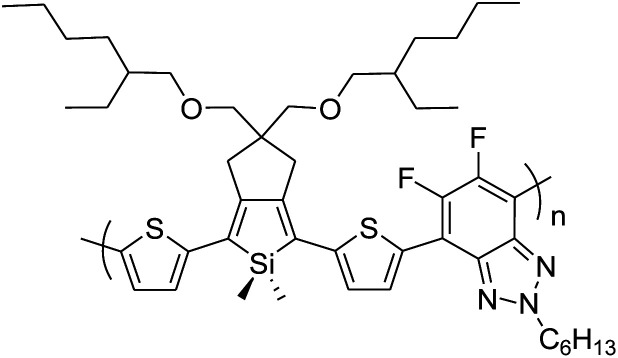	10 000	15 000	1.54	11	65
306b	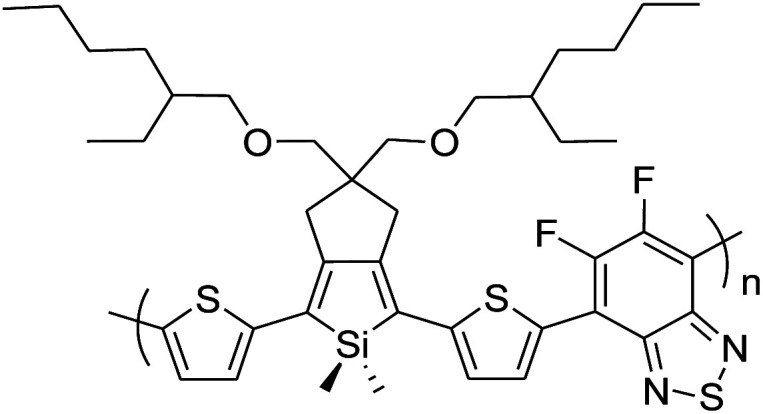	13 000	26 000	2.04	16	80
306c	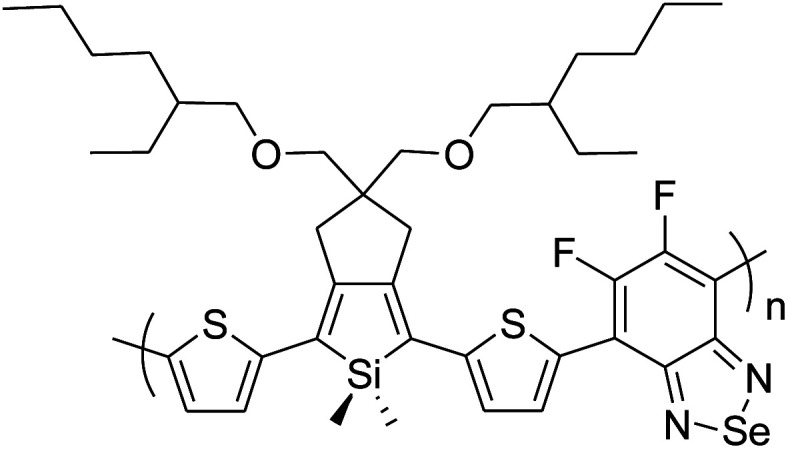	8000	11 000	1.30	10	70

**Scheme 87:**
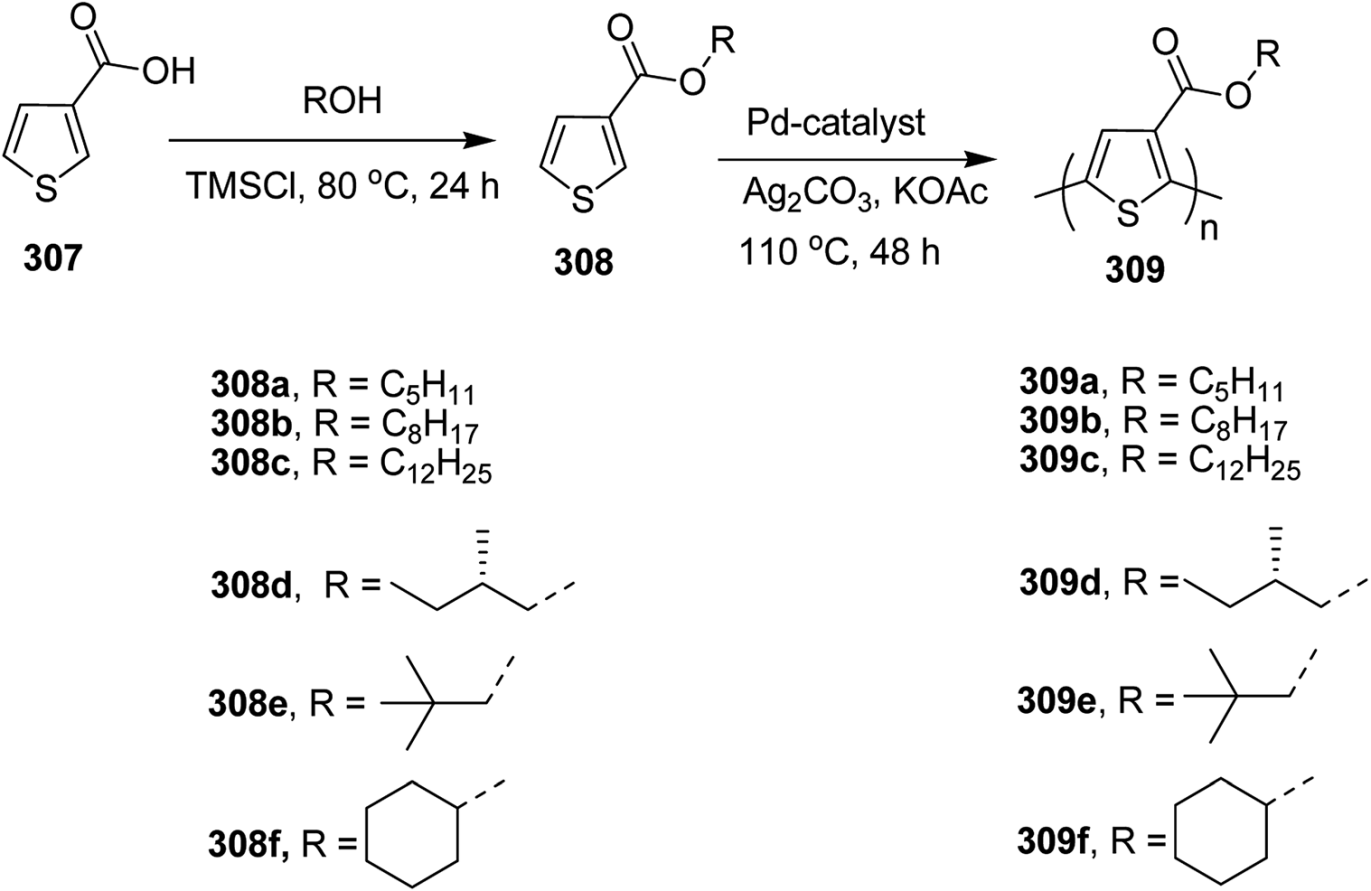
Synthetic route for poly(3-alkylesterthiophenes) 309a–f.

In 2018, Ludi Deng and co-workers synthesized a series of poly(3-alkylesterthiophenes) (P3ET) with high head-to-tail (HT) coupled regioregularity upto ∼92% *via* direct heteroarylation polymerization.^285^ The role of the alkyl-ester side chains on the C–H coupling polymerization as well as their effects on the optical and electrochemical properties and crystallinity were investigated by changing the length and size of the alkyl groups. It was found that higher head-to-tail regioregularity was obtained with larger alkyl ester side chains on position 3 of the thiophene monomers. All P3ET products 309a–f were found to have low crystallinity and particularly lacked order in the π-stacking direction in the solid state due to the steric hindrance of ester substituents. The lower ionization potential of the poly(3-alkylesterthiophenes) 309, compared to the corresponding poly(3-alkylthiophene), suggested that they could be potential candidates for p-type materials. Synthesis of the monomer was started by addition of suspension of thiophene-3-carboxylic acid 307 in anhydrous alcohol, with a subsequent addition of trimethylsilylchloride (TMSCl) *via* syringe. The reaction mixture was stirred at 80 °C for 24 hours. For polymerization, potassium acetate (KOAc), silver carbonate (Ag_2_CO_3_) and DMAc were added to the monomers 308a–f, and the reaction mixture was stirred at 110 °C for 10 minutes, then degassed with argon, which was followed by addition of Pd-catalyst and stirring at 110 °C for 48 hours to afford poly(3-alkylesterthiophenes) 309a–f (Scheme 87).

Various palladium catalysts were employed to examine their influence on direct arylation polymerization reaction for the synthesis of P3ET derivatives. Effect of catalyst types on the yield, polydispersity index, regioregularity and molecular weight of the resultant poly(3-octylesterthiophene) 309b, are displayed in Table 13. Yield, regioregularity, molecular weight and dispersity for DCHCP on poly(3-alkylesterthiophenes) are given in Table 14.

**Table 13:** Effect of catalysts on yield, polydispersity index (*Đ*), HT regioregularity and molecular weight of poly(3-octylesterthiophene) (309b) synthesized by DCHCP^a^

Catalyst	Yield^b^ (%)	*M*_n_ c (kDa)	*Đ* c	HT^d^
Pd(OAc)_2_	64	6.8	1.70	65
Pd(dppf)Cl_2_	80	6.6	1.61	86
Herrmanns' Cat.	73	7.0	1.77	81
Pd(dba)_2_	80	12.4	2.82	82
Pd(PhCN_2_)Cl_2_	64	7.5	1.67	83
Pd(PPh_3_)_4_	67	17.3	3.45	70

a Reaction conditions: substrate (1 equiv.), catalyst (0.05 equiv.), Ag_2_CO_3_ (2.0 equiv.), KOAc (2.0 equiv.) in 2 mL of DMAc at 110 °C for 48 h.

b Isolated yield after purification.

c Estimated by SEC measurements (eluent: THF, standard: polystyrene).

d Estimated by ^1^H NMR.

**Table 14:** Yield, regioregularity, molecular weight and dispersity for DCHCP on poly(3-alkylesterthiophenes)

Sample	Yield^a^ (%)	*M*_n_ b (kDa)	*Đ* b	*M*_n_ c (kg mol^−1^)	HT^d^ (%)
309a	90	10.4	2.01	6.2	89
309b	89	18.0	2.35	7.2	90
309c	90	5.1	1.31	3.6	92
309d	97	15.3	2.42	6.8	82
309e	80	6.0	1.76	4.1	84
309f	97	7.8	1.06	4.6	92

a Isolated yield after purification.

b Estimated by SEC measurements (eluent: THF, standard: polystyrene).

c Calculated from the terminal groups of polymer in ^1^H NMR.

d Estimated by ^1^H NMR.

In aforementioned examples of palladium-catalyzed synthesis of thiophene-based polymers, polymerization proceeded *via* step-growth mechanism. Developing a palladium-catalyzed synthesis proceeding through chain-growth polymerization could be more effective in controlling molecular weights and dispersities of the polymers. Development of dual metal catalysis with orthogonal reactivity could be an effective pathway for this purpose. Luscombe's research group presented their findings for living polymerization of poly(3-hexylthiophene) in 2016. Sabin-Lucian Suraru and co-workers reported dual metal catalysis using gold and palladium.^286^ Initially, gold was used for the C–H activation of the monomer, 2-bromo-3-hexylthienyl-5-gold 310, which then underwent palladium catalyzed cross coupling *via* chain growth polymerization to yield regioregular polythiophene without the use of sensitive organometallic reagents based on base metals such as Grignard reagent.

**Scheme 88:**
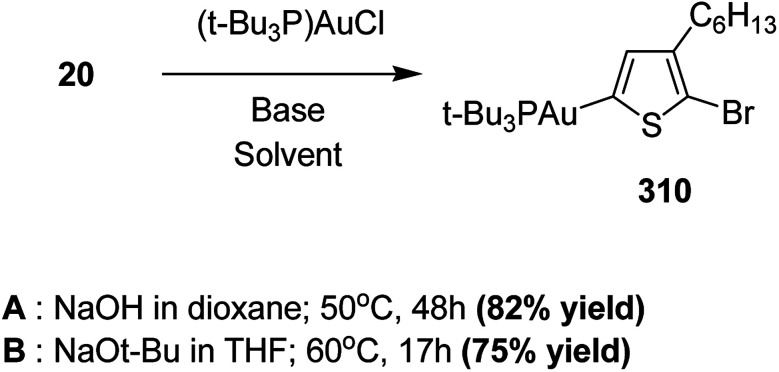
Synthesis of monomer 2-bromo-3-hexyl-thienyl-5-gold 310 by C–H activation.

Auration of 2-bromo-3-hexylthiophene 20 occurred by reacting it with chloro(tri-*tert*-butylphosphine)gold(i) using grounded NaOH in dioxane at 50 °C for 48 hours to obtain the desired monomer 310 in 82% yield. Moreover, the same reaction was also performed in the presence of THF and NaO*t*-Bu in order to match the reaction conditions of C–H activation with that of chain growth polymerization of P3HT 16 more closely (Scheme 88).

**Scheme 89:**
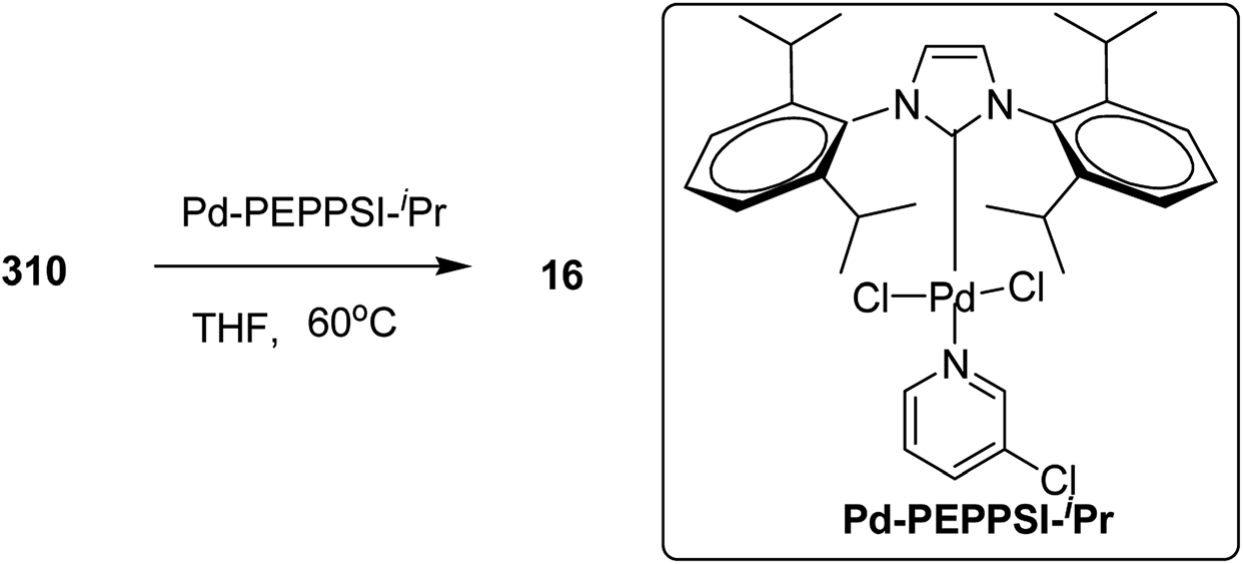
Polymerization of Au-thiophene monomer 310 to yield P3HT 16.

Organo gold monomer 310 was polymerized at 60 °C in THF to provide good solubility to the monomer and avoid precipitation of the polymeric product during the course of the reaction. The use of Pd-PEPPSI-iPr catalyst resulted in a very good yield, while Pd(PPh_3_)_2_Cl_2_ and combination of Pd_2_(dba)_3_ with dppp or PtBu_3_ did not show good activity and resulted only in the formation of short oligomers. This improved performance of PEPPSI catalyst compared to phosphine based ligands, was speculated to be the result of strong sigma donating character of N-heterocyclic carbenes (NHCs) as compared to phosphines (Scheme 89).

Polymerization step was performed by altering monomer-catalyst ratio and four different reactions were conducted using 1, 2, 3 and 5 mol% of Pd-PEPPSI catalyst. Results are given in Table 15, indicating a remarkable agreement with the theoretical molecular weight *M*^th^_n_, expected for a living chain growth polymerization where the Pd catalyst does not leave the growing polymer chain.

**Table 15:** Theoretical and observed *M*_n_ at various Pd-PEPPSI-iPr catalyst loadings

Entry	Pd-PEPPSI-^i^Pr (mol%)	*M* ^th^_n_ (g mol^−1^)	*M* ^gpc^_n_ (g mol^−1^)	*M* ^NMR^_n_ (g mol^−1^)	*M* ^NMR^_n_ a (g mol^−1^)	*Đ*
1	5	3300	3500	3000		1.01
2	3	5500	4100	4200	10 400	1.20^b^
3	2	8300	10 150	10 800		1.32
4	1	16 600	14 500	17 400		1.31

a A second equivalent of monomer was added before quenching to observe continuation of chain growth mechanism.

b Dispersity corresponds to the final polymer after second monomer addition. Before second monomer addition, *Đ* = 1.04.

## 5. Palladium-catalyzed Negishi coupling polymerization

In 2018, Marie-Paule Van Den Eede and co-workers investigated the chiral expression and supramolecular organization in tailor-made conjugated polymers P3Ats, which were synthesized using Pd-RuPhos protocol.^287^ Following two new parameters, effect of the chiral expression and supramolecular organization were determined.

(1) End-groups are required to break the symmetry in block copolymers comprising (*R*)-chiral and (*S*)-chiral blocks of equal lengths. Polymer chains did not aggregated without an end-group.

(2) The supramolecular aggregation is completely disrupted if one single head-to-head (HH) coupling is present in regioregular poly(thiophene). This observation was in contrast to the presence of one tail-to-tail (TT) defect.

**Scheme 90:**
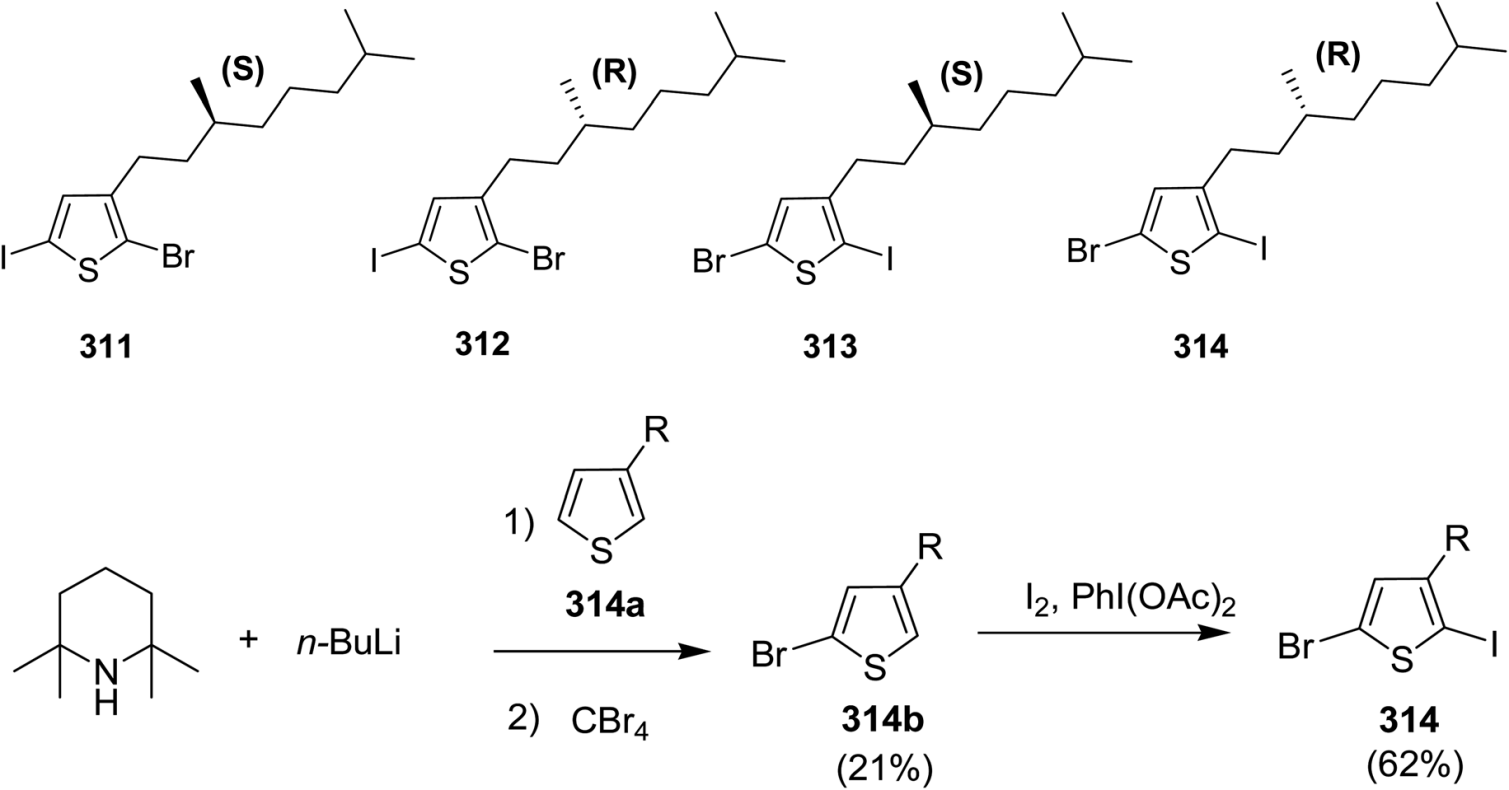
Synthesis of monomer 314.

While the synthesis of the monomers 311–313 were available in the literature,^288,289^ the monomer, 314 was synthesized for the first time. For this synthesis, *in situ* prepared tetramethylpiperidinyllithium was employed to deprotonate 314a at position 5 and subsequent quenching with CBr_4_ yielded 314b. PhI(OAc)_2_ and I_2_ were added to the solution of 314b in DCM to yield 314 (Scheme 90).

All the polymers were prepared *via* Negishi coupling polymerization. For the synthesis of polymers 316–321, initially, a polymerization method not incorporating an end group different from the first bolck's monomeric unit was required. Since Kumada catalyst transfer polymerization of thiophene block copolymers using a standard external *o*-tolyl initiator resulted in block copolymers with an *o*-tolyl end group, another external initiator was needed.^290–295^ Thus, for the polymers 320 and 321 different reaction method was applied as an HH coupling needs to be formed in the beginning of the second block. Kumada catalyst transfer polymerization based on Ni(dppe) or Ni(dppf) catalyzed HH-coupling at much slower rate compared to HT-couplings,^295^ which would result in the mixture of homopolymers of the first block and diblock copolymers with a very long second block. However, Pd-RuPhos polymerization based on deactivation of monomer was developed.^296^ It has also been demonstrated that HH-couplings could form easily and at a rate similar to the HT-couplings. The origin of the controlled nature of this polymerization was deactivation of the monomer, which could dissociate from the growing polymer, leaving a Br-terminated chain. Reinsertion resulted in a further growth.

**Scheme 91:**
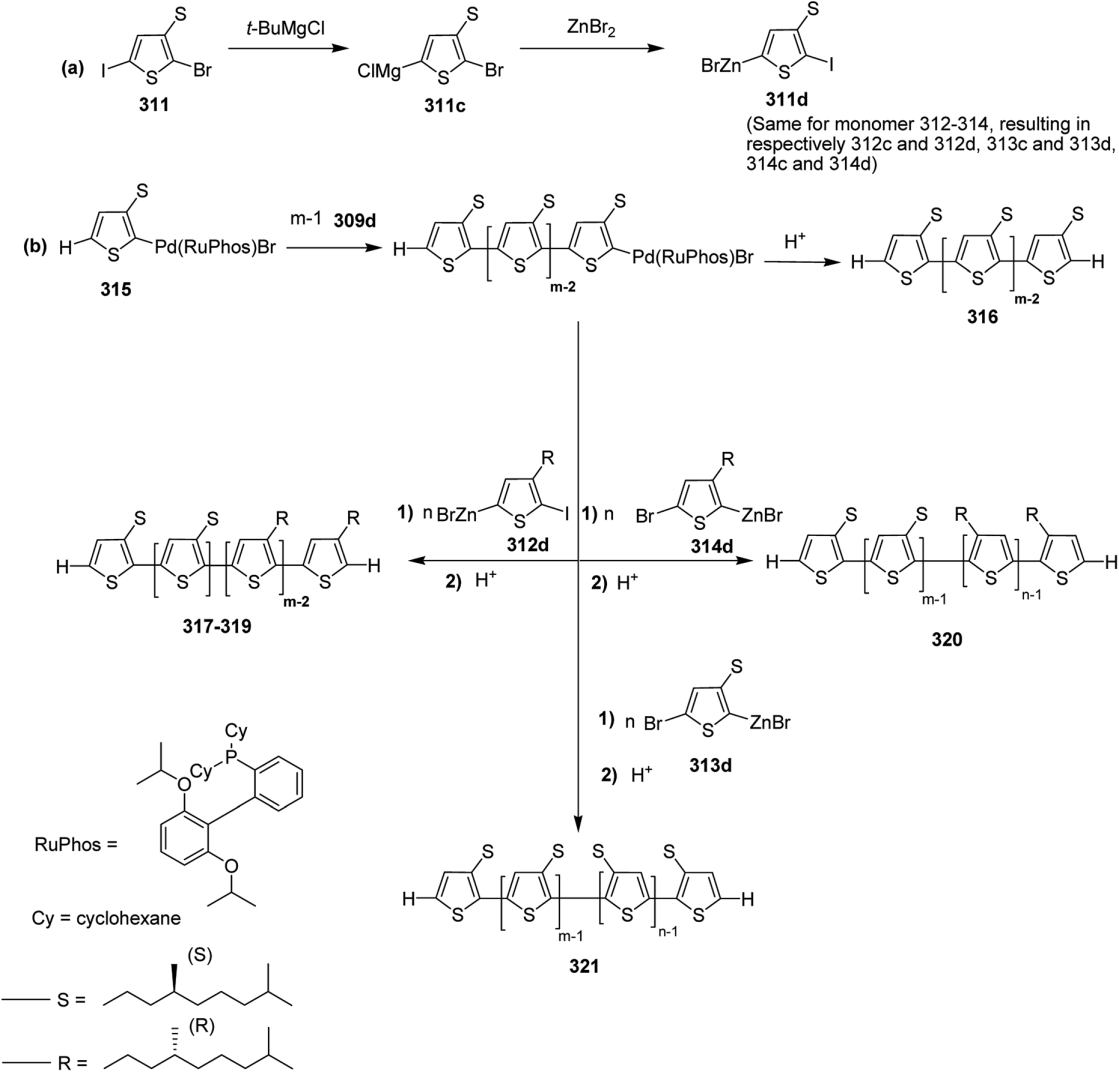
(a) Grignard metathesis and transmetalation of 311–314, (b) Pd-RuPhos polymerization of 316–321 with initiator 315.

For the polymerization; solution of monomer 311 underwent Grignard metathesis (GRIM) reaction with ^*t*^BuMgCl for 60 minutes. Reaction of 311a with ZnBr_2_ solution yielded organozinc monomer 311b, which was added to the solution of initiator 315 and polymerized for 50 min. Acidified THF was used to terminate this polymerization to obtain polymer 316. Predetermined quantities of precursor monomer 312 were transformed into the corresponding organozinc compound 312b to synthesize polymers 317–319. 312b was added to the first (*S*)-chiral block. These polymerizations were also terminated by acidified THF after 50 min. For the synthesis of polymers 320 and 321, same procedure was followed as for 317–319, but with the precursor monomers 314 and 313 for the formation of the second block (Scheme 91). An overview of *M*_n_ and *Đ* of the polymers 316–321 is given in the Table 16.

**Table 16:** An overview of *M*_n_ and *Đ* of polymers obtained by GPC, the degree of polymerization DP calculated from the GPC data, and the DP *m* and *n* obtained by ^1^H NMR

Polymer	*M*_n_ (kg mol^−1^)	DP	*Đ*	*m*	*n*	% (*S*)–% (*R*)
314	4.2	19	1.5	18		100% (*S*)–0% (*R*)
315	3.5	16	1.5	14	2	88% (*S*)–12% (*R*)
316	3.6	16	1.4	12	4	75% (*S*)–25% (*R*)
317	4.2	19	1.4	9	9	50% (*S*)–50% (*R*)
318	4.8	22	1.3	11	11	50% (*S*)–50% (*R*)
319	4.9	22	1.4	11	12	48% (*S*)–52% (*R*)

## 6. Synthesis of thiophene-based polymers *via* combination of Suzuki, Stille and direct arylation polymerization

There are few polymers that could be synthesized using three different protocols, namely Suzuki, Stille and direct arylation polymerization (DArP) reaction. In 2014, Ichiro Imae and co-workers reported the synthesis of poly(quinquethiophene), that is poly(3,3′′′-dihexyl-3,4′,3′′′,4′′′-diethylenedioxy-2,2′:5′,2′′:5′′,2′′′:5′′′,2′′′′quinquethiophene),partially containing 3-hexylthiophene and 3,4-ethylenedioxythiophene (EDOT), *via* Suzuki, Stille and DArP protocol.^297^ Among them, direct C–H coupling reaction produced the polymer with the highest molecular weight. The polymer was soluble in common organic solvents and a significant red shift was observed with fluorescence and absorption spectra compared to the corresponding monomeric unit due to enhancement of π-conjugation length.

**Scheme 92:**
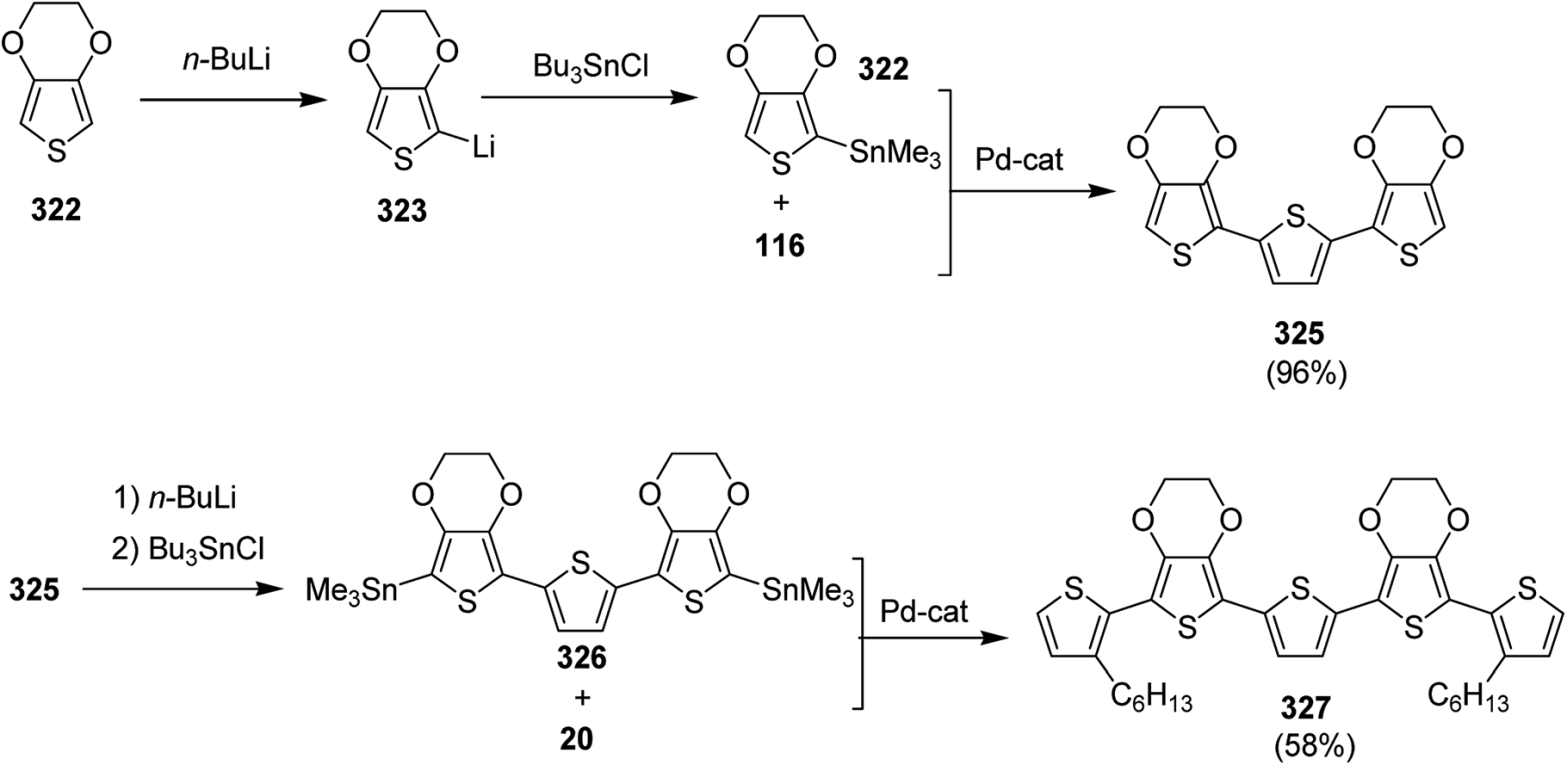
Synthesis of 325 and 327.

In order to synthesize 325, *n*-butyllithium was added to the solution of 3,4-ethylenedioxythiophene 322 in THF at −78 °C to obtain lithiated 3,4-ethylenedioxythiophene 323, which was followed by addition of tri(*n*-butyl)tin chloride to afford 322. The product 322 was then added to a solution of 2,5-dibromothiophene and Pd(CH_3_)_4_ to yield 325. *n*-Butyllithium was added to a solution of 325 in THF at −78 °C, followed by a subsequent addition of tri(*n*-butyl)tin chloride at −78 °C to afford 326, which was added to a solution of 2-bromo-3-hexylthiophene 20 to afford 327 (Scheme 92).

**Scheme 93:**
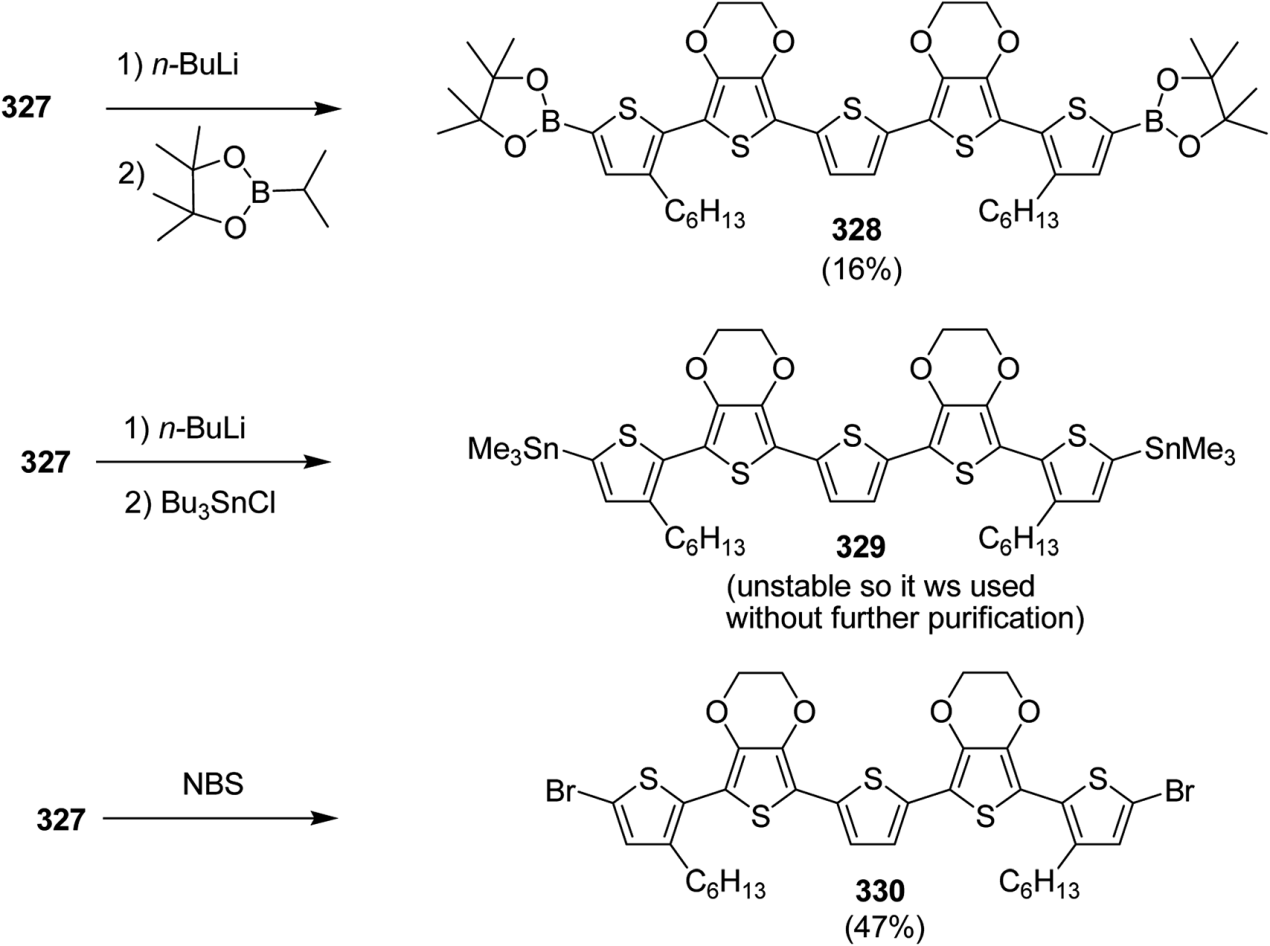
Synthetic route of the monomers 328–330.

Monomer 328 was synthesized by adding *n*-butyllithium to the solution of 327 and stirring the resulting mixture for 1 hour at −78 °C, which was followed by a subsequent addition of 2-isopropoxy-4,4,5,5-tetramethyl-1,3,2-dioxaborolane and further stirring the reaction mixture at −78 °C to afford 328 monomer in 16% yield. Same sequence of steps was applied for the synthesis of monomer 329 by the addition of tri(*n*-butyl)tin chloride and was used for the polymerization reaction without any purification due to the instability of 329. Monomer 330 was obtained in 47% yield by addition of CHCl_3_/CH_3_COOH solution of NBS to HE5T in CHCl_3_/CH_3_COOH and purifying the residual product *via* column chromatography (Scheme 93).

**Scheme 94:**
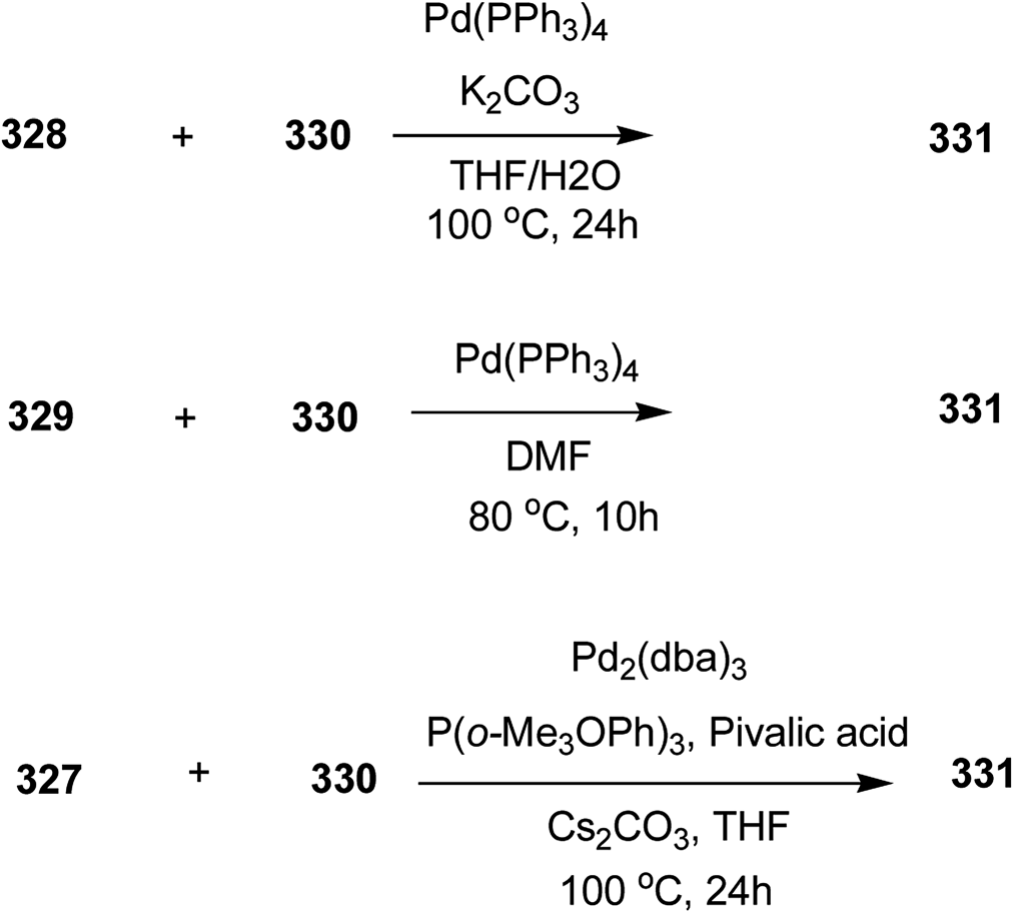
Three types of polycondensation strategies to synthesize 331.

In order to investigate the protocol yielding 327 with higher molecular weight, three types of polymerization reactions were applied to synthesize polymer 331. Application of direct arylation polymerization reaction resulted in higher molecular weight of polyHE5T (*M*_w_ = 63 000), while Suzuki and Stille coupling polycondensation reactions resulted in yielding low molecular weight polymer 331 with *M*_w_ = 22 000 and 28 000, respectively, which was reported to be due to difficulty in the purification of 328 and 329 arising from their low stabilities (Scheme 94).

In 2017, Shu-Wei Chang and co-workers reported the synthesis of double acceptor copolymers 338, 338-DA, 339, 339-DA, 340–342, 342-DA, 343, 343-DA, 344, 344-DA, 345 and 345-DA, consisting of thieno[3,4-*c*]pyrrole-4,6-dione (TPD), benzothiadiazole (BT) and two cyclopentadithiophene (CPDT) units, and a series of copolymers consisting of CPDT–A–CPDT (A = acceptor: BT or TPD) with different number of thiophene units, prepared by a combination of Stille and Suzuki coupling, oxidative and direct arylation polymerization.^298^ Hybridized features were demonstrated by the double acceptor copolymer due to the presence of both TPD and BT. Band gap for both BT and TPD series was increased by increasing the number of electron donor units. Higher OPV performance was exhibited by these donor–accepter alternating copolymers, compared to other polymers with power conversion efficiency of 3–4%. Absorption of wide-range visible light by the active layer consisting of double acceptor polymers and fullerene derivatives exhibited subtractive color, found to be advantageous for the development of transparent building-integrated organic photovoltaics.

**Scheme 95:**
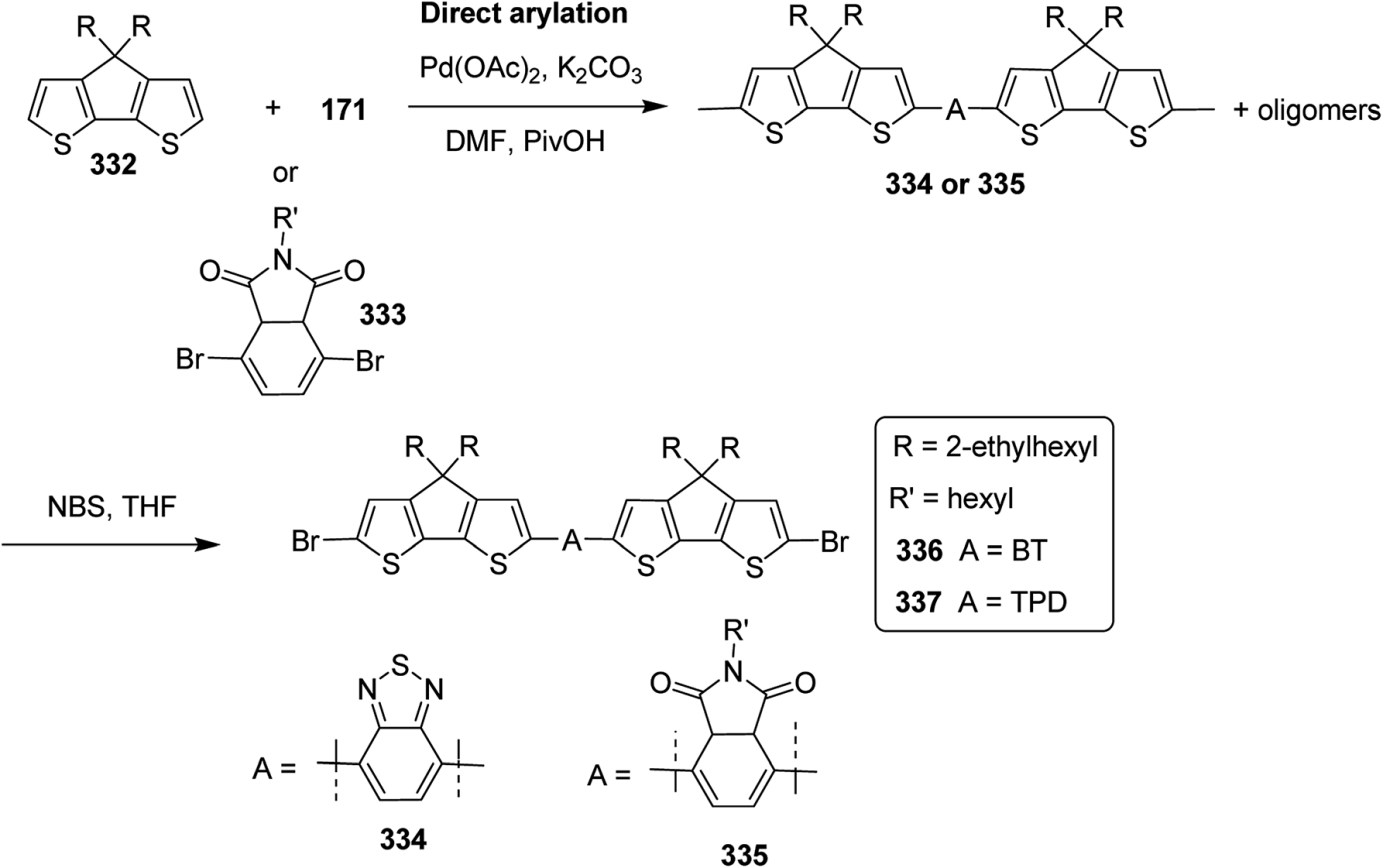
Synthesis of monomers 334–337.

**Scheme 96:**
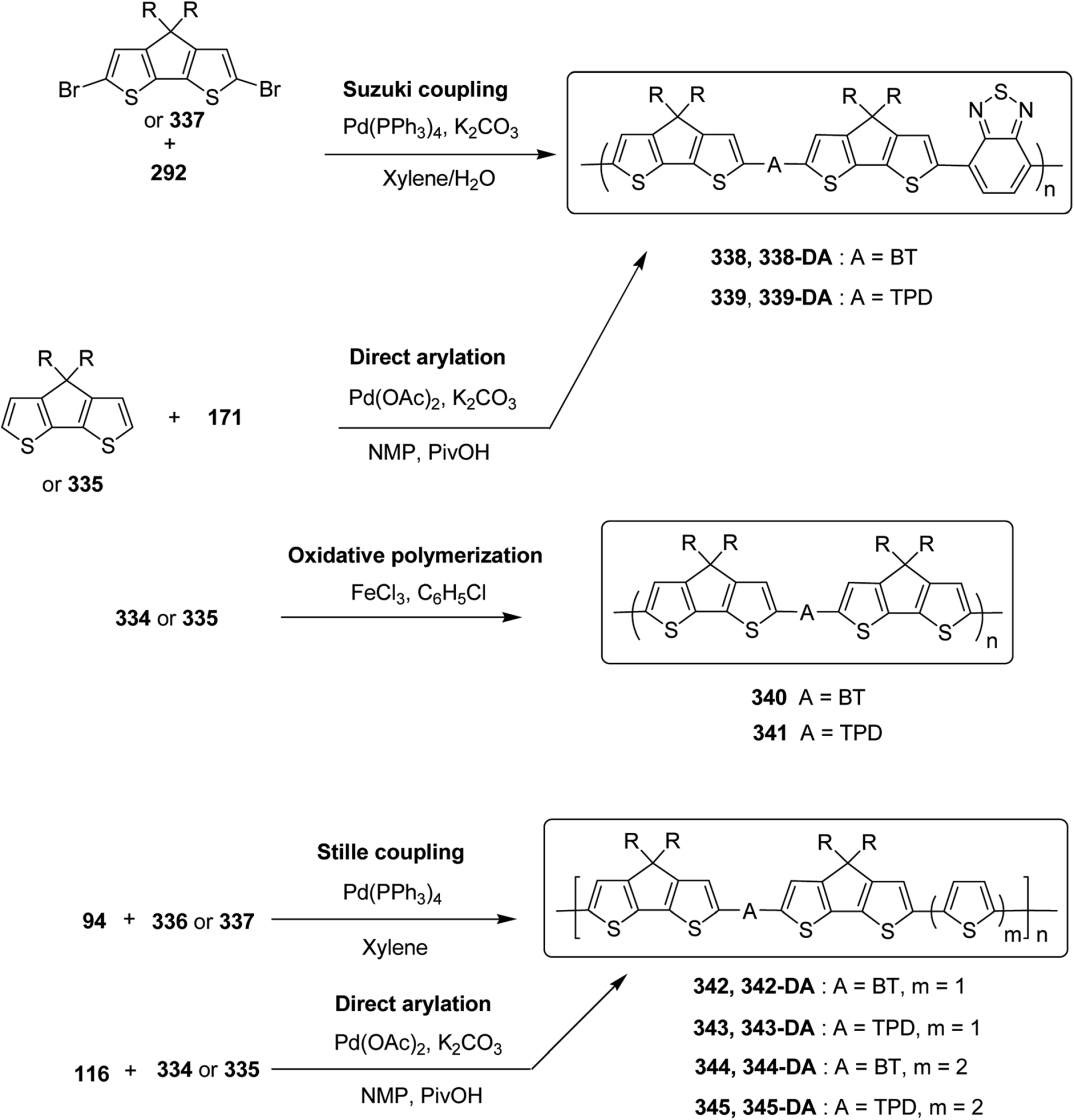
Synthesis of polymers 338, 338-DA, 339, 339-DA, 340–342, 342-DA, 343, 343-DA, 344, 344-DA, 345 and 345-DA, using Suzuki, Stille, oxidative and direct arylation polymerization techniques.

Monomers 334 and 335 consisting of two CPDT 332 and one TPD or BT units were synthesized by direct arylation of dibrominated BT 171 or TPD 333, using Pd(OAc)_2_, PivOH and K_2_CO_3_ in DMF for 2 hours at 80 °C. These monomers were further brominated with NBS to yield monomers 336 and 337 for subsequent use in Suzuki or Stille coupling polycondensation or direct arylation polymerization (Scheme 95). Results of the polymerization reactions are enlisted in Table 17. Direct arylation polymerization resulted in low molecular weight copolymers, which might be a result of low reactivity of 334 and 335 as compared to CPDT that resulted from the presence of electron-deficient TPD or BT units. Use of harsh reaction conditions such as increased temperatures and longer reaction times could lead to the formation of insoluble branched polymeric product due to the presence of multiple reactive protons on CPDT 332. Moreover, CPDT–BT–CPDT 334 showed higher molecular weight than their CPDT–TPD–CPDT 335 counter parts. The monomers containing carbonyl moiety were reported to be effective coupling partners in Stille reaction, while they are less effective in direct arylation due the presence of base in the reaction mixture, which would affect carbonyl groups on TPD (Scheme 96).

**Table 17:** Molecular weights and reaction yields of the polymers

Polymer	Group	Protocol	*M*_n_ a	*M*_w_/*M*_n_^a^	Yield^b^ (%)
336	BT/BT	Suzuki coupling	53 200	3.7	88
336-DA	BT/BT	Direct arylation	71 700	4.5	76
337	TPD/BT	Suzuki coupling	14 100	2.0	70
337-DA	TPD/BT	Direct arylation	8600	1.5	46
338	BT/none	FeCl_3_	51 300	3.7	76
339	TPD/none	FeCl_3_	52 900	2.4	50
340	BT/thiophene	Stille coupling	17 900	1.7	81
340-DA	BT/thiophene	Direct arylation	15 600	1.8	53
341	TPD/thiophene	Stille coupling	50 500	3.1	71
341-DA	TPD/thiophene	Direct arylation	5100	1.7	21
342	BT/bithiophene	Stille coupling	27 600	1.9	84
342-DA	BT/bithiophene	Direct arylation	15 600	1.8	23
343	TPD/bithiophene	Stille coupling	23 400	1.8	66
343-DA	TPD/bithiophene	Direct arylation	11 400	2.0	26

a Calculated from the gel permeation chromatography (GPC) measurements carried out using THF as a solvent and polystyrene as a standard. Polydispersity index and number-average molecular weight *M*_n_ after Soxhlet extraction.

b Yield after Soxhlet extraction.

## 7. Conclusion

Transition metal catalyzed polymerization has made tremendous contribution towards the synthesis of novel polymers of both isolated and fused ring thiophenes. Preferably, substituted thiophene monomers are used to enhance their solubility in organic solvents employed for their polymerization as well as characterization. Palladium and nickel-based catalysts have proved very helpful in the regioregular synthesis of homopolymers as well as block and copolymers of thiophene-based monomeric units with useful optical and conducting properties to be used in organic photovoltaics, field effect transistors, light emitting diodes *etc.* Dehydrobrominative C–H functionalization polycondensation and Kumada catalyst-transfer polymerization (KCTP) are extremely fast growing synthetic strategies that have shown a great control over the polymerization of thiophene-based monomers but still more effort is needed to explore their full potential for the polymerization of more complex monomers. Direct arylation polymerization (DArP) is being developed to combat the shortcomings of conventional methods used for polymer synthesis. DArP protocol is economical to adopt because it consists of lesser number of steps as it excludes the synthesis of stannyl and boronate comonomers to afford desired polymeric products with the same optical and thermal properties as of the polymers synthesized by Suzuki and Stille coupling reactions. Hence, it also avoids the formation of toxic byproducts during the proceedings of the reaction and uncomplicate the purification process of the final product. It has been more than twentyfive years since the first synthesis of regioregular polythiophene but this field is still growing and is believed to have a bright future especially in the area of plastic electronics.

## Conflicts of interest

There are no conflicts to declare.

## Supplementary Material
